# Serum reactome induced by *Bordetella pertussis* infection and Pertussis vaccines: qualitative differences in serum antibody recognition patterns revealed by peptide microarray analysis

**DOI:** 10.1186/s12865-015-0090-3

**Published:** 2015-07-01

**Authors:** Davide Valentini, Giovanni Ferrara, Reza Advani, Hans O Hallander, Markus J Maeurer

**Affiliations:** Center for allogeneic stem cell transplantation, Karolinska University Hospital, Huddinge, Sweden; Therapeutic Immunology, Department of Laboratory Medicine, Karolinska Institutet, Stockholm, Sweden; Lung Allergi Kliniken, Karolinska University Hospital, Stockholm, Sweden; Department of Medicine, Karolinska Institutet, Stockholm, Sweden; Department of Medicine, University of Perugia, Perugia, Italy; The Swedish National Institute of Public Health, Solna, Sweden

**Keywords:** Whooping cough, Vaccine, Immune response, Peptide microarrays

## Abstract

**Background:**

Pertussis (*whooping cough*) remains a public health problem despite extensive vaccination strategies. Better understanding of the host-pathogen interaction and the detailed *B. pertussis (Bp)* target recognition pattern will help in guided vaccine design. We characterized the specific epitope antigen recognition profiles of serum antibodies (‘the reactome’) induced by *whooping cough* and *B. pertussis (Bp)* vaccines from a case–control study conducted in 1996 in infants enrolled in a *Bp* vaccine trial in Sweden (Gustafsson, NEJM, 1996, 334, 349–355).

**Methods:**

Sera from children with *whooping cough*, vaccinated with Diphtheria Tetanus Pertussis (*DTP*) whole-cell (*wc*), acellular 5 (*DPTa5*), or with the 2 component (*a2*) vaccines and from infants receiving only *DT* (n = 10 for each group) were tested with high-content peptide microarrays containing 17 *Bp* proteins displayed as linear (n = 3175) peptide stretches. Slides were incubated with serum and peptide-IgG complexes detected with Cy5-labeled goat anti-human IgG and analyzed using a GenePix 4000B microarray scanner, followed by statistical analysis, using PAM (Prediction Analysis for Microarrays) and the identification of uniquely recognized peptide epitopes.

**Results:**

367/3,085 (11.9%) peptides were recognized in 10/10 sera from children with *whooping cough*, 239 (7.7%) in *DTPwc*, 259 (8.4%) in *DTPa5*, 105 (3.4%) *DTPa2*, 179 (5.8%) in the *DT* groups. Recognition of strongly recognized peptides was similar between *whooping cough* and *DPTwc*, but statistically different between *whooping cough* vs. *DTPa5* (p < 0.05), *DTPa2* and *DT* (p < 0.001 vs. both) vaccines. 6/3,085 and 2/3,085 peptides were exclusively recognized in (10/10) sera from children with *whooping cough* and *DTPa2* vaccination, respectively. *DTPwc* resembles more closely the *whooping cough* reactome as compared to acellular vaccines.

**Conclusion:**

We could identify a unique recognition signature common for each vaccination group (10/10 children). Peptide microarray technology allows detection of subtle differences in epitope signature responses and may help to guide rational vaccine development by the objective description of a clinically relevant immune response that confers protection against infectious pathogens.

**Electronic supplementary material:**

The online version of this article (doi:10.1186/s12865-015-0090-3) contains supplementary material, which is available to authorized users.

## Background

*Pertussis* (*whooping cough*) caused by *B. pertussis* (*Bp*), remains a major global public health problem [1,2]. Despite a vaccine coverage over 90% in newborns, *pertussis* remains endemic in the Western countries [3]. In the first months of 2010, outbreaks have been described in Ireland [4], Israel [5] and USA [6]. In California a new outbreak in 2014 was particularly severe, with 10.831 reported cases from January 1st to December 31st [7] (the worst toll since 1947).

The efficacy of current vaccination programs is likely hampered by adaptation of the pathogen, overcoming the effect of herd immunity [8]. A comprehensive study covering *Bp* clinical isolates from 1935 to 2004 showed the appearance of a *Bp* strain that carries a mutation in the *pertussis* toxin promoter; the increased expression of this virulence factor directly correlated with the resurgence of *pertussis* in the last decades in the Netherlands [9]. Another study from the same country, covering the period 1965 to 1992, showed the circulation of different serotypes of the pathogen in correlation with the use of whole cell or acellular *pertussis* vaccines in different time-frames [9]. Substantial evidence has been accumulated in the last two years that immunity induced by acellular vaccines is much shorter lived than immunity induced by whole cell vaccines [10].

There is an unmet need i) to depict the immunological recognition matrix to understand the specific epitope recognition pattern induced by natural infection with *Bp*, ii) to identify differences in immune recognition induced by available *Bp* vaccines as compared to natural infection, and iii) to objectively define the qualitative differences in humoral target recognition induced by current vaccines [11]. We assessed in the current study the immune recognition pattern in serum from infants with *whooping cough* and in 3 groups of infants randomized to different *Bp* vaccines from a trial conducted 1996 in Sweden [12] using a high-content peptide microarray. The immune recognition profile (or ‘reactome’) represents a detailed molecular recognition ‘fingerprint’ of serum IgG directed against linear epitopes.

## Material and Methods

### Patient samples

Samples were randomly selected among the serum samples from the *pertussis* vaccine Stockholm trial I [12], stored at the bio-bank of the Swedish National Institute of Public Health. Samples from children born during 1992, collected at 14 study sites after the completion of the vaccination (doses at 2, 4, and 6 months of age), were included in the study according to the following scheme as described in detail [12].

1. 10 children who received a diphtheria (D) and tetanus (T), vaccine (DT, produced by Swedish National Bacteriological Laboratory, Stockholm, Sweden) as placebo, and developed *whooping cough*;;
2. 10 children immunized with the diphtheria (D), tetanus (T), pertussis (P) *whole cell* (wc) (*DTPwc*) vaccine licensed in the United States (Connaught Laboratories, Swiftwater, PA, USA);
3. 10 children immunized with the 5 component acellular candidate *DTPa5* vaccine (Connaught Laboratories, Toronto, Canada);
4. 10 ichildren immunized with the 2 component acellular candidate *DTPa2* vaccine (SmithKline Beecham, Rixensart, Belgium);
5. 10 children immunized with the Swedish-produced *DT* vaccine and did not develop whooping cough.

Sera were collected 30 days after the last dose, except for the group which whooping cough (group 1, convalescence sera).

#### Ethics statement

The Stockholm regional ethics committee North (Dnr 911258) has approved the study. All subjects provided informed consent. Both parents of the children provided informed consent on their behalf. The informed consent was provided in a written format, signed and is on file at the Swedish National Institute of Public Health, Stockholm, Sweden.

#### Microarray slides and experiments

Peptide microarray slides were customized and manufactured by JPT (Berlin, Germany). The slides contain three identical sub-arrays with 3,175 unique peptides on each subarray. Each sub-array contains 16 blocks arranged in a regular pattern, with spots arranged in a matrix 16 X 15. An image of a microarray is provided in the Additional file 1: Figure S1 (Schematic microarray template) and a table with the list of the peptides, Additional file 2: Table S1 (S1a, non-variant peptides, S1b, variant peptides) are available in the online data supplement. Each sub-array contains positive controls, negative controls and the unique peptides spanning 17 *Bp* proteins (Table 1), a total of 11,520 spots per slide. The entire amino acid sequence of each *Bp* protein was printed on the microarray, as 15-mer amino acid peptides overlapping the previous and next printed peptides by 11 amino acids; this allows to identify minimal amino acid epitopes of 4 amino acids per spot defined by antibody reactivity. Variant peptides for the 17 *Bp* proteins, published earlier [13], were also printed on the microarray (and are listed in Additional file 2: Table S1, S1a, non-variant peptides, S1b, variant peptides, in the supplementary online material).

**Table 1:** **B. pertussis proteins spotted on the peptide microarray slides**

	**Protein ID**	**Accession nu**	**Function**	**Vaccine component**
1	Pertussis toxin subunit 1 precursor, (ptxA)	O69258	Toxin subunit	***All vaccines***
2	Pertussis toxin subunit 2 precursor, (ptxB)	P04978	Toxin subunit	
3	Pertussis toxin subunit 3 precursor, (ptxC)	P04979	Toxin subunit	
4	Pertussis toxin subunit 4 precursor, (ptxD)	P0A3R5	Toxin subunit	
5	Pertussis toxin subunit 5 precursor, (ptxE)	P04981	Toxin subunit	
6	P.69 protein (pertactin/PRN)	CAA09473	Adhesin	***DTPa5 & DTPwc***
7	Filamentous hemagglutinin (FHA)	AAA22974	Adhesin	***DTPA2,5 & DTPwc***
8	Fim2 pilic subunit (Fim2)	Q8VVA0	Adhesin	***DTPa5***
9	Fim3 pilic subunit (Fim3) precursor	CAA35920/P17835	Adhesin	***DTPa5& DTPwc***
10	Tracheal colonization factor (TCF)	CAA08832/O86135	Adhesin	***DTPwc***
11	Bifunctional hemolysin-adenylate cyclase precursor (ATC/cyaA)	P15318	toxin	***DTPwc***
12	Outer membrane porin protein precursor (OMP)	CAA41398, 1/Q04064	Outer membrane porin protein	***DTPwc***
13	Outer membrane porin protein (OmpQ)	CAD12825, Q8VV98	Outer membrane porin protein	***DTPwc***
14	GTP-binding elongation factor (BipA)	Q7VYR0	Regulatory protein	***DTPwc***
15	Bordetella resistance to killing (BrkA)	AAA51646	Putative adhesion	***DTPwc***
16	Vag8 protein (autotransporter) (Vag8)	CAD12828/Q8VV95	Autotransporter	***DTPwc***
17	Putative autotransporter (BapC)	AAC31207	Autotransporter	***DTPwc***

*B. pertussis* proteins spotted on the peptide microarray slides used in the study. The list includes all the acellular vaccines components and other Bp virulence factors. DTPwc: Diphtheria Tetanus Pertussis whole cell vaccine; DTPa5: Diphtheria Tetanus Pertussis 5 component vaccine; DTPa2: Diphtheria Tetanus Pertussis 2 component vaccine.

Experiments were performed following a standardized protocol [14-16]: 300 μL serum diluted 1/100 in washing solution (filtered PBS, 3% fetal calf serum, FCS, Lot nr 45K3397, Sigma, Munich Germany and 0.5% Tween) were pipetted on the peptide microarray slide, covered with a cover slip (Gene-Frame, Abgene, UK) to evenly distribute the dilution over the slide and incubated at +4°C in a humid chamber for 16 hours; after the removal of the cover slip, the slides were washed 5 times on a shaker for 5 min each (twice with washing solution, twice with sterile water and one wash with filtered Milli Q water at the end).After washing, 300 μL Cy5-labeled goat anti-human IgG, affinity purified secondary antibody (Abcam, UK) diluted 1/500 in the washing solution were added (in the dark), and incubated in the dark 1 hour in a humid chamber at room temperature. Washing steps were repeated after the incubation with the secondary antibody. Prior to scanning, slides were dried with a slid spinner (Euro Tech, UK). Five additional slides were processed using only buffer in the first incubation step, to detect false positive spots due to non-specific binding of the secondary reagent. High-definition images from the slides were acquired with GenePix 4000B microarray scanner (Axon Instruments-Molecular Devices, Union City, US) using the wavelength 635 nm (red channel, for the specific IgG signal quantification) and 532 nm (green channel, positive controls for grid alignment and orientation). Data acquisition from the images was performed with the software Gene Pix 6 Pro (Axon Instruments-Molecular Devices, Union City, US).

#### Data analysis

Data analysis consisted of 4 steps as described [17].

##### Quality control

All images and aligned files were visually inspected to check for artifacts and for spots erroneously flagged by the software. Images of background and foreground intensities were produced for every sub-array by using bioinformatics tools. All spots or areas which did not represent a high quality signal were removed from analysis. Further quality controls were also performed [17] and the intensity values were background-corrected (index = Log2(foreground/background)).

##### False positive, “empty” spots removal and exclusion of low intensity signal spots

All spots showing a response on the buffer slides - and for this reason identified as possible false positive - were removed from the analysis, as well as all spots that did not show any signal (“empty”, with an index value ≤ −50) in the data acquisition process. Low response spots, with a signal below a computed cut off (μ + 2SD, where SD is the standard deviation of μ, the mean value of negative controls in the slides of each study group) were also removed.

##### Normalization

the normalization process was performed using the simple linear model as described before [18,15,14,16,17]. The quality of the normalization was assessed by inspection of the normalized data plot in all the study groups.

##### Analysis and data mining

Data analysis was performed using two different statistical methods: (i) PAM (Prediction Analysis for Microarrays) [19], a predictive analysis which performs sample classification from peptide recognition data providing a list of significant peptides whose response level characterizes each diagnostic group. Compared to other differential recognition analysis methods, PAM is highly selective and allows the detailed examination of each time point in case of consecutive serum testing. This reveals only the peptide target with good predictive power associated with the differentiation of the patient group(s). This will result in a set of peptides constantly weakly recognized in one group and strongly in the other group. (ii) ‘Exclusive recognition analysis’ (ERA) of epitopes predicted by PAM. The latter approach identifies epitopes recognized in serum from individuals exclusively in one group but *never in serum from any individual* in a control reference group, e.g. in the current report the ‘reference’ individuals who received placebo (termed ‘exclusively recognized epitopes’, ERA). Strongly recognized peptides identified in each group were plotted according to index value and number of times they were recognized in the group of interest. Lastly, a 3D-graphical representation of the “reactome” [20] of *B. pertussis* proteins was computed for every group, by plotting mean index value for every peptide, as well as the protein and position on the respective amino acid-sequence of the protein. A similar 3D-plot was computed to compare the signals in two study groups, plotting the Δ value between the mean index values in the two groups (e.g. the Δ value for each individual reactivity, peptide by peptide in ‘test group’ as compared to the ‘reference’ group. The entire set of differences can be compiled in a 3D graphical representation).

All pre-processing and statistical analyses were performed customizing open-source packages of Bioconductor, R software [21,22]. In addition, to assess the statistical significance of the differences in the trends of recognition (defined as the sequence of the observations in 100%, 90% and 80% in serum from children in each group) with whooping cough group vs. all remaining groups, as well as the *DT (control)* group vs. the remaining groups, a Chi-square test for the goodness of fit was used.

#### Epitope comparison with published data

In order to relate our results to the epitopes which have been identified previously in the literature, we searched the B-cell Immune Epitope Database [23] (IEDB) site (http://www.immuneepitope.org/) and homologous sequences highlighted.

## Results

### Differential recognition of Bp epitopes in children with *whooping cough*

**Figure 1:**
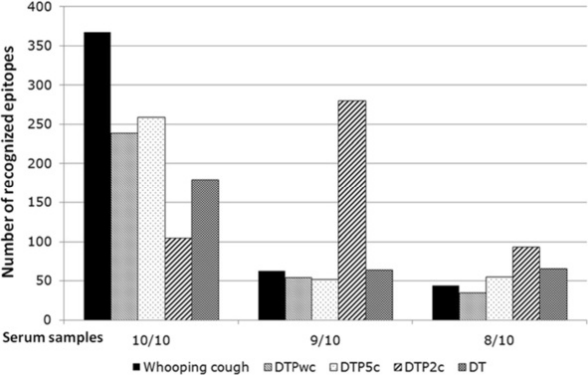
Number of peptides recognized in each study group in all (10 out of 10), 90% (9 out of 10) and 80% (8 out of 10) serum samples obtained from children s in each test group. The trend of recognition (defined by the observations in the three points in each group) was similar between natural infection and DPTwc (p = NS), while it was statistically different in the comparison between natural infection and the remaining groups (p < 0.05 vs. DTPa5, p < 0.001 vs. both DTPa2 and DT).

Three thousand eighty five peptides remained in the analysis after quality control, i.e. after false positive, empty and low intensity signal spots removal. Analysis of sera from children with *whooping cough* showed that 367 (11.9%) of 3,085 peptides were commonly recognized in in 10/10 serum samples. 239 (7.7%) peptides were recognized in 10/10 serum samples from subjects who received the *DTPwc* vaccine, while 259 (8.4%), 105 (3.4%) and 179 (5.8%) peptides were recognized in sera obtained from 10/10 children receiving the *DTPa5* vaccine, the *DTPa2* vaccine or the *DT* vaccine, respectively (without a diagnosis of *whooping cough*). Figure 1 shows the number of peptides recognized in serum from 10/10, 9/10 and 8/10 children in each group [see for details Figure S2 (distribution of peptide recognition for each group) in the online material]. The trend of peptide epitope recognition was similar between natural *Bp* infection and *DPTwc* (p = NS), while it was statistically different between serum recognition patterns associated with *whooping cough* and the groups who received the acellular vaccines (p < 0.05 vs. *DTPa5*, p < 0.001 vs. both *DTPa2* and *DT*). Table 2 shows the frequency of recognition per peptide species, their origin on the respective *Bp* protein, stratified for the study groups.

**Table 2:** **Recognition frequency of target peptides spotted on the microarray slides, stratified per study group and by target proteins**

**B. pertussis protein**	**GENE**	**Whooping cough**	**DTP wc**	**DTPa5**	**DPTa2**	**DT**
Pertussis toxin subunit 1 precursor	PtxS1	4				1
Pertussis toxin subunit 2 precursor	PtxS2	3		1		1
Pertussis toxin subunit 3 precursor	PtxS3	5	2	2		2
Pertussis toxin subunit 4 precursor	PtxS4	2	1	2		1
Pertussis toxin subunit 5 precursor	PtxS5	3	4	2		2
P.69A protein (pertactin)	Prn	11	3	6	2	4
Filamentous hemagglutinin	fhaB	27	10	11	5	14
Fim2 pilic subunit	fim2	1	1			
Serotype 3 fimbrial subunit precursor	fim3	2				
Tracheal colonization factor	tcfA	19	15	10	7	9
Bifunctional hemolysin-adenylate cyclaseprecursor	cyaA	61	41	50	22	39
Outer membrane porin protein precursor	ompP	27	16	16	4	12
Outer membrane porin protein OmpQ	ompQ	37	32	36	12	24
GTP-binding elongation factor	bipA	26	16	13	9	8
Bordetella resistance to killing	brkA	10	7	8	9	6
Vag8 protein (Autotransporter)	vag8	44	28	28	8	18
Putative autotransporter	bapC	69	47	54	21	25

The table shows the recognition frequency of target peptides spotted on the microarray slides, stratified per study group and by target proteins. Whooping cough: children who received the placebo and developed whooping cough; DTPwc: Diphtheria Tetanus Pertussis whole cell vaccine; DTPa5: Diphtheria Tetanus Pertussis 5 component vaccine; DTPa2: Diphtheria Tetanus Pertussis 2 component vaccine; DT: children who received only the Diphtheria Tetanus vaccine; all groups N = 10; none of the children in the vaccine groups, including DT, were diagnosed with a condition related to B. pertussis infection.

**Figure 2:**
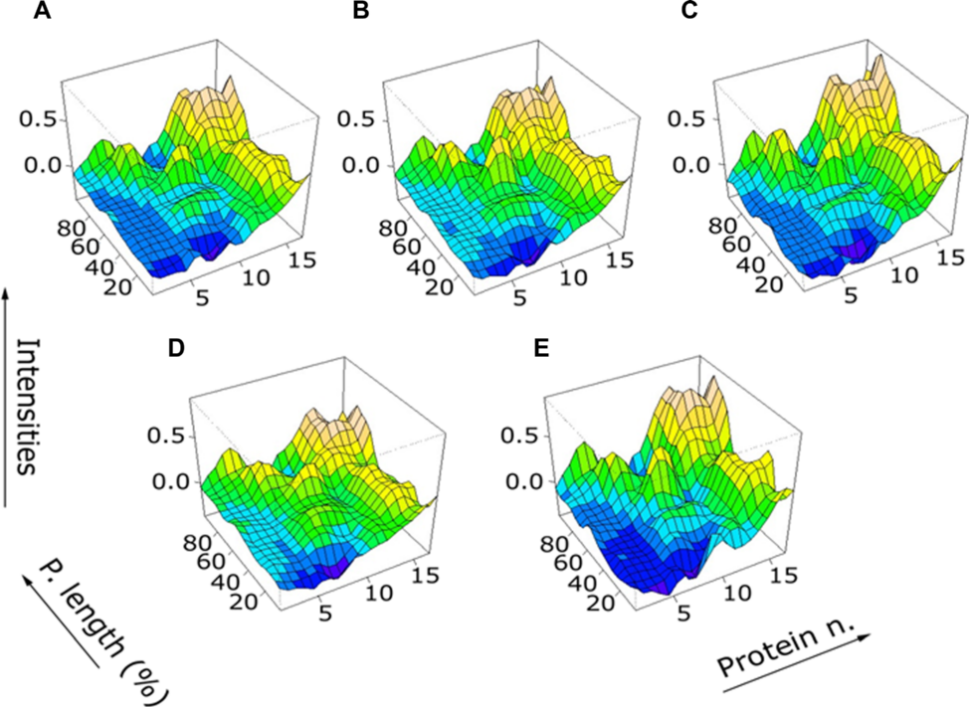
3D-plots representing the mean index value reactivity in each individual study group. **A)** DTPwc: children who received the Diphtheria Tetanus Pertussis whole cell vaccine; **B)** DTPa5: Diphtheria Tetanus Pertussis 5 components vaccine; **C)** DTPa2: i Diphtheria Tetanus Pertussis 2 components vaccine; **D)** Whooping cough: children who received the placebo and developed natural infection; **E)** DT: children vaccinated the placebo, who did not develop whooping cough. The proteins on the microarray are aligned the : 1 - P.69A protein (pertactin); 2 - Bordetella resistance to killing; 3 - Tracheal colonization factor; 4 - Vag8 protein (Autotransporter); 5 - Pertussis toxin subunit 1 precursor; 6 - Pertussis toxin subunit 2 precursor; 7 - Pertussis toxin subunit 3 precursor; 8 - Pertussis toxin subunit 4 precursor; 9 - Pertussis toxin subunit 5 precursor; 10 - Bifunctional hemolysin-adenylate cyclaseprecursor; 11 - Filamentous hemagglutinin; 12 - Fim2 pilic subunit; 13 - Serotype 3 fimbrial subunit precursor; 14 - Outer membrane porin protein precursor; 15 - Outer membrane porin protein OmpQ; 16 - GTP-binding elongation factor; 17 - putative autotransporter. The DTPwc and the DTPa5 vaccines were similar to the pattern obtained from serum from children diagnosed wth whooping cough, while the DTPa2 group showed a shape similar to the IgG recognition pattern detected in the DT group.

Figure 2 shows the 3D representation of the “reactome” in each group, this ‘landscape recognition pattern’ depicts the qualitative appreciation of differences in antibody recognition patterns between different (vaccine) groups as a function of the IgG signal strength to individual *Bp* epitopes. The IgG target recognition pattern induced by the *DTPwc* and the *DTPa5* vaccines were similar to the serum pattern from children with *whooping cough*, while the *DTPa2* group showed a shape similar to the recognition pattern detected in the *DT* group [see also Additional file 3 Video S1 (differences in B. pertussis recognition patterns) in the online data supplement].

### Different IgG reactome and exclusive recognition of *Bp* epitopes in sera from infected children

**Figure. 3:**
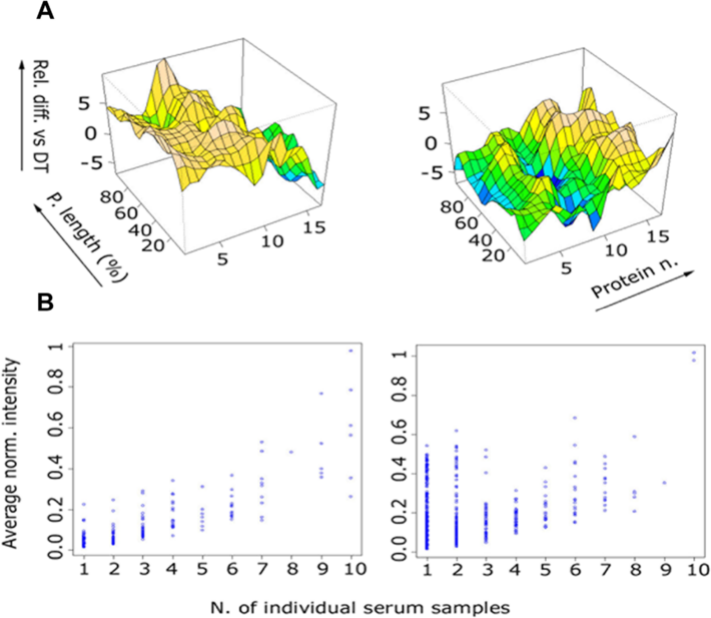
Differential target epitope recognition in patients with whooping cough. Figure 3**A**: 3D-plots representing the differential mean index value in the whooping cough (left panel) and DTPa2 (right panel) groups as compared to the reference the mean index value in the DT group. Figure 3**B**: Inclusive/exclusive analysis of serum reactivity from children in the whooping cough (left panel) and DTPa2 (right panel) groups vs. the DT (control) group. These peptides are exclusively recognized in the respective test group at the indicated frequency (e.g. 10/10 serum samples) and never in serum from any individual in the DT group. 12 peptides were strongly recognized in serum from at least 8 out 10 of the children developing whooping cough; 8 peptides were highly recognized in serum from at least 8 out of 10 infants in the DTPa2 group. No specific pattern of recognition was detected by the inclusive/exclusive analysis in the other study groups. DTPa2: infants who received the Diphtheria Tetanus Pertussis 2 components vaccine, DT: infants not vaccinated and who did tno developed whooping cough.

Next, we visualized differences between the ‘reactome patterns’. The *Bp* serum recognition pattern from individuals with *whooping cough* and from the *DTPa2* groups showed a characteristic shape of IgG recognition curves using the computed Δ value for mean indexes using the *DT* group as a reference (Figure 3A and the Figure S3, 3D-plots recognition of the differential mean index value in each study group, in the online supplementary material). The 3D-plots visualize the differential mean index value in the whooping cough (Figure 3A, left panel) and DTPa2 (Figure 3A, right panel) groups as compared to the reference, i.e. the mean index value in the DT (reference) group. The 3D plots help to visualize the overall recognition pattern, based on serum IgG binding to individual epitopes, yet it does not identify peptides that are exclusively recognized in a (test) group. We show in Figure 3B the exclusive recognition analysis (ERA) of sera from children with whooping cough (Figure 3B, left panel) and DTPa2 (Figure 3B, right panel) groups vs. the DT group. These peptides are exclusively recognized in the respective test group at the indicated frequency (e.g. 10/10 serum samples) and never in serum from any individual in the DT group (n = 10 individuals). 12 peptides were strongly recognized in serum from at least 8 out 10 of the children developing whooping cough and never in any serum samples from an children in the DT (control group, see also Figure S4, i.e. the exclusive recognition analysis (ERA) for peptides exclusively detected in each of the study groups vs. serum from non vaccinated children, in the supplementary material).

**Figure 4:**
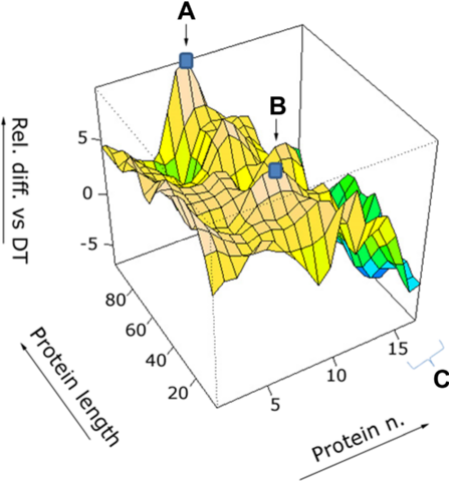
3D-plots representing the differential mean index IgG recognition value in serum from whooping cough group. The mean index IgG value in the DT group was used as reference. This graph highlights proteins with high (**A** and **B**: filamentous hemagglutinin, FHA) and low (**C**: Bordetella resistance to killing, BrkA, Vag8 protein, Putative autotransporter, BapC) mean index values. This ’ landscape analysis’ aids to visualize the global recognition pattern of peptides recognized in serum samples from each group, it also allows to appreciate clear differences in the serum recognition pattern to Bp components in each test group. Note the strong recognition of Bp components to filamentous hemagglutinin, yet the relative decrease of antibody reactivities directed against the Vag8 protein and a factor which confers resistance to killing.

6 strongly and exclusively recognized linear *Bp* epitopes (from *Bp* virulence proteins) could be identified in 10/10 serum samples from patients with whooping cough; 2 peptides were derived from *pertussis* toxin components (1 from PtxS4, and 1 from cyaA); 3 peptides from adhesion proteins (2 from prn, and 1 from FHA); and 1 peptide from the transporter protein ompP. In serum samples from the *DTPa2* group, 1 peptide was derived from the tracheal colonization factor (tcfA) and a different peptide epitope from the GTP-binding elongation factor (bipA). The two peptides specifically recognized in the *DTPa2* group did not belong to the 2 proteins used as vaccine components; the sequence of these 8 peptides is reported in Table 3. We performed then a comparative 3D analysis of *Bp* epitope recognition in serum from children with *whooping cough* versus the control, i.e. the reference group who received DT (Figure 4), to visualize which *Bp* proteins are predominantly recognized in serum from *Bp*- infected children. Strong IgG epitope target recognition could be observed to components of filamentous hemagglutinin, yet comparatively relatively weak IgG reactivity directed against the Vag8 protein and a factor which confers resistance to killing.

**Table 3:** **Sequence of target epitopes exclusively recognized in serum from individuals either with the natural B. pertussis infection or after DTPa2 vaccination**

***B. pertussis*** **protein**	**GENE**	**B-cell epitope**
***Children with whooping cough***		
Pertussis toxin subunit 4 precursor	PtxS4	CFGKDLKRPGSSPME
P.69A protein (pertactin)	Prn	LWYAESNALSKRLGE
P.69A protein (pertactin)	Prn	AVVHLQLATIRRGDA
Filamentous hemagglutinin	fhaB	FAADLRTVYAKQADQ
Bifunctional hemolysin-adenylate cyclaseprecursor	cyaA	HAANQAVDQAGIEKL
Outer membrane porin protein precursor	ompP	FGVNTFADGFKANSY
***Children who received the the DTPa2 vaccine***		
Bifunctional hemolysin-adenylate cyclaseprecursor	cyaA	DQTVSGLEIGLDRGV
Tracheal colonization factor	tcfA	ASNGLRIKDDGTNSM

The Table shows the sequence of target epitopes specifically recognized in serum (10/10) children with whooping cough (n = 6 peptides) and in serum from children who receivedthe Diphtheria, Tetanus, Pertussis acellular 2 components (DTPa2) vaccine (2 peptides). Peptide microarray slides containing unique Bp peptides (n = 3,085 peptides) were processed and analyzed. We list here only peptides which were exclusively recognized in serum samples from individuals either with the natural *Bp* infection or after DTPa2 vaccination and never recognized in any serum samples from individuals who received the placebo (i.e. DT ).

### Differential recognition of *Bp* epitopes identified by PAM segregates *Bp* vaccines

The exclusive epitope recognition analysis yields peptide targets that are unique for each test cohort. A different kind of analysis, PAM, identifies epitopes that are both always strongly recognized in the reference, and always weakly recognized in the ‘test’ group (or vice versa) in serum from each individual in the group. This allows predicting whether a reactivity pattern is associated to a certain defined endpoint (e.g. infection or vaccination, vaccination *versus* placebo). Alternatively, this method allows also comparing groups of individuals, i.e. individuals who received different kinds of *Bp* vaccines.

**Figure 5:**
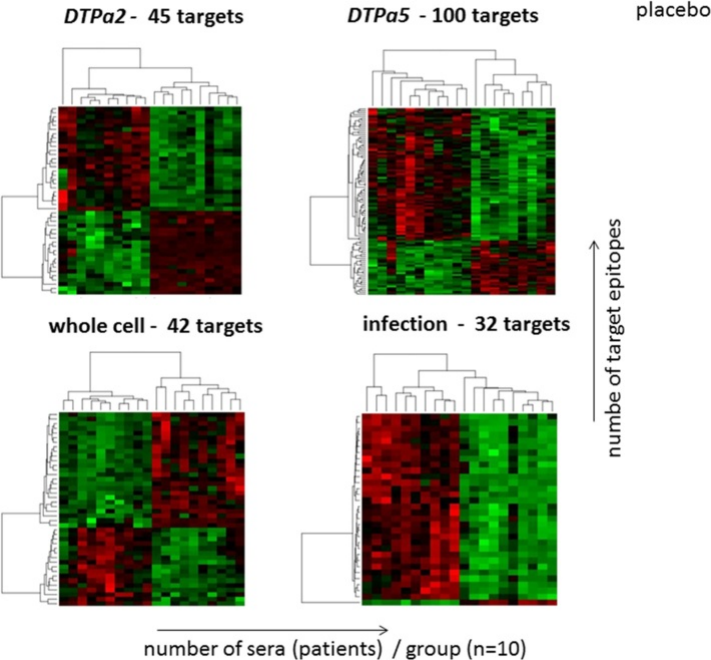
PAM analysis of strongly versus weakly recognized *Bp* target epitopes segregates vaccination with different vaccines compared to healthy controls (who received the DT adjuvant and tested sero-negative for a previous *Bp* infection). Epitopes are recognized in serum from 10/10 individuals in each group, the number of targets that segregates each group is listed. The colors are based on the fluorescence intensity of the peptide microarray results. Red: strong recognition and green: weak recognition. The identity of each target peptide is listed in the Additional file 2: Table S2.

**Figure 6:**
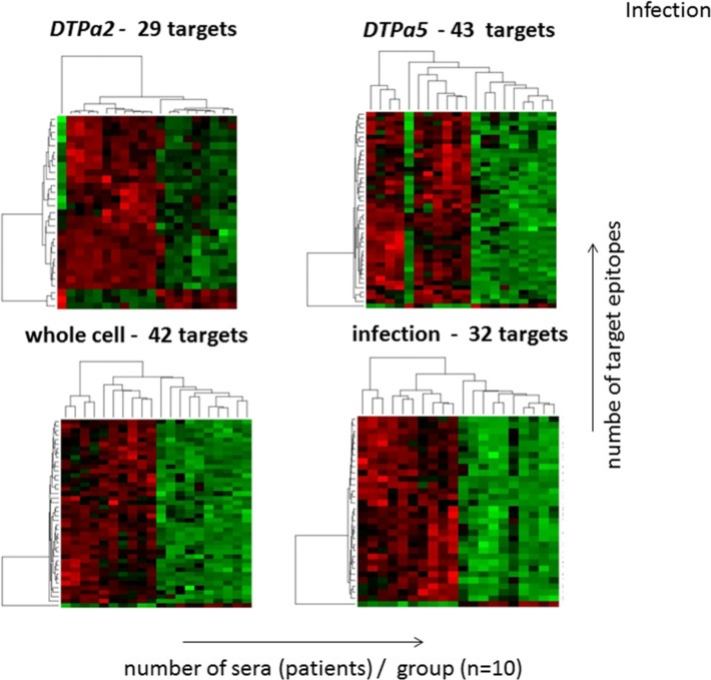
PAM analysis of strongly versus weakly recognized *Bp* target epitopes segregates vaccination with different vaccines compared to individuals with Pertussis. Epitopes are recognized in 10/10 serum samples in each group, the number of targets that segregates each group is listed. The colors are based on the fluorescence intensity of the peptide microarray results. Red: strong recognition and green: weak recognition. The identity of each target peptide is listed in the supplementary online material Table S2.

Figure 5 shows the comparison of the vaccinated groups, as well as the *Bp*-infected group along with the group who received (DT) placebo. The peptide epitope targets are only shown if they exhibited a constant reactivity pattern for each of the 10 individuals in the test groups. The DTPa2 group yielded 45, the DTPa5 100 and the whole cell vaccine 42 *Bp* targets that were differentially recognized as compared to the placebo group. Infection with *Bordetella pertussis* yielded 32 targets that were differentially recognized as compared to the group who received (DT) placebo. The identity of these targets and location within each target molecule is provided in the online Additional file 2: Table S2 (Differential epitope recognition analysis). The comparison between the vaccinated groups with the 10 individual who experienced natural infection resulted in 29 targets for the DTPa2, 43 targets for the DTPa5 and 42 targets for the group who received the whole cell vaccine (Figure 6). As expected, the comparison between the placebo and the infected group results also in 32 *Bp* targets that would segregate infected versus non- *Bp* infected individuals. The detailed listing of the targets is provided in the Additional file 2: Table S2 (Differential epitope recognition analysis).

### Peptide microarray analysis identifies previously described B-cell epitopes

Finally, we examined if already published *Bp* epitopes were captured by the peptide microarray matrix: sixty-five *B. pertussis* B-cell epitopes were retrieved from the IEDB (listed in the Additional file 2: Table S3, B-cell epitopes identified via online data repositories, in the online supplementary material, see also Figure S5, i.e. Plots of previously reported epitopes and epitopes using the peptide microarray approach, in the online data supplement), showing that the platform utilized in this report picks up already described B-cell epitopes. We identified *Bp* target peptides that were frequently recognized; these target peptides exhibited a variant amino acid sequence which occurs in natural *Bp* clinical isolates – the non-variant peptide epitopes were not recognized (see Additional file 2: Table S3, previously described epitopes, and S4, commonly recognized epitopes in serum from the respective patient groups, in the online data supplement).

## Discussion

Primary prevention remains the main intervention to limit pertussis occurrence and new transmission. The protective effect mediated by the *Bp* vaccine(s) appeared to vanish over time [3] and emergence of *Bp* strains carrying mutations of virulence factors has been reported [9]

Our study explored the immune response against *Bp* induced by natural infection and 3 different vaccines in children enrolled in a clinical trial conducted in Sweden [12]: this trial showed that acellular vaccines, in particular *DTPa5*, ensured the best ratio between protection from *whooping cough* and acceptable rates of side effects.

We have been able to i) identify a high number of B-cell epitopes that have been described in the literature and the *Bp* epitope database with the peptide microarray technology described in this report (supplementary Table S5), ii) show robust differences between different vaccines concerning epitope recognition patterns (see Figures 5 and 6), and iii) picked up differences in IgG mutant Bp epitopes, i.e. that not the wildtype, yet the naturally occurring variant *Bp* epitope was recognized (Supplementary Figure S5). These results suggest that peptide microarrays provide a platform to visualize quantitative and qualitative differences in humoral recognition patterns at the epitope level. This may be relevant since genetic changes in *Bp* have been reported [24-28].

Serum from children with *whooping cough* displayed the broadest *Bp* epitope antibody recognition, with a certain number of peptides exclusively recognized in this group. Only serum analysis from individuals vaccinated with *DTPwc* showed a similar trend concerning the number of recognized *Bp* peptides, consistent with the fact that all the components of the bacterial wall are present in this vaccine preparation. The *DTPa5* as well as the *DTPa2* vaccine induced a significantly different humoral recognition pattern. The *DTPa2* vaccine appeared to boost pre-existing *Bp*-reactive antibody responses as compared to the induction and expansion of new antibody reactivity pattern directed to new *Bp* target antigens.. This is reflected in stronger recognition of the proteins Bordetella resistance to killing (BrkA), Vag8 protein, Putative autotransporter (BapC). Conversely, humoral recognition of the filamentous hemagglutinin (FHA) appears to be induced by vaccination with the *DTPwc vaccine* as well as after natural infection with *Bp*.

The immune responses induced by *Bp* in the course of *whooping cough* after resolution is long-lasting and more protracted as compared to the immune response induced by vaccines; it may offer new potential targets to improve vaccine design for *pertussis* once the nature of the antibody reactivity mediating immune protection will be deciphered. The *DTPa2* vaccine has a very good safety profile, yet its effect has been questioned in the past particularly in terms of protection and its duration [12]. This could, in part, be explained by the fact that the *DTPa2* vaccine acts by boosting a pre-existing ‘natural’ *Bp* recognition matrix, as compared to other vaccines which rather induced a shift in serum *Bp* epitope recognition patterns. Both *DTPwc* and *DTPa5* showed a reactome similar to the *Bp* natural infection; future studies may address whether this would be related to increased protection induced by these vaccines compared to the *DTPa2* [12].

There are at least four different, not mutually exclusive explanations concerning the spectrum of *Bp* target recognition induced by different vaccines: i) Vaccines could boost and modulate the recognition matrix for natural occurring and *Bp* specific antibodies directed against *Bordetella spp,* eliciting pre-existing humoral immune responses directed against *Bp* epitopes. This is consistent with the concept that ‘natural antibodies’ are a fundamental part of the immune system and play a crucial role in modulating the recognition (and response) to self and ‘non-self’ infectious antigens [29,30] ii) Children without *Bp* vaccination experience most likely the full-blown disease with the typical *whooping cough* presentation, yet some (non-vaccinated) individuals appear to experience limited disease, suggesting that ‘abortive’ cases or even immune protection may occur in the absence of vaccination [31,32]. Therefore, silent infection or colonization with other *Bordetella species* (which may also express certain virulence factors, e.g. *B. parapertussis* and *B. bronchiseptica*,, as well as *B. trematum* and *B. holmesii*) [33,34] could be responsible in the modulation of the immunological recognition matrix directed against *Bp*.

Most of the serum immune responses in the groups of our study were shared among the individuals in each group (with up to 11.9% of *Bp* peptides recognized in sera from 10/10 infants with *whooping cough*), yet we identified also ‘private’ humoral responses unique for each individuals (see Figure S2 in the online data supplement); iii) Potentially cross-reactive antibodies which target closely related hemagglutinin or fimbriae from other bacterial species may be responsible for different efficacy of the vaccines iv) Vaccination leads not only to epitope-specific immune responses directed against targets contained in the vaccine, yet to other molecular targets as well (epitope spreading). This concept is appreciated in other areas of medicine and contributes to clinical efficacy of some vaccines. For instance, antigen-spreading mediates vaccination-induced regression in human melanoma [35] and the impact of different (vaccine) adjutants on the antibody repertoire to target protective epitopes is appreciated in the development of humoral and cellular immune responses against influenza A [36,37].

If the human proteome is scanned as 5mer peptides, then up to 90% of the viral proteome may show similarity to the human proteome [38]. This information is more easily accessible using peptide microarrays, since IgG recognition patterns are mapped using defined peptide targets that can be tested for amino acid composition similarities with related or unrelated protein targets.

We show here that not only the antibody titers directed against specific *Bp* targets, yet also the detailed recognition focus of vaccine components was different, even if the vaccines contained the same molecular components, supporting the notion that *Bp* vaccine composition impacts on the quality of antibody response [25,39-41].

Peptide-microarray-guided analysis may also help to decipher the phenomenon of ‘epitope suppression’ [42] which has recently gained interest in *Bp* vaccine evaluation. Individuals primed with a *Bp* (one dose) whole cell vaccine exhibited decreased pertussis attack rates as compared to individuals primed with acellular vaccines [43,44]: differential target epitope focus associated with different vaccine formulation was evident in the PAM-analysis reported in the current study (see Figures 5 and 6, the detailed target epitope focus analysis is provided in the Additional file 2: Table S2).

## Conclusion

Microarray analysis offered for the first time a comprehensive characterization of the immune response to *Bp* after natural infection in comparison to 3 vaccines. The report shows the potential of the high-content peptide microarray technique in infectious diseases, detecting epitopes by far more numerous and likely more immunogenic compared to the ones already reported in electronic databases. It also offers a new possibility to objectively decipher immune reactivity in clinically well-defined test groups undergoing vaccination strategies and allows to test for batch-to-batch consistency. Target recognition patterns in serum from individuals who experienced infection with *Bp* (and enjoyed protection for a longer period of time) could guide the development of *Bp* vaccines.

## Additional files

Additional file 1:
**Figure S1-S5.**
Additional file 2:
**Table S1-S4.**
Additional file 3:
**Video S1.**
