# Supplementary material for: Serum reactome induced by Bordetella pertussis infection and Pertussis vaccines: qualitative differences in serum antibody recognition patterns revealed by peptide microarray analysis
Source: BMC Immunol. 2015 Jul 1;16:40. doi: 10.1186/s12865-015-0090-3 (PMC4487959; doi:10.1186/s12865-015-0090-3)
Supplement: Additional file 1: — Figure S1-S5. [file 12865_2015_90_MOESM1_ESM.pdf]

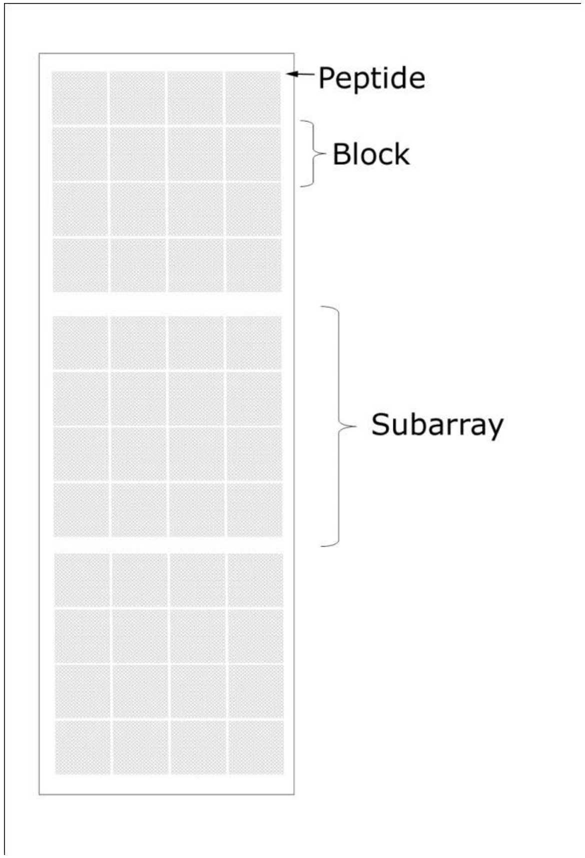

Fig S1. Schematic illustration of a *B. pertussis* microarray: each microarray contains 11,520 spots, organized in 3 subarrays, each subarray organized in 16 blocks. 3,175 unique peptides, positive and negative controls are printed in triplicates (one of each corresponding spot) in every subarray.

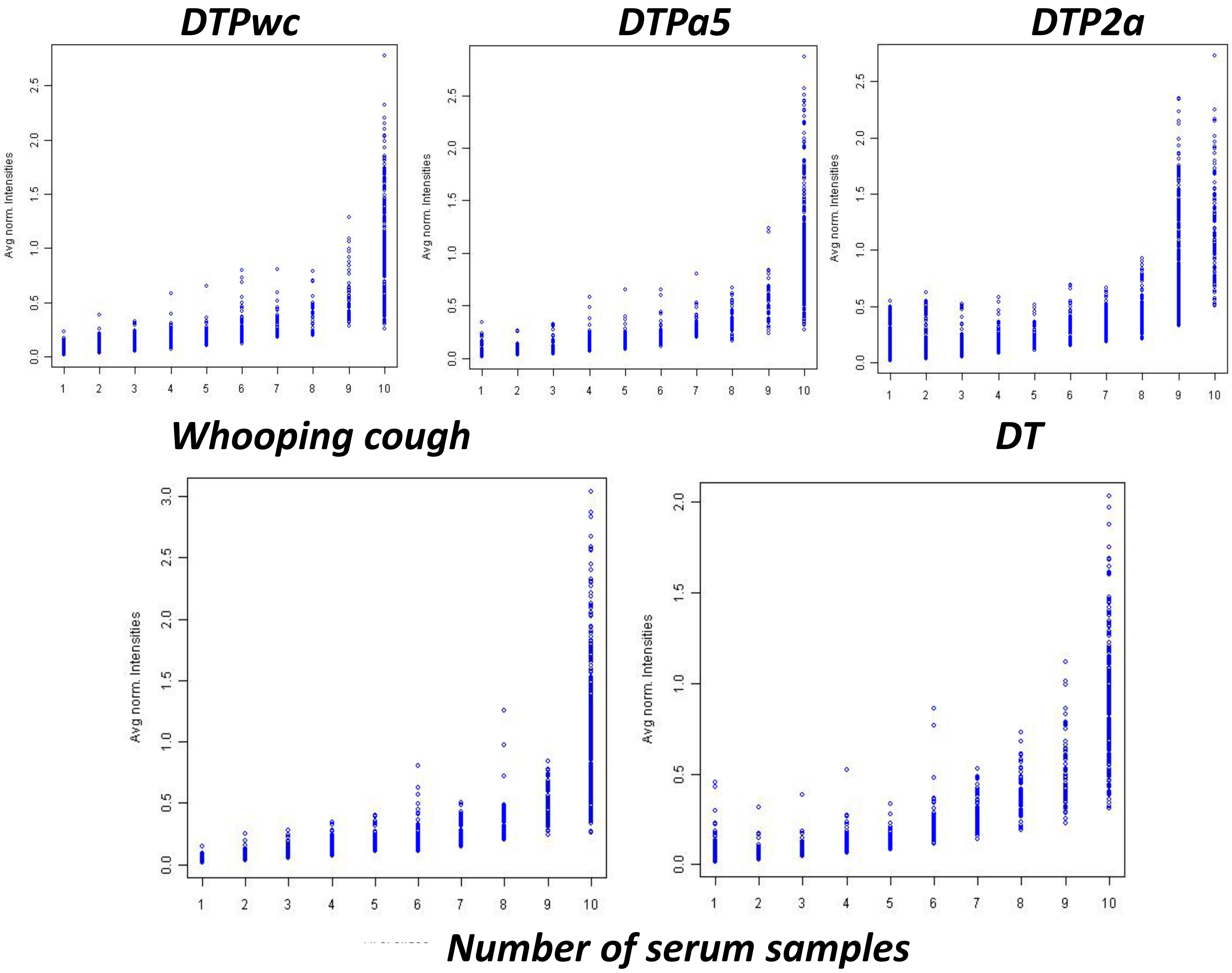

Fig. S2. The plots show the distribution of recognized peptides per average intensity of the signal (Y axis) and number of serum samples in which they were detected (X axis) in each study group. Most of the humural immune responses in study groups were shared among the individuals in each group (with up to 11.9% of Bp peptides recognized in sera from 10/10 infants with whooping cough), yet there are also some ‘private’ humoral responses unique for each individuals, as revealed by peptides recognized only in a few individuals.

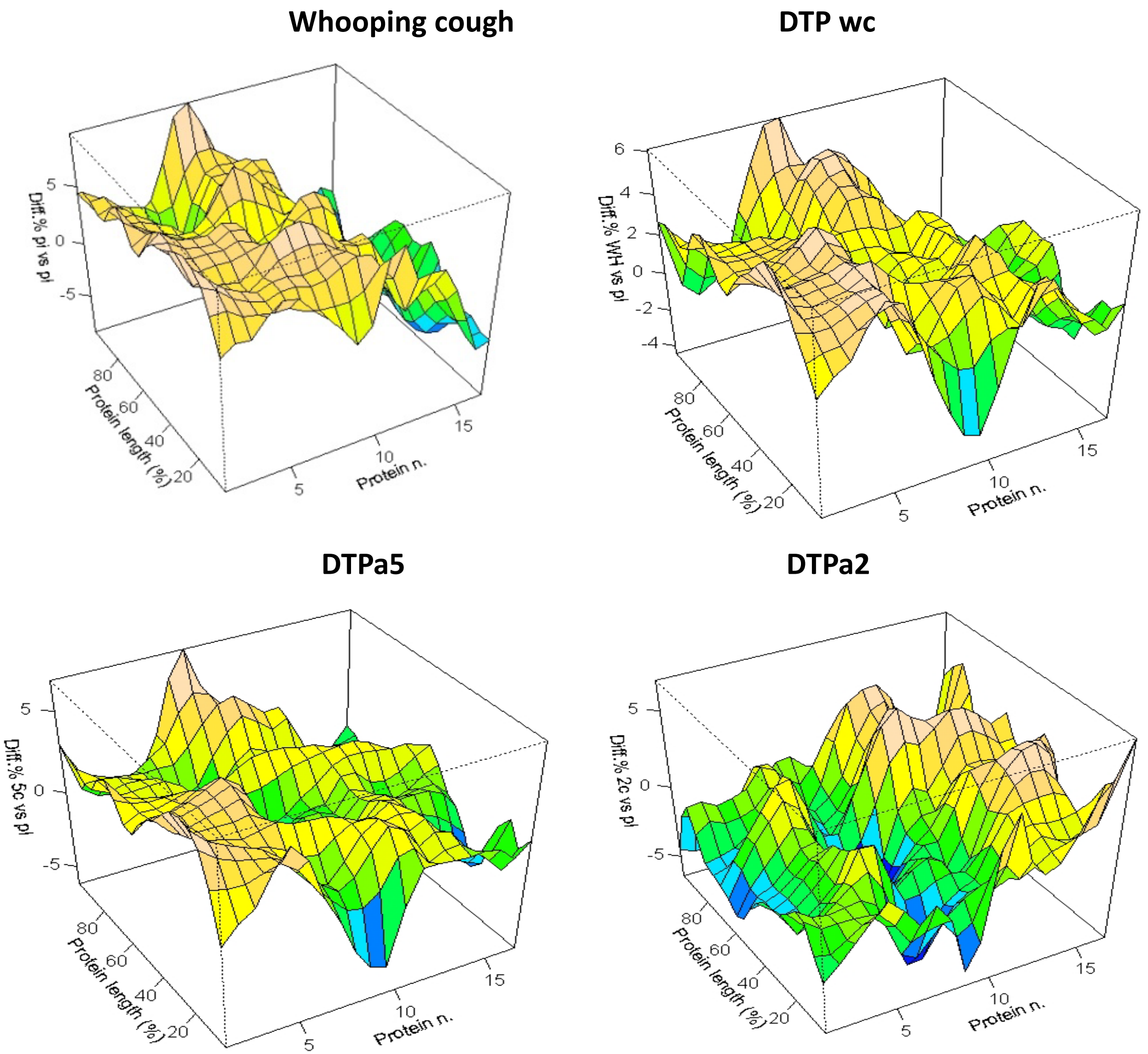

Fig S3. 3D-plots represent the differential mean index value in each study group; the values from serum of the control group (infants not vaccinated and not developing whooping cough) served as the reference. DTPwc: infants who received the Diphtheria Tetanus Pertussis whole cell vaccine; DTPa5: infants who received the Diphtheria Tetanus Pertussis 5 components vaccine; DTPa2: infants who received the Diphtheria Tetanus Pertussis 2 components vaccine. The “reactosome” from individuals with whooping cough and from the DTPa2 groups showed a characteristic shape of their curve. Curves from DTPwc and DTPa5 resembled the curve detected in whooping cough group.

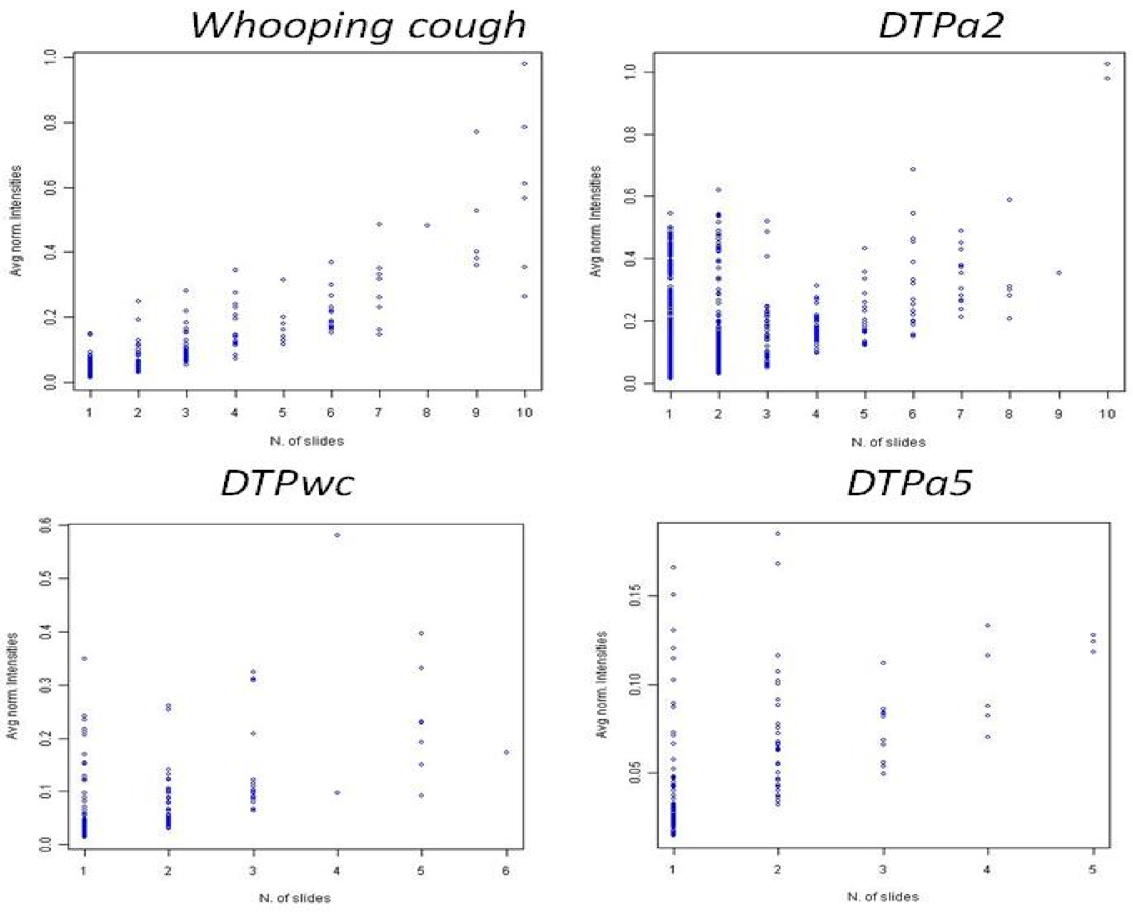

Fig S4. The plots show the exclusive recognition analysis (ERA) for peptides exclusively detected in each of the study groups vs. serum from non vaccinated children (DT vaccinated) stratified per average intensity of the signal (Y axis) and number of serum samples in which they were detected (X axis). Peptides were exclusively recognized in serum samples from the whooping cough and DTPa2 groups; this was not found to be true for serum samples from the other vaccine groups. In the serum samples from patients with whooping cough, the 6 strongly and exclusively recognized linear Bp epitopes in 10/10 subjects derived from different Bp virulence proteins categories: 2 peptides are from pertussis toxin components (1 from PtxS4, and 1 from cyaA); 3 peptides from adhesion proteins (2 from prn, and 1 from FHA); and 1 peptide from the transporter protein ompP. In serum samples from the DTPa2 group, 1 peptide derived from the tracheal colonization factor (tcfA) and a different peptide epitope from the GTP-binding elongation factor (bipA).

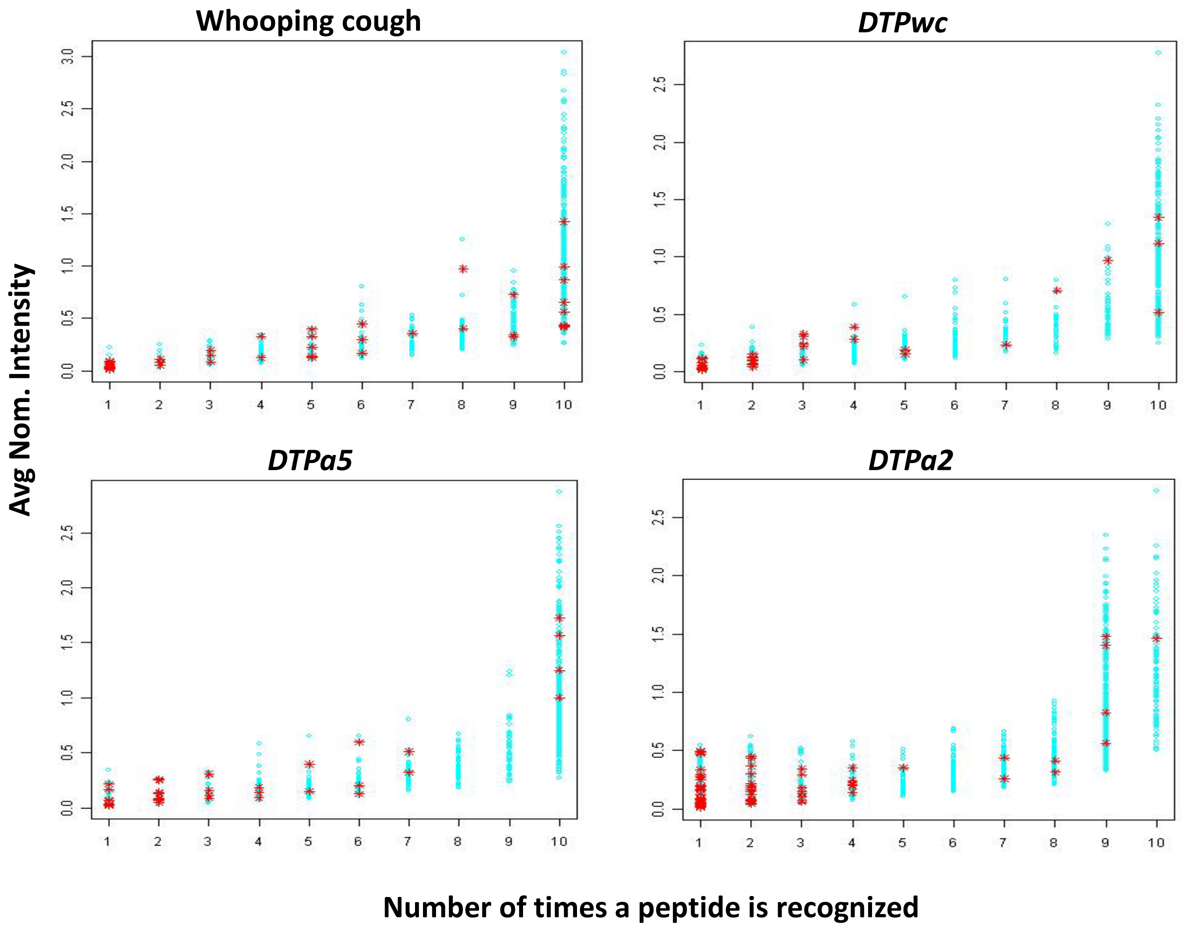

Fig S5. Plot of peptides recognized per average intensity of the signal (Y axis) and number of serum samples in which they were detected (X axis) in serum samples from infants with whooping cough or in infants after immunization with Diphtheria Tetanus Pertussis whole cell vaccine (DTPwc); Diphtheria Tetanus Pertussis 5 components vaccine (DTPa5); Diphtheria Tetanus Pertussis 2 components vaccine (DTPa2). Red stars show peptide containing in their sequence epitopes retrieved from the B-cell database in Immune Epitope Database (http://www.immuneepitope.org/): the peptide microarry platform picks up peptide species that were described to be recognized in the literature. Note that some of these epitopes represent commonly recognized targets (positive in 10/10 samples), others are non frequently recognized 'private epitopes'.
