# Supplementary material for: Serum reactome induced by Bordetella pertussis infection and Pertussis vaccines: qualitative differences in serum antibody recognition patterns revealed by peptide microarray analysis
Source: BMC Immunol. 2015 Jul 1;16:40. doi: 10.1186/s12865-015-0090-3 (PMC4487959; doi:10.1186/s12865-015-0090-3)
Supplement: Additional file 2: — Table S1-S4. [file 12865_2015_90_MOESM2_ESM.pdf]

Reactosomes induced by Bordatella pertussis infection and Pertussis vaccines: serum antibody recognition patterns revealed by peptide microarrays.

Davide Valentini, Giovanni Ferrara, Reza Advani, Hans O. Hallander, Markus Maeurer

ONLINE DATA SUPPLEMENT

Tables S1-S4

Table S1a. List of Bp peptides printed on the microarray corresponding to the Bp target protein.

	Peptide	Protein and accession number
1	TPGIVIPPQEQITQH	Pertussis toxin subunit 2 prec- P04978
2	GPYGRCANKTRALTV	Pertussis toxin subunit 2 prec- P04978
3	ELRGSGDLQEYLRHV	Pertussis toxin subunit 2 prec- P04978
4	RGWSIFALYDGTYLG	Pertussis toxin subunit 2 prec- P04978
5	EYGGVIKDGTPGGAF	Pertussis toxin subunit 2 prec- P04978
6	LKTTFCIMTTRNTGQ	Pertussis toxin subunit 2 prec- P04978
7	KEEQYYDYEDATFQT	Pertussis toxin subunit 3 prec- P04979
8	ALTGISLCNPAASIC	Pertussis toxin subunit 3 prec- P04979
9	GQGGARRSRVRALAW	Pertussis toxin subunit 4 prec- P0A3R5
10	LASGAMTHLSPALAD	Pertussis toxin subunit 4 prec- P0A3R5
11	PYVLVKTNMVVTSVA	Pertussis toxin subunit 4 prec- P0A3R5
12	KPYEVTPTRMLVCGI	Pertussis toxin subunit 4 prec- P0A3R5
13	SSGQLSDDGIRRFLG	P.69A protein (pertactin)- CAA09473
14	VTVKAGKLVADHATL	P.69A protein (pertactin)- CAA09473
15	NVGDTWDDDGIALYV	P.69A protein (pertactin)- CAA09473
16	GEQAQASIADSTLQG	P.69A protein (pertactin)- CAA09473
17	GGVQIERGANVTVQR	P.69A protein (pertactin)- CAA09473
18	AIVDGGLHIGALQSL	P.69A protein (pertactin)- CAA09473
19	SGLFRMNVFADLGLS	P.69A protein (pertactin)- CAA09473
20	KLVVMQDASGQHRLW	P.69A protein (pertactin)- CAA09473
21	RNSGSEPASANTLLL	P.69A protein (pertactin)- CAA09473
22	QTPLGSAATFTLANK	P.69A protein (pertactin)- CAA09473
23	GKVDIGTYRYRLAAN	P.69A protein (pertactin)- CAA09473
24	NGQWSLVGAKAPPAP	P.69A protein (pertactin)- CAA09473
25	GTRAELGLGMAAALG	P.69A protein (pertactin)- CAA09473
26	GHSLYASYEYSKGPK	P.69A protein (pertactin)- CAA09473
27	LAMPWTFHAGYRYSW	P.69A protein (pertactin)- CAA09473
28	VRGMLVPVSEHCTVG	filamentous hemagglutinin- AAA22974
29	TFCGRTRGQARSGAR	filamentous hemagglutinin- AAA22974
30	TSLSVAPNALAWALM	filamentous hemagglutinin- AAA22974
31	AGVVSAGKLASGGGA	filamentous hemagglutinin- AAA22974
32	NVAGGGAVKIASASS	filamentous hemagglutinin- AAA22974
33	GNLAVQGGGKVQATL	filamentous hemagglutinin- AAA22974
34	NAGGTLLVSGRQAVQ	filamentous hemagglutinin- AAA22974
35	GAASSRQALSVNAGG	filamentous hemagglutinin- AAA22974
36	LKADKLSATRRVDVD	filamentous hemagglutinin- AAA22974
37	ALGSLAAKGELTVSA	filamentous hemagglutinin- AAA22974
38	RAATVAELKSLDNIS	filamentous hemagglutinin- AAA22974
39	TGGERVSVQSVNSAS	filamentous hemagglutinin- AAA22974
40	VAISAHGALDVGKVS	filamentous hemagglutinin- AAA22974
41	KSGIGLEGWGAVGAD	filamentous hemagglutinin- AAA22974
42	LGSDGAISVSGRDAV	filamentous hemagglutinin- AAA22974
43	GTLLLRNDALTENGT	filamentous hemagglutinin- AAA22974

44	TISADSAVLEHSTIE	filamentous hemagglutinin- AAA22974
45	KISQSVLAAKGDKGK	filamentous hemagglutinin- AAA22974
46	AVSVKVAKKLFLNGT	filamentous hemagglutinin- AAA22974
47	RAVNDNNETMSGRQI	filamentous hemagglutinin- AAA22974
48	VVDGRPQITDAVTGE	filamentous hemagglutinin- AAA22974
49	MQVKEAATIVAASVS	filamentous hemagglutinin- AAA22974
50	PGTFTAGKDITVTSR	filamentous hemagglutinin- AAA22974
51	GFDNEGKMESNKDIV	filamentous hemagglutinin- AAA22974
52	KTEQFSNGRVLDAKH	filamentous hemagglutinin- AAA22974
53	LTVTASGQADNRGSL	filamentous hemagglutinin- AAA22974
54	AGHDFTVQAQRIDNS	filamentous hemagglutinin- AAA22974
55	NEGVIQAGGHGHIGG	filamentous hemagglutinin- AAA22974
56	VDNRSVVRTVSAMEY	filamentous hemagglutinin- AAA22974
57	KTPLPVSLTALDNRA	filamentous hemagglutinin- AAA22974
58	LSPATWNFQSTYELL	filamentous hemagglutinin- AAA22974
59	YLLDQNRYEYIWGLY	filamentous hemagglutinin- AAA22974
60	TYTEWSVNTLKNLDL	filamentous hemagglutinin- AAA22974
61	MVDARGLGSADALAS	filamentous hemagglutinin- AAA22974
62	ASLDAAQGLEVSGRR	filamentous hemagglutinin- AAA22974
63	AQVADAGLAGPSAVA	filamentous hemagglutinin- AAA22974
64	PAVGAADVGVEPVTG	filamentous hemagglutinin- AAA22974
65	QVDQPVVAVGLEQPV	filamentous hemagglutinin- AAA22974
66	TVRVAPPAVALPRPL	filamentous hemagglutinin- AAA22974
67	KSSTPLGSLFAILSS	filamentous hemagglutinin- AAA22974
68	TETNQSAHANHYGTR	filamentous hemagglutinin- AAA22974
69	EAGTLEGKMQNLEIE	filamentous hemagglutinin- AAA22974
70	GSVDAAHTDLSVARD	filamentous hemagglutinin- AAA22974
71	RFKAAADFAHAEHEK	filamentous hemagglutinin- AAA22974
72	VRQLSLGAKVGAGGY	filamentous hemagglutinin- AAA22974
73	SVDAANVSIDAGKDL	filamentous hemagglutinin- AAA22974
74	LSGSRVRGKHVVLDV	filamentous hemagglutinin- AAA22974
75	GDINATSKQDERNYN	filamentous hemagglutinin- AAA22974
76	SGGGWDASAGVAIQN	filamentous hemagglutinin- AAA22974
77	TLVAPVGSAGFNFNT	filamentous hemagglutinin- AAA22974
78	HDNSRLTNDGAAGVV	filamentous hemagglutinin- AAA22974
79	VVETAQPLPPVKPQK	filamentous hemagglutinin- AAA22974
80	TPGPVAEVGKATVTT	filamentous hemagglutinin- AAA22974
81	QVQSAPPKPAPVAKQ	filamentous hemagglutinin- AAA22974
82	APAPKPKPKPKPKAE	filamentous hemagglutinin- AAA22974
83	PKPGKTTPLSGRHVV	filamentous hemagglutinin- AAA22974
84	QQVQVLQRQASDINN	filamentous hemagglutinin- AAA22974
85	TIVITGSISDQTCVI	Serotype 3 fimbrial subunit-CAA35920 P17835
86	EPSTLNHIKVVQLPK	Serotype 3 fimbrial subunit-CAA35920 P17835
87	SKNALRNDGDTAGAT	Serotype 3 fimbrial subunit-CAA35920 P17835
88	FDIKLKECPQALGAL	Serotype 3 fimbrial subunit-CAA35920 P17835
89	LYFEPGITTNYDTGD	Serotype 3 fimbrial subunit-CAA35920 P17835
90	IAYKQTYNASGNGNL	Serotype 3 fimbrial subunit-CAA35920 P17835

91	PPREEGEPGSKSPAD	tracheal colon factor- CAA08832. O86135
92	GQDGPPPPRDGGDAD	tracheal colon factor- CAA08832. O86135
93	QPPRDDGNGEQQPPK	tracheal colon factor- CAA08832. O86135
94	GGDEGQRPPPAAGNG	tracheal colon factor- CAA08832. O86135
95	NGGNGNAQLPERGDD	tracheal colon factor- CAA08832. O86135
96	GPKPPEGEGGDEGPQ	tracheal colon factor- CAA08832. O86135
97	DGGRTELAVGVASQL	tracheal colon factor- CAA08832. O86135
98	KHGSLFGSYEYAKGS	tracheal colon factor- CAA08832. O86135
99	QTMPWTFHVGYRYAW	tracheal colon factor- CAA08832. O86135
100	ADRESGIPAAVLDGI	Bif. hemolysin-adenylate cyclaseprec- P15318
101	AVAKEKNATLMFRLV	Bif. hemolysin-adenylate cyclaseprec- P15318
102	PHSTSLIAEGVATKG	Bif. hemolysin-adenylate cyclaseprec- P15318
103	AYGVAGKSLFDDGLG	Bif. hemolysin-adenylate cyclaseprec- P15318
104	APGVPSGRSKFSPDV	Bif. hemolysin-adenylate cyclaseprec- P15318
105	ETVPASPGLRRPSLG	Bif. hemolysin-adenylate cyclaseprec- P15318
106	VERQDSGYDSLDGVG	Bif. hemolysin-adenylate cyclaseprec- P15318
107	RSFSLGEVSDMAAVE	Bif. hemolysin-adenylate cyclaseprec- P15318
108	AELEMTRQVLHAGAR	Bif. hemolysin-adenylate cyclaseprec- P15318
109	GGPQAYFEKNLQARH	Bif. hemolysin-adenylate cyclaseprec- P15318
110	QLANSDGLRKMLADL	Bif. hemolysin-adenylate cyclaseprec- P15318
111	AGWNASSVIGVQTTE	Bif. hemolysin-adenylate cyclaseprec- P15318
112	SKSALELAAITGNAD	Bif. hemolysin-adenylate cyclaseprec- P15318
113	LKSVDVFVDRFVQGE	Bif. hemolysin-adenylate cyclaseprec- P15318
114	VAGQPVVLDVAAGGI	Bif. hemolysin-adenylate cyclaseprec- P15318
115	EKWPALNLFSVDHVK	Bif. hemolysin-adenylate cyclaseprec- P15318
116	IENLHGSRLNDRIAG	Bif. hemolysin-adenylate cyclaseprec- P15318
117	DQDNELWGHDGNDTI	Bif. hemolysin-adenylate cyclaseprec- P15318
118	GRGGDDILRGGLGLD	Bif. hemolysin-adenylate cyclaseprec- P15318
119	LYGEDGNDIFLQDDE	Bif. hemolysin-adenylate cyclaseprec- P15318
120	VSDDIDGGAGLDTVD	Bif. hemolysin-adenylate cyclaseprec- P15318
121	FLSLGKGFASLMDEP	Bif. hemolysin-adenylate cyclaseprec- P15318
122	TSNVLRNIENAVGSA	Bif. hemolysin-adenylate cyclaseprec- P15318
123	DDVLIGDAGANVLNG	Bif. hemolysin-adenylate cyclaseprec- P15318
124	AGNDVLSGGAGDDVL	Bif. hemolysin-adenylate cyclaseprec- P15318
125	GDEGSDLLSGDAGND	Bif. hemolysin-adenylate cyclaseprec- P15318
126	LFGGQGDDTYLFGVG	Bif. hemolysin-adenylate cyclaseprec- P15318
127	NDKDADAVNRVGFAT	Out. M.porin protein prec-CAA41398.1 Q04064
128	DNVRAITTGLRYVNG	Out. M.porin protein prec-CAA41398.1 Q04064
129	LNVALSYDQLNASNN	Out. M.porin protein prec-CAA41398.1 Q04064
130	AQGEVDATPRSYGLG	Out. M.porin protein prec-CAA41398.1 Q04064
131	SYDFEVVKLALAYAR	Out. M.porin protein prec-CAA41398.1 Q04064
132	TDGWFGGQGYPVAVT	Out. M.porin protein prec-CAA41398.1 Q04064
133	KYEDGPLLAAFTWDK	Out. M.porin protein OmpQ- CAD12825. Q8VV98
134	NLHDTSATGGRSPQA	Out. M.porin protein OmpQ- CAD12825. Q8VV98
135	QAGFTYDFEALKMAL	Out. M.porin protein OmpQ- CAD12825. Q8VV98
136	WSRQRNGFVGLNGGG	Out. M.porin protein OmpQ- CAD12825. Q8VV98
137	IGLGPEPFAHGGAIN	Out. M.porin protein OmpQ- CAD12825. Q8VV98

138	WLLGLEVPVHGNGAW	Out. M.porin protein OmpQ- CAD12825. Q8VV98
139	RINRGRMRGNMDVAF	GTP-binding elongation factor- Q7VYR0
140	FGPDGEVQRGRINQV	GTP-binding elongation factor- Q7VYR0
141	KFSGLERVVVDEAEA	GTP-binding elongation factor- Q7VYR0
142	DIVLVNGIEDLHIGS	GTP-binding elongation factor- Q7VYR0
143	ITDPSTPEGLPVLRI	GTP-binding elongation factor- Q7VYR0
144	EPTLTMNFMVNTSPL	GTP-binding elongation factor- Q7VYR0
145	LQIPRSAWRLHALAA	Bordetella resistance to killing- AAA51646
146	LALAGMARLAPAAAQ	Bordetella resistance to killing- AAA51646
147	PQPPVAGAPHAQDAG	Bordetella resistance to killing- AAA51646
148	EGEFDHRDNTLIAVF	Bordetella resistance to killing- AAA51646
149	DGVGINLDDDPDELG	Bordetella resistance to killing- AAA51646
150	TAPPTLKDIHISVEH	Bordetella resistance to killing- AAA51646
151	ISVLGFEPQSGSGPA	Bordetella resistance to killing- AAA51646
152	VDMQGGSITTTGNRA	Bordetella resistance to killing- AAA51646
153	GIALTHGSARLEGVA	Bordetella resistance to killing- AAA51646
154	RAEGSGSSAAQLANG	Bordetella resistance to killing- AAA51646
155	LVVSAGSLASAQSGA	Bordetella resistance to killing- AAA51646
156	SVTDTPLKLMPGALA	Bordetella resistance to killing- AAA51646
157	RGWSASGGRWYAGGL	Bordetella resistance to killing- AAA51646
158	GYTYADRTYPGDGGG	Bordetella resistance to killing- AAA51646
159	VKGLHVGGYAAYVGD	Bordetella resistance to killing- AAA51646
160	GYYLDTVLRLGRYDQ	Bordetella resistance to killing- AAA51646
161	YNIAGTDGGRVTADY	Bordetella resistance to killing- AAA51646
162	TSGAAWSLEGGRRFE	Bordetella resistance to killing- AAA51646
163	SATLNDVALETAGQQ	Vag8 protein (Autotr.) CAD12828 Q8VV95
164	PAVVLWQGAQLNAQG	Vag8 protein (Autotr.) CAD12828 Q8VV95
165	VVQVNGAGVSAIHAQ	Vag8 protein (Autotr.) CAD12828 Q8VV95
166	AGSFTLSGSDITARG	Vag8 protein (Autotr.) CAD12828 Q8VV95
167	EVAGIYVQEGMQGTL	Vag8 protein (Autotr.) CAD12828 Q8VV95
168	GTRVTTQGDTAPALQ	Vag8 protein (Autotr.) CAD12828 Q8VV95
169	DDGTESGATKVPLIE	Vag8 protein (Autotr.) CAD12828 Q8VV95
170	EQGHTAFTLGNMGGR	Vag8 protein (Autotr.) CAD12828 Q8VV95
171	DAGARQYELTASEAQ	Vag8 protein (Autotr.) CAD12828 Q8VV95
172	DKARTWQLTPTNELS	Vag8 protein (Autotr.) CAD12828 Q8VV95
173	TATAAVNAMAIAASQ	Vag8 protein (Autotr.) CAD12828 Q8VV95
174	IWQAEMDVLLRHMSG	Vag8 protein (Autotr.) CAD12828 Q8VV95
175	NNVAVVATGSGKVAI	putative autotransporter- AAC31207
176	NAELLGASGMYATFG	putative autotransporter- AAC31207
177	QVDMKGGRILAHNTN	putative autotransporter- AAC31207
178	LGSQGYADGPYGGVV	putative autotransporter- AAC31207
179	TEDGQVNLEGAKVSA	putative autotransporter- AAC31207
180	GLGAAGLWLLGDKDT	putative autotransporter- AAC31207
181	ELPEGSQTKFTLANR	putative autotransporter- AAC31207
182	GVVDAGAFRYRLTPD	putative autotransporter- AAC31207
183	GVWGLERTSQLSAVA	putative autotransporter- AAC31207
184	AALNTGGVGAASSIW	putative autotransporter- AAC31207

185	AEGNALSKRLGELRL	putative autotransporter- AAC31207
186	PGAGGFWGRTFAQKQ	putative autotransporter- AAC31207
187	TEYSNARYVSQQTRA	Pertussis toxin subunit 1 prec- O69258
188	PNPYTSRRSVASIVG	Pertussis toxin subunit 1 prec- O69258
189	LVRIAPVIGACMARQ	Pertussis toxin subunit 1 prec- O69258
190	ESSEAMAAWSERAGE	Pertussis toxin subunit 1 prec- O69258
191	EAMVLVYYESIAYSF	Pertussis toxin subunit 1 prec- O69258
192	SVLPLALLGSHVARA	Pertussis toxin subunit 2 prec- P04978
193	DAPPGAGFIYRETFC	Pertussis toxin subunit 3 prec- P04979
194	TTIYKTGQPAADHYY	Pertussis toxin subunit 3 prec- P04979
195	KVTATRLLASTNSRL	Pertussis toxin subunit 3 prec- P04979
196	AVFVRDGQSVIGACA	Pertussis toxin subunit 3 prec- P04979
197	PYEGRYRDMYDALRR	Pertussis toxin subunit 3 prec- P04979
198	LYMIYMSGLAVRVHV	Pertussis toxin subunit 3 prec- P04979
199	MNMSLSRIVKAAPLR	P.69A protein (pertactin)- CAA09473
200	TTLAMALGALGAAPA	P.69A protein (pertactin)- CAA09473
201	HADWNNQSIVKTGER	P.69A protein (pertactin)- CAA09473
202	HGIHIQGSDPGGVRT	P.69A protein (pertactin)- CAA09473
203	SGTTIKVSGRQAQGI	P.69A protein (pertactin)- CAA09473
204	LENPAAELQFRNGSV	P.69A protein (pertactin)- CAA09473
205	LTLTGGADAQGDIVA	P.69A protein (pertactin)- CAA09473
206	ELPSIPGTSIGPLDV	P.69A protein (pertactin)- CAA09473
207	LASQARWTGATRAVD	P.69A protein (pertactin)- CAA09473
208	LSIDNATWVMTDNSN	P.69A protein (pertactin)- CAA09473
209	GALRLASDGSVDFQQ	P.69A protein (pertactin)- CAA09473
210	AEAGRFKVLTVNTLA	P.69A protein (pertactin)- CAA09473
211	EAGRRFTHADGWFLE	P.69A protein (pertactin)- CAA09473
212	QAELAVFRAGGGAYR	P.69A protein (pertactin)- CAA09473
213	ANGLRVRDEGGSSVL	P.69A protein (pertactin)- CAA09473
214	RLGLEVGKRIELAGG	P.69A protein (pertactin)- CAA09473
215	QVQPYIKASVLQEFD	P.69A protein (pertactin)- CAA09473
216	AGTVHTNGIAHRTEL	P.69A protein (pertactin)- CAA09473
217	ADIAVVAGANRYDHA	filamentous hemagglutinin- AAA22974
218	RRATPIAAGARGAAA	filamentous hemagglutinin- AAA22974
219	AYAIDGTAAGAMYGK	filamentous hemagglutinin- AAA22974
220	ITLVSSDSGLGVRQL	filamentous hemagglutinin- AAA22974
221	SLSSPSAITVSSQGE	filamentous hemagglutinin- AAA22974
222	ALGDATVQRGPLSLK	filamentous hemagglutinin- AAA22974
223	VSAKDMRSRGAVTVS	filamentous hemagglutinin- AAA22974
224	GGAVNLGDVQSDGQV	filamentous hemagglutinin- AAA22974
225	ATSAGAMTVRDVAAA	filamentous hemagglutinin- AAA22974
226	DLALQAGDALQAGFL	filamentous hemagglutinin- AAA22974
227	SAGAMTVNGRDAVRL	filamentous hemagglutinin- AAA22974
228	GAHAGGQLRVSSDGQ	filamentous hemagglutinin- AAA22974
229	AAEVAGALELSGQGV	filamentous hemagglutinin- AAA22974
230	VDRASASRARIDSTG	filamentous hemagglutinin- AAA22974
231	VGIGALKAGAVEAAS	filamentous hemagglutinin- AAA22974

232	RRARRALRQDFFTPG	filamentous hemagglutinin- AAA22974
233	VVVRAQGNVTVGRGD	filamentous hemagglutinin- AAA22974
234	HQGVLAQGDIIMDAK	filamentous hemagglutinin- AAA22974
235	GDYTVSADAIALAAQ	filamentous hemagglutinin- AAA22974
236	TQRGGAANLTSRHDT	filamentous hemagglutinin- AAA22974
237	FSNKIRLMGPLQVNA	filamentous hemagglutinin- AAA22974
238	GPVSNTGNLKVREGV	filamentous hemagglutinin- AAA22974
239	VTAASFDNETGAEVM	filamentous hemagglutinin- AAA22974
240	KSATLTTSGAARNAG	filamentous hemagglutinin- AAA22974
241	AGEDMHLDAPRIENT	filamentous hemagglutinin- AAA22974
242	KLSGEVQRKGVQDVG	filamentous hemagglutinin- AAA22974
243	GEHGRWSGIGYVNYW	filamentous hemagglutinin- AAA22974
244	RAGNGKKAGTIAAPW	filamentous hemagglutinin- AAA22974
245	GGDLTAEQSLIEVGK	filamentous hemagglutinin- AAA22974
246	LYLNAGARKDEHRHL	filamentous hemagglutinin- AAA22974
247	TLSNGAIHNGENAAQ	filamentous hemagglutinin- AAA22974
248	RGRPEGLKIGAHSAT	filamentous hemagglutinin- AAA22974
249	VSGSFDALRDVGLEK	filamentous hemagglutinin- AAA22974
250	LDIDDALAAVLVNPH	filamentous hemagglutinin- AAA22974
251	FTRIGAAQTSLADGA	filamentous hemagglutinin- AAA22974
252	GPALARQARQAPETD	filamentous hemagglutinin- AAA22974
253	VDVSGRDIGIEGGKL	filamentous hemagglutinin- AAA22974
254	GKDVRLKADTVKVAT	filamentous hemagglutinin- AAA22974
255	MRYDDKGRLAARGDG	filamentous hemagglutinin- AAA22974
256	LDAQGGQLHIEAKRL	filamentous hemagglutinin- AAA22974
257	TAGATLKGGKVKLDV	filamentous hemagglutinin- AAA22974
258	DVKLGGVYEAGSSYE	filamentous hemagglutinin- AAA22974
259	YLGGNLSIEATEGDA	filamentous hemagglutinin- AAA22974
260	LVGAKFGGGDQVSLK	filamentous hemagglutinin- AAA22974
261	AKSVNLMAAESTFES	filamentous hemagglutinin- AAA22974
262	SESHNFHASADANLG	filamentous hemagglutinin- AAA22974
263	NAVQGAVGLGLTAGM	filamentous hemagglutinin- AAA22974
264	TSHQITNETGKTYAG	filamentous hemagglutinin- AAA22974
265	PKVETAQPLPPRPVA	filamentous hemagglutinin- AAA22974
266	QVVPVTPPKVEVAKV	filamentous hemagglutinin- AAA22974
267	VVPRPKVETAQPLPP	filamentous hemagglutinin- AAA22974
268	PVVAEKVTTPAVQPQ	filamentous hemagglutinin- AAA22974
269	AKVETVQPVKPETTK	filamentous hemagglutinin- AAA22974
270	LPKPLPVAKVTKAPP	filamentous hemagglutinin- AAA22974
271	TAATEAQGVQVRISN	Fim2 pilic subunit- CAD12823.1 Q8VVA0
272	NDSKITMGANEATQQ	Fim2 pilic subunit- CAD12823.1 Q8VVA0
273	AGFDPEVQTGGTSKT	Fim2 pilic subunit- CAD12823.1 Q8VVA0
274	TMRYLASYVKKNGDV	Fim2 pilic subunit- CAD12823.1 Q8VVA0
275	ASAITTYVGFSVVYP	Fim2 pilic subunit- CAD12823.1 Q8VVA0
276	LILAASPVLPALAND	Serotype 3 fimbrial subunit-CAA35920 P17835
277	SSGGHNPGVGGGTHE	tracheal colon factor- CAA08832. O86135
278	GLPGIGKVGGSAPGP	tracheal colon factor- CAA08832. O86135

279	TSTGSGPDAGMASGA	tracheal colon factor- CAA08832. O86135
280	STSPGASGGAGKDAM	tracheal colon factor- CAA08832. O86135
281	PSEGERPDSGMSDSG	tracheal colon factor- CAA08832. O86135
282	GGESSAGGLNPDGAG	tracheal colon factor- CAA08832. O86135
283	LELGKRFTWPGAWYV	tracheal colon factor- CAA08832. O86135
284	PQLEVAAFHAQGADY	tracheal colon factor- CAA08832. O86135
285	ASNGLRIKDDGTNSM	tracheal colon factor- CAA08832. O86135
286	GRLGLHVGRQFDLGD	tracheal colon factor- CAA08832. O86135
287	RVVQPYMKLSWVQEF	tracheal colon factor- CAA08832. O86135
288	GKGTVRTNDIRHKVR	tracheal colon factor- CAA08832. O86135
289	VGTEARRQFRYDGDM	Bif. hemolysin-adenylate cyclaseprec- P15318
290	IGVITDFELEVRNAL	Bif. hemolysin-adenylate cyclaseprec- P15318
291	RRAHAVGAQDVVQHG	Bif. hemolysin-adenylate cyclaseprec- P15318
292	EQNNPFPEADEKIFV	Bif. hemolysin-adenylate cyclaseprec- P15318
293	SATGESQMLTRGQLK	Bif. hemolysin-adenylate cyclaseprec- P15318
294	YIGQQRGEGYVFYEN	Bif. hemolysin-adenylate cyclaseprec- P15318
295	DKLAQESSAYGYEGD	Bif. hemolysin-adenylate cyclaseprec- P15318
296	LLAQLYRDKTAAEGA	Bif. hemolysin-adenylate cyclaseprec- P15318
297	AGVSAVLSTVGAAVS	Bif. hemolysin-adenylate cyclaseprec- P15318
298	AAAASVVGAPVAVVT	Bif. hemolysin-adenylate cyclaseprec- P15318
299	LLTGALNGILRGVQQ	Bif. hemolysin-adenylate cyclaseprec- P15318
300	IIEKLANDYARKIDE	Bif. hemolysin-adenylate cyclaseprec- P15318
301	SITGNAHDNFLAGGS	Bif. hemolysin-adenylate cyclaseprec- P15318
302	DDRLDGGAGNDTLVG	Bif. hemolysin-adenylate cyclaseprec- P15318
303	EGQNTVIGGAGDDVF	Bif. hemolysin-adenylate cyclaseprec- P15318
304	QDLGVWSNQLDGGAG	Bif. hemolysin-adenylate cyclaseprec- P15318
305	DTVKYNVHQPSEERL	Bif. hemolysin-adenylate cyclaseprec- P15318
306	RMGDTGIHADLQKGT	Bif. hemolysin-adenylate cyclaseprec- P15318
307	ELADRITGDAQANVL	Bif. hemolysin-adenylate cyclaseprec- P15318
308	GAGGADVLAGGEGDD	Bif. hemolysin-adenylate cyclaseprec- P15318
309	LLGGDGDDQLSGDAG	Bif. hemolysin-adenylate cyclaseprec- P15318
310	DRLYGEAGDDWFFQD	Bif. hemolysin-adenylate cyclaseprec- P15318
311	ANAGNLLDGGDGRDT	Bif. hemolysin-adenylate cyclaseprec- P15318
312	DFSGPGRGLDAGAKG	Bif. hemolysin-adenylate cyclaseprec- P15318
313	GFNSGNGNSAQDGRL	Out. M.porin protein prec-CAA41398.1 Q04064
314	GRQATIGLQSESWGR	Out. M.porin protein prec-CAA41398.1 Q04064
315	DFGRQTNIASKYFGS	Out. M.porin protein prec-CAA41398.1 Q04064
316	DPFGAGFGQANIGMG	Out. M.porin protein prec-CAA41398.1 Q04064
317	SAMNTVRYDNMVMYQ	Out. M.porin protein prec-CAA41398.1 Q04064
318	PSYSGFQFGIGYSFS	Out. M.porin protein prec-CAA41398.1 Q04064
319	DYGTWVGLGHAGVGE	Out. M.porin protein OmpQ- CAD12825. Q8VV98
320	SLGRQQSIGLQYGGQ	Out. M.porin protein OmpQ- CAD12825. Q8VV98
321	EIASWRDMGMGALFK	Out. M.porin protein OmpQ- CAD12825. Q8VV98
322	SDNYRVNNLVNYLSP	Out. M.porin protein OmpQ- CAD12825. Q8VV98
323	FSGWQWGVGYAFDVE	Out. M.porin protein OmpQ- CAD12825. Q8VV98
324	GDTGRFDRSPAFSTG	Out. M.porin protein OmpQ- CAD12825. Q8VV98
325	KIDRPGARPDFVINA	GTP-binding elongation factor- Q7VYR0

326	FELFDKLGATEEQLD	GTP-binding elongation factor- Q7VYR0
327	PVVYASGLSGYAGLT	GTP-binding elongation factor- Q7VYR0
328	DVRSGDMRPLFEAIM	GTP-binding elongation factor- Q7VYR0
329	YVPQRDDDANGPLQM	GTP-binding elongation factor- Q7VYR0
330	IISLDYNSYVGKIGV	GTP-binding elongation factor- Q7VYR0
331	VSPGEPLYEGMIIGI	GTP-binding elongation factor- Q7VYR0
332	SRDNDLVVNPIKGKQ	GTP-binding elongation factor- Q7VYR0
333	TNVRASGTDEAVRLV	GTP-binding elongation factor- Q7VYR0
334	PIQMSLEYAVEFIDD	GTP-binding elongation factor- Q7VYR0
335	ELVEITPKSIRLRKR	GTP-binding elongation factor- Q7VYR0
336	YLQEHERRRASRENA	GTP-binding elongation factor- Q7VYR0
337	GTVSVQGDDGAGVVA	Bordetella resistance to killing- AAA51646
338	AGLLDALPPGGTVRL	Bordetella resistance to killing- AAA51646
339	GTTVSTDGANTDAVL	Bordetella resistance to killing- AAA51646
340	RGDAARAEVVNTVLR	Bordetella resistance to killing- AAA51646
341	AKSLAAGVSAQHGGR	Bordetella resistance to killing- AAA51646
342	TLRQTRIETAGAGAE	Bordetella resistance to killing- AAA51646
343	GTWRYSLAEDPKTHV	Bordetella resistance to killing- AAA51646
344	SLQRAGQALSGAANA	Bordetella resistance to killing- AAA51646
345	VNAADLSSIALAESN	Bordetella resistance to killing- AAA51646
346	LDKRLGELRLRADAG	Bordetella resistance to killing- AAA51646
347	PWARTFSERQQISNR	Bordetella resistance to killing- AAA51646
348	ARAYDQTVSGLEIGL	Bordetella resistance to killing- AAA51646
349	NVTGRTAEVRVTGGT	Vag8 protein (Autotr.) CAD12828 Q8VV95
350	RTSGNQAQGLRVGTE	Vag8 protein (Autotr.) CAD12828 Q8VV95
351	APDNTALGASVFLQN	Vag8 protein (Autotr.) CAD12828 Q8VV95
352	IIETSGTGALGVSVH	Vag8 protein (Autotr.) CAD12828 Q8VV95
353	PQGGGGTRLSMSGTT	Vag8 protein (Autotr.) CAD12828 Q8VV95
354	RTRGDDSFALQLSGP	Vag8 protein (Autotr.) CAD12828 Q8VV95
355	WAGRTDGAVHTVRLL	Vag8 protein (Autotr.) CAD12828 Q8VV95
356	RGVWTVTGDSRVAEV	Vag8 protein (Autotr.) CAD12828 Q8VV95
357	LEGGTLAFAPPAQPK	Vag8 protein (Autotr.) CAD12828 Q8VV95
358	AFKTLVATQGISGTG	Vag8 protein (Autotr.) CAD12828 Q8VV95
359	IVMNAHLPSGTADVL	Vag8 protein (Autotr.) CAD12828 Q8VV95
360	APQGFGDRQVLVVNN	Vag8 protein (Autotr.) CAD12828 Q8VV95
361	NIVEPYARLGWAQEL	Vag8 protein (Autotr.) CAD12828 Q8VV95
362	ADNAVYTNGIRHVTR	Vag8 protein (Autotr.) CAD12828 Q8VV95
363	RGGFAEARVGVGALL	Vag8 protein (Autotr.) CAD12828 Q8VV95
364	KRHALYADYEYAKGA	Vag8 protein (Autotr.) CAD12828 Q8VV95
365	FEAPWTLQLGYRYSW	Vag8 protein (Autotr.) CAD12828 Q8VV95
366	PSIRVQGGVVQGGMG	putative autotransporter- AAC31207
367	LRIEDGTWTVTGSST	putative autotransporter- AAC31207
368	NSLHLQAGKVAYATP	putative autotransporter- AAC31207
369	ESDGEFKHLRVKTLS	putative autotransporter- AAC31207
370	SGLFEMNASADLSDG	putative autotransporter- AAC31207
371	LLVVSDEASGQHKVL	putative autotransporter- AAC31207
372	RGAGTEPTGVESLTL	putative autotransporter- AAC31207

373	VRTNGYGLRTDLSGG	putative autotransporter- AAC31207
374	AELALGLAAALGRGH	putative autotransporter- AAC31207
375	LYTSYEYAKGNKLTL	putative autotransporter- AAC31207
376	YLEHRMQEAVEAERA	Pertussis toxin subunit 1 prec- O69258
377	RGTGHFIGYIYEVRA	Pertussis toxin subunit 1 prec- O69258
378	NNFYGAASSYFEYVD	Pertussis toxin subunit 1 prec- O69258
379	YGDNAGRILAGALAT	Pertussis toxin subunit 1 prec- O69258
380	QSEYLAHRRIPPENI	Pertussis toxin subunit 1 prec- O69258
381	RVTRVYHNGITGETT	Pertussis toxin subunit 1 prec- O69258
382	NKKLLHHILPILVLA	Pertussis toxin subunit 3 prec- P04979
383	LGMRTAQAVAPGIVI	Pertussis toxin subunit 3 prec- P04979
384	PKALFTQQGGAYGRC	Pertussis toxin subunit 3 prec- P04979
385	NGTRALTVAELRGNA	Pertussis toxin subunit 3 prec- P04979
386	LQTYLRQITPGWSIY	Pertussis toxin subunit 3 prec- P04979
387	LYDGTYLGQAYGGII	Pertussis toxin subunit 3 prec- P04979
388	SPADVAGLPTHLYKN	Pertussis toxin subunit 5 prec- P04981
389	TVQELALKLKGKNQE	Pertussis toxin subunit 5 prec- P04981
390	CLTAFMSGRSLVRAC	Pertussis toxin subunit 5 prec- P04981
391	SDAGHEHDTWFDTML	Pertussis toxin subunit 5 prec- P04981
392	FAISAYALKSRIALT	Pertussis toxin subunit 5 prec- P04981
393	EDSPYPGTPGDLLEL	Pertussis toxin subunit 5 prec- P04981
394	GPVLDGWYGVDVSGS	P.69A protein (pertactin)- CAA09473
395	VELAQSIVEAPELGA	P.69A protein (pertactin)- CAA09473
396	IRVGRGARVTVSGGS	P.69A protein (pertactin)- CAA09473
397	SAPHGNVIETGGARR	P.69A protein (pertactin)- CAA09473
398	APQAAPLSITLQAGA	P.69A protein (pertactin)- CAA09473
399	AQGKALLYRVLPEPV	P.69A protein (pertactin)- CAA09473
400	AGFELGADHAVAVAG	P.69A protein (pertactin)- CAA09473
401	RWHLGGLAGYTRGDR	P.69A protein (pertactin)- CAA09473
402	FTGDGGGHTDSVHVG	P.69A protein (pertactin)- CAA09473
403	YATYIADSGFYLDAT	P.69A protein (pertactin)- CAA09473
404	RASRLENDFKVAGSD	P.69A protein (pertactin)- CAA09473
405	YAVKGKYRTHGVGAS	P.69A protein (pertactin)- CAA09473
406	SRLAGTLEVYGKGAD	filamentous hemagglutinin- AAA22974
407	IIANPNGISVNGLST	filamentous hemagglutinin- AAA22974
408	NASNLTLTTGRPSVN	filamentous hemagglutinin- AAA22974
409	GRIGLDVQQGTVTIE	filamentous hemagglutinin- AAA22974
410	GGVNATGLGYFDVVA	filamentous hemagglutinin- AAA22974
411	LVKLQGAVSSKQGKP	filamentous hemagglutinin- AAA22974
412	ALAARNLQSKGAIGV	filamentous hemagglutinin- AAA22974
413	GGEAVSVANANSDAE	filamentous hemagglutinin- AAA22974
414	RVRGRGQVDLHDLSA	filamentous hemagglutinin- AAA22974
415	RGADISGEGRVNIGR	filamentous hemagglutinin- AAA22974
416	RSDSDVKVSAHGALS	filamentous hemagglutinin- AAA22974
417	DSMTALGAIGVQAGG	filamentous hemagglutinin- AAA22974
418	QSAKASGTLHVQGGE	filamentous hemagglutinin- AAA22974
419	LDLGTLAAVGAVDVN	filamentous hemagglutinin- AAA22974

420	TGDVRVAKLVSDAGA	filamentous hemagglutinin- AAA22974
421	LQAGRSMTLGIVDTT	filamentous hemagglutinin- AAA22974
422	DLQARAQQKLELGSV	filamentous hemagglutinin- AAA22974
423	SDGGLQAAAGGALSL	filamentous hemagglutinin- AAA22974
424	TFQSQAPAGTAGALV	filamentous hemagglutinin- AAA22974
425	KAAEAIVHDGVMATK	filamentous hemagglutinin- AAA22974
426	EMQIAGKGGGSPTVT	filamentous hemagglutinin- AAA22974
427	GAKATTSANKLSVDV	filamentous hemagglutinin- AAA22974
428	SWDNAGSLDIKKGGA	filamentous hemagglutinin- AAA22974
429	VTVAGRYAEHGEVSI	filamentous hemagglutinin- AAA22974
430	AKSLTTEIETGNPGS	filamentous hemagglutinin- AAA22974
431	IAEVQENIDNKQAIV	filamentous hemagglutinin- AAA22974
432	GKDLTLSSAHGNVAN	filamentous hemagglutinin- AAA22974
433	ANALLWAAGELTVKA	filamentous hemagglutinin- AAA22974
434	NITNKRAALIEAGGN	filamentous hemagglutinin- AAA22974
435	RLTAAVALLNKLGRI	filamentous hemagglutinin- AAA22974
436	LRTVYAKQADQATID	filamentous hemagglutinin- AAA22974
437	ETDKVAQRYKSQIDA	filamentous hemagglutinin- AAA22974
438	RLQAIQPGRVTLAKA	filamentous hemagglutinin- AAA22974
439	SAALGADWRALGHSQ	filamentous hemagglutinin- AAA22974
440	MQRWKDFKAGKRGAE	filamentous hemagglutinin- AAA22974
441	AFYPKEQTVLAAGAG	filamentous hemagglutinin- AAA22974
442	DRDFVWYVDTVIDGQ	filamentous hemagglutinin- AAA22974
443	VLAPRLYLTEATRQG	filamentous hemagglutinin- AAA22974
444	TDQYAGGGALIASGG	filamentous hemagglutinin- AAA22974
445	VTVNTDGHDVSSVNG	filamentous hemagglutinin- AAA22974
446	IQGRSVKVDAGKGKV	filamentous hemagglutinin- AAA22974
447	VADSKGAGGGIEADD	filamentous hemagglutinin- AAA22974
448	TKYVSEQTSQSSGWS	filamentous hemagglutinin- AAA22974
449	EVASTASARSSLLTA	filamentous hemagglutinin- AAA22974
450	TRLGDSVAQNVEDGR	filamentous hemagglutinin- AAA22974
451	IRGELMAAQVAAEAT	filamentous hemagglutinin- AAA22974
452	LVTADTAAVALSAGI	filamentous hemagglutinin- AAA22974
453	ADFDSSHSRSTSQNT	filamentous hemagglutinin- AAA22974
454	QVGGGVKGTLNQDAA	filamentous hemagglutinin- AAA22974
455	ATVVQRNKHWAGGGS	filamentous hemagglutinin- AAA22974
456	FSVAGKSLKKKNQVR	filamentous hemagglutinin- AAA22974
457	VETPTPDVVDGPPSR	filamentous hemagglutinin- AAA22974
458	TTPPASPQPIRATVE	filamentous hemagglutinin- AAA22974
459	SSPPPVSVATVEVVP	filamentous hemagglutinin- AAA22974
460	ITGTITDTTCVIEDP	Fim2 pilic subunit- CAD12823.1 Q8VVA0
461	GPNHTKVVQLPKISK	Fim2 pilic subunit- CAD12823.1 Q8VVA0
462	ALKANGDQAGRTPFI	Fim2 pilic subunit- CAD12823.1 Q8VVA0
463	KLKDCPSSLGNGVKA	Fim2 pilic subunit- CAD12823.1 Q8VVA0
464	FEPGPTTDYSTGDLR	Fim2 pilic subunit- CAD12823.1 Q8VVA0
465	YKMVYATNPQTQLSN	Fim2 pilic subunit- CAD12823.1 Q8VVA0
466	LGAGMWTLSPPSAWA	tracheal colon factor- CAA08832. O86135

467	KLPSLLTDDELKLVL	tracheal colon factor- CAA08832. O86135
468	TGMSLEDFKRSLQES	tracheal colon factor- CAA08832. O86135
469	PSALATPPSSSPPVA	tracheal colon factor- CAA08832. O86135
470	PGPGSVAEAPSGSGH	tracheal colon factor- CAA08832. O86135
471	DNPSPPVVGVGPGMA	tracheal colon factor- CAA08832. O86135
472	ISGFELGADTALPVA	tracheal colon factor- CAA08832. O86135
473	GRWHVGAVAGYTNGR	tracheal colon factor- CAA08832. O86135
474	KFDRGGTGDDDSVHV	tracheal colon factor- CAA08832. O86135
475	AYATYIEDGGFYMDG	tracheal colon factor- CAA08832. O86135
476	VRVSRIRHAFKVDDA	tracheal colon factor- CAA08832. O86135
477	GRRVRGQYRGNGVGA	tracheal colon factor- CAA08832. O86135
478	GRYAVQYRRKGGDDF	Bif. hemolysin-adenylate cyclaseprec- P15318
479	AVKVIGNAAGIPLTA	Bif. hemolysin-adenylate cyclaseprec- P15318
480	IDMFAIMPHLSNFRD	Bif. hemolysin-adenylate cyclaseprec- P15318
481	ARSSVTSGDSVTDYL	Bif. hemolysin-adenylate cyclaseprec- P15318
482	RTRRAASEATGGLDR	Bif. hemolysin-adenylate cyclaseprec- P15318
483	RIDLLWKIARAGARS	Bif. hemolysin-adenylate cyclaseprec- P15318
484	LGGGIAAAVGAGMSL	Bif. hemolysin-adenylate cyclaseprec- P15318
485	DDAPAGQKAAAGAEI	Bif. hemolysin-adenylate cyclaseprec- P15318
486	LQLTGGTVELASSIA	Bif. hemolysin-adenylate cyclaseprec- P15318
487	ALAAARGVTSGLQVA	Bif. hemolysin-adenylate cyclaseprec- P15318
488	ASAGAAAGALAAALS	Bif. hemolysin-adenylate cyclaseprec- P15318
489	MEIYGLVQQSHYADQ	Bif. hemolysin-adenylate cyclaseprec- P15318
490	SRIHYDGGAGTNTVS	Bif. hemolysin-adenylate cyclaseprec- P15318
491	AALGRQDSITVSADG	Bif. hemolysin-adenylate cyclaseprec- P15318
492	RFNVRKQLNNANVYR	Bif. hemolysin-adenylate cyclaseprec- P15318
493	GVATQTTAYGKRTEN	Bif. hemolysin-adenylate cyclaseprec- P15318
494	QYRHVELARVGQLVE	Bif. hemolysin-adenylate cyclaseprec- P15318
495	DTLEHVQHIIGGAGN	Bif. hemolysin-adenylate cyclaseprec- P15318
496	AGNDTLYGGLGDDTL	Bif. hemolysin-adenylate cyclaseprec- P15318
497	GGAGNDWFGQTQARE	Bif. hemolysin-adenylate cyclaseprec- P15318
498	DVLRGGDGVDTVDYS	Bif. hemolysin-adenylate cyclaseprec- P15318
499	TGAHAGIAAGRIGLG	Bif. hemolysin-adenylate cyclaseprec- P15318
500	LADLGAGRVDKLGEA	Bif. hemolysin-adenylate cyclaseprec- P15318
501	SSAYDTVSGIENVVG	Bif. hemolysin-adenylate cyclaseprec- P15318
502	RVPDTLMQSLAVNWR	Bif. hemolysin-adenylate cyclaseprec- P15318
503	GFAGAAQAETSVTLY	Out. M.porin protein prec-CAA41398.1 Q04064
504	IIDTGIGYNDVDFKV	Out. M.porin protein prec-CAA41398.1 Q04064
505	GANADDSDFKYNHSR	Out. M.porin protein prec-CAA41398.1 Q04064
506	GMINGVQNGSRWGLR	Out. M.porin protein prec-CAA41398.1 Q04064
507	TEDLGDGLQAVFQLE	Out. M.porin protein prec-CAA41398.1 Q04064
508	LVVAAMAAGSSSVFW	Out. M.porin protein OmpQ- CAD12825. Q8VV98
509	VAPARAANQLELYGV	Out. M.porin protein OmpQ- CAD12825. Q8VV98
510	DVGLATTRVSGLGTR	Out. M.porin protein OmpQ- CAD12825. Q8VV98
511	QVLGGGQTDNLWGLR	Out. M.porin protein OmpQ- CAD12825. Q8VV98
512	TEELDGGWRASFGLE	Out. M.porin protein OmpQ- CAD12825. Q8VV98
513	GFDAANGTRNDDARL	Out. M.porin protein OmpQ- CAD12825. Q8VV98

514	RENQSVAERVMDSND	GTP-binding elongation factor- Q7VYR0
515	EKERGITILAKNCAV	GTP-binding elongation factor- Q7VYR0
516	YEGTHINIVDTPGHA	GTP-binding elongation factor- Q7VYR0
517	FGGEVERVLSMVDGV	GTP-binding elongation factor- Q7VYR0
518	LLVDAVEGPMPQTIF	GTP-binding elongation factor- Q7VYR0
519	TRKALALGLKPIVVV	GTP-binding elongation factor- Q7VYR0
520	MEELGRRKGDLQDMQ	GTP-binding elongation factor- Q7VYR0
521	DGRGRTRLEYIIPAR	GTP-binding elongation factor- Q7VYR0
522	LIGFQNEFLTLTRGT	GTP-binding elongation factor- Q7VYR0
523	LMSHIFHEYAPLREG	GTP-binding elongation factor- Q7VYR0
524	IGERRNGVLISQDNG	GTP-binding elongation factor- Q7VYR0
525	AVAYALWKLQDRGRM	GTP-binding elongation factor- Q7VYR0
526	DATLQSILGPALIAD	Bordetella resistance to killing- AAA51646
527	GSISVAGGSIDMDMG	Bordetella resistance to killing- AAA51646
528	GFPPPPPPLPGAPLA	Bordetella resistance to killing- AAA51646
529	HPPLDRVAAVHAGQD	Bordetella resistance to killing- AAA51646
530	KVTLREVALRAHGPQ	Bordetella resistance to killing- AAA51646
531	TGVYAYMPGSEITLQ	Bordetella resistance to killing- AAA51646
532	RGGRVEFQAPAPEAS	Bordetella resistance to killing- AAA51646
533	KTLTLQTLDGNGVFV	Bordetella resistance to killing- AAA51646
534	NTNVAAGQNDQLRVT	Bordetella resistance to killing- AAA51646
535	RADGQHRVLVRNAGG	Bordetella resistance to killing- AAA51646
536	ADSRGARLGLVHTQG	Bordetella resistance to killing- AAA51646
537	GNATFRLANVGKAVD	Bordetella resistance to killing- AAA51646
538	GVDAALGKGHNLYAS	Bordetella resistance to killing- AAA51646
539	EYAAGDRINIPWSFH	Bordetella resistance to killing- AAA51646
540	ARGWYGAGGRHPIHF	Vag8 protein (Autotr.) CAD12828 Q8VV95
541	ISAGAALMLGLLDVA	Vag8 protein (Autotr.) CAD12828 Q8VV95
542	AAAVTAAQRIDGGAA	Vag8 protein (Autotr.) CAD12828 Q8VV95
543	LGDVAIATTKASEHG	Vag8 protein (Autotr.) CAD12828 Q8VV95
544	AALLESGHLTVDGSV	Vag8 protein (Autotr.) CAD12828 Q8VV95
545	HGHGAAGLEVDGESN	Vag8 protein (Autotr.) CAD12828 Q8VV95
546	SLLNGARLSSDQPTA	Vag8 protein (Autotr.) CAD12828 Q8VV95
547	RLIDPRSVLNLDIKD	Vag8 protein (Autotr.) CAD12828 Q8VV95
548	AQLLGDIAPEAQQPD	Vag8 protein (Autotr.) CAD12828 Q8VV95
549	SPEQARVRVALADGG	Vag8 protein (Autotr.) CAD12828 Q8VV95
550	VKYNRFRHGFDIRTT	Vag8 protein (Autotr.) CAD12828 Q8VV95
551	LKRVDAKHRSHGLGA	Vag8 protein (Autotr.) CAD12828 Q8VV95
552	LRGGRRIDIDGGWYV	Vag8 protein (Autotr.) CAD12828 Q8VV95
553	PQASVAWFHAGGSRY	Vag8 protein (Autotr.) CAD12828 Q8VV95
554	ASNGLRVRADGAHSW	Vag8 protein (Autotr.) CAD12828 Q8VV95
555	LRAGAEAGRQMRLAN	Vag8 protein (Autotr.) CAD12828 Q8VV95
556	RLSSGVDARGDIVTL	putative autotransporter- AAC31207
557	PSAPPDSAEQPDAEP	putative autotransporter- AAC31207
558	PDAELEPDAAAQSDA	putative autotransporter- AAC31207
559	ANARVMAQVDGGEPV	putative autotransporter- AAC31207
560	VPIPAPSHPDAPIDV	putative autotransporter- AAC31207

561	IDSGAQWRGMTKTVN	putative autotransporter- AAC31207
562	RGKYRANGVGATLEA	putative autotransporter- AAC31207
563	KRFTLHDGWFVEPQS	putative autotransporter- AAC31207
564	VSLFHASGGTYRAAN	putative autotransporter- AAC31207
565	LSVKDEGGTSAVLRL	putative autotransporter- AAC31207
566	LAAGRRIDLGKDRVI	putative autotransporter- AAC31207
567	PYATLSWLQEFKGVT	putative autotransporter- AAC31207
568	MRCTRAIRQTARTGW	Pertussis toxin subunit 1 prec- O69258
569	TWLAILAVTAPVTSP	Pertussis toxin subunit 1 prec- O69258
570	WADDPPATVYRYDSR	Pertussis toxin subunit 1 prec- O69258
571	PEDVFQNGFTAWGNN	Pertussis toxin subunit 1 prec- O69258
572	NVLDHLTGRSCQVGS	Pertussis toxin subunit 1 prec- O69258
573	NSAFVSTSSSRRYTE	Pertussis toxin subunit 1 prec- O69258
574	ATDHYYSNVTATRLL	Pertussis toxin subunit 2 prec- P04978
575	STNSRLCAVFVRSGQ	Pertussis toxin subunit 2 prec- P04978
576	VIGACTSPYDGKYWS	Pertussis toxin subunit 2 prec- P04978
577	YSRLRKMLYLIYVAG	Pertussis toxin subunit 2 prec- P04978
578	SVRVHVSKEEQYYDY	Pertussis toxin subunit 2 prec- P04978
579	DATFETYALTGISIC	Pertussis toxin subunit 2 prec- P04978
580	AKLGAAASSPDAHVP	Pertussis toxin subunit 4 prec- P0A3R5
581	CFGKDLKRPGSSPME	Pertussis toxin subunit 4 prec- P0A3R5
582	MLRAVFMQQRPLRMF	Pertussis toxin subunit 4 prec- P0A3R5
583	GPKQLTFEGKPALEL	Pertussis toxin subunit 4 prec- P0A3R5
584	ELIRMVECSGKQDCP	Pertussis toxin subunit 4 prec- P0A3R5
585	PMHTIASILLSVLGI	Pertussis toxin subunit 5 prec- P04981
586	PEDLPPSRVVLRDTN	P.69A protein (pertactin)- CAA09473
587	TAVPASGAPAAVSVL	P.69A protein (pertactin)- CAA09473
588	ASELTLDGGHITGGR	P.69A protein (pertactin)- CAA09473
589	AGVAAMQGAVVHLQR	P.69A protein (pertactin)- CAA09473
590	TIRRGDAPAGGAVPG	P.69A protein (pertactin)- CAA09473
591	AVPGGAVPGGFGPGG	P.69A protein (pertactin)- CAA09473
592	PAPQPGPQPPQPPQP	P.69A protein (pertactin)- CAA09473
593	PEAPAPQPPAGRELS	P.69A protein (pertactin)- CAA09473
594	AANAAVNTGGVGLAS	P.69A protein (pertactin)- CAA09473
595	LWYAESNALSKRLGE	P.69A protein (pertactin)- CAA09473
596	RLNPDAGGAWGRGFA	P.69A protein (pertactin)- CAA09473
597	RQQLDNRAGRRFDQK	P.69A protein (pertactin)- CAA09473
598	ACTGLPLVTHAQGLV	filamentous hemagglutinin- AAA22974
599	QGQTQVLQGGNKVPV	filamentous hemagglutinin- AAA22974
600	NIADPNSGGVSHNKF	filamentous hemagglutinin- AAA22974
601	QFNVANPGVVFNNGL	filamentous hemagglutinin- AAA22974
602	DGVSRIGGALTKNPN	filamentous hemagglutinin- AAA22974
603	TRQASAILAEVTDTS	filamentous hemagglutinin- AAA22974
604	KQAVALGSASSNALS	filamentous hemagglutinin- AAA22974
605	RAGGALKAGKLSATG	filamentous hemagglutinin- AAA22974
606	LDVDGKQAVTLGSVA	filamentous hemagglutinin- AAA22974
607	DGALSVSAGGNLRAN	filamentous hemagglutinin- AAA22974

608	LVSSAQLEVRGQREV	filamentous hemagglutinin- AAA22974
609	LDDASSARGMTVVAA	filamentous hemagglutinin- AAA22974
610	VDQARSLADISLGAE	filamentous hemagglutinin- AAA22974
611	GATLGAVEAAGSIDV	filamentous hemagglutinin- AAA22974
612	GGSTVAANSLHANRD	filamentous hemagglutinin- AAA22974
613	RVSGKDAVRVTAATS	filamentous hemagglutinin- AAA22974
614	GGLHVSSGRQLDLGA	filamentous hemagglutinin- AAA22974
615	QARGALALDGGAGVA	filamentous hemagglutinin- AAA22974
616	RKDESVVSDAALVAD	filamentous hemagglutinin- AAA22974
617	GPIVVEAGELVSHAG	filamentous hemagglutinin- AAA22974
618	IGNGRNKENGASVTV	filamentous hemagglutinin- AAA22974
619	TTGNLVNKGYISAGK	filamentous hemagglutinin- AAA22974
620	GVLEVGGALTNEFLV	filamentous hemagglutinin- AAA22974
621	SDGTQRIEAQRIENR	filamentous hemagglutinin- AAA22974
622	TMAAGHDATLKAPHL	filamentous hemagglutinin- AAA22974
623	NTGQVVAGHDIHIIN	filamentous hemagglutinin- AAA22974
624	AKLENTGRVDARNDI	filamentous hemagglutinin- AAA22974
625	LDVADFTNTGSLYAE	filamentous hemagglutinin- AAA22974
626	DATLTLAQGTQRDLV	filamentous hemagglutinin- AAA22974
627	DQDHILPVAEGTLRV	filamentous hemagglutinin- AAA22974
628	YQAKPAPTAPPMPKA	filamentous hemagglutinin- AAA22974
629	ELDLRGHTLESAEGR	filamentous hemagglutinin- AAA22974
630	IFGEYKKLQGEYEKA	filamentous hemagglutinin- AAA22974
631	MAVQAVEAYGEATRR	filamentous hemagglutinin- AAA22974
632	HDQLGQRYGKALGGM	filamentous hemagglutinin- AAA22974
633	AETKEVDGIIQEFAA	filamentous hemagglutinin- AAA22974
634	ETRIKFIDQSKFYGS	filamentous hemagglutinin- AAA22974
635	YFFEQIGYKPDRAAR	filamentous hemagglutinin- AAA22974
636	AGDNYFDTTLVREQV	filamentous hemagglutinin- AAA22974
637	RALGGYESRLPVRGV	filamentous hemagglutinin- AAA22974
638	LVAKLMDSAGTVGKA	filamentous hemagglutinin- AAA22974
639	GLKVGVAPTAQQLKQ	filamentous hemagglutinin- AAA22974
640	AGFSLGSESGLEAHA	filamentous hemagglutinin- AAA22974
641	RGMTAGAEVKVGYRA	filamentous hemagglutinin- AAA22974
642	HEQSSETEKSYRNAN	filamentous hemagglutinin- AAA22974
643	NFGGGSVEAGNVLDI	filamentous hemagglutinin- AAA22974
644	GADINRNRYGGAAKG	filamentous hemagglutinin- AAA22974
645	AGTEEALRMRAKKVE	filamentous hemagglutinin- AAA22974
646	SDGLTGHVKGDANLT	filamentous hemagglutinin- AAA22974
647	ATIADLSGKGNLKVD	filamentous hemagglutinin- AAA22974
648	AVNAQNLKDYRDKDG	filamentous hemagglutinin- AAA22974
649	SGGLNVGISSTTLAP	filamentous hemagglutinin- AAA22974
650	VGVAFGRVAGEDYQA	filamentous hemagglutinin- AAA22974
651	QRATIDVGQTKDPAR	filamentous hemagglutinin- AAA22974
652	KSLPGGKLPKPVTVK	filamentous hemagglutinin- AAA22974
653	TDENGKPQTYTINRR	filamentous hemagglutinin- AAA22974
654	DLMKLNGKVLSTKTT	filamentous hemagglutinin- AAA22974

655	GLEQTFRLRSRISAA	filamentous hemagglutinin- AAA22974
656	MQIPFQRALRLCLRA	Fim2 pilic subunit- CAD12823.1 Q8VVA0
657	LAAIASAAHADDGTI	Fim2 pilic subunit- CAD12823.1 Q8VVA0
658	TVSSATKAKGVEFRL	Serotype 3 fimbrial subunit-CAA35920 P17835
659	NLNGQHIRMGTDKTT	Serotype 3 fimbrial subunit-CAA35920 P17835
660	AAQTFTGKVTNGSKS	Serotype 3 fimbrial subunit-CAA35920 P17835
661	TLRYLASYVKKPKED	Serotype 3 fimbrial subunit-CAA35920 P17835
662	DAAQITSYVGFSVVY	Serotype 3 fimbrial subunit-CAA35920 P17835
663	RATPCRGAVRALALA	tracheal colon factor- CAA08832. O86135
664	PQGGGEQDAPEVPPV	tracheal colon factor- CAA08832. O86135
665	PAPPAGNGVYDPGTH	tracheal colon factor- CAA08832. O86135
666	LTTPASAAVSLASSS	tracheal colon factor- CAA08832. O86135
667	GVWQAEMNALSKRMG	tracheal colon factor- CAA08832. O86135
668	LRLTPVAGGVWGRAF	tracheal colon factor- CAA08832. O86135
669	RRQDVDNRVSREFRQ	tracheal colon factor- CAA08832. O86135
670	GVHAKSSDWGLQAGY	Bif. hemolysin-adenylate cyclaseprec- P15318
671	PVNPNLSKLFGRAPE	Bif. hemolysin-adenylate cyclaseprec- P15318
672	IARADNDVNSSLAHG	Bif. hemolysin-adenylate cyclaseprec- P15318
673	TAVDLTLSKERLDYL	Bif. hemolysin-adenylate cyclaseprec- P15318
674	QAGLVTGMADGVVAS	Bif. hemolysin-adenylate cyclaseprec- P15318
675	HAGYEQFEFRVKETS	Bif. hemolysin-adenylate cyclaseprec- P15318
676	DDAEPGVSGASAHWG	Bif. hemolysin-adenylate cyclaseprec- P15318
677	RALQGAQAVAAAQRL	Bif. hemolysin-adenylate cyclaseprec- P15318
678	HAIALMTQFGRAGST	Bif. hemolysin-adenylate cyclaseprec- P15318
679	TPQEAASLSAAVFGL	Bif. hemolysin-adenylate cyclaseprec- P15318
680	EASSAVAETVSGFFR	Bif. hemolysin-adenylate cyclaseprec- P15318
681	SSRWAGGFGVAGGAM	Bif. hemolysin-adenylate cyclaseprec- P15318
682	IASRKGERPALTFIT	Bif. hemolysin-adenylate cyclaseprec- P15318
683	LAAPGEEQRRRTKTG	Bif. hemolysin-adenylate cyclaseprec- P15318
684	SEFTTFVEIVGKQDR	Bif. hemolysin-adenylate cyclaseprec- P15318
685	RIRDGAADTTIDLAK	Bif. hemolysin-adenylate cyclaseprec- P15318
686	VSQLVDANGVLKHSI	Bif. hemolysin-adenylate cyclaseprec- P15318
687	LDVIGGDGDDVVLAN	Bif. hemolysin-adenylate cyclaseprec- P15318
688	SAMIHPGRIVAPHEY	Bif. hemolysin-adenylate cyclaseprec- P15318
689	FGIEADLSREWVRKA	Bif. hemolysin-adenylate cyclaseprec- P15318
690	ALGVDYYDNVRNVEN	Bif. hemolysin-adenylate cyclaseprec- P15318
691	IGTSMKDVLIGDAQA	Bif. hemolysin-adenylate cyclaseprec- P15318
692	TLMGQGGDDTVRGGD	Bif. hemolysin-adenylate cyclaseprec- P15318
693	DDLLFGGDGNDMLYG	Bif. hemolysin-adenylate cyclaseprec- P15318
694	GHDTIYESGGGHDTI	Bif. hemolysin-adenylate cyclaseprec- P15318
695	INAGADQLWFARQGN	Bif. hemolysin-adenylate cyclaseprec- P15318
696	LEIRILGTDDALTVH	Bif. hemolysin-adenylate cyclaseprec- P15318
697	WYRDADHRVEIIHAA	Bif. hemolysin-adenylate cyclaseprec- P15318
698	QAVDQAGIEKLVEAM	Bif. hemolysin-adenylate cyclaseprec- P15318
699	QYPDPGAAAAAPPAA	Bif. hemolysin-adenylate cyclaseprec- P15318
700	PSGDKFGGFGVNTFA	Out. M.porin protein prec-CAA41398.1 Q04064
701	GFKANSYMVGLSAPI	Out. M.porin protein prec-CAA41398.1 Q04064

702	GASNVFGSWQMVDPK	Out. M.porin protein prec-CAA41398.1 Q04064
703	TGGDEKMNVFSLGYT	Out. M.porin protein prec-CAA41398.1 Q04064
704	DLSKRTNLYAYGSYA	Out. M.porin protein prec-CAA41398.1 Q04064
705	NFAFLEDAKSTAVGV	Out. M.porin protein prec-CAA41398.1 Q04064
706	VQGSMARPDWHWANG	Out. M.porin protein OmpQ- CAD12825. Q8VV98
707	QASKAYVVTLGYRQD	Out. M.porin protein OmpQ- CAD12825. Q8VV98
708	SARTSLYAYGGYMKG	Out. M.porin protein OmpQ- CAD12825. Q8VV98
709	DPEDPFASDVGRATR	Out. M.porin protein OmpQ- CAD12825. Q8VV98
710	MTRALRNVAIIAHVD	GTP-binding elongation factor- Q7VYR0
711	GKTTLVDQLLRQSGT	GTP-binding elongation factor- Q7VYR0
712	GREGKFVTSRQIRDR	GTP-binding elongation factor- Q7VYR0
713	ERELKSNVALRVRDT	GTP-binding elongation factor- Q7VYR0
714	DDTVFEVSGRGELHL	GTP-binding elongation factor- Q7VYR0
715	ILLENMRREGYELAV	GTP-binding elongation factor- Q7VYR0
716	RPRVVFKEIDGVKCE	GTP-binding elongation factor- Q7VYR0
717	FEALTVDVEDAHQGG	GTP-binding elongation factor- Q7VYR0
718	NPMSKPAIGVRVSGA	Bordetella resistance to killing- AAA51646
719	RALTLAGSTIDATEG	Bordetella resistance to killing- AAA51646
720	IPAVVRRGGTLELDG	Bordetella resistance to killing- AAA51646
721	TVAGGEGMEPMTVSD	Bordetella resistance to killing- AAA51646
722	GSRLSVRGGVLGGEA	Bordetella resistance to killing- AAA51646
723	GVGLVRAAQGGQASI	Bordetella resistance to killing- AAA51646
724	STVSVRLTDGATAQG	Bordetella resistance to killing- AAA51646
725	NGVFLQQHSTIPVAV	Bordetella resistance to killing- AAA51646
726	LESGALARGDIVADG	Bordetella resistance to killing- AAA51646
727	KPLDAGISLSVASGA	Bordetella resistance to killing- AAA51646
728	WHGATQVLQSATLGK	Bordetella resistance to killing- AAA51646
729	GTWVVNADSRVQDMS	Bordetella resistance to killing- AAA51646
730	PNDWFAEPQAEVMLW	Bordetella resistance to killing- AAA51646
731	TSGKRYRASNGLRVK	Bordetella resistance to killing- AAA51646
732	DANTATLGRLGLRFG	Bordetella resistance to killing- AAA51646
733	RIALAGGNIVQPYAR	Bordetella resistance to killing- AAA51646
734	GWTQEFKSTGDVRTN	Bordetella resistance to killing- AAA51646
735	IGHAGAGRHGRVELG	Bordetella resistance to killing- AAA51646
736	EDAGTHVSMNGGALS	Vag8 protein (Autotr.) CAD12828 Q8VV95
737	SGANSPAAWLLAGGS	Vag8 protein (Autotr.) CAD12828 Q8VV95
738	QFRDTVLRTVGEASH	Vag8 protein (Autotr.) CAD12828 Q8VV95
739	VDVAAHSEVELAHAQ	Vag8 protein (Autotr.) CAD12828 Q8VV95
740	RADGQGAHGLVVTRS	Vag8 protein (Autotr.) CAD12828 Q8VV95
741	AMVRAGSLVESTGDG	Vag8 protein (Autotr.) CAD12828 Q8VV95
742	HSIGSPGGFWARGLS	Vag8 protein (Autotr.) CAD12828 Q8VV95
743	RQRLDTGYGPWQKQT	Vag8 protein (Autotr.) CAD12828 Q8VV95
744	SGIELGLDRRVAGGA	Vag8 protein (Autotr.) CAD12828 Q8VV95
745	TAWSVGMLAGYSETR	Vag8 protein (Autotr.) CAD12828 Q8VV95
746	DGGAYRAGHVHSAHV	Vag8 protein (Autotr.) CAD12828 Q8VV95
747	AYVSYLNDSGSYVDG	Vag8 protein (Autotr.) CAD12828 Q8VV95
748	PRASLRNTDVHGEVA	putative autotransporter- AAC31207

749	IALGFNGEANISGGS	putative autotransporter- AAC31207
750	SVEDGAVLTTLTPDA	putative autotransporter- AAC31207
751	EYYYDYALSMEHLPA	putative autotransporter- AAC31207
752	APLTPVRVTLSDGAR	putative autotransporter- AAC31207
753	SGETLIAHGGLLPMT	putative autotransporter- AAC31207
754	LDNKAGRRFDQKVYG	putative autotransporter- AAC31207
755	ELGADHAIAGQQGRW	putative autotransporter- AAC31207
756	VGGLLGYTRARRSFI	putative autotransporter- AAC31207
757	DGAGHTDSAHIGAYA	putative autotransporter- AAC31207
758	YVADNGFYFDSTLRA	putative autotransporter- AAC31207
759	RFENDFTVTATDAVS	putative autotransporter- AAC31207
760	VIPPQEQITQHGGPY	Pertussis toxin subunit 2 prec- P04978
761	RCANKTRALTVAELR	Pertussis toxin subunit 2 prec- P04978
762	SGDLQEYLRHVTRGW	Pertussis toxin subunit 2 prec- P04978
763	IFALYDGTYLGGEYG	Pertussis toxin subunit 2 prec- P04978
764	VIKDGTPGGAFDLKT	Pertussis toxin subunit 2 prec- P04978
765	FCIMTTRNTGQPATD	Pertussis toxin subunit 2 prec- P04978
766	YYDYEDATFQTYALT	Pertussis toxin subunit 3 prec- P04979
767	MLRRFPTRTTAPGQG	Pertussis toxin subunit 4 prec- P0A3R5
768	ARRSRVRALAWLLAS	Pertussis toxin subunit 4 prec- P0A3R5
769	AMTHLSPALADVPYV	Pertussis toxin subunit 4 prec- P0A3R5
770	VKTNMVVTSVAMKPY	Pertussis toxin subunit 4 prec- P0A3R5
771	VTPTRMLVCGIAAKL	Pertussis toxin subunit 4 prec- P0A3R5
772	LSDDGIRRFLGTVTV	P.69A protein (pertactin)- CAA09473
773	AGKLVADHATLANVG	P.69A protein (pertactin)- CAA09473
774	TWDDDGIALYVAGEQ	P.69A protein (pertactin)- CAA09473
775	QASIADSTLQGAGGV	P.69A protein (pertactin)- CAA09473
776	IERGANVTVQRSAIV	P.69A protein (pertactin)- CAA09473
777	GGLHIGALQSLQPED	P.69A protein (pertactin)- CAA09473
778	RMNVFADLGLSDKLV	P.69A protein (pertactin)- CAA09473
779	MQDASGQHRLWVRNS	P.69A protein (pertactin)- CAA09473
780	SEPASANTLLLVQTP	P.69A protein (pertactin)- CAA09473
781	GSAATFTLANKDGKV	P.69A protein (pertactin)- CAA09473
782	IGTYRYRLAANGNGQ	P.69A protein (pertactin)- CAA09473
783	SLVGAKAPPAPKPAP	P.69A protein (pertactin)- CAA09473
784	ELGLGMAAALGRGHS	P.69A protein (pertactin)- CAA09473
785	YASYEYSKGPKLAMP	P.69A protein (pertactin)- CAA09473
786	MNTNLYRLVFSHVRG	filamentous hemagglutinin- AAA22974
787	LVPVSEHCTVGNTFC	filamentous hemagglutinin- AAA22974
788	RTRGQARSGARATSL	filamentous hemagglutinin- AAA22974
789	VAPNALAWALMLACT	filamentous hemagglutinin- AAA22974
790	SAGKLASGGGAVNVA	filamentous hemagglutinin- AAA22974
791	GGAVKIASASSVGNL	filamentous hemagglutinin- AAA22974
792	VQGGGKVQATLLNAG	filamentous hemagglutinin- AAA22974
793	TLLVSGRQAVQLGAA	filamentous hemagglutinin- AAA22974
794	SRQALSVNAGGALKA	filamentous hemagglutinin- AAA22974
795	KLSATRRVDVDGKQA	filamentous hemagglutinin- AAA22974

796	LAAKGELTVSAARAA	filamentous hemagglutinin- AAA22974
797	VAELKSLDNISVTGG	filamentous hemagglutinin- AAA22974
798	RVSVQSVNSASRVAI	filamentous hemagglutinin- AAA22974
799	AHGALDVGKVSAKSG	filamentous hemagglutinin- AAA22974
800	GLEGWGAVGADSLGS	filamentous hemagglutinin- AAA22974
801	GAISVSGRDAVRVDQ	filamentous hemagglutinin- AAA22974
802	LRNDALTENGTVTIS	filamentous hemagglutinin- AAA22974
803	DSAVLEHSTIESKIS	filamentous hemagglutinin- AAA22974
804	SVLAAKGDKGKPAVS	filamentous hemagglutinin- AAA22974
805	KVAKKLFLNGTLRAV	filamentous hemagglutinin- AAA22974
806	DNNETMSGRQIDVVD	filamentous hemagglutinin- AAA22974
807	RPQITDAVTGEARKD	filamentous hemagglutinin- AAA22974
808	EAATIVAASVSNPGT	filamentous hemagglutinin- AAA22974
809	TAGKDITVTSRGGFD	filamentous hemagglutinin- AAA22974
810	EGKMESNKDIVIKTE	filamentous hemagglutinin- AAA22974
811	FSNGRVLDAKHDLTV	filamentous hemagglutinin- AAA22974
812	ASGQADNRGSLKAGH	filamentous hemagglutinin- AAA22974
813	FTVQAQRIDNSGTMA	filamentous hemagglutinin- AAA22974
814	IQAGGHGHIGGDVDN	filamentous hemagglutinin- AAA22974
815	SVVRTVSAMEYFKTP	filamentous hemagglutinin- AAA22974
816	PVSLTALDNRAGLSP	filamentous hemagglutinin- AAA22974
817	TWNFQSTYELLDYLL	filamentous hemagglutinin- AAA22974
818	QNRYEYIWGLYPTYT	filamentous hemagglutinin- AAA22974
819	WSVNTLKNLDLGYQA	filamentous hemagglutinin- AAA22974
820	RGLGSADALASLASL	filamentous hemagglutinin- AAA22974
821	AAQGLEVSGRRNAQV	filamentous hemagglutinin- AAA22974
822	DAGLAGPSAVAAPAV	filamentous hemagglutinin- AAA22974
823	AADVGVEPVTGDQVD	filamentous hemagglutinin- AAA22974
824	PVVAVGLEQPVATVR	filamentous hemagglutinin- AAA22974
825	APPAVALPRPLFETR	filamentous hemagglutinin- AAA22974
826	PLGSLFAILSSTTET	filamentous hemagglutinin- AAA22974
827	QSAHANHYGTRIEAG	filamentous hemagglutinin- AAA22974
828	LEGKMQNLEIEGGSV	filamentous hemagglutinin- AAA22974
829	AAHTDLSVARDARFK	filamentous hemagglutinin- AAA22974
830	AADFAHAEHEKDVRQ	filamentous hemagglutinin- AAA22974
831	SLGAKVGAGGYEAGF	filamentous hemagglutinin- AAA22974
832	ANVSIDAGKDLNLSG	filamentous hemagglutinin- AAA22974
833	RVRGKHVVLDVEGDI	filamentous hemagglutinin- AAA22974
834	ATSKQDERNYNSSGG	filamentous hemagglutinin- AAA22974
835	WDASAGVAIQNRTLV	filamentous hemagglutinin- AAA22974
836	PVGSAGFNFNTEHDN	filamentous hemagglutinin- AAA22974
837	RLTNDGAAGVVASDG	filamentous hemagglutinin- AAA22974
838	AQPLPPVKPQKATPG	filamentous hemagglutinin- AAA22974
839	VAEVGKATVTTVQVQ	filamentous hemagglutinin- AAA22974
840	APPKPAPVAKQPAPA	filamentous hemagglutinin- AAA22974
841	KPKPKPKPKAERPKP	filamentous hemagglutinin- AAA22974
842	KTTPLSGRHVVQQQV	filamentous hemagglutinin- AAA22974

843	VLQRQASDINNTKSL	filamentous hemagglutinin- AAA22974
844	TGSISDQTCVIEEPS	Serotype 3 fimbrial subunit-CAA35920 P17835
845	LNHIKVVQLPKISKN	Serotype 3 fimbrial subunit-CAA35920 P17835
846	LRNDGDTAGATPFDI	Serotype 3 fimbrial subunit-CAA35920 P17835
847	LKECPQALGALKLYF	Serotype 3 fimbrial subunit-CAA35920 P17835
848	PGITTNYDTGDLIAY	Serotype 3 fimbrial subunit-CAA35920 P17835
849	QTYNASGNGNLSTVS	Serotype 3 fimbrial subunit-CAA35920 P17835
850	EGEPGSKSPADGGQD	tracheal colon factor- CAA08832. O86135
851	PPPPRDGGDADPQPP	tracheal colon factor- CAA08832. O86135
852	DDGNGEQQPPKGGGD	tracheal colon factor- CAA08832. O86135
853	GQRPPPAAGNGGNGG	tracheal colon factor- CAA08832. O86135
854	GNAQLPERGDDAGPK	tracheal colon factor- CAA08832. O86135
855	PEGEGGDEGPQPPQG	tracheal colon factor- CAA08832. O86135
856	TELAVGVASQLGKHG	tracheal colon factor- CAA08832. O86135
857	LFGSYEYAKGSRQTM	tracheal colon factor- CAA08832. O86135
858	MQQSHQAGYANAADR	Bif. hemolysin-adenylate cyclaseprec- P15318
859	SGIPAAVLDGIKAVA	Bif. hemolysin-adenylate cyclaseprec- P15318
860	EKNATLMFRLVNPHS	Bif. hemolysin-adenylate cyclaseprec- P15318
861	SLIAEGVATKGLGVH	Bif. hemolysin-adenylate cyclaseprec- P15318
862	AGKSLFDDGLGAAPG	Bif. hemolysin-adenylate cyclaseprec- P15318
863	PSGRSKFSPDVLETV	Bif. hemolysin-adenylate cyclaseprec- P15318
864	ASPGLRRPSLGAVER	Bif. hemolysin-adenylate cyclaseprec- P15318
865	DSGYDSLDGVGSRSF	Bif. hemolysin-adenylate cyclaseprec- P15318
866	LGEVSDMAAVEAAEL	Bif. hemolysin-adenylate cyclaseprec- P15318
867	MTRQVLHAGARQDDA	Bif. hemolysin-adenylate cyclaseprec- P15318
868	AYFEKNLQARHEQLA	Bif. hemolysin-adenylate cyclaseprec- P15318
869	SDGLRKMLADLQAGW	Bif. hemolysin-adenylate cyclaseprec- P15318
870	ASSVIGVQTTEISKS	Bif. hemolysin-adenylate cyclaseprec- P15318
871	LELAAITGNADNLKS	Bif. hemolysin-adenylate cyclaseprec- P15318
872	DVFVDRFVQGERVAG	Bif. hemolysin-adenylate cyclaseprec- P15318
873	PVVLDVAAGGIDIAS	Bif. hemolysin-adenylate cyclaseprec- P15318
874	ALNLFSVDHVKNIEN	Bif. hemolysin-adenylate cyclaseprec- P15318
875	HGSRLNDRIAGDDQD	Bif. hemolysin-adenylate cyclaseprec- P15318
876	ELWGHDGNDTIRGRG	Bif. hemolysin-adenylate cyclaseprec- P15318
877	DDILRGGLGLDTLYG	Bif. hemolysin-adenylate cyclaseprec- P15318
878	DGNDIFLQDDETVSD	Bif. hemolysin-adenylate cyclaseprec- P15318
879	IDGGAGLDTVDYSAM	Bif. hemolysin-adenylate cyclaseprec- P15318
880	GKGFASLMDEPETSN	Bif. hemolysin-adenylate cyclaseprec- P15318
881	LRNIENAVGSARDDV	Bif. hemolysin-adenylate cyclaseprec- P15318
882	IGDAGANVLNGLAGN	Bif. hemolysin-adenylate cyclaseprec- P15318
883	VLSGGAGDDVLLGDE	Bif. hemolysin-adenylate cyclaseprec- P15318
884	SDLLSGDAGNDDLFG	Bif. hemolysin-adenylate cyclaseprec- P15318
885	QGDDTYLFGVGYGHD	Bif. hemolysin-adenylate cyclaseprec- P15318
886	ADAVNRVGFATADNV	Out. M.porin protein prec-CAA41398.1 Q04064
887	AITTGLRYVNGPLNV	Out. M.porin protein prec-CAA41398.1 Q04064
888	LSYDQLNASNNQAQG	Out. M.porin protein prec-CAA41398.1 Q04064
889	VDATPRSYGLGGSYD	Out. M.porin protein prec-CAA41398.1 Q04064

890	EVVKLALAYARTTDG	Out. M.porin protein prec-CAA41398.1 Q04064
891	FGGQGYPVAVTLPSG	Out. M.porin protein prec-CAA41398.1 Q04064
892	GPLLAAFTWDKLNLH	Out. M.porin protein OmpQ- CAD12825. Q8VV98
893	TSATGGRSPQALQAG	Out. M.porin protein OmpQ- CAD12825. Q8VV98
894	TYDFEALKMALAWSR	Out. M.porin protein OmpQ- CAD12825. Q8VV98
895	RNGFVGLNGGGQIGL	Out. M.porin protein OmpQ- CAD12825. Q8VV98
896	PEPFAHGGAINAWLL	Out. M.porin protein OmpQ- CAD12825. Q8VV98
897	LEVPVHGNGAWLVQG	Out. M.porin protein OmpQ- CAD12825. Q8VV98
898	GRMRGNMDVAFRFGP	GTP-binding elongation factor- Q7VYR0
899	GEVQRGRINQVMKFS	GTP-binding elongation factor- Q7VYR0
900	LERVVVDEAEAGDIV	GTP-binding elongation factor- Q7VYR0
901	VNGIEDLHIGSTITD	GTP-binding elongation factor- Q7VYR0
902	STPEGLPVLRIDEPT	GTP-binding elongation factor- Q7VYR0
903	TMNFMVNTSPLAGRE	GTP-binding elongation factor- Q7VYR0
904	RSAWRLHALAAALAL	Bordetella resistance to killing- AAA51646
905	GMARLAPAAAQAPQP	Bordetella resistance to killing- AAA51646
906	VAGAPHAQDAGQEGE	Bordetella resistance to killing- AAA51646
907	DHRDNTLIAVFDDGV	Bordetella resistance to killing- AAA51646
908	INLDDDPDELGETAP	Bordetella resistance to killing- AAA51646
909	TLKDIHISVEHKNPM	Bordetella resistance to killing- AAA51646
910	GFEPQSGSGPASVDM	Bordetella resistance to killing- AAA51646
911	GGSITTTGNRAAGIA	Bordetella resistance to killing- AAA51646
912	THGSARLEGVAVRAE	Bordetella resistance to killing- AAA51646
913	SGSSAAQLANGTLVV	Bordetella resistance to killing- AAA51646
914	AGSLASAQSGAISVT	Bordetella resistance to killing- AAA51646
915	TPLKLMPGALASSTV	Bordetella resistance to killing- AAA51646
916	ASGGRWYAGGLLGYT	Bordetella resistance to killing- AAA51646
917	ADRTYPGDGGGKVKG	Bordetella resistance to killing- AAA51646
918	HVGGYAAYVGDGGYY	Bordetella resistance to killing- AAA51646
919	DTVLRLGRYDQQYNI	Bordetella resistance to killing- AAA51646
920	GTDGGRVTADYRTSG	Bordetella resistance to killing- AAA51646
921	AWSLEGGRRFELPND	Bordetella resistance to killing- AAA51646
922	NDVALETAGQQAPAV	Vag8 protein (Autotr.) CAD12828 Q8VV95
923	LWQGAQLNAQGLVVQ	Vag8 protein (Autotr.) CAD12828 Q8VV95
924	NGAGVSAIHAQDAGS	Vag8 protein (Autotr.) CAD12828 Q8VV95
925	TLSGSDITARGLEVA	Vag8 protein (Autotr.) CAD12828 Q8VV95
926	IYVQEGMQGTLTGTR	Vag8 protein (Autotr.) CAD12828 Q8VV95
927	TTQGDTAPALQVEDA	Vag8 protein (Autotr.) CAD12828 Q8VV95
928	ESGATKVPLIEDEQG	Vag8 protein (Autotr.) CAD12828 Q8VV95
929	TAFTLGNMGGRVDAG	Vag8 protein (Autotr.) CAD12828 Q8VV95
930	RQYELTASEAQADKA	Vag8 protein (Autotr.) CAD12828 Q8VV95
931	TWQLTPTNELSTTAT	Vag8 protein (Autotr.) CAD12828 Q8VV95
932	AVNAMAIAASQRIWQ	Vag8 protein (Autotr.) CAD12828 Q8VV95
933	EMDVLLRHMSGLHSI	Vag8 protein (Autotr.) CAD12828 Q8VV95
934	VVATGSGKVAIENAE	putative autotransporter- AAC31207
935	LGASGMYATFGAQVD	putative autotransporter- AAC31207
936	KGGRILAHNTNILGS	putative autotransporter- AAC31207

937	GYADGPYGGVVVTED	putative autotransporter- AAC31207
938	QVNLEGAKVSATGLG	putative autotransporter- AAC31207
939	AGLWLLGDKDTSPRA	putative autotransporter- AAC31207
940	GSQTKFTLANRGGVV	putative autotransporter- AAC31207
941	AGAFRYRLTPDNGVW	putative autotransporter- AAC31207
942	LERTSQLSAVANAAL	putative autotransporter- AAC31207
943	TGGVGAASSIWYAEG	putative autotransporter- AAC31207
944	ALSKRLGELRLDPGA	putative autotransporter- AAC31207
945	GFWGRTFAQKQQLDN	putative autotransporter- AAC31207
946	NARYVSQQTRANPNP	Pertussis toxin subunit 1 prec- O69258
947	TSRRSVASIVGTLVR	Pertussis toxin subunit 1 prec- O69258
948	APVIGACMARQAESS	Pertussis toxin subunit 1 prec- O69258
949	AMAAWSERAGEAMVL	Pertussis toxin subunit 1 prec- O69258
950	MPIDRKTLCHLLSVL	Pertussis toxin subunit 2 prec- P04978
951	LALLGSHVARASTPG	Pertussis toxin subunit 2 prec- P04978
952	GAGFIYRETFCITTI	Pertussis toxin subunit 3 prec- P04979
953	KTGQPAADHYYSKVT	Pertussis toxin subunit 3 prec- P04979
954	TRLLASTNSRLCAVF	Pertussis toxin subunit 3 prec- P04979
955	RDGQSVIGACASPYE	Pertussis toxin subunit 3 prec- P04979
956	RYRDMYDALRRLLYM	Pertussis toxin subunit 3 prec- P04979
957	YMSGLAVRVHVSKEE	Pertussis toxin subunit 3 prec- P04979
958	LSRIVKAAPLRRTTL	P.69A protein (pertactin)- CAA09473
959	MALGALGAAPAAHAD	P.69A protein (pertactin)- CAA09473
960	NNQSIVKTGERQHGI	P.69A protein (pertactin)- CAA09473
961	IQGSDPGGVRTASGT	P.69A protein (pertactin)- CAA09473
962	IKVSGRQAQGILLEN	P.69A protein (pertactin)- CAA09473
963	AAELQFRNGSVTSSG	P.69A protein (pertactin)- CAA09473
964	GGADAQGDIVATELP	P.69A protein (pertactin)- CAA09473
965	IPGTSIGPLDVALAS	P.69A protein (pertactin)- CAA09473
966	ARWTGATRAVDSLSI	P.69A protein (pertactin)- CAA09473
967	NATWVMTDNSNVGAL	P.69A protein (pertactin)- CAA09473
968	LASDGSVDFQQPAEA	P.69A protein (pertactin)- CAA09473
969	RFKVLTVNTLAGSGL	P.69A protein (pertactin)- CAA09473
970	RFTHADGWFLEPQAE	P.69A protein (pertactin)- CAA09473
971	AVFRAGGGAYRAANG	P.69A protein (pertactin)- CAA09473
972	RVRDEGGSSVLGRLG	P.69A protein (pertactin)- CAA09473
973	EVGKRIELAGGRQVQ	P.69A protein (pertactin)- CAA09473
974	YIKASVLQEFDGAGT	P.69A protein (pertactin)- CAA09473
975	HTNGIAHRTELRGTR	P.69A protein (pertactin)- CAA09473
976	VVAGANRYDHATRRA	filamentous hemagglutinin- AAA22974
977	PIAAGARGAAAGAYA	filamentous hemagglutinin- AAA22974
978	DGTAAGAMYGKHITL	filamentous hemagglutinin- AAA22974
979	SSDSGLGVRQLGSLS	filamentous hemagglutinin- AAA22974
980	PSAITVSSQGEIALG	filamentous hemagglutinin- AAA22974
981	ATVQRGPLSLKGAGV	filamentous hemagglutinin- AAA22974
982	DMRSRGAVTVSGGGA	filamentous hemagglutinin- AAA22974
983	NLGDVQSDGQVRATS	filamentous hemagglutinin- AAA22974

984	GAMTVRDVAAAADLA	filamentous hemagglutinin- AAA22974
985	QAGDALQAGFLKSAG	filamentous hemagglutinin- AAA22974
986	MTVNGRDAVRLDGAH	filamentous hemagglutinin- AAA22974
987	GGQLRVSSDGQAALG	filamentous hemagglutinin- AAA22974
988	AGALELSGQGVTVDR	filamentous hemagglutinin- AAA22974
989	SASRARIDSTGSVGI	filamentous hemagglutinin- AAA22974
990	ALKAGAVEAASPRRA	filamentous hemagglutinin- AAA22974
991	RALRQDFFTPGSVVV	filamentous hemagglutinin- AAA22974
992	AQGNVTVGRGDPHQG	filamentous hemagglutinin- AAA22974
993	LAQGDIIMDAKGGTL	filamentous hemagglutinin- AAA22974
994	VSADAIALAAQVTQR	filamentous hemagglutinin- AAA22974
995	GAANLTSRHDTRFSN	filamentous hemagglutinin- AAA22974
996	IRLMGPLQVNAGGPV	filamentous hemagglutinin- AAA22974
997	NTGNLKVREGVTVTA	filamentous hemagglutinin- AAA22974
998	SFDNETGAEVMAKSA	filamentous hemagglutinin- AAA22974
999	LTTSGAARNAGKMQV	filamentous hemagglutinin- AAA22974
1000	MHLDAPRIENTAKLS	filamentous hemagglutinin- AAA22974
1001	EVQRKGVQDVGGGEH	filamentous hemagglutinin- AAA22974
1002	RWSGIGYVNYWLRAG	filamentous hemagglutinin- AAA22974
1003	GKKAGTIAAPWYGGD	filamentous hemagglutinin- AAA22974
1004	TAEQSLIEVGKDLYL	filamentous hemagglutinin- AAA22974
1005	AGARKDEHRHLLNEG	filamentous hemagglutinin- AAA22974
1006	GAIHNGENAAQNRGR	filamentous hemagglutinin- AAA22974
1007	EGLKIGAHSATSVSG	filamentous hemagglutinin- AAA22974
1008	FDALRDVGLEKRLDI	filamentous hemagglutinin- AAA22974
1009	DALAAVLVNPHIFTR	filamentous hemagglutinin- AAA22974
1010	GAAQTSLADGAAGPA	filamentous hemagglutinin- AAA22974
1011	ARQARQAPETDGMVD	filamentous hemagglutinin- AAA22974
1012	GRDIGIEGGKLRGKD	filamentous hemagglutinin- AAA22974
1013	RLKADTVKVATSMRY	filamentous hemagglutinin- AAA22974
1014	DKGRLAARGDGALDA	filamentous hemagglutinin- AAA22974
1015	GGQLHIEAKRLETAG	filamentous hemagglutinin- AAA22974
1016	TLKGGKVKLDVDDVK	filamentous hemagglutinin- AAA22974
1017	GGVYEAGSSYENKSS	filamentous hemagglutinin- AAA22974
1018	NLSIEATEGDATLVG	filamentous hemagglutinin- AAA22974
1019	KFGGGDQVSLKAAKS	filamentous hemagglutinin- AAA22974
1020	NLMAAESTFESYSES	filamentous hemagglutinin- AAA22974
1021	NFHASADANLGANAV	filamentous hemagglutinin- AAA22974
1022	GAVGLGLTAGMGTSH	filamentous hemagglutinin- AAA22974
1023	ITNETGKTYAGTSVD	filamentous hemagglutinin- AAA22974
1024	TAQPLPPRPVAAQVV	filamentous hemagglutinin- AAA22974
1025	VTPPKVEVAKVEVVP	filamentous hemagglutinin- AAA22974
1026	PKVETAQPLPPRPVV	filamentous hemagglutinin- AAA22974
1027	EKVTTPAVQPQLAKV	filamentous hemagglutinin- AAA22974
1028	TVQPVKPETTKPLPK	filamentous hemagglutinin- AAA22974
1029	LPVAKVTKAPPPVVE	filamentous hemagglutinin- AAA22974
1030	EAQGVQVRISNLNDS	Fim2 pilic subunit- CAD12823.1 Q8VVA0

1031	ITMGANEATQQAAGF	Fim2 pilic subunit- CAD12823.1 Q8VVA0
1032	PEVQTGGTSKTVTMR	Fim2 pilic subunit- CAD12823.1 Q8VVA0
1033	LASYVKKNGDVEASA	Fim2 pilic subunit- CAD12823.1 Q8VVA0
1034	MSKFSYPALRAALIL	Serotype 3 fimbrial subunit-CAA35920 P17835
1035	ASPVLPALANDGTIV	Serotype 3 fimbrial subunit-CAA35920 P17835
1036	HNPGVGGGTHENGLP	tracheal colon factor- CAA08832. O86135
1037	IGKVGGSAPGPDTST	tracheal colon factor- CAA08832. O86135
1038	SGPDAGMASGAGSTS	tracheal colon factor- CAA08832. O86135
1039	GASGGAGKDAMPPSE	tracheal colon factor- CAA08832. O86135
1040	ERPDSGMSDSGRGGE	tracheal colon factor- CAA08832. O86135
1041	SAGGLNPDGAGKPPR	tracheal colon factor- CAA08832. O86135
1042	KRFTWPGAWYVEPQL	tracheal colon factor- CAA08832. O86135
1043	VAAFHAQGADYTASN	tracheal colon factor- CAA08832. O86135
1044	LRIKDDGTNSMLGRL	tracheal colon factor- CAA08832. O86135
1045	LHVGRQFDLGDGRVV	tracheal colon factor- CAA08832. O86135
1046	PYMKLSWVQEFDGKG	tracheal colon factor- CAA08832. O86135
1047	VRTNDIRHKVRLDGG	tracheal colon factor- CAA08832. O86135
1048	ARRQFRYDGDMNIGV	Bif. hemolysin-adenylate cyclaseprec- P15318
1049	TDFELEVRNALNRRA	Bif. hemolysin-adenylate cyclaseprec- P15318
1050	AVGAQDVVQHGTEQN	Bif. hemolysin-adenylate cyclaseprec- P15318
1051	PFPEADEKIFVVSAT	Bif. hemolysin-adenylate cyclaseprec- P15318
1052	ESQMLTRGQLKEYIG	Bif. hemolysin-adenylate cyclaseprec- P15318
1053	QRGEGYVFYENRAYG	Bif. hemolysin-adenylate cyclaseprec- P15318
1054	QESSAYGYEGDALLA	Bif. hemolysin-adenylate cyclaseprec- P15318
1055	LYRDKTAAEGAVAGV	Bif. hemolysin-adenylate cyclaseprec- P15318
1056	AVLSTVGAAVSIAAA	Bif. hemolysin-adenylate cyclaseprec- P15318
1057	SVVGAPVAVVTSLLT	Bif. hemolysin-adenylate cyclaseprec- P15318
1058	ALNGILRGVQQPIIE	Bif. hemolysin-adenylate cyclaseprec- P15318
1059	LANDYARKIDELGGP	Bif. hemolysin-adenylate cyclaseprec- P15318
1060	NAHDNFLAGGSGDDR	Bif. hemolysin-adenylate cyclaseprec- P15318
1061	DGGAGNDTLVGGEGQ	Bif. hemolysin-adenylate cyclaseprec- P15318
1062	TVIGGAGDDVFLQDL	Bif. hemolysin-adenylate cyclaseprec- P15318
1063	VWSNQLDGGAGVDTV	Bif. hemolysin-adenylate cyclaseprec- P15318
1064	YNVHQPSEERLERMG	Bif. hemolysin-adenylate cyclaseprec- P15318
1065	TGIHADLQKGTVEKW	Bif. hemolysin-adenylate cyclaseprec- P15318
1066	RITGDAQANVLRGAG	Bif. hemolysin-adenylate cyclaseprec- P15318
1067	ADVLAGGEGDDVLLG	Bif. hemolysin-adenylate cyclaseprec- P15318
1068	DGDDQLSGDAGRDRL	Bif. hemolysin-adenylate cyclaseprec- P15318
1069	GEAGDDWFFQDAANA	Bif. hemolysin-adenylate cyclaseprec- P15318
1070	NLLDGGDGRDTVDFS	Bif. hemolysin-adenylate cyclaseprec- P15318
1071	PGRGLDAGAKGVFLS	Bif. hemolysin-adenylate cyclaseprec- P15318
1072	GNGNSAQDGRLFGRQ	Out. M.porin protein prec-CAA41398.1 Q04064
1073	TIGLQSESWGRLDFG	Out. M.porin protein prec-CAA41398.1 Q04064
1074	QTNIASKYFGSIDPF	Out. M.porin protein prec-CAA41398.1 Q04064
1075	AGFGQANIGMGMSAM	Out. M.porin protein prec-CAA41398.1 Q04064
1076	TVRYDNMVMYQTPSY	Out. M.porin protein prec-CAA41398.1 Q04064
1077	GFQFGIGYSFSANDK	Out. M.porin protein prec-CAA41398.1 Q04064

1078	WVGLGHAGVGELSLG	Out. M.porin protein OmpQ- CAD12825. Q8VV98
1079	QQSIGLQYGGQLEIA	Out. M.porin protein OmpQ- CAD12825. Q8VV98
1080	WRDMGMGALFKASDN	Out. M.porin protein OmpQ- CAD12825. Q8VV98
1081	RVNNLVNYLSPEFSG	Out. M.porin protein OmpQ- CAD12825. Q8VV98
1082	QWGVGYAFDVESGDT	Out. M.porin protein OmpQ- CAD12825. Q8VV98
1083	RFDRSPAFSTGLKYE	Out. M.porin protein OmpQ- CAD12825. Q8VV98
1084	PGARPDFVINATFEL	GTP-binding elongation factor- Q7VYR0
1085	DKLGATEEQLDFPVV	GTP-binding elongation factor- Q7VYR0
1086	ASGLSGYAGLTDDVR	GTP-binding elongation factor- Q7VYR0
1087	GDMRPLFEAIMKYVP	GTP-binding elongation factor- Q7VYR0
1088	RDDDANGPLQMQIIS	GTP-binding elongation factor- Q7VYR0
1089	DYNSYVGKIGVGRIN	GTP-binding elongation factor- Q7VYR0
1090	EPLYEGMIIGIHSRD	GTP-binding elongation factor- Q7VYR0
1091	DLVVNPIKGKQLTNV	GTP-binding elongation factor- Q7VYR0
1092	ASGTDEAVRLVPPIQ	GTP-binding elongation factor- Q7VYR0
1093	SLEYAVEFIDDDELV	GTP-binding elongation factor- Q7VYR0
1094	ITPKSIRLRKRYLQE	GTP-binding elongation factor- Q7VYR0
1095	MYLDRFRQCPSSLQI	Bordetella resistance to killing- AAA51646
1096	VQGDDGAGVVAGAGL	Bordetella resistance to killing- AAA51646
1097	DALPPGGTVRLDGTT	Bordetella resistance to killing- AAA51646
1098	STDGANTDAVLVRGD	Bordetella resistance to killing- AAA51646
1099	ARAEVVNTVLRTAKS	Bordetella resistance to killing- AAA51646
1100	AAGVSAQHGGRVTLR	Bordetella resistance to killing- AAA51646
1101	TRIETAGAGAEGISV	Bordetella resistance to killing- AAA51646
1102	YSLAEDPKTHVWSLQ	Bordetella resistance to killing- AAA51646
1103	AGQALSGAANAAVNA	Bordetella resistance to killing- AAA51646
1104	DLSSIALAESNALDK	Bordetella resistance to killing- AAA51646
1105	LGELRLRADAGGPWA	Bordetella resistance to killing- AAA51646
1106	TFSERQQISNRHARA	Bordetella resistance to killing- AAA51646
1107	DQTVSGLEIGLDRGW	Bordetella resistance to killing- AAA51646
1108	RTAEVRVTGGTIRTS	Vag8 protein (Autotr.) CAD12828 Q8VV95
1109	NQAQGLRVGTENAPD	Vag8 protein (Autotr.) CAD12828 Q8VV95
1110	TALGASVFLQNLIIE	Vag8 protein (Autotr.) CAD12828 Q8VV95
1111	SGTGALGVSVHEPQG	Vag8 protein (Autotr.) CAD12828 Q8VV95
1112	GGTRLSMSGTTVRTR	Vag8 protein (Autotr.) CAD12828 Q8VV95
1113	DDSFALQLSGPASAT	Vag8 protein (Autotr.) CAD12828 Q8VV95
1114	TDGAVHTVRLLDRGV	Vag8 protein (Autotr.) CAD12828 Q8VV95
1115	TVTGDSRVAEVKLEG	Vag8 protein (Autotr.) CAD12828 Q8VV95
1116	TLAFAPPAQPKGAFK	Vag8 protein (Autotr.) CAD12828 Q8VV95
1117	LVATQGISGTGTIVM	Vag8 protein (Autotr.) CAD12828 Q8VV95
1118	AHLPSGTADVLVAPQ	Vag8 protein (Autotr.) CAD12828 Q8VV95
1119	FGDRQVLVVNNTDDG	Vag8 protein (Autotr.) CAD12828 Q8VV95
1120	PYARLGWAQELGADN	Vag8 protein (Autotr.) CAD12828 Q8VV95
1121	VYTNGIRHVTRSRGG	Vag8 protein (Autotr.) CAD12828 Q8VV95
1122	AEARVGVGALLGKRH	Vag8 protein (Autotr.) CAD12828 Q8VV95
1123	LYADYEYAKGARFEA	Vag8 protein (Autotr.) CAD12828 Q8VV95
1124	MCDTCRDDDGTSPSI	putative autotransporter- AAC31207

1125	VQGGVVQGGMGANNV	putative autotransporter- AAC31207
1126	DGTWTVTGSSTVNSL	putative autotransporter- AAC31207
1127	LQAGKVAYATPAESD	putative autotransporter- AAC31207
1128	EFKHLRVKTLSGSGL	putative autotransporter- AAC31207
1129	EMNASADLSDGDLLV	putative autotransporter- AAC31207
1130	SDEASGQHKVLVRGA	putative autotransporter- AAC31207
1131	TEPTGVESLTLVELP	putative autotransporter- AAC31207
1132	GYGLRTDLSGGRAEL	putative autotransporter- AAC31207
1133	LGLAAALGRGHQLYT	putative autotransporter- AAC31207
1134	YEYAKGNKLTLPWTF	putative autotransporter- AAC31207
1135	RMQEAVEAERAGRGT	Pertussis toxin subunit 1 prec- O69258
1136	HFIGYIYEVRADNNF	Pertussis toxin subunit 1 prec- O69258
1137	GAASSYFEYVDTYGD	Pertussis toxin subunit 1 prec- O69258
1138	AGRILAGALATYQSE	Pertussis toxin subunit 1 prec- O69258
1139	LAHRRIPPENIRRVT	Pertussis toxin subunit 1 prec- O69258
1140	VYHNGITGETTTTEY	Pertussis toxin subunit 1 prec- O69258
1141	LHHILPILVLALLGM	Pertussis toxin subunit 3 prec- P04979
1142	TAQAVAPGIVIPPKA	Pertussis toxin subunit 3 prec- P04979
1143	FTQQGGAYGRCPNGT	Pertussis toxin subunit 3 prec- P04979
1144	ALTVAELRGNAELQT	Pertussis toxin subunit 3 prec- P04979
1145	LRQITPGWSIYGLYD	Pertussis toxin subunit 3 prec- P04979
1146	TYLGQAYGGIIKDAP	Pertussis toxin subunit 3 prec- P04979
1147	VAGLPTHLYKNFTVQ	Pertussis toxin subunit 5 prec- P04981
1148	LALKLKGKNQEFCLT	Pertussis toxin subunit 5 prec- P04981
1149	FMSGRSLVRACLSDA	Pertussis toxin subunit 5 prec- P04981
1150	HEHDTWFDTMLGFAI	Pertussis toxin subunit 5 prec- P04981
1151	AYALKSRIALTVEDS	Pertussis toxin subunit 5 prec- P04981
1152	YPGTPGDLLELQICP	Pertussis toxin subunit 5 prec- P04981
1153	DGWYGVDVSGSSVEL	P.69A protein (pertactin)- CAA09473
1154	QSIVEAPELGAAIRV	P.69A protein (pertactin)- CAA09473
1155	RGARVTVSGGSLSAP	P.69A protein (pertactin)- CAA09473
1156	GNVIETGGARRFAPQ	P.69A protein (pertactin)- CAA09473
1157	APLSITLQAGAHAQG	P.69A protein (pertactin)- CAA09473
1158	ALLYRVLPEPVKLTL	P.69A protein (pertactin)- CAA09473
1159	LGADHAVAVAGGRWH	P.69A protein (pertactin)- CAA09473
1160	GGLAGYTRGDRGFTG	P.69A protein (pertactin)- CAA09473
1161	GGGHTDSVHVGGYAT	P.69A protein (pertactin)- CAA09473
1162	IADSGFYLDATLRAS	P.69A protein (pertactin)- CAA09473
1163	LENDFKVAGSDGYAV	P.69A protein (pertactin)- CAA09473
1164	GKYRTHGVGASLEAG	P.69A protein (pertactin)- CAA09473
1165	GTLEVYGKGADLIIA	filamentous hemagglutinin- AAA22974
1166	PNGISVNGLSTLNAS	filamentous hemagglutinin- AAA22974
1167	LTLTTGRPSVNGGRI	filamentous hemagglutinin- AAA22974
1168	LDVQQGTVTIERGGV	filamentous hemagglutinin- AAA22974
1169	ATGLGYFDVVARLVK	filamentous hemagglutinin- AAA22974
1170	QGAVSSKQGKPLADI	filamentous hemagglutinin- AAA22974
1171	RNLQSKGAIGVQGGE	filamentous hemagglutinin- AAA22974

1172	VSVANANSDAELRVR	filamentous hemagglutinin- AAA22974
1173	RGQVDLHDLSAARGA	filamentous hemagglutinin- AAA22974
1174	ISGEGRVNIGRARSD	filamentous hemagglutinin- AAA22974
1175	DVKVSAHGALSIDSM	filamentous hemagglutinin- AAA22974
1176	ALGAIGVQAGGSVSA	filamentous hemagglutinin- AAA22974
1177	ASGTLHVQGGEHLDL	filamentous hemagglutinin- AAA22974
1178	TLAAVGAVDVNGTGD	filamentous hemagglutinin- AAA22974
1179	RVAKLVSDAGADLQA	filamentous hemagglutinin- AAA22974
1180	RSMTLGIVDTTGDLQ	filamentous hemagglutinin- AAA22974
1181	RAQQKLELGSVKSDG	filamentous hemagglutinin- AAA22974
1182	LQAAAGGALSLAAAE	filamentous hemagglutinin- AAA22974
1183	QAPAGTAGALVVKAA	filamentous hemagglutinin- AAA22974
1184	AIVHDGVMATKGEMQ	filamentous hemagglutinin- AAA22974
1185	AGKGGGSPTVTAGAK	filamentous hemagglutinin- AAA22974
1186	TTSANKLSVDVASWD	filamentous hemagglutinin- AAA22974
1187	AGSLDIKKGGAQVTV	filamentous hemagglutinin- AAA22974
1188	GRYAEHGEVSIQGDY	filamentous hemagglutinin- AAA22974
1189	TTEIETGNPGSLIAE	filamentous hemagglutinin- AAA22974
1190	QENIDNKQAIVVGKD	filamentous hemagglutinin- AAA22974
1191	TLSSAHGNVANEANA	filamentous hemagglutinin- AAA22974
1192	LWAAGELTVKAQNIT	filamentous hemagglutinin- AAA22974
1193	KRAALIEAGGNARLT	filamentous hemagglutinin- AAA22974
1194	AVALLNKLGRIRAGE	filamentous hemagglutinin- AAA22974
1195	YAKQADQATIDAETD	filamentous hemagglutinin- AAA22974
1196	VAQRYKSQIDAVRLQ	filamentous hemagglutinin- AAA22974
1197	IQPGRVTLAKALSAA	filamentous hemagglutinin- AAA22974
1198	GADWRALGHSQLMQR	filamentous hemagglutinin- AAA22974
1199	KDFKAGKRGAEIAFY	filamentous hemagglutinin- AAA22974
1200	KEQTVLAAGAGLTLS	filamentous hemagglutinin- AAA22974
1201	VWYVDTVIDGQKVLA	filamentous hemagglutinin- AAA22974
1202	RLYLTEATRQGITDQ	filamentous hemagglutinin- AAA22974
1203	AGGGALIASGGDVTV	filamentous hemagglutinin- AAA22974
1204	TDGHDVSSVNGLIQG	filamentous hemagglutinin- AAA22974
1205	SVKVDAGKGKVVVAD	filamentous hemagglutinin- AAA22974
1206	KGAGGGIEADDEVDV	filamentous hemagglutinin- AAA22974
1207	SEQTSQSSGWSVEVA	filamentous hemagglutinin- AAA22974
1208	TASARSSLLTAATRL	filamentous hemagglutinin- AAA22974
1209	DSVAQNVEDGREIRG	filamentous hemagglutinin- AAA22974
1210	LMAAQVAAEATQLVT	filamentous hemagglutinin- AAA22974
1211	DTAAVALSAGISADF	filamentous hemagglutinin- AAA22974
1212	SSHSRSTSQNTQYLG	filamentous hemagglutinin- AAA22974
1213	GVKGTLNQDAAQATV	filamentous hemagglutinin- AAA22974
1214	QRNKHWAGGGSEFSV	filamentous hemagglutinin- AAA22974
1215	GKSLKKKNQVRPVET	filamentous hemagglutinin- AAA22974
1216	TPDVVDGPPSRPTTP	filamentous hemagglutinin- AAA22974
1217	ASPQPIRATVEVSSP	filamentous hemagglutinin- AAA22974
1218	PVSVATVEVVPRPKV	filamentous hemagglutinin- AAA22974

1219	ITDTTCVIEDPSGPN	Fim2 pilic subunit- CAD12823.1 Q8VVA0
1220	TKVVQLPKISKNALK	Fim2 pilic subunit- CAD12823.1 Q8VVA0
1221	NGDQAGRTPFIIKLK	Fim2 pilic subunit- CAD12823.1 Q8VVA0
1222	CPSSLGNGVKAYFEP	Fim2 pilic subunit- CAD12823.1 Q8VVA0
1223	PTTDYSTGDLRAYKM	Fim2 pilic subunit- CAD12823.1 Q8VVA0
1224	YATNPQTQLSNITAA	Fim2 pilic subunit- CAD12823.1 Q8VVA0
1225	MWTLSPPSAWALKLP	tracheal colon factor- CAA08832. O86135
1226	LLTDDELKLVLPTGM	tracheal colon factor- CAA08832. O86135
1227	LEDFKRSLQESAPSA	tracheal colon factor- CAA08832. O86135
1228	ATPPSSSPPVAKPGP	tracheal colon factor- CAA08832. O86135
1229	SVAEAPSGSGHKDNP	tracheal colon factor- CAA08832. O86135
1230	PPVVGVGPGMAESSG	tracheal colon factor- CAA08832. O86135
1231	ELGADTALPVADGRW	tracheal colon factor- CAA08832. O86135
1232	VGAVAGYTNGRIKFD	tracheal colon factor- CAA08832. O86135
1233	GGTGDDDSVHVGAYA	tracheal colon factor- CAA08832. O86135
1234	YIEDGGFYMDGIVRV	tracheal colon factor- CAA08832. O86135
1235	RIRHAFKVDDAKGRR	tracheal colon factor- CAA08832. O86135
1236	RGQYRGNGVGASLEL	tracheal colon factor- CAA08832. O86135
1237	VQYRRKGGDDFEAVK	Bif. hemolysin-adenylate cyclaseprec- P15318
1238	IGNAAGIPLTADIDM	Bif. hemolysin-adenylate cyclaseprec- P15318
1239	AIMPHLSNFRDSARS	Bif. hemolysin-adenylate cyclaseprec- P15318
1240	VTSGDSVTDYLARTR	Bif. hemolysin-adenylate cyclaseprec- P15318
1241	AASEATGGLDRERID	Bif. hemolysin-adenylate cyclaseprec- P15318
1242	LWKIARAGARSAVGT	Bif. hemolysin-adenylate cyclaseprec- P15318
1243	IAAAVGAGMSLTDDA	Bif. hemolysin-adenylate cyclaseprec- P15318
1244	AGQKAAAGAEIALQL	Bif. hemolysin-adenylate cyclaseprec- P15318
1245	GGTVELASSIALALA	Bif. hemolysin-adenylate cyclaseprec- P15318
1246	ARGVTSGLQVAGASA	Bif. hemolysin-adenylate cyclaseprec- P15318
1247	AAAGALAAALSPMEI	Bif. hemolysin-adenylate cyclaseprec- P15318
1248	GLVQQSHYADQLDKL	Bif. hemolysin-adenylate cyclaseprec- P15318
1249	YDGGAGTNTVSYAAL	Bif. hemolysin-adenylate cyclaseprec- P15318
1250	RQDSITVSADGERFN	Bif. hemolysin-adenylate cyclaseprec- P15318
1251	RKQLNNANVYREGVA	Bif. hemolysin-adenylate cyclaseprec- P15318
1252	QTTAYGKRTENVQYR	Bif. hemolysin-adenylate cyclaseprec- P15318
1253	VELARVGQLVEVDTL	Bif. hemolysin-adenylate cyclaseprec- P15318
1254	HVQHIIGGAGNDSIT	Bif. hemolysin-adenylate cyclaseprec- P15318
1255	TLYGGLGDDTLEGGA	Bif. hemolysin-adenylate cyclaseprec- P15318
1256	NDWFGQTQAREHDVL	Bif. hemolysin-adenylate cyclaseprec- P15318
1257	GGDGVDTVDYSQTGA	Bif. hemolysin-adenylate cyclaseprec- P15318
1258	AGIAAGRIGLGILAD	Bif. hemolysin-adenylate cyclaseprec- P15318
1259	GAGRVDKLGEAGSSA	Bif. hemolysin-adenylate cyclaseprec- P15318
1260	DTVSGIENVVGTELA	Bif. hemolysin-adenylate cyclaseprec- P15318
1261	MKKTLLAAALLAGFA	Out. M.porin protein prec-CAA41398.1 Q04064
1262	AAQAETSVTLYGIID	Out. M.porin protein prec-CAA41398.1 Q04064
1263	GIGYNDVDFKVKGAN	Out. M.porin protein prec-CAA41398.1 Q04064
1264	DDSDFKYNHSRFGMI	Out. M.porin protein prec-CAA41398.1 Q04064
1265	GVQNGSRWGLRGTED	Out. M.porin protein prec-CAA41398.1 Q04064

1266	GDGLQAVFQLESGFN	Out. M.porin protein prec-CAA41398.1 Q04064
1267	AMAAGSSSVFWSVAP	Out. M.porin protein OmpQ- CAD12825. Q8VV98
1268	RAANQLELYGVVDVG	Out. M.porin protein OmpQ- CAD12825. Q8VV98
1269	ATTRVSGLGTRQQVL	Out. M.porin protein OmpQ- CAD12825. Q8VV98
1270	GGQTDNLWGLRGTEE	Out. M.porin protein OmpQ- CAD12825. Q8VV98
1271	DGGWRASFGLESGFD	Out. M.porin protein OmpQ- CAD12825. Q8VV98
1272	ANGTRNDDARLFDYG	Out. M.porin protein OmpQ- CAD12825. Q8VV98
1273	SVAERVMDSNDIEKE	GTP-binding elongation factor- Q7VYR0
1274	GITILAKNCAVEYEG	GTP-binding elongation factor- Q7VYR0
1275	HINIVDTPGHADFGG	GTP-binding elongation factor- Q7VYR0
1276	VERVLSMVDGVLLLV	GTP-binding elongation factor- Q7VYR0
1277	AVEGPMPQTIFVTRK	GTP-binding elongation factor- Q7VYR0
1278	LALGLKPIVVVNKID	GTP-binding elongation factor- Q7VYR0
1279	GRRKGDLQDMQPDGR	GTP-binding elongation factor- Q7VYR0
1280	RTRLEYIIPARGLIG	GTP-binding elongation factor- Q7VYR0
1281	QNEFLTLTRGTGLMS	GTP-binding elongation factor- Q7VYR0
1282	IFHEYAPLREGSIGE	GTP-binding elongation factor- Q7VYR0
1283	RNGVLISQDNGDAVA	GTP-binding elongation factor- Q7VYR0
1284	ALWKLQDRGRMFVSP	GTP-binding elongation factor- Q7VYR0
1285	QSILGPALIADGGSI	Bordetella resistance to killing- AAA51646
1286	VAGGSIDMDMGPGFP	Bordetella resistance to killing- AAA51646
1287	PPPPLPGAPLAAHPP	Bordetella resistance to killing- AAA51646
1288	DRVAAVHAGQDGKVT	Bordetella resistance to killing- AAA51646
1289	REVALRAHGPQATGV	Bordetella resistance to killing- AAA51646
1290	AYMPGSEITLQGGTV	Bordetella resistance to killing- AAA51646
1291	VEFQAPAPEASYKTL	Bordetella resistance to killing- AAA51646
1292	LQTLDGNGVFVLNTN	Bordetella resistance to killing- AAA51646
1293	AAGQNDQLRVTGRAD	Bordetella resistance to killing- AAA51646
1294	QHRVLVRNAGGEADS	Bordetella resistance to killing- AAA51646
1295	GARLGLVHTQGQGNA	Bordetella resistance to killing- AAA51646
1296	FRLANVGKAVDLGTW	Bordetella resistance to killing- AAA51646
1297	ALGKGHNLYASYEYA	Bordetella resistance to killing- AAA51646
1298	GDRINIPWSFHAGYR	Bordetella resistance to killing- AAA51646
1299	YGAGGRHPIHFQISA	Vag8 protein (Autotr.) CAD12828 Q8VV95
1300	AALMLGLLDVAGAAA	Vag8 protein (Autotr.) CAD12828 Q8VV95
1301	TAAQRIDGGAAFLGD	Vag8 protein (Autotr.) CAD12828 Q8VV95
1302	AIATTKASEHGINVT	Vag8 protein (Autotr.) CAD12828 Q8VV95
1303	ESGHLTVDGSVVHGH	Vag8 protein (Autotr.) CAD12828 Q8VV95
1304	AAGLEVDGESNVSLL	Vag8 protein (Autotr.) CAD12828 Q8VV95
1305	GARLSSDQPTAIRLI	Vag8 protein (Autotr.) CAD12828 Q8VV95
1306	PRSVLNLDIKDRAQL	Vag8 protein (Autotr.) CAD12828 Q8VV95
1307	GDIAPEAQQPDGSPE	Vag8 protein (Autotr.) CAD12828 Q8VV95
1308	ARVRVALADGGTWAG	Vag8 protein (Autotr.) CAD12828 Q8VV95
1309	RFRHGFDIRTTDLKR	Vag8 protein (Autotr.) CAD12828 Q8VV95
1310	DAKHRSHGLGALLRG	Vag8 protein (Autotr.) CAD12828 Q8VV95
1311	RRIDIDGGWYVEPQA	Vag8 protein (Autotr.) CAD12828 Q8VV95
1312	VAWFHAGGSRYEASN	Vag8 protein (Autotr.) CAD12828 Q8VV95

1313	LRVRADGAHSWVLRA	Vag8 protein (Autotr.) CAD12828 Q8VV95
1314	AEAGRQMRLANGNIV	Vag8 protein (Autotr.) CAD12828 Q8VV95
1315	GVDARGDIVTLPPSA	putative autotransporter- AAC31207
1316	PDSAEQPDAEPEPDA	putative autotransporter- AAC31207
1317	LEPDAAAQSDAKANA	putative autotransporter- AAC31207
1318	VMAQVDGGEPVAVPI	putative autotransporter- AAC31207
1319	APSHPDAPIDVFIDS	putative autotransporter- AAC31207
1320	AQWRGMTKTVNALRI	putative autotransporter- AAC31207
1321	RANGVGATLEAGKRF	putative autotransporter- AAC31207
1322	LHDGWFVEPQSEVSL	putative autotransporter- AAC31207
1323	HASGGTYRAANNLSV	putative autotransporter- AAC31207
1324	DEGGTSAVLRLGLAA	putative autotransporter- AAC31207
1325	RRIDLGKDRVIQPYA	putative autotransporter- AAC31207
1326	LSWLQEFKGVTTVRT	putative autotransporter- AAC31207
1327	RAIRQTARTGWLTWL	Pertussis toxin subunit 1 prec- O69258
1328	ILAVTAPVTSPAWAD	Pertussis toxin subunit 1 prec- O69258
1329	PPATVYRYDSRPPED	Pertussis toxin subunit 1 prec- O69258
1330	FQNGFTAWGNNDNVL	Pertussis toxin subunit 1 prec- O69258
1331	HLTGRSCQVGSSNSA	Pertussis toxin subunit 1 prec- O69258
1332	VSTSSSRRYTEVYLE	Pertussis toxin subunit 1 prec- O69258
1333	YYSNVTATRLLSSTN	Pertussis toxin subunit 2 prec- P04978
1334	RLCAVFVRSGQPVIG	Pertussis toxin subunit 2 prec- P04978
1335	CTSPYDGKYWSMYSR	Pertussis toxin subunit 2 prec- P04978
1336	RKMLYLIYVAGISVR	Pertussis toxin subunit 2 prec- P04978
1337	HVSKEEQYYDYEDAT	Pertussis toxin subunit 2 prec- P04978
1338	ETYALTGISICNPGS	Pertussis toxin subunit 2 prec- P04978
1339	AAASSPDAHVPFCFG	Pertussis toxin subunit 4 prec- P0A3R5
1340	DLKRPGSSPMEVMLR	Pertussis toxin subunit 4 prec- P0A3R5
1341	VFMQQRPLRMFLGPK	Pertussis toxin subunit 4 prec- P0A3R5
1342	LTFEGKPALELIRMV	Pertussis toxin subunit 4 prec- P0A3R5
1343	MQRQAGLPLKANPMH	Pertussis toxin subunit 5 prec- P04981
1344	IASILLSVLGIYSPA	Pertussis toxin subunit 5 prec- P04981
1345	PPSRVVLRDTNVTAV	P.69A protein (pertactin)- CAA09473
1346	ASGAPAAVSVLGASE	P.69A protein (pertactin)- CAA09473
1347	TLDGGHITGGRAAGV	P.69A protein (pertactin)- CAA09473
1348	AMQGAVVHLQRATIR	P.69A protein (pertactin)- CAA09473
1349	GDAPAGGAVPGGAVP	P.69A protein (pertactin)- CAA09473
1350	GAVPGGFGPGGFGPV	P.69A protein (pertactin)- CAA09473
1351	PGPQPPQPPQPQPEA	P.69A protein (pertactin)- CAA09473
1352	APQPPAGRELSAAAN	P.69A protein (pertactin)- CAA09473
1353	AVNTGGVGLASTLWY	P.69A protein (pertactin)- CAA09473
1354	ESNALSKRLGELRLN	P.69A protein (pertactin)- CAA09473
1355	DAGGAWGRGFAQRQQ	P.69A protein (pertactin)- CAA09473
1356	DNRAGRRFDQKVAGF	P.69A protein (pertactin)- CAA09473
1357	LPLVTHAQGLVPQGQ	filamentous hemagglutinin- AAA22974
1358	QVLQGGNKVPVVNIA	filamentous hemagglutinin- AAA22974
1359	PNSGGVSHNKFQQFN	filamentous hemagglutinin- AAA22974

1360	ANPGVVFNNGLTDGV	filamentous hemagglutinin- AAA22974
1361	RIGGALTKNPNLTRQ	filamentous hemagglutinin- AAA22974
1362	SAILAEVTDTSPSRL	filamentous hemagglutinin- AAA22974
1363	ALGSASSNALSVRAG	filamentous hemagglutinin- AAA22974
1364	ALKAGKLSATGRLDV	filamentous hemagglutinin- AAA22974
1365	GKQAVTLGSVASDGA	filamentous hemagglutinin- AAA22974
1366	SVSAGGNLRANELVS	filamentous hemagglutinin- AAA22974
1367	AQLEVRGQREVALDD	filamentous hemagglutinin- AAA22974
1368	SSARGMTVVAAGALA	filamentous hemagglutinin- AAA22974
1369	RSLADISLGAEGGAT	filamentous hemagglutinin- AAA22974
1370	GAVEAAGSIDVRGGS	filamentous hemagglutinin- AAA22974
1371	VAANSLHANRDVRVS	filamentous hemagglutinin- AAA22974
1372	KDAVRVTAATSGGGL	filamentous hemagglutinin- AAA22974
1373	VSSGRQLDLGAVQAR	filamentous hemagglutinin- AAA22974
1374	ALALDGGAGVALQSA	filamentous hemagglutinin- AAA22974
1375	SVVSDAALVADGGPI	filamentous hemagglutinin- AAA22974
1376	VEAGELVSHAGGIGN	filamentous hemagglutinin- AAA22974
1377	RNKENGASVTVRTTG	filamentous hemagglutinin- AAA22974
1378	LVNKGYISAGKQGVL	filamentous hemagglutinin- AAA22974
1379	VGGALTNEFLVGSDG	filamentous hemagglutinin- AAA22974
1380	QRIEAQRIENRGTFQ	filamentous hemagglutinin- AAA22974
1381	GHDATLKAPHLRNTG	filamentous hemagglutinin- AAA22974
1382	VVAGHDIHIINSAKL	filamentous hemagglutinin- AAA22974
1383	NTGRVDARNDIALDV	filamentous hemagglutinin- AAA22974
1384	DFTNTGSLYAEHDAT	filamentous hemagglutinin- AAA22974
1385	TLAQGTQRDLVVDQD	filamentous hemagglutinin- AAA22974
1386	ILPVAEGTLRVKAKS	filamentous hemagglutinin- AAA22974
1387	PAPTAPPMPKAPELD	filamentous hemagglutinin- AAA22974
1388	RGHTLESAEGRKIFG	filamentous hemagglutinin- AAA22974
1389	YKKLQGEYEKAKMAV	filamentous hemagglutinin- AAA22974
1390	AVEAYGEATRRVHDQ	filamentous hemagglutinin- AAA22974
1391	GQRYGKALGGMDAET	filamentous hemagglutinin- AAA22974
1392	EVDGIIQEFAADLRT	filamentous hemagglutinin- AAA22974
1393	KFIDQSKFYGSRYFF	filamentous hemagglutinin- AAA22974
1394	QIGYKPDRAARVAGD	filamentous hemagglutinin- AAA22974
1395	YFDTTLVREQVRRAL	filamentous hemagglutinin- AAA22974
1396	GYESRLPVRGVALVA	filamentous hemagglutinin- AAA22974
1397	LMDSAGTVGKALGLK	filamentous hemagglutinin- AAA22974
1398	GVAPTAQQLKQADRD	filamentous hemagglutinin- AAA22974
1399	LGSESGLEAHAGRGM	filamentous hemagglutinin- AAA22974
1400	AGAEVKVGYRASHEQ	filamentous hemagglutinin- AAA22974
1401	SETEKSYRNANLNFG	filamentous hemagglutinin- AAA22974
1402	GSVEAGNVLDIGGAD	filamentous hemagglutinin- AAA22974
1403	NRNRYGGAAKGNAGT	filamentous hemagglutinin- AAA22974
1404	EALRMRAKKVESTKY	filamentous hemagglutinin- AAA22974
1405	TGHVKGDANLTGATI	filamentous hemagglutinin- AAA22974
1406	DLSGKGNLKVDGAVN	filamentous hemagglutinin- AAA22974

1407	QNLKDYRDKDGGSGG	filamentous hemagglutinin- AAA22974
1408	NVGISSTTLAPTVGV	filamentous hemagglutinin- AAA22974
1409	FGRVAGEDYQAEQRA	filamentous hemagglutinin- AAA22974
1410	IDVGQTKDPARLQVG	filamentous hemagglutinin- AAA22974
1411	GGKLPKPVTVKLTDE	filamentous hemagglutinin- AAA22974
1412	GKPQTYTINRREDLM	filamentous hemagglutinin- AAA22974
1413	LNGKVLSTKTTLGLE	filamentous hemagglutinin- AAA22974
1414	TFRLRSRISAARTTG	filamentous hemagglutinin- AAA22974
1415	FQRALRLCLRAALAA	Fim2 pilic subunit- CAD12823.1 Q8VVA0
1416	ASAAHADDGTIVITG	Fim2 pilic subunit- CAD12823.1 Q8VVA0
1417	ATKAKGVEFRLANLN	Serotype 3 fimbrial subunit-CAA35920 P17835
1418	QHIRMGTDKTTQAAQ	Serotype 3 fimbrial subunit-CAA35920 P17835
1419	FTGKVTNGSKSYTLR	Serotype 3 fimbrial subunit-CAA35920 P17835
1420	LASYVKKPKEDVDAA	Serotype 3 fimbrial subunit-CAA35920 P17835
1421	AAQITSYVGFSVVYP	Serotype 3 fimbrial subunit-CAA35920 P17835
1422	CRGAVRALALALLGA	tracheal colon factor- CAA08832. O86135
1423	GEQDAPEVPPVAPAP	tracheal colon factor- CAA08832. O86135
1424	AGNGVYDPGTHTLTT	tracheal colon factor- CAA08832. O86135
1425	ASAAVSLASSSHGVW	tracheal colon factor- CAA08832. O86135
1426	AEMNALSKRMGELRL	tracheal colon factor- CAA08832. O86135
1427	PVAGGVWGRAFGRRQ	tracheal colon factor- CAA08832. O86135
1428	VDNRVSREFRQTISG	tracheal colon factor- CAA08832. O86135
1429	KSSDWGLQAGYIPVN	Bif. hemolysin-adenylate cyclaseprec- P15318
1430	NLSKLFGRAPEVIAR	Bif. hemolysin-adenylate cyclaseprec- P15318
1431	DNDVNSSLAHGHTAV	Bif. hemolysin-adenylate cyclaseprec- P15318
1432	LTLSKERLDYLRQAG	Bif. hemolysin-adenylate cyclaseprec- P15318
1433	VTGMADGVVASNHAG	Bif. hemolysin-adenylate cyclaseprec- P15318
1434	EQFEFRVKETSDGRY	Bif. hemolysin-adenylate cyclaseprec- P15318
1435	PGVSGASAHWGQRAL	Bif. hemolysin-adenylate cyclaseprec- P15318
1436	GAQAVAAAQRLVHAI	Bif. hemolysin-adenylate cyclaseprec- P15318
1437	LMTQFGRAGSTNTPQ	Bif. hemolysin-adenylate cyclaseprec- P15318
1438	AASLSAAVFGLGEAS	Bif. hemolysin-adenylate cyclaseprec- P15318
1439	AVAETVSGFFRGSSR	Bif. hemolysin-adenylate cyclaseprec- P15318
1440	AGGFGVAGGAMALGG	Bif. hemolysin-adenylate cyclaseprec- P15318
1441	KGERPALTFITPLAA	Bif. hemolysin-adenylate cyclaseprec- P15318
1442	GEEQRRRTKTGKSEF	Bif. hemolysin-adenylate cyclaseprec- P15318
1443	TFVEIVGKQDRWRIR	Bif. hemolysin-adenylate cyclaseprec- P15318
1444	GAADTTIDLAKVVSQ	Bif. hemolysin-adenylate cyclaseprec- P15318
1445	VDANGVLKHSIKLDV	Bif. hemolysin-adenylate cyclaseprec- P15318
1446	GGDGDDVVLANASRI	Bif. hemolysin-adenylate cyclaseprec- P15318
1447	HPGRIVAPHEYGFGI	Bif. hemolysin-adenylate cyclaseprec- P15318
1448	ADLSREWVRKASALG	Bif. hemolysin-adenylate cyclaseprec- P15318
1449	DYYDNVRNVENVIGT	Bif. hemolysin-adenylate cyclaseprec- P15318
1450	MKDVLIGDAQANTLM	Bif. hemolysin-adenylate cyclaseprec- P15318
1451	QGGDDTVRGGDGDDL	Bif. hemolysin-adenylate cyclaseprec- P15318
1452	FGGDGNDMLYGDAGN	Bif. hemolysin-adenylate cyclaseprec- P15318
1453	IYESGGGHDTIRINA	Bif. hemolysin-adenylate cyclaseprec- P15318

1454	ADQLWFARQGNDLEI	Bif. hemolysin-adenylate cyclaseprec- P15318
1455	ILGTDDALTVHDWYR	Bif. hemolysin-adenylate cyclaseprec- P15318
1456	ADHRVEIIHAANQAV	Bif. hemolysin-adenylate cyclaseprec- P15318
1457	QAGIEKLVEAMAQYP	Bif. hemolysin-adenylate cyclaseprec- P15318
1458	PGAAAAAPPAARVPD	Bif. hemolysin-adenylate cyclaseprec- P15318
1459	KFGGFGVNTFADGFK	Out. M.porin protein prec-CAA41398.1 Q04064
1460	NSYMVGLSAPIGGAS	Out. M.porin protein prec-CAA41398.1 Q04064
1461	VFGSWQMVDPKLTGG	Out. M.porin protein prec-CAA41398.1 Q04064
1462	EKMNVFSLGYTYDLS	Out. M.porin protein prec-CAA41398.1 Q04064
1463	RTNLYAYGSYAKNFA	Out. M.porin protein prec-CAA41398.1 Q04064
1464	LEDAKSTAVGVGIRH	Out. M.porin protein prec-CAA41398.1 Q04064
1465	MARPDWHWANGQQAS	Out. M.porin protein OmpQ- CAD12825. Q8VV98
1466	AYVVTLGYRQDLSAR	Out. M.porin protein OmpQ- CAD12825. Q8VV98
1467	SLYAYGGYMKGYDPE	Out. M.porin protein OmpQ- CAD12825. Q8VV98
1468	PFASDVGRATRFGVG	Out. M.porin protein OmpQ- CAD12825. Q8VV98
1469	LRNVAIIAHVDHGKT	GTP-binding elongation factor- Q7VYR0
1470	LVDQLLRQSGTFREN	GTP-binding elongation factor- Q7VYR0
1471	KFVTSRQIRDRLERE	GTP-binding elongation factor- Q7VYR0
1472	KSNVALRVRDTGDDT	GTP-binding elongation factor- Q7VYR0
1473	FEVSGRGELHLTILL	GTP-binding elongation factor- Q7VYR0
1474	NMRREGYELAVSRPR	GTP-binding elongation factor- Q7VYR0
1475	VFKEIDGVKCEPFEA	GTP-binding elongation factor- Q7VYR0
1476	TVDVEDAHQGGVMEE	GTP-binding elongation factor- Q7VYR0
1477	KPAIGVRVSGAGRAL	Bordetella resistance to killing- AAA51646
1478	LAGSTIDATEGGIPA	Bordetella resistance to killing- AAA51646
1479	VRRGGTLELDGVTVA	Bordetella resistance to killing- AAA51646
1480	GEGMEPMTVSDAGSR	Bordetella resistance to killing- AAA51646
1481	SVRGGVLGGEAPGVG	Bordetella resistance to killing- AAA51646
1482	VRAAQGGQASIIDAT	Bordetella resistance to killing- AAA51646
1483	VRLTDGATAQGGNGV	Bordetella resistance to killing- AAA51646
1484	LQQHSTIPVAVALES	Bordetella resistance to killing- AAA51646
1485	ALARGDIVADGNKPL	Bordetella resistance to killing- AAA51646
1486	AGISLSVASGAAWHG	Bordetella resistance to killing- AAA51646
1487	TQVLQSATLGKGGTW	Bordetella resistance to killing- AAA51646
1488	VNADSRVQDMSMRGG	Bordetella resistance to killing- AAA51646
1489	FAEPQAEVMLWRTSG	Bordetella resistance to killing- AAA51646
1490	RYRASNGLRVKVDAN	Bordetella resistance to killing- AAA51646
1491	ATLGRLGLRFGRRIA	Bordetella resistance to killing- AAA51646
1492	AGGNIVQPYARLGWT	Bordetella resistance to killing- AAA51646
1493	EFKSTGDVRTNGIGH	Bordetella resistance to killing- AAA51646
1494	GAGRHGRVELGAGVD	Bordetella resistance to killing- AAA51646
1495	THVSMNGGALSTSGA	Vag8 protein (Autotr.) CAD12828 Q8VV95
1496	SPAAWLLAGGSAQFR	Vag8 protein (Autotr.) CAD12828 Q8VV95
1497	TVLRTVGEASHGVDV	Vag8 protein (Autotr.) CAD12828 Q8VV95
1498	AHSEVELAHAQVRAD	Vag8 protein (Autotr.) CAD12828 Q8VV95
1499	QGAHGLVVTRSSAMV	Vag8 protein (Autotr.) CAD12828 Q8VV95
1500	AGSLVESTGDGAAAL	Vag8 protein (Autotr.) CAD12828 Q8VV95

1501	SPGGFWARGLSQRQR	Vag8 protein (Autotr.) CAD12828 Q8VV95
1502	DTGYGPWQKQTVSGI	Vag8 protein (Autotr.) CAD12828 Q8VV95
1503	LGLDRRVAGGATTAW	Vag8 protein (Autotr.) CAD12828 Q8VV95
1504	VGMLAGYSETRRDGG	Vag8 protein (Autotr.) CAD12828 Q8VV95
1505	YRAGHVHSAHVGAYV	Vag8 protein (Autotr.) CAD12828 Q8VV95
1506	YLNDSGSYVDGVVKY	Vag8 protein (Autotr.) CAD12828 Q8VV95
1507	LRNTDVHGEVAAIAL	putative autotransporter- AAC31207
1508	FNGEANISGGSLSVE	putative autotransporter- AAC31207
1509	GAVLTTLTPDAVEYY	putative autotransporter- AAC31207
1510	DYALSMEHLPADAPL	putative autotransporter- AAC31207
1511	PVRVTLSDGARASGE	putative autotransporter- AAC31207
1512	LIAHGGLLPMTLRLS	putative autotransporter- AAC31207
1513	AGRRFDQKVYGFELG	putative autotransporter- AAC31207
1514	DHAIAGQQGRWHVGG	putative autotransporter- AAC31207
1515	LGYTRARRSFIDDGA	putative autotransporter- AAC31207
1516	HTDSAHIGAYAAYVA	putative autotransporter- AAC31207
1517	NGFYFDSTLRASRFE	putative autotransporter- AAC31207
1518	DFTVTATDAVSVRGK	putative autotransporter- AAC31207
1519	QEQITQHGGPYGRCA	Pertussis toxin subunit 2 prec- P04978
1520	KTRALTVAELRGSGD	Pertussis toxin subunit 2 prec- P04978
1521	QEYLRHVTRGWSIFA	Pertussis toxin subunit 2 prec- P04978
1522	YDGTYLGGEYGGVIK	Pertussis toxin subunit 2 prec- P04978
1523	GTPGGAFDLKTTFCI	Pertussis toxin subunit 2 prec- P04978
1524	TTRNTGQPATDHYYS	Pertussis toxin subunit 2 prec- P04978
1525	EDATFQTYALTGISL	Pertussis toxin subunit 3 prec- P04979
1526	FPTRTTAPGQGGARR	Pertussis toxin subunit 4 prec- P0A3R5
1527	RVRALAWLLASGAMT	Pertussis toxin subunit 4 prec- P0A3R5
1528	LSPALADVPYVLVKT	Pertussis toxin subunit 4 prec- P0A3R5
1529	MVVTSVAMKPYEVTP	Pertussis toxin subunit 4 prec- P0A3R5
1530	RMLVCGIAAKLGAAA	Pertussis toxin subunit 4 prec- P0A3R5
1531	GIRRFLGTVTVKAGK	P.69A protein (pertactin)- CAA09473
1532	VADHATLANVGDTWD	P.69A protein (pertactin)- CAA09473
1533	DGIALYVAGEQAQAS	P.69A protein (pertactin)- CAA09473
1534	ADSTLQGAGGVQIER	P.69A protein (pertactin)- CAA09473
1535	ANVTVQRSAIVDGGL	P.69A protein (pertactin)- CAA09473
1536	IGALQSLQPEDLPPS	P.69A protein (pertactin)- CAA09473
1537	FADLGLSDKLVVMQD	P.69A protein (pertactin)- CAA09473
1538	SGQHRLWVRNSGSEP	P.69A protein (pertactin)- CAA09473
1539	SANTLLLVQTPLGSA	P.69A protein (pertactin)- CAA09473
1540	TFTLANKDGKVDIGT	P.69A protein (pertactin)- CAA09473
1541	RYRLAANGNGQWSLV	P.69A protein (pertactin)- CAA09473
1542	AKAPPAPKPAPQPGP	P.69A protein (pertactin)- CAA09473
1543	GMAAALGRGHSLYAS	P.69A protein (pertactin)- CAA09473
1544	EYSKGPKLAMPWTFH	P.69A protein (pertactin)- CAA09473
1545	LYRLVFSHVRGMLVP	filamentous hemagglutinin- AAA22974
1546	SEHCTVGNTFCGRTR	filamentous hemagglutinin- AAA22974
1547	QARSGARATSLSVAP	filamentous hemagglutinin- AAA22974

1548	ALAWALMLACTGLPL	filamentous hemagglutinin- AAA22974
1549	LASGGGAVNVAGGGA	filamentous hemagglutinin- AAA22974
1550	KIASASSVGNLAVQG	filamentous hemagglutinin- AAA22974
1551	GKVQATLLNAGGTLL	filamentous hemagglutinin- AAA22974
1552	SGRQAVQLGAASSRQ	filamentous hemagglutinin- AAA22974
1553	LSVNAGGALKADKLS	filamentous hemagglutinin- AAA22974
1554	TRRVDVDGKQAVALG	filamentous hemagglutinin- AAA22974
1555	GELTVSAARAATVAE	filamentous hemagglutinin- AAA22974
1556	KSLDNISVTGGERVS	filamentous hemagglutinin- AAA22974
1557	QSVNSASRVAISAHG	filamentous hemagglutinin- AAA22974
1558	LDVGKVSAKSGIGLE	filamentous hemagglutinin- AAA22974
1559	WGAVGADSLGSDGAI	filamentous hemagglutinin- AAA22974
1560	VSGRDAVRVDQARSL	filamentous hemagglutinin- AAA22974
1561	ALTENGTVTISADSA	filamentous hemagglutinin- AAA22974
1562	LEHSTIESKISQSVL	filamentous hemagglutinin- AAA22974
1563	AKGDKGKPAVSVKVA	filamentous hemagglutinin- AAA22974
1564	KLFLNGTLRAVNDNN	filamentous hemagglutinin- AAA22974
1565	TMSGRQIDVVDGRPQ	filamentous hemagglutinin- AAA22974
1566	TDAVTGEARKDESVV	filamentous hemagglutinin- AAA22974
1567	IVAASVSNPGTFTAG	filamentous hemagglutinin- AAA22974
1568	DITVTSRGGFDNEGK	filamentous hemagglutinin- AAA22974
1569	ESNKDIVIKTEQFSN	filamentous hemagglutinin- AAA22974
1570	RVLDAKHDLTVTASG	filamentous hemagglutinin- AAA22974
1571	ADNRGSLKAGHDFTV	filamentous hemagglutinin- AAA22974
1572	AQRIDNSGTMAAGHD	filamentous hemagglutinin- AAA22974
1573	GHGHIGGDVDNRSVV	filamentous hemagglutinin- AAA22974
1574	TVSAMEYFKTPLPVS	filamentous hemagglutinin- AAA22974
1575	TALDNRAGLSPATWN	filamentous hemagglutinin- AAA22974
1576	QSTYELLDYLLDQNR	filamentous hemagglutinin- AAA22974
1577	EYIWGLYPTYTEWSV	filamentous hemagglutinin- AAA22974
1578	TLKNLDLGYQAKPAP	filamentous hemagglutinin- AAA22974
1579	SADALASLASLDAAQ	filamentous hemagglutinin- AAA22974
1580	LEVSGRRNAQVADAG	filamentous hemagglutinin- AAA22974
1581	AGPSAVAAPAVGAAD	filamentous hemagglutinin- AAA22974
1582	GVEPVTGDQVDQPVV	filamentous hemagglutinin- AAA22974
1583	VGLEQPVATVRVAPP	filamentous hemagglutinin- AAA22974
1584	VALPRPLFETRIKFI	filamentous hemagglutinin- AAA22974
1585	LFAILSSTTETNQSA	filamentous hemagglutinin- AAA22974
1586	ANHYGTRIEAGTLEG	filamentous hemagglutinin- AAA22974
1587	MQNLEIEGGSVDAAH	filamentous hemagglutinin- AAA22974
1588	DLSVARDARFKAAAD	filamentous hemagglutinin- AAA22974
1589	AHAEHEKDVRQLSLG	filamentous hemagglutinin- AAA22974
1590	KVGAGGYEAGFSLGS	filamentous hemagglutinin- AAA22974
1591	IDAGKDLNLSGSRVR	filamentous hemagglutinin- AAA22974
1592	KHVVLDVEGDINATS	filamentous hemagglutinin- AAA22974
1593	QDERNYNSSGGGWDA	filamentous hemagglutinin- AAA22974
1594	AGVAIQNRTLVAPVG	filamentous hemagglutinin- AAA22974

1595	AGFNFNTEHDNSRLT	filamentous hemagglutinin- AAA22974
1596	DGAAGVVASDGLTGH	filamentous hemagglutinin- AAA22974
1597	PPVKPQKATPGPVAE	filamentous hemagglutinin- AAA22974
1598	GKATVTTVQVQSAPP	filamentous hemagglutinin- AAA22974
1599	PAPVAKQPAPAPKPK	filamentous hemagglutinin- AAA22974
1600	KPKPKAERPKPGKTT	filamentous hemagglutinin- AAA22974
1601	LSGRHVVQQQVQVLQ	filamentous hemagglutinin- AAA22974
1602	QASDINNTKSLPGGK	filamentous hemagglutinin- AAA22974
1603	SDQTCVIEEPSTLNH	Serotype 3 fimbrial subunit-CAA35920 P17835
1604	KVVQLPKISKNALRN	Serotype 3 fimbrial subunit-CAA35920 P17835
1605	GDTAGATPFDIKLKE	Serotype 3 fimbrial subunit-CAA35920 P17835
1606	PQALGALKLYFEPGI	Serotype 3 fimbrial subunit-CAA35920 P17835
1607	TNYDTGDLIAYKQTY	Serotype 3 fimbrial subunit-CAA35920 P17835
1608	ASGNGNLSTVSSATK	Serotype 3 fimbrial subunit-CAA35920 P17835
1609	GSKSPADGGQDGPPP	tracheal colon factor- CAA08832. O86135
1610	RDGGDADPQPPRDDG	tracheal colon factor- CAA08832. O86135
1611	GEQQPPKGGGDEGQR	tracheal colon factor- CAA08832. O86135
1612	PPAAGNGGNGGNGNA	tracheal colon factor- CAA08832. O86135
1613	LPERGDDAGPKPPEG	tracheal colon factor- CAA08832. O86135
1614	GGDEGPQPPQGGGEQ	tracheal colon factor- CAA08832. O86135
1615	VGVASQLGKHGSLFG	tracheal colon factor- CAA08832. O86135
1616	YEYAKGSRQTMPWTF	tracheal colon factor- CAA08832. O86135
1617	HQAGYANAADRESGI	Bif. hemolysin-adenylate cyclaseprec- P15318
1618	AAVLDGIKAVAKEKN	Bif. hemolysin-adenylate cyclaseprec- P15318
1619	TLMFRLVNPHSTSLI	Bif. hemolysin-adenylate cyclaseprec- P15318
1620	EGVATKGLGVHAKSS	Bif. hemolysin-adenylate cyclaseprec- P15318
1621	LFDDGLGAAPGVPSG	Bif. hemolysin-adenylate cyclaseprec- P15318
1622	SKFSPDVLETVPASP	Bif. hemolysin-adenylate cyclaseprec- P15318
1623	LRRPSLGAVERQDSG	Bif. hemolysin-adenylate cyclaseprec- P15318
1624	DSLDGVGSRSFSLGE	Bif. hemolysin-adenylate cyclaseprec- P15318
1625	SDMAAVEAAELEMTR	Bif. hemolysin-adenylate cyclaseprec- P15318
1626	VLHAGARQDDAEPGV	Bif. hemolysin-adenylate cyclaseprec- P15318
1627	KNLQARHEQLANSDG	Bif. hemolysin-adenylate cyclaseprec- P15318
1628	RKMLADLQAGWNASS	Bif. hemolysin-adenylate cyclaseprec- P15318
1629	IGVQTTEISKSALEL	Bif. hemolysin-adenylate cyclaseprec- P15318
1630	AITGNADNLKSVDVF	Bif. hemolysin-adenylate cyclaseprec- P15318
1631	DRFVQGERVAGQPVV	Bif. hemolysin-adenylate cyclaseprec- P15318
1632	DVAAGGIDIASRKGE	Bif. hemolysin-adenylate cyclaseprec- P15318
1633	FSVDHVKNIENLHGS	Bif. hemolysin-adenylate cyclaseprec- P15318
1634	LNDRIAGDDQDNELW	Bif. hemolysin-adenylate cyclaseprec- P15318
1635	HDGNDTIRGRGGDDI	Bif. hemolysin-adenylate cyclaseprec- P15318
1636	RGGLGLDTLYGEDGN	Bif. hemolysin-adenylate cyclaseprec- P15318
1637	IFLQDDETVSDDIDG	Bif. hemolysin-adenylate cyclaseprec- P15318
1638	AGLDTVDYSAMIHPG	Bif. hemolysin-adenylate cyclaseprec- P15318
1639	ASLMDEPETSNVLRN	Bif. hemolysin-adenylate cyclaseprec- P15318
1640	ENAVGSARDDVLIGD	Bif. hemolysin-adenylate cyclaseprec- P15318
1641	GANVLNGLAGNDVLS	Bif. hemolysin-adenylate cyclaseprec- P15318

1642	GAGDDVLLGDEGSDL	Bif. hemolysin-adenylate cyclaseprec- P15318
1643	SGDAGNDDLFGGQGD	Bif. hemolysin-adenylate cyclaseprec- P15318
1644	TYLFGVGYGHDTIYE	Bif. hemolysin-adenylate cyclaseprec- P15318
1645	NRVGFATADNVRAIT	Out. M.porin protein prec-CAA41398.1 Q04064
1646	GLRYVNGPLNVALSY	Out. M.porin protein prec-CAA41398.1 Q04064
1647	QLNASNNQAQGEVDA	Out. M.porin protein prec-CAA41398.1 Q04064
1648	PRSYGLGGSYDFEVV	Out. M.porin protein prec-CAA41398.1 Q04064
1649	LALAYARTTDGWFGG	Out. M.porin protein prec-CAA41398.1 Q04064
1650	GYPVAVTLPSGDKFG	Out. M.porin protein prec-CAA41398.1 Q04064
1651	AAFTWDKLNLHDTSA	Out. M.porin protein OmpQ- CAD12825. Q8VV98
1652	GGRSPQALQAGFTYD	Out. M.porin protein OmpQ- CAD12825. Q8VV98
1653	EALKMALAWSRQRNG	Out. M.porin protein OmpQ- CAD12825. Q8VV98
1654	VGLNGGGQIGLGPEP	Out. M.porin protein OmpQ- CAD12825. Q8VV98
1655	AHGGAINAWLLGLEV	Out. M.porin protein OmpQ- CAD12825. Q8VV98
1656	VHGNGAWLVQGSMAR	Out. M.porin protein OmpQ- CAD12825. Q8VV98
1657	GNMDVAFRFGPDGEV	GTP-binding elongation factor- Q7VYR0
1658	RGRINQVMKFSGLER	GTP-binding elongation factor- Q7VYR0
1659	VVDEAEAGDIVLVNG	GTP-binding elongation factor- Q7VYR0
1660	EDLHIGSTITDPSTP	GTP-binding elongation factor- Q7VYR0
1661	GLPVLRIDEPTLTMN	GTP-binding elongation factor- Q7VYR0
1662	MVNTSPLAGREGKFV	GTP-binding elongation factor- Q7VYR0
1663	RLHALAAALALAGMA	Bordetella resistance to killing- AAA51646
1664	LAPAAAQAPQPPVAG	Bordetella resistance to killing- AAA51646
1665	PHAQDAGQEGEFDHR	Bordetella resistance to killing- AAA51646
1666	NTLIAVFDDGVGINL	Bordetella resistance to killing- AAA51646
1667	DDPDELGETAPPTLK	Bordetella resistance to killing- AAA51646
1668	IHISVEHKNPMSKPA	Bordetella resistance to killing- AAA51646
1669	QSGSGPASVDMQGGS	Bordetella resistance to killing- AAA51646
1670	TTTGNRAAGIALTHG	Bordetella resistance to killing- AAA51646
1671	ARLEGVAVRAEGSGS	Bordetella resistance to killing- AAA51646
1672	AAQLANGTLVVSAGS	Bordetella resistance to killing- AAA51646
1673	ASAQSGAISVTDTPL	Bordetella resistance to killing- AAA51646
1674	LMPGALASSTVSVRL	Bordetella resistance to killing- AAA51646
1675	RWYAGGLLGYTYADR	Bordetella resistance to killing- AAA51646
1676	YPGDGGGKVKGLHVG	Bordetella resistance to killing- AAA51646
1677	YAAYVGDGGYYLDTV	Bordetella resistance to killing- AAA51646
1678	RLGRYDQQYNIAGTD	Bordetella resistance to killing- AAA51646
1679	GRVTADYRTSGAAWS	Bordetella resistance to killing- AAA51646
1680	EGGRRFELPNDWFAE	Bordetella resistance to killing- AAA51646
1681	LETAGQQAPAVVLWQ	Vag8 protein (Autotr.) CAD12828 Q8VV95
1682	AQLNAQGLVVQVNGA	Vag8 protein (Autotr.) CAD12828 Q8VV95
1683	VSAIHAQDAGSFTLS	Vag8 protein (Autotr.) CAD12828 Q8VV95
1684	SDITARGLEVAGIYV	Vag8 protein (Autotr.) CAD12828 Q8VV95
1685	EGMQGTLTGTRVTTQ	Vag8 protein (Autotr.) CAD12828 Q8VV95
1686	DTAPALQVEDAGTHV	Vag8 protein (Autotr.) CAD12828 Q8VV95
1687	TKVPLIEDEQGHTAF	Vag8 protein (Autotr.) CAD12828 Q8VV95
1688	LGNMGGRVDAGARQY	Vag8 protein (Autotr.) CAD12828 Q8VV95

1689	LTASEAQADKARTWQ	Vag8 protein (Autotr.) CAD12828 Q8VV95
1690	TPTNELSTTATAAVN	Vag8 protein (Autotr.) CAD12828 Q8VV95
1691	MAIAASQRIWQAEMD	Vag8 protein (Autotr.) CAD12828 Q8VV95
1692	LLRHMSGLHSIGSPG	Vag8 protein (Autotr.) CAD12828 Q8VV95
1693	GSGKVAIENAELLGA	putative autotransporter- AAC31207
1694	GMYATFGAQVDMKGG	putative autotransporter- AAC31207
1695	ILAHNTNILGSQGYA	putative autotransporter- AAC31207
1696	GPYGGVVVTEDGQVN	putative autotransporter- AAC31207
1697	EGAKVSATGLGAAGL	putative autotransporter- AAC31207
1698	LLGDKDTSPRASLRN	putative autotransporter- AAC31207
1699	KFTLANRGGVVDAGA	putative autotransporter- AAC31207
1700	RYRLTPDNGVWGLER	putative autotransporter- AAC31207
1701	SQLSAVANAALNTGG	putative autotransporter- AAC31207
1702	GAASSIWYAEGNALS	putative autotransporter- AAC31207
1703	RLGELRLDPGAGGFW	putative autotransporter- AAC31207
1704	RTFAQKQQLDNKAGR	putative autotransporter- AAC31207
1705	VSQQTRANPNPYTSR	Pertussis toxin subunit 1 prec- O69258
1706	SVASIVGTLVRIAPV	Pertussis toxin subunit 1 prec- O69258
1707	GACMARQAESSEAMA	Pertussis toxin subunit 1 prec- O69258
1708	WSERAGEAMVLVYYE	Pertussis toxin subunit 1 prec- O69258
1709	RKTLCHLLSVLPLAL	Pertussis toxin subunit 2 prec- P04978
1710	GSHVARASTPGIVIP	Pertussis toxin subunit 2 prec- P04978
1711	IYRETFCITTIYKTG	Pertussis toxin subunit 3 prec- P04979
1712	PAADHYYSKVTATRL	Pertussis toxin subunit 3 prec- P04979
1713	ASTNSRLCAVFVRDG	Pertussis toxin subunit 3 prec- P04979
1714	SVIGACASPYEGRYR	Pertussis toxin subunit 3 prec- P04979
1715	MYDALRRLLYMIYMS	Pertussis toxin subunit 3 prec- P04979
1716	LAVRVHVSKEEQYYD	Pertussis toxin subunit 3 prec- P04979
1717	VKAAPLRRTTLAMAL	P.69A protein (pertactin)- CAA09473
1718	ALGAAPAAHADWNNQ	P.69A protein (pertactin)- CAA09473
1719	IVKTGERQHGIHIQG	P.69A protein (pertactin)- CAA09473
1720	DPGGVRTASGTTIKV	P.69A protein (pertactin)- CAA09473
1721	GRQAQGILLENPAAE	P.69A protein (pertactin)- CAA09473
1722	QFRNGSVTSSGQLSD	P.69A protein (pertactin)- CAA09473
1723	AQGDIVATELPSIPG	P.69A protein (pertactin)- CAA09473
1724	SIGPLDVALASQARW	P.69A protein (pertactin)- CAA09473
1725	GATRAVDSLSIDNAT	P.69A protein (pertactin)- CAA09473
1726	VMTDNSNVGALRLAS	P.69A protein (pertactin)- CAA09473
1727	GSVDFQQPAEAGRFK	P.69A protein (pertactin)- CAA09473
1728	LTVNTLAGSGLFRMN	P.69A protein (pertactin)- CAA09473
1729	ADGWFLEPQAELAVF	P.69A protein (pertactin)- CAA09473
1730	AGGGAYRAANGLRVR	P.69A protein (pertactin)- CAA09473
1731	EGGSSVLGRLGLEVG	P.69A protein (pertactin)- CAA09473
1732	RIELAGGRQVQPYIK	P.69A protein (pertactin)- CAA09473
1733	SVLQEFDGAGTVHTN	P.69A protein (pertactin)- CAA09473
1734	IAHRTELRGTRAELG	P.69A protein (pertactin)- CAA09473
1735	ANRYDHATRRATPIA	filamentous hemagglutinin- AAA22974

1736	GARGAAAGAYAIDGT	filamentous hemagglutinin- AAA22974
1737	AGAMYGKHITLVSSD	filamentous hemagglutinin- AAA22974
1738	GLGVRQLGSLSSPSA	filamentous hemagglutinin- AAA22974
1739	TVSSQGEIALGDATV	filamentous hemagglutinin- AAA22974
1740	RGPLSLKGAGVVSAG	filamentous hemagglutinin- AAA22974
1741	RGAVTVSGGGAVNLG	filamentous hemagglutinin- AAA22974
1742	VQSDGQVRATSAGAM	filamentous hemagglutinin- AAA22974
1743	VRDVAAAADLALQAG	filamentous hemagglutinin- AAA22974
1744	ALQAGFLKSAGAMTV	filamentous hemagglutinin- AAA22974
1745	GRDAVRLDGAHAGGQ	filamentous hemagglutinin- AAA22974
1746	RVSSDGQAALGSLAA	filamentous hemagglutinin- AAA22974
1747	ELSGQGVTVDRASAS	filamentous hemagglutinin- AAA22974
1748	ARIDSTGSVGIGALK	filamentous hemagglutinin- AAA22974
1749	GAVEAASPRRARRAL	filamentous hemagglutinin- AAA22974
1750	QDFFTPGSVVVRAQG	filamentous hemagglutinin- AAA22974
1751	VTVGRGDPHQGVLAQ	filamentous hemagglutinin- AAA22974
1752	DIIMDAKGGTLLLRN	filamentous hemagglutinin- AAA22974
1753	AIALAAQVTQRGGAA	filamentous hemagglutinin- AAA22974
1754	LTSRHDTRFSNKIRL	filamentous hemagglutinin- AAA22974
1755	GPLQVNAGGPVSNTG	filamentous hemagglutinin- AAA22974
1756	LKVREGVTVTAASFD	filamentous hemagglutinin- AAA22974
1757	ETGAEVMAKSATLTT	filamentous hemagglutinin- AAA22974
1758	GAARNAGKMQVKEAA	filamentous hemagglutinin- AAA22974
1759	APRIENTAKLSGEVQ	filamentous hemagglutinin- AAA22974
1760	KGVQDVGGGEHGRWS	filamentous hemagglutinin- AAA22974
1761	IGYVNYWLRAGNGKK	filamentous hemagglutinin- AAA22974
1762	GTIAAPWYGGDLTAE	filamentous hemagglutinin- AAA22974
1763	SLIEVGKDLYLNAGA	filamentous hemagglutinin- AAA22974
1764	KDEHRHLLNEGVIQA	filamentous hemagglutinin- AAA22974
1765	NGENAAQNRGRPEGL	filamentous hemagglutinin- AAA22974
1766	IGAHSATSVSGSFDA	filamentous hemagglutinin- AAA22974
1767	RDVGLEKRLDIDDAL	filamentous hemagglutinin- AAA22974
1768	AVLVNPHIFTRIGAA	filamentous hemagglutinin- AAA22974
1769	TSLADGAAGPALARQ	filamentous hemagglutinin- AAA22974
1770	RQAPETDGMVDARGL	filamentous hemagglutinin- AAA22974
1771	GIEGGKLRGKDVRLK	filamentous hemagglutinin- AAA22974
1772	DTVKVATSMRYDDKG	filamentous hemagglutinin- AAA22974
1773	LAARGDGALDAQGGQ	filamentous hemagglutinin- AAA22974
1774	HIEAKRLETAGATLK	filamentous hemagglutinin- AAA22974
1775	GKVKLDVDDVKLGGV	filamentous hemagglutinin- AAA22974
1776	EAGSSYENKSSTPLG	filamentous hemagglutinin- AAA22974
1777	EATEGDATLVGAKFG	filamentous hemagglutinin- AAA22974
1778	GDQVSLKAAKSVNLM	filamentous hemagglutinin- AAA22974
1779	AESTFESYSESHNFH	filamentous hemagglutinin- AAA22974
1780	SADANLGANAVQGAV	filamentous hemagglutinin- AAA22974
1781	LGLTAGMGTSHQITN	filamentous hemagglutinin- AAA22974
1782	TGKTYAGTSVDAANV	filamentous hemagglutinin- AAA22974

1783	LPPRPVAAQVVPVTP	filamentous hemagglutinin- AAA22974
1784	KVEVAKVEVVPRPKV	filamentous hemagglutinin- AAA22974
1785	TAQPLPPRPVVAEKV	filamentous hemagglutinin- AAA22974
1786	TPAVQPQLAKVETVQ	filamentous hemagglutinin- AAA22974
1787	VKPETTKPLPKPLPV	filamentous hemagglutinin- AAA22974
1788	KVTKAPPPVVETAQP	filamentous hemagglutinin- AAA22974
1789	VQVRISNLNDSKITM	Fim2 pilic subunit- CAD12823.1 Q8VVA0
1790	ANEATQQAAGFDPEV	Fim2 pilic subunit- CAD12823.1 Q8VVA0
1791	TGGTSKTVTMRYLAS	Fim2 pilic subunit- CAD12823.1 Q8VVA0
1792	VKKNGDVEASAITTY	Fim2 pilic subunit- CAD12823.1 Q8VVA0
1793	SYPALRAALILAASP	Serotype 3 fimbrial subunit-CAA35920 P17835
1794	LPALANDGTIVITGS	Serotype 3 fimbrial subunit-CAA35920 P17835
1795	VGGGTHENGLPGIGK	tracheal colon factor- CAA08832. O86135
1796	GGSAPGPDTSTGSGP	tracheal colon factor- CAA08832. O86135
1797	AGMASGAGSTSPGAS	tracheal colon factor- CAA08832. O86135
1798	GAGKDAMPPSEGERP	tracheal colon factor- CAA08832. O86135
1799	SGMSDSGRGGESSAG	tracheal colon factor- CAA08832. O86135
1800	LNPDGAGKPPREEGE	tracheal colon factor- CAA08832. O86135
1801	WPGAWYVEPQLEVAA	tracheal colon factor- CAA08832. O86135
1802	HAQGADYTASNGLRI	tracheal colon factor- CAA08832. O86135
1803	DDGTNSMLGRLGLHV	tracheal colon factor- CAA08832. O86135
1804	RQFDLGDGRVVQPYM	tracheal colon factor- CAA08832. O86135
1805	LSWVQEFDGKGTVRT	tracheal colon factor- CAA08832. O86135
1806	DIRHKVRLDGGRTEL	tracheal colon factor- CAA08832. O86135
1807	FRYDGDMNIGVITDF	Bif. hemolysin-adenylate cyclaseprec- P15318
1808	LEVRNALNRRAHAVG	Bif. hemolysin-adenylate cyclaseprec- P15318
1809	QDVVQHGTEQNNPFP	Bif. hemolysin-adenylate cyclaseprec- P15318
1810	ADEKIFVVSATGESQ	Bif. hemolysin-adenylate cyclaseprec- P15318
1811	LTRGQLKEYIGQQRG	Bif. hemolysin-adenylate cyclaseprec- P15318
1812	GYVFYENRAYGVAGK	Bif. hemolysin-adenylate cyclaseprec- P15318
1813	AYGYEGDALLAQLYR	Bif. hemolysin-adenylate cyclaseprec- P15318
1814	KTAAEGAVAGVSAVL	Bif. hemolysin-adenylate cyclaseprec- P15318
1815	TVGAAVSIAAAASVV	Bif. hemolysin-adenylate cyclaseprec- P15318
1816	APVAVVTSLLTGALN	Bif. hemolysin-adenylate cyclaseprec- P15318
1817	ILRGVQQPIIEKLAN	Bif. hemolysin-adenylate cyclaseprec- P15318
1818	YARKIDELGGPQAYF	Bif. hemolysin-adenylate cyclaseprec- P15318
1819	NFLAGGSGDDRLDGG	Bif. hemolysin-adenylate cyclaseprec- P15318
1820	GNDTLVGGEGQNTVI	Bif. hemolysin-adenylate cyclaseprec- P15318
1821	GAGDDVFLQDLGVWS	Bif. hemolysin-adenylate cyclaseprec- P15318
1822	QLDGGAGVDTVKYNV	Bif. hemolysin-adenylate cyclaseprec- P15318
1823	QPSEERLERMGDTGI	Bif. hemolysin-adenylate cyclaseprec- P15318
1824	ADLQKGTVEKWPALN	Bif. hemolysin-adenylate cyclaseprec- P15318
1825	DAQANVLRGAGGADV	Bif. hemolysin-adenylate cyclaseprec- P15318
1826	AGGEGDDVLLGGDGD	Bif. hemolysin-adenylate cyclaseprec- P15318
1827	QLSGDAGRDRLYGEA	Bif. hemolysin-adenylate cyclaseprec- P15318
1828	DDWFFQDAANAGNLL	Bif. hemolysin-adenylate cyclaseprec- P15318
1829	GGDGRDTVDFSGPGR	Bif. hemolysin-adenylate cyclaseprec- P15318

1830	LDAGAKGVFLSLGKG	Bif. hemolysin-adenylate cyclaseprec- P15318
1831	SAQDGRLFGRQATIG	Out. M.porin protein prec-CAA41398.1 Q04064
1832	QSESWGRLDFGRQTN	Out. M.porin protein prec-CAA41398.1 Q04064
1833	ASKYFGSIDPFGAGF	Out. M.porin protein prec-CAA41398.1 Q04064
1834	QANIGMGMSAMNTVR	Out. M.porin protein prec-CAA41398.1 Q04064
1835	DNMVMYQTPSYSGFQ	Out. M.porin protein prec-CAA41398.1 Q04064
1836	GIGYSFSANDKDADA	Out. M.porin protein prec-CAA41398.1 Q04064
1837	GHAGVGELSLGRQQS	Out. M.porin protein OmpQ- CAD12825. Q8VV98
1838	GLQYGGQLEIASWRD	Out. M.porin protein OmpQ- CAD12825. Q8VV98
1839	GMGALFKASDNYRVN	Out. M.porin protein OmpQ- CAD12825. Q8VV98
1840	LVNYLSPEFSGWQWG	Out. M.porin protein OmpQ- CAD12825. Q8VV98
1841	GYAFDVESGDTGRFD	Out. M.porin protein OmpQ- CAD12825. Q8VV98
1842	SPAFSTGLKYEDGPL	Out. M.porin protein OmpQ- CAD12825. Q8VV98
1843	PDFVINATFELFDKL	GTP-binding elongation factor- Q7VYR0
1844	ATEEQLDFPVVYASG	GTP-binding elongation factor- Q7VYR0
1845	SGYAGLTDDVRSGDM	GTP-binding elongation factor- Q7VYR0
1846	PLFEAIMKYVPQRDD	GTP-binding elongation factor- Q7VYR0
1847	ANGPLQMQIISLDYN	GTP-binding elongation factor- Q7VYR0
1848	YVGKIGVGRINRGRM	GTP-binding elongation factor- Q7VYR0
1849	EGMIIGIHSRDNDLV	GTP-binding elongation factor- Q7VYR0
1850	NPIKGKQLTNVRASG	GTP-binding elongation factor- Q7VYR0
1851	DEAVRLVPPIQMSLE	GTP-binding elongation factor- Q7VYR0
1852	AVEFIDDDELVEITP	GTP-binding elongation factor- Q7VYR0
1853	SIRLRKRYLQEHERR	GTP-binding elongation factor- Q7VYR0
1854	RFRQCPSSLQIPRSA	Bordetella resistance to killing- AAA51646
1855	DGAGVVAGAGLLDAL	Bordetella resistance to killing- AAA51646
1856	PGGTVRLDGTTVSTD	Bordetella resistance to killing- AAA51646
1857	ANTDAVLVRGDAARA	Bordetella resistance to killing- AAA51646
1858	VVNTVLRTAKSLAAG	Bordetella resistance to killing- AAA51646
1859	SAQHGGRVTLRQTRI	Bordetella resistance to killing- AAA51646
1860	TAGAGAEGISVLGFE	Bordetella resistance to killing- AAA51646
1861	EDPKTHVWSLQRAGQ	Bordetella resistance to killing- AAA51646
1862	LSGAANAAVNAADLS	Bordetella resistance to killing- AAA51646
1863	IALAESNALDKRLGE	Bordetella resistance to killing- AAA51646
1864	RLRADAGGPWARTFS	Bordetella resistance to killing- AAA51646
1865	RQQISNRHARAYDQT	Bordetella resistance to killing- AAA51646
1866	SGLEIGLDRGWSASG	Bordetella resistance to killing- AAA51646
1867	VRVTGGTIRTSGNQA	Vag8 protein (Autotr.) CAD12828 Q8VV95
1868	GLRVGTENAPDNTAL	Vag8 protein (Autotr.) CAD12828 Q8VV95
1869	ASVFLQNLIIETSGT	Vag8 protein (Autotr.) CAD12828 Q8VV95
1870	ALGVSVHEPQGGGGT	Vag8 protein (Autotr.) CAD12828 Q8VV95
1871	LSMSGTTVRTRGDDS	Vag8 protein (Autotr.) CAD12828 Q8VV95
1872	ALQLSGPASATLNDV	Vag8 protein (Autotr.) CAD12828 Q8VV95
1873	VHTVRLLDRGVWTVT	Vag8 protein (Autotr.) CAD12828 Q8VV95
1874	DSRVAEVKLEGGTLA	Vag8 protein (Autotr.) CAD12828 Q8VV95
1875	APPAQPKGAFKTLVA	Vag8 protein (Autotr.) CAD12828 Q8VV95
1876	QGISGTGTIVMNAHL	Vag8 protein (Autotr.) CAD12828 Q8VV95

1877	SGTADVLVAPQGFGD	Vag8 protein (Autotr.) CAD12828 Q8VV95
1878	QVLVVNNTDDGTESG	Vag8 protein (Autotr.) CAD12828 Q8VV95
1879	LGWAQELGADNAVYT	Vag8 protein (Autotr.) CAD12828 Q8VV95
1880	GIRHVTRSRGGFAEA	Vag8 protein (Autotr.) CAD12828 Q8VV95
1881	VGVGALLGKRHALYA	Vag8 protein (Autotr.) CAD12828 Q8VV95
1882	YEYAKGARFEAPWTL	Vag8 protein (Autotr.) CAD12828 Q8VV95
1883	CRDDDGTSPSIRVQG	putative autotransporter- AAC31207
1884	VVQGGMGANNVAVVA	putative autotransporter- AAC31207
1885	TVTGSSTVNSLHLQA	putative autotransporter- AAC31207
1886	KVAYATPAESDGEFK	putative autotransporter- AAC31207
1887	LRVKTLSGSGLFEMN	putative autotransporter- AAC31207
1888	SADLSDGDLLVVSDE	putative autotransporter- AAC31207
1889	SGQHKVLVRGAGTEP	putative autotransporter- AAC31207
1890	GVESLTLVELPEGSQ	putative autotransporter- AAC31207
1891	RTDLSGGRAELALGL	putative autotransporter- AAC31207
1892	AALGRGHQLYTSYEY	putative autotransporter- AAC31207
1893	KGNKLTLPWTFHLGY	putative autotransporter- AAC31207
1894	AVEAERAGRGTGHFI	Pertussis toxin subunit 1 prec- O69258
1895	YIYEVRADNNFYGAA	Pertussis toxin subunit 1 prec- O69258
1896	SYFEYVDTYGDNAGR	Pertussis toxin subunit 1 prec- O69258
1897	LAGALATYQSEYLAH	Pertussis toxin subunit 1 prec- O69258
1898	GITGETTTTEYSNAR	Pertussis toxin subunit 1 prec- O69258
1899	LPILVLALLGMRTAQ	Pertussis toxin subunit 3 prec- P04979
1900	VAPGIVIPPKALFTQ	Pertussis toxin subunit 3 prec- P04979
1901	GGAYGRCPNGTRALT	Pertussis toxin subunit 3 prec- P04979
1902	AELRGNAELQTYLRQ	Pertussis toxin subunit 3 prec- P04979
1903	TPGWSIYGLYDGTYL	Pertussis toxin subunit 3 prec- P04979
1904	QAYGGIIKDAPPGAG	Pertussis toxin subunit 3 prec- P04979
1905	PTHLYKNFTVQELAL	Pertussis toxin subunit 5 prec- P04981
1906	LKGKNQEFCLTAFMS	Pertussis toxin subunit 5 prec- P04981
1907	RSLVRACLSDAGHEH	Pertussis toxin subunit 5 prec- P04981
1908	TWFDTMLGFAISAYA	Pertussis toxin subunit 5 prec- P04981
1909	KSRIALTVEDSPYPG	Pertussis toxin subunit 5 prec- P04981
1910	PGDLLELQICPLNGY	Pertussis toxin subunit 5 prec- P04981
1911	GVDVSGSSVELAQSI	P.69A protein (pertactin)- CAA09473
1912	EAPELGAAIRVGRGA	P.69A protein (pertactin)- CAA09473
1913	VTVSGGSLSAPHGNV	P.69A protein (pertactin)- CAA09473
1914	ETGGARRFAPQAAPL	P.69A protein (pertactin)- CAA09473
1915	ITLQAGAHAQGKALL	P.69A protein (pertactin)- CAA09473
1916	RVLPEPVKLTLTGGA	P.69A protein (pertactin)- CAA09473
1917	HAVAVAGGRWHLGGL	P.69A protein (pertactin)- CAA09473
1918	GYTRGDRGFTGDGGG	P.69A protein (pertactin)- CAA09473
1919	TDSVHVGGYATYIAD	P.69A protein (pertactin)- CAA09473
1920	GFYLDATLRASRLEN	P.69A protein (pertactin)- CAA09473
1921	FKVAGSDGYAVKGKY	P.69A protein (pertactin)- CAA09473
1922	THGVGASLEAGRRFT	P.69A protein (pertactin)- CAA09473
1923	VYGKGADLIIANPNG	filamentous hemagglutinin- AAA22974

1924	SVNGLSTLNASNLTL	filamentous hemagglutinin- AAA22974
1925	TGRPSVNGGRIGLDV	filamentous hemagglutinin- AAA22974
1926	QGTVTIERGGVNATG	filamentous hemagglutinin- AAA22974
1927	GYFDVVARLVKLQGA	filamentous hemagglutinin- AAA22974
1928	SSKQGKPLADIAVVA	filamentous hemagglutinin- AAA22974
1929	SKGAIGVQGGEAVSV	filamentous hemagglutinin- AAA22974
1930	NANSDAELRVRGRGQ	filamentous hemagglutinin- AAA22974
1931	DLHDLSAARGADISG	filamentous hemagglutinin- AAA22974
1932	GRVNIGRARSDSDVK	filamentous hemagglutinin- AAA22974
1933	SAHGALSIDSMTALG	filamentous hemagglutinin- AAA22974
1934	IGVQAGGSVSAKDMR	filamentous hemagglutinin- AAA22974
1935	LHVQGGEHLDLGTLA	filamentous hemagglutinin- AAA22974
1936	VGAVDVNGTGDVRVA	filamentous hemagglutinin- AAA22974
1937	LVSDAGADLQAGRSM	filamentous hemagglutinin- AAA22974
1938	LGIVDTTGDLQARAQ	filamentous hemagglutinin- AAA22974
1939	KLELGSVKSDGGLQA	filamentous hemagglutinin- AAA22974
1940	AGGALSLAAAEVAGA	filamentous hemagglutinin- AAA22974
1941	GTAGALVVKAAEAIV	filamentous hemagglutinin- AAA22974
1942	DGVMATKGEMQIAGK	filamentous hemagglutinin- AAA22974
1943	GGSPTVTAGAKATTS	filamentous hemagglutinin- AAA22974
1944	NKLSVDVASWDNAGS	filamentous hemagglutinin- AAA22974
1945	DIKKGGAQVTVAGRY	filamentous hemagglutinin- AAA22974
1946	EHGEVSIQGDYTVSA	filamentous hemagglutinin- AAA22974
1947	ETGNPGSLIAEVQEN	filamentous hemagglutinin- AAA22974
1948	DNKQAIVVGKDLTLS	filamentous hemagglutinin- AAA22974
1949	AHGNVANEANALLWA	filamentous hemagglutinin- AAA22974
1950	GELTVKAQNITNKRA	filamentous hemagglutinin- AAA22974
1951	LIEAGGNARLTAAVA	filamentous hemagglutinin- AAA22974
1952	LNKLGRIRAGEDMHL	filamentous hemagglutinin- AAA22974
1953	ADQATIDAETDKVAQ	filamentous hemagglutinin- AAA22974
1954	YKSQIDAVRLQAIQP	filamentous hemagglutinin- AAA22974
1955	RVTLAKALSAALGAD	filamentous hemagglutinin- AAA22974
1956	RALGHSQLMQRWKDF	filamentous hemagglutinin- AAA22974
1957	AGKRGAEIAFYPKEQ	filamentous hemagglutinin- AAA22974
1958	VLAAGAGLTLSNGAI	filamentous hemagglutinin- AAA22974
1959	DTVIDGQKVLAPRLY	filamentous hemagglutinin- AAA22974
1960	TEATRQGITDQYAGG	filamentous hemagglutinin- AAA22974
1961	ALIASGGDVTVNTDG	filamentous hemagglutinin- AAA22974
1962	DVSSVNGLIQGRSVK	filamentous hemagglutinin- AAA22974
1963	DAGKGKVVVADSKGA	filamentous hemagglutinin- AAA22974
1964	GGIEADDEVDVSGRD	filamentous hemagglutinin- AAA22974
1965	SQSSGWSVEVASTAS	filamentous hemagglutinin- AAA22974
1966	RSSLLTAATRLGDSV	filamentous hemagglutinin- AAA22974
1967	QNVEDGREIRGELMA	filamentous hemagglutinin- AAA22974
1968	QVAAEATQLVTADTA	filamentous hemagglutinin- AAA22974
1969	VALSAGISADFDSSH	filamentous hemagglutinin- AAA22974
1970	RSTSQNTQYLGGNLS	filamentous hemagglutinin- AAA22974

1971	TLNQDAAQATVVQRN	filamentous hemagglutinin- AAA22974
1972	HWAGGGSEFSVAGKS	filamentous hemagglutinin- AAA22974
1973	KKKNQVRPVETPTPD	filamentous hemagglutinin- AAA22974
1974	VDGPPSRPTTPPASP	filamentous hemagglutinin- AAA22974
1975	PIRATVEVSSPPPVS	filamentous hemagglutinin- AAA22974
1976	ATVEVVPRPKVETAQ	filamentous hemagglutinin- AAA22974
1977	TCVIEDPSGPNHTKV	Fim2 pilic subunit- CAD12823.1 Q8VVA0
1978	QLPKISKNALKANGD	Fim2 pilic subunit- CAD12823.1 Q8VVA0
1979	AGRTPFIIKLKDCPS	Fim2 pilic subunit- CAD12823.1 Q8VVA0
1980	LGNGVKAYFEPGPTT	Fim2 pilic subunit- CAD12823.1 Q8VVA0
1981	YSTGDLRAYKMVYAT	Fim2 pilic subunit- CAD12823.1 Q8VVA0
1982	PQTQLSNITAATEAQ	Fim2 pilic subunit- CAD12823.1 Q8VVA0
1983	SPPSAWALKLPSLLT	tracheal colon factor- CAA08832. O86135
1984	DELKLVLPTGMSLED	tracheal colon factor- CAA08832. O86135
1985	KRSLQESAPSALATP	tracheal colon factor- CAA08832. O86135
1986	SSSPPVAKPGPGSVA	tracheal colon factor- CAA08832. O86135
1987	APSGSGHKDNPSPPV	tracheal colon factor- CAA08832. O86135
1988	GVGPGMAESSGGHNP	tracheal colon factor- CAA08832. O86135
1989	DTALPVADGRWHVGA	tracheal colon factor- CAA08832. O86135
1990	AGYTNGRIKFDRGGT	tracheal colon factor- CAA08832. O86135
1991	DDDSVHVGAYATYIE	tracheal colon factor- CAA08832. O86135
1992	GGFYMDGIVRVSRIR	tracheal colon factor- CAA08832. O86135
1993	AFKVDDAKGRRVRGQ	tracheal colon factor- CAA08832. O86135
1994	RGNGVGASLELGKRF	tracheal colon factor- CAA08832. O86135
1995	RKGGDDFEAVKVIGN	Bif. hemolysin-adenylate cyclaseprec- P15318
1996	AGIPLTADIDMFAIM	Bif. hemolysin-adenylate cyclaseprec- P15318
1997	HLSNFRDSARSSVTS	Bif. hemolysin-adenylate cyclaseprec- P15318
1998	DSVTDYLARTRRAAS	Bif. hemolysin-adenylate cyclaseprec- P15318
1999	ATGGLDRERIDLLWK	Bif. hemolysin-adenylate cyclaseprec- P15318
2000	ARAGARSAVGTEARR	Bif. hemolysin-adenylate cyclaseprec- P15318
2001	VGAGMSLTDDAPAGQ	Bif. hemolysin-adenylate cyclaseprec- P15318
2002	AAAGAEIALQLTGGT	Bif. hemolysin-adenylate cyclaseprec- P15318
2003	ELASSIALALAAARG	Bif. hemolysin-adenylate cyclaseprec- P15318
2004	TSGLQVAGASAGAAA	Bif. hemolysin-adenylate cyclaseprec- P15318
2005	ALAAALSPMEIYGLV	Bif. hemolysin-adenylate cyclaseprec- P15318
2006	QSHYADQLDKLAQES	Bif. hemolysin-adenylate cyclaseprec- P15318
2007	AGTNTVSYAALGRQD	Bif. hemolysin-adenylate cyclaseprec- P15318
2008	ITVSADGERFNVRKQ	Bif. hemolysin-adenylate cyclaseprec- P15318
2009	NNANVYREGVATQTT	Bif. hemolysin-adenylate cyclaseprec- P15318
2010	YGKRTENVQYRHVEL	Bif. hemolysin-adenylate cyclaseprec- P15318
2011	RVGQLVEVDTLEHVQ	Bif. hemolysin-adenylate cyclaseprec- P15318
2012	IIGGAGNDSITGNAH	Bif. hemolysin-adenylate cyclaseprec- P15318
2013	GLGDDTLEGGAGNDW	Bif. hemolysin-adenylate cyclaseprec- P15318
2014	GQTQAREHDVLRGGD	Bif. hemolysin-adenylate cyclaseprec- P15318
2015	VDTVDYSQTGAHAGI	Bif. hemolysin-adenylate cyclaseprec- P15318
2016	AGRIGLGILADLGAG	Bif. hemolysin-adenylate cyclaseprec- P15318
2017	VDKLGEAGSSAYDTV	Bif. hemolysin-adenylate cyclaseprec- P15318

2018	GIENVVGTELADRIT	Bif. hemolysin-adenylate cyclaseprec- P15318
2019	LLAAALLAGFAGAAQ	Out. M.porin protein prec-CAA41398.1 Q04064
2020	ETSVTLYGIIDTGIG	Out. M.porin protein prec-CAA41398.1 Q04064
2021	NDVDFKVKGANADDS	Out. M.porin protein prec-CAA41398.1 Q04064
2022	FKYNHSRFGMINGVQ	Out. M.porin protein prec-CAA41398.1 Q04064
2023	GSRWGLRGTEDLGDG	Out. M.porin protein prec-CAA41398.1 Q04064
2024	QAVFQLESGFNSGNG	Out. M.porin protein prec-CAA41398.1 Q04064
2025	GSSSVFWSVAPARAA	Out. M.porin protein OmpQ- CAD12825. Q8VV98
2026	QLELYGVVDVGLATT	Out. M.porin protein OmpQ- CAD12825. Q8VV98
2027	VSGLGTRQQVLGGGQ	Out. M.porin protein OmpQ- CAD12825. Q8VV98
2028	DNLWGLRGTEELDGG	Out. M.porin protein OmpQ- CAD12825. Q8VV98
2029	RASFGLESGFDAANG	Out. M.porin protein OmpQ- CAD12825. Q8VV98
2030	RNDDARLFDYGTWVG	Out. M.porin protein OmpQ- CAD12825. Q8VV98
2031	RVMDSNDIEKERGIT	GTP-binding elongation factor- Q7VYR0
2032	LAKNCAVEYEGTHIN	GTP-binding elongation factor- Q7VYR0
2033	VDTPGHADFGGEVER	GTP-binding elongation factor- Q7VYR0
2034	LSMVDGVLLLVDAVE	GTP-binding elongation factor- Q7VYR0
2035	PMPQTIFVTRKALAL	GTP-binding elongation factor- Q7VYR0
2036	LKPIVVVNKIDRPGA	GTP-binding elongation factor- Q7VYR0
2037	GDLQDMQPDGRGRTR	GTP-binding elongation factor- Q7VYR0
2038	EYIIPARGLIGFQNE	GTP-binding elongation factor- Q7VYR0
2039	LTLTRGTGLMSHIFH	GTP-binding elongation factor- Q7VYR0
2040	YAPLREGSIGERRNG	GTP-binding elongation factor- Q7VYR0
2041	LISQDNGDAVAYALW	GTP-binding elongation factor- Q7VYR0
2042	LQDRGRMFVSPGEPL	GTP-binding elongation factor- Q7VYR0
2043	GPALIADGGSISVAG	Bordetella resistance to killing- AAA51646
2044	SIDMDMGPGFPPPPP	Bordetella resistance to killing- AAA51646
2045	LPGAPLAAHPPLDRV	Bordetella resistance to killing- AAA51646
2046	AVHAGQDGKVTLREV	Bordetella resistance to killing- AAA51646
2047	LRAHGPQATGVYAYM	Bordetella resistance to killing- AAA51646
2048	GSEITLQGGTVSVQG	Bordetella resistance to killing- AAA51646
2049	APAPEASYKTLTLQT	Bordetella resistance to killing- AAA51646
2050	DGNGVFVLNTNVAAG	Bordetella resistance to killing- AAA51646
2051	NDQLRVTGRADGQHR	Bordetella resistance to killing- AAA51646
2052	LVRNAGGEADSRGAR	Bordetella resistance to killing- AAA51646
2053	GLVHTQGQGNATFRL	Bordetella resistance to killing- AAA51646
2054	NVGKAVDLGTWRYSL	Bordetella resistance to killing- AAA51646
2055	GHNLYASYEYAAGDR	Bordetella resistance to killing- AAA51646
2056	INIPWSFHAGYRYSF	Bordetella resistance to killing- AAA51646
2057	GRHPIHFQISAGAAL	Vag8 protein (Autotr.) CAD12828 Q8VV95
2058	LGLLDVAGAAAVTAA	Vag8 protein (Autotr.) CAD12828 Q8VV95
2059	RIDGGAAFLGDVAIA	Vag8 protein (Autotr.) CAD12828 Q8VV95
2060	TKASEHGINVTGRTA	Vag8 protein (Autotr.) CAD12828 Q8VV95
2061	LTVDGSVVHGHGAAG	Vag8 protein (Autotr.) CAD12828 Q8VV95
2062	EVDGESNVSLLNGAR	Vag8 protein (Autotr.) CAD12828 Q8VV95
2063	SSDQPTAIRLIDPRS	Vag8 protein (Autotr.) CAD12828 Q8VV95
2064	LNLDIKDRAQLLGDI	Vag8 protein (Autotr.) CAD12828 Q8VV95

2065	PEAQQPDGSPEQARV	Vag8 protein (Autotr.) CAD12828 Q8VV95
2066	VALADGGTWAGRTDG	Vag8 protein (Autotr.) CAD12828 Q8VV95
2067	GFDIRTTDLKRVDAK	Vag8 protein (Autotr.) CAD12828 Q8VV95
2068	RSHGLGALLRGGRRI	Vag8 protein (Autotr.) CAD12828 Q8VV95
2069	IDGGWYVEPQASVAW	Vag8 protein (Autotr.) CAD12828 Q8VV95
2070	HAGGSRYEASNGLRV	Vag8 protein (Autotr.) CAD12828 Q8VV95
2071	ADGAHSWVLRAGAEA	Vag8 protein (Autotr.) CAD12828 Q8VV95
2072	RQMRLANGNIVEPYA	Vag8 protein (Autotr.) CAD12828 Q8VV95
2073	RGDIVTLPPSAPPDS	putative autotransporter- AAC31207
2074	EQPDAEPEPDAELEP	putative autotransporter- AAC31207
2075	AAAQSDAKANARVMA	putative autotransporter- AAC31207
2076	VDGGEPVAVPIPAPS	putative autotransporter- AAC31207
2077	PDAPIDVFIDSGAQW	putative autotransporter- AAC31207
2078	GMTKTVNALRIEDGT	putative autotransporter- AAC31207
2079	VGATLEAGKRFTLHD	putative autotransporter- AAC31207
2080	WFVEPQSEVSLFHAS	putative autotransporter- AAC31207
2081	GTYRAANNLSVKDEG	putative autotransporter- AAC31207
2082	TSAVLRLGLAAGRRI	putative autotransporter- AAC31207
2083	LGKDRVIQPYATLSW	putative autotransporter- AAC31207
2084	QEFKGVTTVRTNGYG	putative autotransporter- AAC31207
2085	QTARTGWLTWLAILA	Pertussis toxin subunit 1 prec- O69258
2086	TAPVTSPAWADDPPA	Pertussis toxin subunit 1 prec- O69258
2087	VYRYDSRPPEDVFQN	Pertussis toxin subunit 1 prec- O69258
2088	FTAWGNNDNVLDHLT	Pertussis toxin subunit 1 prec- O69258
2089	RSCQVGSSNSAFVST	Pertussis toxin subunit 1 prec- O69258
2090	SSRRYTEVYLEHRMQ	Pertussis toxin subunit 1 prec- O69258
2091	VTATRLLSSTNSRLC	Pertussis toxin subunit 2 prec- P04978
2092	VFVRSGQPVIGACTS	Pertussis toxin subunit 2 prec- P04978
2093	YLIYVAGISVRVHVS	Pertussis toxin subunit 2 prec- P04978
2094	EEQYYDYEDATFETY	Pertussis toxin subunit 2 prec- P04978
2095	ALTGISICNPGSSLC	Pertussis toxin subunit 2 prec- P04978
2096	SPDAHVPFCFGKDLK	Pertussis toxin subunit 4 prec- P0A3R5
2097	PGSSPMEVMLRAVFM	Pertussis toxin subunit 4 prec- P0A3R5
2098	QRPLRMFLGPKQLTF	Pertussis toxin subunit 4 prec- P0A3R5
2099	GKPALELIRMVECSG	Pertussis toxin subunit 4 prec- P0A3R5
2100	AGLPLKANPMHTIAS	Pertussis toxin subunit 5 prec- P04981
2101	LLSVLGIYSPADVAG	Pertussis toxin subunit 5 prec- P04981
2102	VVLRDTNVTAVPASG	P.69A protein (pertactin)- CAA09473
2103	PAAVSVLGASELTLD	P.69A protein (pertactin)- CAA09473
2104	GHITGGRAAGVAAMQ	P.69A protein (pertactin)- CAA09473
2105	AVVHLQRATIRRGDA	P.69A protein (pertactin)- CAA09473
2106	AGGAVPGGAVPGGAV	P.69A protein (pertactin)- CAA09473
2107	GGFGPGGFGPVLDGW	P.69A protein (pertactin)- CAA09473
2108	PPQPPQPQPEAPAPQ	P.69A protein (pertactin)- CAA09473
2109	PAGRELSAAANAAVN	P.69A protein (pertactin)- CAA09473
2110	GGVGLASTLWYAESN	P.69A protein (pertactin)- CAA09473
2111	LSKRLGELRLNPDAG	P.69A protein (pertactin)- CAA09473

2112	AWGRGFAQRQQLDNR	P.69A protein (pertactin)- CAA09473
2113	GRRFDQKVAGFELGA	P.69A protein (pertactin)- CAA09473
2114	THAQGLVPQGQTQVL	filamentous hemagglutinin- AAA22974
2115	GGNKVPVVNIADPNS	filamentous hemagglutinin- AAA22974
2116	GVSHNKFQQFNVANP	filamentous hemagglutinin- AAA22974
2117	VVFNNGLTDGVSRIG	filamentous hemagglutinin- AAA22974
2118	ALTKNPNLTRQASAI	filamentous hemagglutinin- AAA22974
2119	AEVTDTSPSRLAGTL	filamentous hemagglutinin- AAA22974
2120	ASSNALSVRAGGALK	filamentous hemagglutinin- AAA22974
2121	GKLSATGRLDVDGKQ	filamentous hemagglutinin- AAA22974
2122	VTLGSVASDGALSVS	filamentous hemagglutinin- AAA22974
2123	GGNLRANELVSSAQL	filamentous hemagglutinin- AAA22974
2124	VRGQREVALDDASSA	filamentous hemagglutinin- AAA22974
2125	GMTVVAAGALAARNL	filamentous hemagglutinin- AAA22974
2126	DISLGAEGGATLGAV	filamentous hemagglutinin- AAA22974
2127	AAGSIDVRGGSTVAA	filamentous hemagglutinin- AAA22974
2128	SLHANRDVRVSGKDA	filamentous hemagglutinin- AAA22974
2129	RVTAATSGGGLHVSS	filamentous hemagglutinin- AAA22974
2130	RQLDLGAVQARGALA	filamentous hemagglutinin- AAA22974
2131	DGGAGVALQSAKASG	filamentous hemagglutinin- AAA22974
2132	DAALVADGGPIVVEA	filamentous hemagglutinin- AAA22974
2133	ELVSHAGGIGNGRNK	filamentous hemagglutinin- AAA22974
2134	NGASVTVRTTGNLVN	filamentous hemagglutinin- AAA22974
2135	GYISAGKQGVLEVGG	filamentous hemagglutinin- AAA22974
2136	LTNEFLVGSDGTQRI	filamentous hemagglutinin- AAA22974
2137	AQRIENRGTFQSQAP	filamentous hemagglutinin- AAA22974
2138	TLKAPHLRNTGQVVA	filamentous hemagglutinin- AAA22974
2139	HDIHIINSAKLENTG	filamentous hemagglutinin- AAA22974
2140	VDARNDIALDVADFT	filamentous hemagglutinin- AAA22974
2141	TGSLYAEHDATLTLA	filamentous hemagglutinin- AAA22974
2142	GTQRDLVVDQDHILP	filamentous hemagglutinin- AAA22974
2143	AEGTLRVKAKSLTTE	filamentous hemagglutinin- AAA22974
2144	APPMPKAPELDLRGH	filamentous hemagglutinin- AAA22974
2145	LESAEGRKIFGEYKK	filamentous hemagglutinin- AAA22974
2146	QGEYEKAKMAVQAVE	filamentous hemagglutinin- AAA22974
2147	YGEATRRVHDQLGQR	filamentous hemagglutinin- AAA22974
2148	GKALGGMDAETKEVD	filamentous hemagglutinin- AAA22974
2149	IIQEFAADLRTVYAK	filamentous hemagglutinin- AAA22974
2150	QSKFYGSRYFFEQIG	filamentous hemagglutinin- AAA22974
2151	KPDRAARVAGDNYFD	filamentous hemagglutinin- AAA22974
2152	TLVREQVRRALGGYE	filamentous hemagglutinin- AAA22974
2153	RLPVRGVALVAKLMD	filamentous hemagglutinin- AAA22974
2154	AGTVGKALGLKVGVA	filamentous hemagglutinin- AAA22974
2155	TAQQLKQADRDFVWY	filamentous hemagglutinin- AAA22974
2156	SGLEAHAGRGMTAGA	filamentous hemagglutinin- AAA22974
2157	VKVGYRASHEQSSET	filamentous hemagglutinin- AAA22974
2158	KSYRNANLNFGGGSV	filamentous hemagglutinin- AAA22974

2159	AGNVLDIGGADINRN	filamentous hemagglutinin- AAA22974
2160	YGGAAKGNAGTEEAL	filamentous hemagglutinin- AAA22974
2161	MRAKKVESTKYVSEQ	filamentous hemagglutinin- AAA22974
2162	KGDANLTGATIADLS	filamentous hemagglutinin- AAA22974
2163	KGNLKVDGAVNAQNL	filamentous hemagglutinin- AAA22974
2164	DYRDKDGGSGGLNVG	filamentous hemagglutinin- AAA22974
2165	SSTTLAPTVGVAFGR	filamentous hemagglutinin- AAA22974
2166	AGEDYQAEQRATIDV	filamentous hemagglutinin- AAA22974
2167	QTKDPARLQVGGGVK	filamentous hemagglutinin- AAA22974
2168	PKPVTVKLTDENGKP	filamentous hemagglutinin- AAA22974
2169	TYTINRREDLMKLNG	filamentous hemagglutinin- AAA22974
2170	VLSTKTTLGLEQTFR	filamentous hemagglutinin- AAA22974
2171	RSRISAARTTGSSMK	filamentous hemagglutinin- AAA22974
2172	LRLCLRAALAAIASA	Fim2 pilic subunit- CAD12823.1 Q8VVA0
2173	HADDGTIVITGTITD	Fim2 pilic subunit- CAD12823.1 Q8VVA0
2174	KGVEFRLANLNGQHI	Serotype 3 fimbrial subunit-CAA35920 P17835
2175	MGTDKTTQAAQTFTG	Serotype 3 fimbrial subunit-CAA35920 P17835
2176	VTNGSKSYTLRYLAS	Serotype 3 fimbrial subunit-CAA35920 P17835
2177	VKKPKEDVDAAQITS	Serotype 3 fimbrial subunit-CAA35920 P17835
2178	VRALALALLGAGMWT	tracheal colon factor- CAA08832. O86135
2179	APEVPPVAPAPPAGN	tracheal colon factor- CAA08832. O86135
2180	VYDPGTHTLTTPASA	tracheal colon factor- CAA08832. O86135
2181	VSLASSSHGVWQAEM	tracheal colon factor- CAA08832. O86135
2182	ALSKRMGELRLTPVA	tracheal colon factor- CAA08832. O86135
2183	GVWGRAFGRRQDVDN	tracheal colon factor- CAA08832. O86135
2184	VSREFRQTISGFELG	tracheal colon factor- CAA08832. O86135
2185	WGLQAGYIPVNPNLS	Bif. hemolysin-adenylate cyclaseprec- P15318
2186	LFGRAPEVIARADND	Bif. hemolysin-adenylate cyclaseprec- P15318
2187	NSSLAHGHTAVDLTL	Bif. hemolysin-adenylate cyclaseprec- P15318
2188	KERLDYLRQAGLVTG	Bif. hemolysin-adenylate cyclaseprec- P15318
2189	ADGVVASNHAGYEQF	Bif. hemolysin-adenylate cyclaseprec- P15318
2190	FRVKETSDGRYAVQY	Bif. hemolysin-adenylate cyclaseprec- P15318
2191	GASAHWGQRALQGAQ	Bif. hemolysin-adenylate cyclaseprec- P15318
2192	VAAAQRLVHAIALMT	Bif. hemolysin-adenylate cyclaseprec- P15318
2193	FGRAGSTNTPQEAAS	Bif. hemolysin-adenylate cyclaseprec- P15318
2194	SAAVFGLGEASSAVA	Bif. hemolysin-adenylate cyclaseprec- P15318
2195	TVSGFFRGSSRWAGG	Bif. hemolysin-adenylate cyclaseprec- P15318
2196	GVAGGAMALGGGIAA	Bif. hemolysin-adenylate cyclaseprec- P15318
2197	PALTFITPLAAPGEE	Bif. hemolysin-adenylate cyclaseprec- P15318
2198	RRRTKTGKSEFTTFV	Bif. hemolysin-adenylate cyclaseprec- P15318
2199	IVGKQDRWRIRDGAA	Bif. hemolysin-adenylate cyclaseprec- P15318
2200	TTIDLAKVVSQLVDA	Bif. hemolysin-adenylate cyclaseprec- P15318
2201	GVLKHSIKLDVIGGD	Bif. hemolysin-adenylate cyclaseprec- P15318
2202	DDVVLANASRIHYDG	Bif. hemolysin-adenylate cyclaseprec- P15318
2203	IVAPHEYGFGIEADL	Bif. hemolysin-adenylate cyclaseprec- P15318
2204	REWVRKASALGVDYY	Bif. hemolysin-adenylate cyclaseprec- P15318
2205	NVRNVENVIGTSMKD	Bif. hemolysin-adenylate cyclaseprec- P15318

2206	LIGDAQANTLMGQGG	Bif. hemolysin-adenylate cyclaseprec- P15318
2207	DTVRGGDGDDLLFGG	Bif. hemolysin-adenylate cyclaseprec- P15318
2208	GNDMLYGDAGNDTLY	Bif. hemolysin-adenylate cyclaseprec- P15318
2209	GGGHDTIRINAGADQ	Bif. hemolysin-adenylate cyclaseprec- P15318
2210	WFARQGNDLEIRILG	Bif. hemolysin-adenylate cyclaseprec- P15318
2211	DDALTVHDWYRDADH	Bif. hemolysin-adenylate cyclaseprec- P15318
2212	VEIIHAANQAVDQAG	Bif. hemolysin-adenylate cyclaseprec- P15318
2213	EKLVEAMAQYPDPGA	Bif. hemolysin-adenylate cyclaseprec- P15318
2214	AAAPPAARVPDTLMQ	Bif. hemolysin-adenylate cyclaseprec- P15318
2215	FGVNTFADGFKANSY	Out. M.porin protein prec-CAA41398.1 Q04064
2216	VGLSAPIGGASNVFG	Out. M.porin protein prec-CAA41398.1 Q04064
2217	WQMVDPKLTGGDEKM	Out. M.porin protein prec-CAA41398.1 Q04064
2218	VFSLGYTYDLSKRTN	Out. M.porin protein prec-CAA41398.1 Q04064
2219	YAYGSYAKNFAFLED	Out. M.porin protein prec-CAA41398.1 Q04064
2220	DAKSTAVGVGIRHRF	Out. M.porin protein prec-CAA41398.1 Q04064
2221	DWHWANGQQASKAYV	Out. M.porin protein OmpQ- CAD12825. Q8VV98
2222	TLGYRQDLSARTSLY	Out. M.porin protein OmpQ- CAD12825. Q8VV98
2223	YGGYMKGYDPEDPFA	Out. M.porin protein OmpQ- CAD12825. Q8VV98
2224	DVGRATRFGVGMTQR	Out. M.porin protein OmpQ- CAD12825. Q8VV98
2225	AIIAHVDHGKTTLVD	GTP-binding elongation factor- Q7VYR0
2226	LLRQSGTFRENQSVA	GTP-binding elongation factor- Q7VYR0
2227	SRQIRDRLERELKSN	GTP-binding elongation factor- Q7VYR0
2228	ALRVRDTGDDTVFEV	GTP-binding elongation factor- Q7VYR0
2229	GRGELHLTILLENMR	GTP-binding elongation factor- Q7VYR0
2230	EGYELAVSRPRVVFK	GTP-binding elongation factor- Q7VYR0
2231	IDGVKCEPFEALTVD	GTP-binding elongation factor- Q7VYR0
2232	EDAHQGGVMEELGRR	GTP-binding elongation factor- Q7VYR0
2233	GVRVSGAGRALTLAG	Bordetella resistance to killing- AAA51646
2234	TIDATEGGIPAVVRR	Bordetella resistance to killing- AAA51646
2235	GTLELDGVTVAGGEG	Bordetella resistance to killing- AAA51646
2236	EPMTVSDAGSRLSVR	Bordetella resistance to killing- AAA51646
2237	GVLGGEAPGVGLVRA	Bordetella resistance to killing- AAA51646
2238	QGGQASIIDATLQSI	Bordetella resistance to killing- AAA51646
2239	DGATAQGGNGVFLQQ	Bordetella resistance to killing- AAA51646
2240	STIPVAVALESGALA	Bordetella resistance to killing- AAA51646
2241	GDIVADGNKPLDAGI	Bordetella resistance to killing- AAA51646
2242	LSVASGAAWHGATQV	Bordetella resistance to killing- AAA51646
2243	QSATLGKGGTWVVNA	Bordetella resistance to killing- AAA51646
2244	SRVQDMSMRGGRVEF	Bordetella resistance to killing- AAA51646
2245	QAEVMLWRTSGKRYR	Bordetella resistance to killing- AAA51646
2246	SNGLRVKVDANTATL	Bordetella resistance to killing- AAA51646
2247	RLGLRFGRRIALAGG	Bordetella resistance to killing- AAA51646
2248	IVQPYARLGWTQEFK	Bordetella resistance to killing- AAA51646
2249	TGDVRTNGIGHAGAG	Bordetella resistance to killing- AAA51646
2250	HGRVELGAGVDAALG	Bordetella resistance to killing- AAA51646
2251	MNGGALSTSGANSPA	Vag8 protein (Autotr.) CAD12828 Q8VV95
2252	WLLAGGSAQFRDTVL	Vag8 protein (Autotr.) CAD12828 Q8VV95

2253	TVGEASHGVDVAAHS	Vag8 protein (Autotr.) CAD12828 Q8VV95
2254	VELAHAQVRADGQGA	Vag8 protein (Autotr.) CAD12828 Q8VV95
2255	GLVVTRSSAMVRAGS	Vag8 protein (Autotr.) CAD12828 Q8VV95
2256	VESTGDGAAALLESG	Vag8 protein (Autotr.) CAD12828 Q8VV95
2257	FWARGLSQRQRLDTG	Vag8 protein (Autotr.) CAD12828 Q8VV95
2258	GPWQKQTVSGIELGL	Vag8 protein (Autotr.) CAD12828 Q8VV95
2259	RRVAGGATTAWSVGM	Vag8 protein (Autotr.) CAD12828 Q8VV95
2260	AGYSETRRDGGAYRA	Vag8 protein (Autotr.) CAD12828 Q8VV95
2261	HVHSAHVGAYVSYLN	Vag8 protein (Autotr.) CAD12828 Q8VV95
2262	SGSYVDGVVKYNRFR	Vag8 protein (Autotr.) CAD12828 Q8VV95
2263	DVHGEVAAIALGFNG	putative autotransporter- AAC31207
2264	ANISGGSLSVEDGAV	putative autotransporter- AAC31207
2265	TTLTPDAVEYYYDYA	putative autotransporter- AAC31207
2266	SMEHLPADAPLTPVR	putative autotransporter- AAC31207
2267	TLSDGARASGETLIA	putative autotransporter- AAC31207
2268	GGLLPMTLRLSSGVD	putative autotransporter- AAC31207
2269	FDQKVYGFELGADHA	putative autotransporter- AAC31207
2270	AGQQGRWHVGGLLGY	putative autotransporter- AAC31207
2271	RARRSFIDDGAGHTD	putative autotransporter- AAC31207
2272	AHIGAYAAYVADNGF	putative autotransporter- AAC31207
2273	FDSTLRASRFENDFT	putative autotransporter- AAC31207
2274	TATDAVSVRGKYRAN	putative autotransporter- AAC31207
2275	TQHGGPYGRCANKTR	Pertussis toxin subunit 2 prec- P04978
2276	LTVAELRGSGDLQEY	Pertussis toxin subunit 2 prec- P04978
2277	RHVTRGWSIFALYDG	Pertussis toxin subunit 2 prec- P04978
2278	YLGGEYGGVIKDGTP	Pertussis toxin subunit 2 prec- P04978
2279	GAFDLKTTFCIMTTR	Pertussis toxin subunit 2 prec- P04978
2280	TGQPATDHYYSNVTA	Pertussis toxin subunit 2 prec- P04978
2281	FQTYALTGISLCNPA	Pertussis toxin subunit 3 prec- P04979
2282	TTAPGQGGARRSRVR	Pertussis toxin subunit 4 prec- P0A3R5
2283	LAWLLASGAMTHLSP	Pertussis toxin subunit 4 prec- P0A3R5
2284	LADVPYVLVKTNMVV	Pertussis toxin subunit 4 prec- P0A3R5
2285	SVAMKPYEVTPTRML	Pertussis toxin subunit 4 prec- P0A3R5
2286	CGIAAKLGAAASSPD	Pertussis toxin subunit 4 prec- P0A3R5
2287	FLGTVTVKAGKLVAD	P.69A protein (pertactin)- CAA09473
2288	ATLANVGDTWDDDGI	P.69A protein (pertactin)- CAA09473
2289	LYVAGEQAQASIADS	P.69A protein (pertactin)- CAA09473
2290	LQGAGGVQIERGANV	P.69A protein (pertactin)- CAA09473
2291	VQRSAIVDGGLHIGA	P.69A protein (pertactin)- CAA09473
2292	QSLQPEDLPPSRVVL	P.69A protein (pertactin)- CAA09473
2293	GLSDKLVVMQDASGQ	P.69A protein (pertactin)- CAA09473
2294	RLWVRNSGSEPASAN	P.69A protein (pertactin)- CAA09473
2295	LLLVQTPLGSAATFT	P.69A protein (pertactin)- CAA09473
2296	ANKDGKVDIGTYRYR	P.69A protein (pertactin)- CAA09473
2297	AANGNGQWSLVGAKA	P.69A protein (pertactin)- CAA09473
2298	PAPKPAPQPGPQPPQ	P.69A protein (pertactin)- CAA09473
2299	ALGRGHSLYASYEYS	P.69A protein (pertactin)- CAA09473

2300	VFSHVRGMLVPVSEH	filamentous hemagglutinin- AAA22974
2301	TVGNTFCGRTRGQAR	filamentous hemagglutinin- AAA22974
2302	GARATSLSVAPNALA	filamentous hemagglutinin- AAA22974
2303	ALMLACTGLPLVTHA	filamentous hemagglutinin- AAA22974
2304	GGAVNVAGGGAVKIA	filamentous hemagglutinin- AAA22974
2305	ASSVGNLAVQGGGKV	filamentous hemagglutinin- AAA22974
2306	ATLLNAGGTLLVSGR	filamentous hemagglutinin- AAA22974
2307	AVQLGAASSRQALSV	filamentous hemagglutinin- AAA22974
2308	AGGALKADKLSATRR	filamentous hemagglutinin- AAA22974
2309	DVDGKQAVALGSASS	filamentous hemagglutinin- AAA22974
2310	VSAARAATVAELKSL	filamentous hemagglutinin- AAA22974
2311	NISVTGGERVSVQSV	filamentous hemagglutinin- AAA22974
2312	SASRVAISAHGALDV	filamentous hemagglutinin- AAA22974
2313	KVSAKSGIGLEGWGA	filamentous hemagglutinin- AAA22974
2314	GADSLGSDGAISVSG	filamentous hemagglutinin- AAA22974
2315	DAVRVDQARSLADIS	filamentous hemagglutinin- AAA22974
2316	NGTVTISADSAVLEH	filamentous hemagglutinin- AAA22974
2317	TIESKISQSVLAAKG	filamentous hemagglutinin- AAA22974
2318	KGKPAVSVKVAKKLF	filamentous hemagglutinin- AAA22974
2319	NGTLRAVNDNNETMS	filamentous hemagglutinin- AAA22974
2320	RQIDVVDGRPQITDA	filamentous hemagglutinin- AAA22974
2321	TGEARKDESVVSDAA	filamentous hemagglutinin- AAA22974
2322	SVSNPGTFTAGKDIT	filamentous hemagglutinin- AAA22974
2323	TSRGGFDNEGKMESN	filamentous hemagglutinin- AAA22974
2324	DIVIKTEQFSNGRVL	filamentous hemagglutinin- AAA22974
2325	AKHDLTVTASGQADN	filamentous hemagglutinin- AAA22974
2326	GSLKAGHDFTVQAQR	filamentous hemagglutinin- AAA22974
2327	DNSGTMAAGHDATLK	filamentous hemagglutinin- AAA22974
2328	IGGDVDNRSVVRTVS	filamentous hemagglutinin- AAA22974
2329	MEYFKTPLPVSLTAL	filamentous hemagglutinin- AAA22974
2330	NRAGLSPATWNFQST	filamentous hemagglutinin- AAA22974
2331	ELLDYLLDQNRYEYI	filamentous hemagglutinin- AAA22974
2332	GLYPTYTEWSVNTLK	filamentous hemagglutinin- AAA22974
2333	LDLGYQAKPAPTAPP	filamentous hemagglutinin- AAA22974
2334	LASLASLDAAQGLEV	filamentous hemagglutinin- AAA22974
2335	GRRNAQVADAGLAGP	filamentous hemagglutinin- AAA22974
2336	AVAAPAVGAADVGVE	filamentous hemagglutinin- AAA22974
2337	VTGDQVDQPVVAVGL	filamentous hemagglutinin- AAA22974
2338	QPVATVRVAPPAVAL	filamentous hemagglutinin- AAA22974
2339	RPLFETRIKFIDQSK	filamentous hemagglutinin- AAA22974
2340	LSSTTETNQSAHANH	filamentous hemagglutinin- AAA22974
2341	GTRIEAGTLEGKMQN	filamentous hemagglutinin- AAA22974
2342	EIEGGSVDAAHTDLS	filamentous hemagglutinin- AAA22974
2343	ARDARFKAAADFAHA	filamentous hemagglutinin- AAA22974
2344	HEKDVRQLSLGAKVG	filamentous hemagglutinin- AAA22974
2345	GGYEAGFSLGSESGL	filamentous hemagglutinin- AAA22974
2346	KDLNLSGSRVRGKHV	filamentous hemagglutinin- AAA22974

2347	LDVEGDINATSKQDE	filamentous hemagglutinin- AAA22974
2348	NYNSSGGGWDASAGV	filamentous hemagglutinin- AAA22974
2349	IQNRTLVAPVGSAGF	filamentous hemagglutinin- AAA22974
2350	FNTEHDNSRLTNDGA	filamentous hemagglutinin- AAA22974
2351	GVVASDGLTGHVKGD	filamentous hemagglutinin- AAA22974
2352	PQKATPGPVAEVGKA	filamentous hemagglutinin- AAA22974
2353	VTTVQVQSAPPKPAP	filamentous hemagglutinin- AAA22974
2354	AKQPAPAPKPKPKPK	filamentous hemagglutinin- AAA22974
2355	KAERPKPGKTTPLSG	filamentous hemagglutinin- AAA22974
2356	HVVQQQVQVLQRQAS	filamentous hemagglutinin- AAA22974
2357	INNTKSLPGGKLPKP	filamentous hemagglutinin- AAA22974
2358	CVIEEPSTLNHIKVV	Serotype 3 fimbrial subunit-CAA35920 P17835
2359	LPKISKNALRNDGDT	Serotype 3 fimbrial subunit-CAA35920 P17835
2360	GATPFDIKLKECPQA	Serotype 3 fimbrial subunit-CAA35920 P17835
2361	GALKLYFEPGITTNY	Serotype 3 fimbrial subunit-CAA35920 P17835
2362	TGDLIAYKQTYNASG	Serotype 3 fimbrial subunit-CAA35920 P17835
2363	GNLSTVSSATKAKGV	Serotype 3 fimbrial subunit-CAA35920 P17835
2364	PADGGQDGPPPPRDG	tracheal colon factor- CAA08832. O86135
2365	DADPQPPRDDGNGEQ	tracheal colon factor- CAA08832. O86135
2366	PPKGGGDEGQRPPPA	tracheal colon factor- CAA08832. O86135
2367	GNGGNGGNGNAQLPE	tracheal colon factor- CAA08832. O86135
2368	GDDAGPKPPEGEGGD	tracheal colon factor- CAA08832. O86135
2369	GPQPPQGGGEQDAPE	tracheal colon factor- CAA08832. O86135
2370	SQLGKHGSLFGSYEY	tracheal colon factor- CAA08832. O86135
2371	KGSRQTMPWTFHVGY	tracheal colon factor- CAA08832. O86135
2372	YANAADRESGIPAAV	Bif. hemolysin-adenylate cyclaseprec- P15318
2373	DGIKAVAKEKNATLM	Bif. hemolysin-adenylate cyclaseprec- P15318
2374	RLVNPHSTSLIAEGV	Bif. hemolysin-adenylate cyclaseprec- P15318
2375	TKGLGVHAKSSDWGL	Bif. hemolysin-adenylate cyclaseprec- P15318
2376	GLGAAPGVPSGRSKF	Bif. hemolysin-adenylate cyclaseprec- P15318
2377	PDVLETVPASPGLRR	Bif. hemolysin-adenylate cyclaseprec- P15318
2378	SLGAVERQDSGYDSL	Bif. hemolysin-adenylate cyclaseprec- P15318
2379	GVGSRSFSLGEVSDM	Bif. hemolysin-adenylate cyclaseprec- P15318
2380	AVEAAELEMTRQVLH	Bif. hemolysin-adenylate cyclaseprec- P15318
2381	GARQDDAEPGVSGAS	Bif. hemolysin-adenylate cyclaseprec- P15318
2382	ARHEQLANSDGLRKM	Bif. hemolysin-adenylate cyclaseprec- P15318
2383	ADLQAGWNASSVIGV	Bif. hemolysin-adenylate cyclaseprec- P15318
2384	TTEISKSALELAAIT	Bif. hemolysin-adenylate cyclaseprec- P15318
2385	NADNLKSVDVFVDRF	Bif. hemolysin-adenylate cyclaseprec- P15318
2386	QGERVAGQPVVLDVA	Bif. hemolysin-adenylate cyclaseprec- P15318
2387	GGIDIASRKGERPAL	Bif. hemolysin-adenylate cyclaseprec- P15318
2388	HVKNIENLHGSRLND	Bif. hemolysin-adenylate cyclaseprec- P15318
2389	IAGDDQDNELWGHDG	Bif. hemolysin-adenylate cyclaseprec- P15318
2390	DTIRGRGGDDILRGG	Bif. hemolysin-adenylate cyclaseprec- P15318
2391	GLDTLYGEDGNDIFL	Bif. hemolysin-adenylate cyclaseprec- P15318
2392	DDETVSDDIDGGAGL	Bif. hemolysin-adenylate cyclaseprec- P15318
2393	TVDYSAMIHPGRIVA	Bif. hemolysin-adenylate cyclaseprec- P15318

2394	DEPETSNVLRNIENA	Bif. hemolysin-adenylate cyclaseprec- P15318
2395	GSARDDVLIGDAGAN	Bif. hemolysin-adenylate cyclaseprec- P15318
2396	LNGLAGNDVLSGGAG	Bif. hemolysin-adenylate cyclaseprec- P15318
2397	DVLLGDEGSDLLSGD	Bif. hemolysin-adenylate cyclaseprec- P15318
2398	GNDDLFGGQGDDTYL	Bif. hemolysin-adenylate cyclaseprec- P15318
2399	GVGYGHDTIYESGGG	Bif. hemolysin-adenylate cyclaseprec- P15318
2400	FATADNVRAITTGLR	Out. M.porin protein prec-CAA41398.1 Q04064
2401	VNGPLNVALSYDQLN	Out. M.porin protein prec-CAA41398.1 Q04064
2402	SNNQAQGEVDATPRS	Out. M.porin protein prec-CAA41398.1 Q04064
2403	GLGGSYDFEVVKLAL	Out. M.porin protein prec-CAA41398.1 Q04064
2404	YARTTDGWFGGQGYP	Out. M.porin protein prec-CAA41398.1 Q04064
2405	AVTLPSGDKFGGFGV	Out. M.porin protein prec-CAA41398.1 Q04064
2406	WDKLNLHDTSATGGR	Out. M.porin protein OmpQ- CAD12825. Q8VV98
2407	PQALQAGFTYDFEAL	Out. M.porin protein OmpQ- CAD12825. Q8VV98
2408	MALAWSRQRNGFVGL	Out. M.porin protein OmpQ- CAD12825. Q8VV98
2409	GGGQIGLGPEPFAHG	Out. M.porin protein OmpQ- CAD12825. Q8VV98
2410	AINAWLLGLEVPVHG	Out. M.porin protein OmpQ- CAD12825. Q8VV98
2411	GAWLVQGSMARPDWH	Out. M.porin protein OmpQ- CAD12825. Q8VV98
2412	VAFRFGPDGEVQRGR	GTP-binding elongation factor- Q7VYR0
2413	NQVMKFSGLERVVVD	GTP-binding elongation factor- Q7VYR0
2414	AEAGDIVLVNGIEDL	GTP-binding elongation factor- Q7VYR0
2415	IGSTITDPSTPEGLP	GTP-binding elongation factor- Q7VYR0
2416	LRIDEPTLTMNFMVN	GTP-binding elongation factor- Q7VYR0
2417	SPLAGREGKFVTSRQ	GTP-binding elongation factor- Q7VYR0
2418	LAAALALAGMARLAP	Bordetella resistance to killing- AAA51646
2419	AAQAPQPPVAGAPHA	Bordetella resistance to killing- AAA51646
2420	DAGQEGEFDHRDNTL	Bordetella resistance to killing- AAA51646
2421	AVFDDGVGINLDDDP	Bordetella resistance to killing- AAA51646
2422	ELGETAPPTLKDIHI	Bordetella resistance to killing- AAA51646
2423	VEHKNPMSKPAIGVR	Bordetella resistance to killing- AAA51646
2424	GPASVDMQGGSITTT	Bordetella resistance to killing- AAA51646
2425	NRAAGIALTHGSARL	Bordetella resistance to killing- AAA51646
2426	GVAVRAEGSGSSAAQ	Bordetella resistance to killing- AAA51646
2427	ANGTLVVSAGSLASA	Bordetella resistance to killing- AAA51646
2428	SGAISVTDTPLKLMP	Bordetella resistance to killing- AAA51646
2429	ALASSTVSVRLTDGA	Bordetella resistance to killing- AAA51646
2430	GGLLGYTYADRTYPG	Bordetella resistance to killing- AAA51646
2431	GGGKVKGLHVGGYAA	Bordetella resistance to killing- AAA51646
2432	VGDGGYYLDTVLRLG	Bordetella resistance to killing- AAA51646
2433	YDQQYNIAGTDGGRV	Bordetella resistance to killing- AAA51646
2434	ADYRTSGAAWSLEGG	Bordetella resistance to killing- AAA51646
2435	RFELPNDWFAEPQAE	Bordetella resistance to killing- AAA51646
2436	GQQAPAVVLWQGAQL	Vag8 protein (Autotr.) CAD12828 Q8VV95
2437	AQGLVVQVNGAGVSA	Vag8 protein (Autotr.) CAD12828 Q8VV95
2438	HAQDAGSFTLSGSDI	Vag8 protein (Autotr.) CAD12828 Q8VV95
2439	ARGLEVAGIYVQEGM	Vag8 protein (Autotr.) CAD12828 Q8VV95
2440	GTLTGTRVTTQGDTA	Vag8 protein (Autotr.) CAD12828 Q8VV95

2441	ALQVEDAGTHVSMNG	Vag8 protein (Autotr.) CAD12828 Q8VV95
2442	LIEDEQGHTAFTLGN	Vag8 protein (Autotr.) CAD12828 Q8VV95
2443	GGRVDAGARQYELTA	Vag8 protein (Autotr.) CAD12828 Q8VV95
2444	EAQADKARTWQLTPT	Vag8 protein (Autotr.) CAD12828 Q8VV95
2445	ELSTTATAAVNAMAI	Vag8 protein (Autotr.) CAD12828 Q8VV95
2446	ASQRIWQAEMDVLLR	Vag8 protein (Autotr.) CAD12828 Q8VV95
2447	MSGLHSIGSPGGFWA	Vag8 protein (Autotr.) CAD12828 Q8VV95
2448	VAIENAELLGASGMY	putative autotransporter- AAC31207
2449	TFGAQVDMKGGRILA	putative autotransporter- AAC31207
2450	NTNILGSQGYADGPY	putative autotransporter- AAC31207
2451	GVVVTEDGQVNLEGA	putative autotransporter- AAC31207
2452	VSATGLGAAGLWLLG	putative autotransporter- AAC31207
2453	KDTSPRASLRNTDVH	putative autotransporter- AAC31207
2454	ANRGGVVDAGAFRYR	putative autotransporter- AAC31207
2455	TPDNGVWGLERTSQL	putative autotransporter- AAC31207
2456	AVANAALNTGGVGAA	putative autotransporter- AAC31207
2457	SIWYAEGNALSKRLG	putative autotransporter- AAC31207
2458	LRLDPGAGGFWGRTF	putative autotransporter- AAC31207
2459	QKQQLDNKAGRRFDQ	putative autotransporter- AAC31207
2460	TRANPNPYTSRRSVA	Pertussis toxin subunit 1 prec- O69258
2461	IVGTLVRIAPVIGAC	Pertussis toxin subunit 1 prec- O69258
2462	ARQAESSEAMAAWSE	Pertussis toxin subunit 1 prec- O69258
2463	AGEAMVLVYYESIAY	Pertussis toxin subunit 1 prec- O69258
2464	CHLLSVLPLALLGSH	Pertussis toxin subunit 2 prec- P04978
2465	ARASTPGIVIPPQEQ	Pertussis toxin subunit 2 prec- P04978
2466	TFCITTIYKTGQPAA	Pertussis toxin subunit 3 prec- P04979
2467	HYYSKVTATRLLAST	Pertussis toxin subunit 3 prec- P04979
2468	SRLCAVFVRDGQSVI	Pertussis toxin subunit 3 prec- P04979
2469	ACASPYEGRYRDMYD	Pertussis toxin subunit 3 prec- P04979
2470	LRRLLYMIYMSGLAV	Pertussis toxin subunit 3 prec- P04979
2471	VHVSKEEQYYDYEDA	Pertussis toxin subunit 3 prec- P04979
2472	PLRRTTLAMALGALG	P.69A protein (pertactin)- CAA09473
2473	APAAHADWNNQSIVK	P.69A protein (pertactin)- CAA09473
2474	GERQHGIHIQGSDPG	P.69A protein (pertactin)- CAA09473
2475	VRTASGTTIKVSGRQ	P.69A protein (pertactin)- CAA09473
2476	QGILLENPAAELQFR	P.69A protein (pertactin)- CAA09473
2477	GSVTSSGQLSDDGIR	P.69A protein (pertactin)- CAA09473
2478	IVATELPSIPGTSIG	P.69A protein (pertactin)- CAA09473
2479	LDVALASQARWTGAT	P.69A protein (pertactin)- CAA09473
2480	AVDSLSIDNATWVMT	P.69A protein (pertactin)- CAA09473
2481	NSNVGALRLASDGSV	P.69A protein (pertactin)- CAA09473
2482	FQQPAEAGRFKVLTV	P.69A protein (pertactin)- CAA09473
2483	TLAGSGLFRMNVFAD	P.69A protein (pertactin)- CAA09473
2484	FLEPQAELAVFRAGG	P.69A protein (pertactin)- CAA09473
2485	AYRAANGLRVRDEGG	P.69A protein (pertactin)- CAA09473
2486	SVLGRLGLEVGKRIE	P.69A protein (pertactin)- CAA09473
2487	AGGRQVQPYIKASVL	P.69A protein (pertactin)- CAA09473

2488	EFDGAGTVHTNGIAH	P.69A protein (pertactin)- CAA09473
2489	TELRGTRAELGLGMA	P.69A protein (pertactin)- CAA09473
2490	DHATRRATPIAAGAR	filamentous hemagglutinin- AAA22974
2491	AAAGAYAIDGTAAGA	filamentous hemagglutinin- AAA22974
2492	YGKHITLVSSDSGLG	filamentous hemagglutinin- AAA22974
2493	RQLGSLSSPSAITVS	filamentous hemagglutinin- AAA22974
2494	QGEIALGDATVQRGP	filamentous hemagglutinin- AAA22974
2495	SLKGAGVVSAGKLAS	filamentous hemagglutinin- AAA22974
2496	TVSGGGAVNLGDVQS	filamentous hemagglutinin- AAA22974
2497	GQVRATSAGAMTVRD	filamentous hemagglutinin- AAA22974
2498	AAAADLALQAGDALQ	filamentous hemagglutinin- AAA22974
2499	GFLKSAGAMTVNGRD	filamentous hemagglutinin- AAA22974
2500	VRLDGAHAGGQLRVS	filamentous hemagglutinin- AAA22974
2501	DGQAALGSLAAKGEL	filamentous hemagglutinin- AAA22974
2502	QGVTVDRASASRARI	filamentous hemagglutinin- AAA22974
2503	STGSVGIGALKAGAV	filamentous hemagglutinin- AAA22974
2504	AASPRRARRALRQDF	filamentous hemagglutinin- AAA22974
2505	TPGSVVVRAQGNVTV	filamentous hemagglutinin- AAA22974
2506	RGDPHQGVLAQGDII	filamentous hemagglutinin- AAA22974
2507	DAKGGTLLLRNDALT	filamentous hemagglutinin- AAA22974
2508	AAQVTQRGGAANLTS	filamentous hemagglutinin- AAA22974
2509	HDTRFSNKIRLMGPL	filamentous hemagglutinin- AAA22974
2510	VNAGGPVSNTGNLKV	filamentous hemagglutinin- AAA22974
2511	EGVTVTAASFDNETG	filamentous hemagglutinin- AAA22974
2512	EVMAKSATLTTSGAA	filamentous hemagglutinin- AAA22974
2513	NAGKMQVKEAATIVA	filamentous hemagglutinin- AAA22974
2514	ENTAKLSGEVQRKGV	filamentous hemagglutinin- AAA22974
2515	DVGGGEHGRWSGIGY	filamentous hemagglutinin- AAA22974
2516	NYWLRAGNGKKAGTI	filamentous hemagglutinin- AAA22974
2517	APWYGGDLTAEQSLI	filamentous hemagglutinin- AAA22974
2518	VGKDLYLNAGARKDE	filamentous hemagglutinin- AAA22974
2519	RHLLNEGVIQAGGHG	filamentous hemagglutinin- AAA22974
2520	AAQNRGRPEGLKIGA	filamentous hemagglutinin- AAA22974
2521	SATSVSGSFDALRDV	filamentous hemagglutinin- AAA22974
2522	LEKRLDIDDALAAVL	filamentous hemagglutinin- AAA22974
2523	NPHIFTRIGAAQTSL	filamentous hemagglutinin- AAA22974
2524	DGAAGPALARQARQA	filamentous hemagglutinin- AAA22974
2525	ETDGMVDARGLGSAD	filamentous hemagglutinin- AAA22974
2526	GKLRGKDVRLKADTV	filamentous hemagglutinin- AAA22974
2527	VATSMRYDDKGRLAA	filamentous hemagglutinin- AAA22974
2528	GDGALDAQGGQLHIE	filamentous hemagglutinin- AAA22974
2529	KRLETAGATLKGGKV	filamentous hemagglutinin- AAA22974
2530	LDVDDVKLGGVYEAG	filamentous hemagglutinin- AAA22974
2531	SYENKSSTPLGSLFA	filamentous hemagglutinin- AAA22974
2532	GDATLVGAKFGGGDQ	filamentous hemagglutinin- AAA22974
2533	SLKAAKSVNLMAAES	filamentous hemagglutinin- AAA22974
2534	FESYSESHNFHASAD	filamentous hemagglutinin- AAA22974

2535	NLGANAVQGAVGLGL	filamentous hemagglutinin- AAA22974
2536	AGMGTSHQITNETGK	filamentous hemagglutinin- AAA22974
2537	YAGTSVDAANVSIDA	filamentous hemagglutinin- AAA22974
2538	PVAAQVVPVTPPKVE	filamentous hemagglutinin- AAA22974
2539	AKVEVVPRPKVETAQ	filamentous hemagglutinin- AAA22974
2540	LPPRPVVAEKVTTPA	filamentous hemagglutinin- AAA22974
2541	QPQLAKVETVQPVKP	filamentous hemagglutinin- AAA22974
2542	TTKPLPKPLPVAKVT	filamentous hemagglutinin- AAA22974
2543	APPPVVETAQPLPPV	filamentous hemagglutinin- AAA22974
2544	ISNLNDSKITMGANE	Fim2 pilic subunit- CAD12823.1 Q8VVA0
2545	TQQAAGFDPEVQTGG	Fim2 pilic subunit- CAD12823.1 Q8VVA0
2546	SKTVTMRYLASYVKK	Fim2 pilic subunit- CAD12823.1 Q8VVA0
2547	GDVEASAITTYVGFS	Fim2 pilic subunit- CAD12823.1 Q8VVA0
2548	LRAALILAASPVLPA	Serotype 3 fimbrial subunit-CAA35920 P17835
2549	ANDGTIVITGSISDQ	Serotype 3 fimbrial subunit-CAA35920 P17835
2550	THENGLPGIGKVGGS	tracheal colon factor- CAA08832. O86135
2551	PGPDTSTGSGPDAGM	tracheal colon factor- CAA08832. O86135
2552	SGAGSTSPGASGGAG	tracheal colon factor- CAA08832. O86135
2553	DAMPPSEGERPDSGM	tracheal colon factor- CAA08832. O86135
2554	DSGRGGESSAGGLNP	tracheal colon factor- CAA08832. O86135
2555	GAGKPPREEGEPGSK	tracheal colon factor- CAA08832. O86135
2556	WYVEPQLEVAAFHAQ	tracheal colon factor- CAA08832. O86135
2557	ADYTASNGLRIKDDG	tracheal colon factor- CAA08832. O86135
2558	NSMLGRLGLHVGRQF	tracheal colon factor- CAA08832. O86135
2559	LGDGRVVQPYMKLSW	tracheal colon factor- CAA08832. O86135
2560	QEFDGKGTVRTNDIR	tracheal colon factor- CAA08832. O86135
2561	KVRLDGGRTELAVGV	tracheal colon factor- CAA08832. O86135
2562	GDMNIGVITDFELEV	Bif. hemolysin-adenylate cyclaseprec- P15318
2563	NALNRRAHAVGAQDV	Bif. hemolysin-adenylate cyclaseprec- P15318
2564	QHGTEQNNPFPEADE	Bif. hemolysin-adenylate cyclaseprec- P15318
2565	IFVVSATGESQMLTR	Bif. hemolysin-adenylate cyclaseprec- P15318
2566	QLKEYIGQQRGEGYV	Bif. hemolysin-adenylate cyclaseprec- P15318
2567	YENRAYGVAGKSLFD	Bif. hemolysin-adenylate cyclaseprec- P15318
2568	EGDALLAQLYRDKTA	Bif. hemolysin-adenylate cyclaseprec- P15318
2569	EGAVAGVSAVLSTVG	Bif. hemolysin-adenylate cyclaseprec- P15318
2570	AVSIAAAASVVGAPV	Bif. hemolysin-adenylate cyclaseprec- P15318
2571	VVTSLLTGALNGILR	Bif. hemolysin-adenylate cyclaseprec- P15318
2572	VQQPIIEKLANDYAR	Bif. hemolysin-adenylate cyclaseprec- P15318
2573	IDELGGPQAYFEKNL	Bif. hemolysin-adenylate cyclaseprec- P15318
2574	GGSGDDRLDGGAGND	Bif. hemolysin-adenylate cyclaseprec- P15318
2575	LVGGEGQNTVIGGAG	Bif. hemolysin-adenylate cyclaseprec- P15318
2576	DVFLQDLGVWSNQLD	Bif. hemolysin-adenylate cyclaseprec- P15318
2577	GAGVDTVKYNVHQPS	Bif. hemolysin-adenylate cyclaseprec- P15318
2578	ERLERMGDTGIHADL	Bif. hemolysin-adenylate cyclaseprec- P15318
2579	KGTVEKWPALNLFSV	Bif. hemolysin-adenylate cyclaseprec- P15318
2580	NVLRGAGGADVLAGG	Bif. hemolysin-adenylate cyclaseprec- P15318
2581	GDDVLLGGDGDDQLS	Bif. hemolysin-adenylate cyclaseprec- P15318

2582	DAGRDRLYGEAGDDW	Bif. hemolysin-adenylate cyclaseprec- P15318
2583	FQDAANAGNLLDGGD	Bif. hemolysin-adenylate cyclaseprec- P15318
2584	RDTVDFSGPGRGLDA	Bif. hemolysin-adenylate cyclaseprec- P15318
2585	AKGVFLSLGKGFASL	Bif. hemolysin-adenylate cyclaseprec- P15318
2586	GRLFGRQATIGLQSE	Out. M.porin protein prec-CAA41398.1 Q04064
2587	WGRLDFGRQTNIASK	Out. M.porin protein prec-CAA41398.1 Q04064
2588	FGSIDPFGAGFGQAN	Out. M.porin protein prec-CAA41398.1 Q04064
2589	GMGMSAMNTVRYDNM	Out. M.porin protein prec-CAA41398.1 Q04064
2590	MYQTPSYSGFQFGIG	Out. M.porin protein prec-CAA41398.1 Q04064
2591	SFSANDKDADAVNRV	Out. M.porin protein prec-CAA41398.1 Q04064
2592	VGELSLGRQQSIGLQ	Out. M.porin protein OmpQ- CAD12825. Q8VV98
2593	GGQLEIASWRDMGMG	Out. M.porin protein OmpQ- CAD12825. Q8VV98
2594	LFKASDNYRVNNLVN	Out. M.porin protein OmpQ- CAD12825. Q8VV98
2595	LSPEFSGWQWGVGYA	Out. M.porin protein OmpQ- CAD12825. Q8VV98
2596	DVESGDTGRFDRSPA	Out. M.porin protein OmpQ- CAD12825. Q8VV98
2597	STGLKYEDGPLLAAF	Out. M.porin protein OmpQ- CAD12825. Q8VV98
2598	INATFELFDKLGATE	GTP-binding elongation factor- Q7VYR0
2599	QLDFPVVYASGLSGY	GTP-binding elongation factor- Q7VYR0
2600	GLTDDVRSGDMRPLF	GTP-binding elongation factor- Q7VYR0
2601	AIMKYVPQRDDDANG	GTP-binding elongation factor- Q7VYR0
2602	LQMQIISLDYNSYVG	GTP-binding elongation factor- Q7VYR0
2603	IGVGRINRGRMRGNM	GTP-binding elongation factor- Q7VYR0
2604	IGIHSRDNDLVVNPI	GTP-binding elongation factor- Q7VYR0
2605	GKQLTNVRASGTDEA	GTP-binding elongation factor- Q7VYR0
2606	RLVPPIQMSLEYAVE	GTP-binding elongation factor- Q7VYR0
2607	IDDDELVEITPKSIR	GTP-binding elongation factor- Q7VYR0
2608	RKRYLQEHERRRASR	GTP-binding elongation factor- Q7VYR0
2609	CPSSLQIPRSAWRLH	Bordetella resistance to killing- AAA51646
2610	VVAGAGLLDALPPGG	Bordetella resistance to killing- AAA51646
2611	VRLDGTTVSTDGANT	Bordetella resistance to killing- AAA51646
2612	AVLVRGDAARAEVVN	Bordetella resistance to killing- AAA51646
2613	VLRTAKSLAAGVSAQ	Bordetella resistance to killing- AAA51646
2614	GGRVTLRQTRIETAG	Bordetella resistance to killing- AAA51646
2615	GAEGISVLGFEPQSG	Bordetella resistance to killing- AAA51646
2616	THVWSLQRAGQALSG	Bordetella resistance to killing- AAA51646
2617	ANAAVNAADLSSIAL	Bordetella resistance to killing- AAA51646
2618	ESNALDKRLGELRLR	Bordetella resistance to killing- AAA51646
2619	DAGGPWARTFSERQQ	Bordetella resistance to killing- AAA51646
2620	SNRHARAYDQTVSGL	Bordetella resistance to killing- AAA51646
2621	IGLDRGWSASGGRWY	Bordetella resistance to killing- AAA51646
2622	GGTIRTSGNQAQGLR	Vag8 protein (Autotr.) CAD12828 Q8VV95
2623	GTENAPDNTALGASV	Vag8 protein (Autotr.) CAD12828 Q8VV95
2624	LQNLIIETSGTGALG	Vag8 protein (Autotr.) CAD12828 Q8VV95
2625	SVHEPQGGGGTRLSM	Vag8 protein (Autotr.) CAD12828 Q8VV95
2626	GTTVRTRGDDSFALQ	Vag8 protein (Autotr.) CAD12828 Q8VV95
2627	SGPASATLNDVALET	Vag8 protein (Autotr.) CAD12828 Q8VV95
2628	RLLDRGVWTVTGDSR	Vag8 protein (Autotr.) CAD12828 Q8VV95

2629	AEVKLEGGTLAFAPP	Vag8 protein (Autotr.) CAD12828 Q8VV95
2630	QPKGAFKTLVATQGI	Vag8 protein (Autotr.) CAD12828 Q8VV95
2631	GTGTIVMNAHLPSGT	Vag8 protein (Autotr.) CAD12828 Q8VV95
2632	DVLVAPQGFGDRQVL	Vag8 protein (Autotr.) CAD12828 Q8VV95
2633	VNNTDDGTESGATKV	Vag8 protein (Autotr.) CAD12828 Q8VV95
2634	QELGADNAVYTNGIR	Vag8 protein (Autotr.) CAD12828 Q8VV95
2635	VTRSRGGFAEARVGV	Vag8 protein (Autotr.) CAD12828 Q8VV95
2636	ALLGKRHALYADYEY	Vag8 protein (Autotr.) CAD12828 Q8VV95
2637	KGARFEAPWTLQLGY	Vag8 protein (Autotr.) CAD12828 Q8VV95
2638	DGTSPSIRVQGGVVQ	putative autotransporter- AAC31207
2639	GMGANNVAVVATGSG	putative autotransporter- AAC31207
2640	SSTVNSLHLQAGKVA	putative autotransporter- AAC31207
2641	ATPAESDGEFKHLRV	putative autotransporter- AAC31207
2642	TLSGSGLFEMNASAD	putative autotransporter- AAC31207
2643	SDGDLLVVSDEASGQ	putative autotransporter- AAC31207
2644	KVLVRGAGTEPTGVE	putative autotransporter- AAC31207
2645	LTLVELPEGSQTKFT	putative autotransporter- AAC31207
2646	SGGRAELALGLAAAL	putative autotransporter- AAC31207
2647	RGHQLYTSYEYAKGN	putative autotransporter- AAC31207
2648	LTLPWTFHLGYRYTW	putative autotransporter- AAC31207
2649	ERAGRGTGHFIGYIY	Pertussis toxin subunit 1 prec- O69258
2650	VRADNNFYGAASSYF	Pertussis toxin subunit 1 prec- O69258
2651	YVDTYGDNAGRILAG	Pertussis toxin subunit 1 prec- O69258
2652	LATYQSEYLAHRRIP	Pertussis toxin subunit 1 prec- O69258
2653	ENIRRVTRVYHNGIT	Pertussis toxin subunit 1 prec- O69258
2654	ETTTTEYSNARYVSQ	Pertussis toxin subunit 1 prec- O69258
2655	VLALLGMRTAQAVAP	Pertussis toxin subunit 3 prec- P04979
2656	IVIPPKALFTQQGGA	Pertussis toxin subunit 3 prec- P04979
2657	GRCPNGTRALTVAEL	Pertussis toxin subunit 3 prec- P04979
2658	GNAELQTYLRQITPG	Pertussis toxin subunit 3 prec- P04979
2659	SIYGLYDGTYLGQAY	Pertussis toxin subunit 3 prec- P04979
2660	GIIKDAPPGAGFIYR	Pertussis toxin subunit 3 prec- P04979
2661	YKNFTVQELALKLKG	Pertussis toxin subunit 5 prec- P04981
2662	NQEFCLTAFMSGRSL	Pertussis toxin subunit 5 prec- P04981
2663	RACLSDAGHEHDTWF	Pertussis toxin subunit 5 prec- P04981
2664	TMLGFAISAYALKSR	Pertussis toxin subunit 5 prec- P04981
2665	ALTVEDSPYPGTPGD	Pertussis toxin subunit 5 prec- P04981
2666	DLLELQICPLNGYCE	Pertussis toxin subunit 5 prec- P04981
2667	SGSSVELAQSIVEAP	P.69A protein (pertactin)- CAA09473
2668	LGAAIRVGRGARVTV	P.69A protein (pertactin)- CAA09473
2669	GGSLSAPHGNVIETG	P.69A protein (pertactin)- CAA09473
2670	ARRFAPQAAPLSITL	P.69A protein (pertactin)- CAA09473
2671	AGAHAQGKALLYRVL	P.69A protein (pertactin)- CAA09473
2672	EPVKLTLTGGADAQG	P.69A protein (pertactin)- CAA09473
2673	VAGGRWHLGGLAGYT	P.69A protein (pertactin)- CAA09473
2674	GDRGFTGDGGGHTDS	P.69A protein (pertactin)- CAA09473
2675	HVGGYATYIADSGFY	P.69A protein (pertactin)- CAA09473

2676	DATLRASRLENDFKV	P.69A protein (pertactin)- CAA09473
2677	GSDGYAVKGKYRTHG	P.69A protein (pertactin)- CAA09473
2678	GASLEAGRRFTHADG	P.69A protein (pertactin)- CAA09473
2679	GADLIIANPNGISVN	filamentous hemagglutinin- AAA22974
2680	LSTLNASNLTLTTGR	filamentous hemagglutinin- AAA22974
2681	SVNGGRIGLDVQQGT	filamentous hemagglutinin- AAA22974
2682	TIERGGVNATGLGYF	filamentous hemagglutinin- AAA22974
2683	VVARLVKLQGAVSSK	filamentous hemagglutinin- AAA22974
2684	GKPLADIAVVAGANR	filamentous hemagglutinin- AAA22974
2685	IGVQGGEAVSVANAN	filamentous hemagglutinin- AAA22974
2686	DAELRVRGRGQVDLH	filamentous hemagglutinin- AAA22974
2687	LSAARGADISGEGRV	filamentous hemagglutinin- AAA22974
2688	IGRARSDSDVKVSAH	filamentous hemagglutinin- AAA22974
2689	ALSIDSMTALGAIGV	filamentous hemagglutinin- AAA22974
2690	AGGSVSAKDMRSRGA	filamentous hemagglutinin- AAA22974
2691	GGEHLDLGTLAAVGA	filamentous hemagglutinin- AAA22974
2692	DVNGTGDVRVAKLVS	filamentous hemagglutinin- AAA22974
2693	AGADLQAGRSMTLGI	filamentous hemagglutinin- AAA22974
2694	DTTGDLQARAQQKLE	filamentous hemagglutinin- AAA22974
2695	GSVKSDGGLQAAAGG	filamentous hemagglutinin- AAA22974
2696	LSLAAAEVAGALELS	filamentous hemagglutinin- AAA22974
2697	ALVVKAAEAIVHDGV	filamentous hemagglutinin- AAA22974
2698	ATKGEMQIAGKGGGS	filamentous hemagglutinin- AAA22974
2699	TVTAGAKATTSANKL	filamentous hemagglutinin- AAA22974
2700	VDVASWDNAGSLDIK	filamentous hemagglutinin- AAA22974
2701	GGAQVTVAGRYAEHG	filamentous hemagglutinin- AAA22974
2702	VSIQGDYTVSADAIA	filamentous hemagglutinin- AAA22974
2703	PGSLIAEVQENIDNK	filamentous hemagglutinin- AAA22974
2704	AIVVGKDLTLSSAHG	filamentous hemagglutinin- AAA22974
2705	VANEANALLWAAGEL	filamentous hemagglutinin- AAA22974
2706	VKAQNITNKRAALIE	filamentous hemagglutinin- AAA22974
2707	GGNARLTAAVALLNK	filamentous hemagglutinin- AAA22974
2708	GRIRAGEDMHLDAPR	filamentous hemagglutinin- AAA22974
2709	TIDAETDKVAQRYKS	filamentous hemagglutinin- AAA22974
2710	IDAVRLQAIQPGRVT	filamentous hemagglutinin- AAA22974
2711	AKALSAALGADWRAL	filamentous hemagglutinin- AAA22974
2712	HSQLMQRWKDFKAGK	filamentous hemagglutinin- AAA22974
2713	GAEIAFYPKEQTVLA	filamentous hemagglutinin- AAA22974
2714	GAGLTLSNGAIHNGE	filamentous hemagglutinin- AAA22974
2715	DGQKVLAPRLYLTEA	filamentous hemagglutinin- AAA22974
2716	RQGITDQYAGGGALI	filamentous hemagglutinin- AAA22974
2717	SGGDVTVNTDGHDVS	filamentous hemagglutinin- AAA22974
2718	VNGLIQGRSVKVDAG	filamentous hemagglutinin- AAA22974
2719	GKVVVADSKGAGGGI	filamentous hemagglutinin- AAA22974
2720	ADDEVDVSGRDIGIE	filamentous hemagglutinin- AAA22974
2721	GWSVEVASTASARSS	filamentous hemagglutinin- AAA22974
2722	LTAATRLGDSVAQNV	filamentous hemagglutinin- AAA22974

2723	DGREIRGELMAAQVA	filamentous hemagglutinin- AAA22974
2724	EATQLVTADTAAVAL	filamentous hemagglutinin- AAA22974
2725	AGISADFDSSHSRST	filamentous hemagglutinin- AAA22974
2726	QNTQYLGGNLSIEAT	filamentous hemagglutinin- AAA22974
2727	DAAQATVVQRNKHWA	filamentous hemagglutinin- AAA22974
2728	GGSEFSVAGKSLKKK	filamentous hemagglutinin- AAA22974
2729	QVRPVETPTPDVVDG	filamentous hemagglutinin- AAA22974
2730	PSRPTTPPASPQPIR	filamentous hemagglutinin- AAA22974
2731	TVEVSSPPPVSVATV	filamentous hemagglutinin- AAA22974
2732	EDPSGPNHTKVVQLP	Fim2 pilic subunit- CAD12823.1 Q8VVA0
2733	ISKNALKANGDQAGR	Fim2 pilic subunit- CAD12823.1 Q8VVA0
2734	PFIIKLKDCPSSLGN	Fim2 pilic subunit- CAD12823.1 Q8VVA0
2735	VKAYFEPGPTTDYST	Fim2 pilic subunit- CAD12823.1 Q8VVA0
2736	DLRAYKMVYATNPQT	Fim2 pilic subunit- CAD12823.1 Q8VVA0
2737	LSNITAATEAQGVQV	Fim2 pilic subunit- CAD12823.1 Q8VVA0
2738	AWALKLPSLLTDDEL	tracheal colon factor- CAA08832. O86135
2739	LVLPTGMSLEDFKRS	tracheal colon factor- CAA08832. O86135
2740	QESAPSALATPPSSS	tracheal colon factor- CAA08832. O86135
2741	PVAKPGPGSVAEAPS	tracheal colon factor- CAA08832. O86135
2742	SGHKDNPSPPVVGVG	tracheal colon factor- CAA08832. O86135
2743	GMAESSGGHNPGVGG	tracheal colon factor- CAA08832. O86135
2744	PVADGRWHVGAVAGY	tracheal colon factor- CAA08832. O86135
2745	NGRIKFDRGGTGDDD	tracheal colon factor- CAA08832. O86135
2746	VHVGAYATYIEDGGF	tracheal colon factor- CAA08832. O86135
2747	MDGIVRVSRIRHAFK	tracheal colon factor- CAA08832. O86135
2748	DDAKGRRVRGQYRGN	tracheal colon factor- CAA08832. O86135
2749	VGASLELGKRFTWPG	tracheal colon factor- CAA08832. O86135
2750	DDFEAVKVIGNAAGI	Bif. hemolysin-adenylate cyclaseprec- P15318
2751	LTADIDMFAIMPHLS	Bif. hemolysin-adenylate cyclaseprec- P15318
2752	FRDSARSSVTSGDSV	Bif. hemolysin-adenylate cyclaseprec- P15318
2753	DYLARTRRAASEATG	Bif. hemolysin-adenylate cyclaseprec- P15318
2754	LDRERIDLLWKIARA	Bif. hemolysin-adenylate cyclaseprec- P15318
2755	ARSAVGTEARRQFRY	Bif. hemolysin-adenylate cyclaseprec- P15318
2756	MSLTDDAPAGQKAAA	Bif. hemolysin-adenylate cyclaseprec- P15318
2757	AEIALQLTGGTVELA	Bif. hemolysin-adenylate cyclaseprec- P15318
2758	SIALALAAARGVTSG	Bif. hemolysin-adenylate cyclaseprec- P15318
2759	QVAGASAGAAAGALA	Bif. hemolysin-adenylate cyclaseprec- P15318
2760	ALSPMEIYGLVQQSH	Bif. hemolysin-adenylate cyclaseprec- P15318
2761	ADQLDKLAQESSAYG	Bif. hemolysin-adenylate cyclaseprec- P15318
2762	TVSYAALGRQDSITV	Bif. hemolysin-adenylate cyclaseprec- P15318
2763	ADGERFNVRKQLNNA	Bif. hemolysin-adenylate cyclaseprec- P15318
2764	VYREGVATQTTAYGK	Bif. hemolysin-adenylate cyclaseprec- P15318
2765	TENVQYRHVELARVG	Bif. hemolysin-adenylate cyclaseprec- P15318
2766	LVEVDTLEHVQHIIG	Bif. hemolysin-adenylate cyclaseprec- P15318
2767	AGNDSITGNAHDNFL	Bif. hemolysin-adenylate cyclaseprec- P15318
2768	DTLEGGAGNDWFGQT	Bif. hemolysin-adenylate cyclaseprec- P15318
2769	AREHDVLRGGDGVDT	Bif. hemolysin-adenylate cyclaseprec- P15318

2770	DYSQTGAHAGIAAGR	Bif. hemolysin-adenylate cyclaseprec- P15318
2771	GLGILADLGAGRVDK	Bif. hemolysin-adenylate cyclaseprec- P15318
2772	GEAGSSAYDTVSGIE	Bif. hemolysin-adenylate cyclaseprec- P15318
2773	VVGTELADRITGDAQ	Bif. hemolysin-adenylate cyclaseprec- P15318
2774	ALLAGFAGAAQAETS	Out. M.porin protein prec-CAA41398.1 Q04064
2775	TLYGIIDTGIGYNDV	Out. M.porin protein prec-CAA41398.1 Q04064
2776	FKVKGANADDSDFKY	Out. M.porin protein prec-CAA41398.1 Q04064
2777	HSRFGMINGVQNGSR	Out. M.porin protein prec-CAA41398.1 Q04064
2778	GLRGTEDLGDGLQAV	Out. M.porin protein prec-CAA41398.1 Q04064
2779	QLESGFNSGNGNSAQ	Out. M.porin protein prec-CAA41398.1 Q04064
2780	VFWSVAPARAANQLE	Out. M.porin protein OmpQ- CAD12825. Q8VV98
2781	YGVVDVGLATTRVSG	Out. M.porin protein OmpQ- CAD12825. Q8VV98
2782	GTRQQVLGGGQTDNL	Out. M.porin protein OmpQ- CAD12825. Q8VV98
2783	GLRGTEELDGGWRAS	Out. M.porin protein OmpQ- CAD12825. Q8VV98
2784	GLESGFDAANGTRND	Out. M.porin protein OmpQ- CAD12825. Q8VV98
2785	ARLFDYGTWVGLGHA	Out. M.porin protein OmpQ- CAD12825. Q8VV98
2786	SNDIEKERGITILAK	GTP-binding elongation factor- Q7VYR0
2787	CAVEYEGTHINIVDT	GTP-binding elongation factor- Q7VYR0
2788	GHADFGGEVERVLSM	GTP-binding elongation factor- Q7VYR0
2789	DGVLLLVDAVEGPMP	GTP-binding elongation factor- Q7VYR0
2790	TIFVTRKALALGLKP	GTP-binding elongation factor- Q7VYR0
2791	VVVNKIDRPGARPDF	GTP-binding elongation factor- Q7VYR0
2792	DMQPDGRGRTRLEYI	GTP-binding elongation factor- Q7VYR0
2793	PARGLIGFQNEFLTL	GTP-binding elongation factor- Q7VYR0
2794	RGTGLMSHIFHEYAP	GTP-binding elongation factor- Q7VYR0
2795	REGSIGERRNGVLIS	GTP-binding elongation factor- Q7VYR0
2796	DNGDAVAYALWKLQD	GTP-binding elongation factor- Q7VYR0
2797	GRMFVSPGEPLYEGM	GTP-binding elongation factor- Q7VYR0
2798	IADGGSISVAGGSID	Bordetella resistance to killing- AAA51646
2799	DMGPGFPPPPPPLPG	Bordetella resistance to killing- AAA51646
2800	PLAAHPPLDRVAAVH	Bordetella resistance to killing- AAA51646
2801	GQDGKVTLREVALRA	Bordetella resistance to killing- AAA51646
2802	GPQATGVYAYMPGSE	Bordetella resistance to killing- AAA51646
2803	TLQGGTVSVQGDDGA	Bordetella resistance to killing- AAA51646
2804	EASYKTLTLQTLDGN	Bordetella resistance to killing- AAA51646
2805	VFVLNTNVAAGQNDQ	Bordetella resistance to killing- AAA51646
2806	RVTGRADGQHRVLVR	Bordetella resistance to killing- AAA51646
2807	AGGEADSRGARLGLV	Bordetella resistance to killing- AAA51646
2808	TQGQGNATFRLANVG	Bordetella resistance to killing- AAA51646
2809	AVDLGTWRYSLAEDP	Bordetella resistance to killing- AAA51646
2810	YASYEYAAGDRINIP	Bordetella resistance to killing- AAA51646
2811	MAGQARGWYGAGGRH	Vag8 protein (Autotr.) CAD12828 Q8VV95
2812	IHFQISAGAALMLGL	Vag8 protein (Autotr.) CAD12828 Q8VV95
2813	DVAGAAAVTAAQRID	Vag8 protein (Autotr.) CAD12828 Q8VV95
2814	GAAFLGDVAIATTKA	Vag8 protein (Autotr.) CAD12828 Q8VV95
2815	EHGINVTGRTAEVRV	Vag8 protein (Autotr.) CAD12828 Q8VV95
2816	GSVVHGHGAAGLEVD	Vag8 protein (Autotr.) CAD12828 Q8VV95

2817	ESNVSLLNGARLSSD	Vag8 protein (Autotr.) CAD12828 Q8VV95
2818	PTAIRLIDPRSVLNL	Vag8 protein (Autotr.) CAD12828 Q8VV95
2819	IKDRAQLLGDIAPEA	Vag8 protein (Autotr.) CAD12828 Q8VV95
2820	QPDGSPEQARVRVAL	Vag8 protein (Autotr.) CAD12828 Q8VV95
2821	DGGTWAGRTDGAVHT	Vag8 protein (Autotr.) CAD12828 Q8VV95
2822	RTTDLKRVDAKHRSH	Vag8 protein (Autotr.) CAD12828 Q8VV95
2823	LGALLRGGRRIDIDG	Vag8 protein (Autotr.) CAD12828 Q8VV95
2824	WYVEPQASVAWFHAG	Vag8 protein (Autotr.) CAD12828 Q8VV95
2825	SRYEASNGLRVRADG	Vag8 protein (Autotr.) CAD12828 Q8VV95
2826	HSWVLRAGAEAGRQM	Vag8 protein (Autotr.) CAD12828 Q8VV95
2827	LANGNIVEPYARLGW	Vag8 protein (Autotr.) CAD12828 Q8VV95
2828	VTLPPSAPPDSAEQP	putative autotransporter- AAC31207
2829	AEPEPDAELEPDAAA	putative autotransporter- AAC31207
2830	SDAKANARVMAQVDG	putative autotransporter- AAC31207
2831	EPVAVPIPAPSHPDA	putative autotransporter- AAC31207
2832	IDVFIDSGAQWRGMT	putative autotransporter- AAC31207
2833	TVNALRIEDGTWTVT	putative autotransporter- AAC31207
2834	LEAGKRFTLHDGWFV	putative autotransporter- AAC31207
2835	PQSEVSLFHASGGTY	putative autotransporter- AAC31207
2836	AANNLSVKDEGGTSA	putative autotransporter- AAC31207
2837	LRLGLAAGRRIDLGK	putative autotransporter- AAC31207
2838	RVIQPYATLSWLQEF	putative autotransporter- AAC31207
2839	GVTTVRTNGYGLRTD	putative autotransporter- AAC31207
2840	TGWLTWLAILAVTAP	Pertussis toxin subunit 1 prec- O69258
2841	TSPAWADDPPATVYR	Pertussis toxin subunit 1 prec- O69258
2842	DSRPPEDVFQNGFTA	Pertussis toxin subunit 1 prec- O69258
2843	GNNDNVLDHLTGRSC	Pertussis toxin subunit 1 prec- O69258
2844	VGSSNSAFVSTSSSR	Pertussis toxin subunit 1 prec- O69258
2845	YTEVYLEHRMQEAVE	Pertussis toxin subunit 1 prec- O69258
2846	RLLSSTNSRLCAVFV	Pertussis toxin subunit 2 prec- P04978
2847	SGQPVIGACTSPYDG	Pertussis toxin subunit 2 prec- P04978
2848	YWSMYSRLRKMLYLI	Pertussis toxin subunit 2 prec- P04978
2849	VAGISVRVHVSKEEQ	Pertussis toxin subunit 2 prec- P04978
2850	YDYEDATFETYALTG	Pertussis toxin subunit 2 prec- P04978
2851	MLINNKKLLHHILPI	Pertussis toxin subunit 3 prec- P04979
2852	HVPFCFGKDLKRPGS	Pertussis toxin subunit 4 prec- P0A3R5
2853	PMEVMLRAVFMQQRP	Pertussis toxin subunit 4 prec- P0A3R5
2854	RMFLGPKQLTFEGKP	Pertussis toxin subunit 4 prec- P0A3R5
2855	LELIRMVECSGKQDC	Pertussis toxin subunit 4 prec- P0A3R5
2856	LKANPMHTIASILLS	Pertussis toxin subunit 5 prec- P04981
2857	LGIYSPADVAGLPTH	Pertussis toxin subunit 5 prec- P04981
2858	DTNVTAVPASGAPAA	P.69A protein (pertactin)- CAA09473
2859	SVLGASELTLDGGHI	P.69A protein (pertactin)- CAA09473
2860	GGRAAGVAAMQGAVV	P.69A protein (pertactin)- CAA09473
2861	LQRATIRRGDAPAGG	P.69A protein (pertactin)- CAA09473
2862	VPGGAVPGGAVPGGF	P.69A protein (pertactin)- CAA09473
2863	PGGFGPVLDGWYGVD	P.69A protein (pertactin)- CAA09473

2864	PQPQPEAPAPQPPAG	P.69A protein (pertactin)- CAA09473
2865	ELSAAANAAVNTGGV	P.69A protein (pertactin)- CAA09473
2866	LASTLWYAESNALSK	P.69A protein (pertactin)- CAA09473
2867	LGELRLNPDAGGAWG	P.69A protein (pertactin)- CAA09473
2868	GFAQRQQLDNRAGRR	P.69A protein (pertactin)- CAA09473
2869	DQKVAGFELGADHAV	P.69A protein (pertactin)- CAA09473
2870	GLVPQGQTQVLQGGN	filamentous hemagglutinin- AAA22974
2871	VPVVNIADPNSGGVS	filamentous hemagglutinin- AAA22974
2872	NKFQQFNVANPGVVF	filamentous hemagglutinin- AAA22974
2873	NGLTDGVSRIGGALT	filamentous hemagglutinin- AAA22974
2874	NPNLTRQASAILAEV	filamentous hemagglutinin- AAA22974
2875	DTSPSRLAGTLEVYG	filamentous hemagglutinin- AAA22974
2876	ALSVRAGGALKAGKL	filamentous hemagglutinin- AAA22974
2877	ATGRLDVDGKQAVTL	filamentous hemagglutinin- AAA22974
2878	SVASDGALSVSAGGN	filamentous hemagglutinin- AAA22974
2879	RANELVSSAQLEVRG	filamentous hemagglutinin- AAA22974
2880	REVALDDASSARGMT	filamentous hemagglutinin- AAA22974
2881	VAAGALAARNLQSKG	filamentous hemagglutinin- AAA22974
2882	GAEGGATLGAVEAAG	filamentous hemagglutinin- AAA22974
2883	IDVRGGSTVAANSLH	filamentous hemagglutinin- AAA22974
2884	NRDVRVSGKDAVRVT	filamentous hemagglutinin- AAA22974
2885	ATSGGGLHVSSGRQL	filamentous hemagglutinin- AAA22974
2886	LGAVQARGALALDGG	filamentous hemagglutinin- AAA22974
2887	GVALQSAKASGTLHV	filamentous hemagglutinin- AAA22974
2888	VADGGPIVVEAGELV	filamentous hemagglutinin- AAA22974
2889	HAGGIGNGRNKENGA	filamentous hemagglutinin- AAA22974
2890	VTVRTTGNLVNKGYI	filamentous hemagglutinin- AAA22974
2891	AGKQGVLEVGGALTN	filamentous hemagglutinin- AAA22974
2892	FLVGSDGTQRIEAQR	filamentous hemagglutinin- AAA22974
2893	ENRGTFQSQAPAGTA	filamentous hemagglutinin- AAA22974
2894	PHLRNTGQVVAGHDI	filamentous hemagglutinin- AAA22974
2895	IINSAKLENTGRVDA	filamentous hemagglutinin- AAA22974
2896	NDIALDVADFTNTGS	filamentous hemagglutinin- AAA22974
2897	YAEHDATLTLAQGTQ	filamentous hemagglutinin- AAA22974
2898	DLVVDQDHILPVAEG	filamentous hemagglutinin- AAA22974
2899	LRVKAKSLTTEIETG	filamentous hemagglutinin- AAA22974
2900	PKAPELDLRGHTLES	filamentous hemagglutinin- AAA22974
2901	EGRKIFGEYKKLQGE	filamentous hemagglutinin- AAA22974
2902	EKAKMAVQAVEAYGE	filamentous hemagglutinin- AAA22974
2903	TRRVHDQLGQRYGKA	filamentous hemagglutinin- AAA22974
2904	GGMDAETKEVDGIIQ	filamentous hemagglutinin- AAA22974
2905	FAADLRTVYAKQADQ	filamentous hemagglutinin- AAA22974
2906	YGSRYFFEQIGYKPD	filamentous hemagglutinin- AAA22974
2907	AARVAGDNYFDTTLV	filamentous hemagglutinin- AAA22974
2908	EQVRRALGGYESRLP	filamentous hemagglutinin- AAA22974
2909	RGVALVAKLMDSAGT	filamentous hemagglutinin- AAA22974
2910	GKALGLKVGVAPTAQ	filamentous hemagglutinin- AAA22974

2911	LKQADRDFVWYVDTV	filamentous hemagglutinin- AAA22974
2912	AHAGRGMTAGAEVKV	filamentous hemagglutinin- AAA22974
2913	YRASHEQSSETEKSY	filamentous hemagglutinin- AAA22974
2914	NANLNFGGGSVEAGN	filamentous hemagglutinin- AAA22974
2915	LDIGGADINRNRYGG	filamentous hemagglutinin- AAA22974
2916	AKGNAGTEEALRMRA	filamentous hemagglutinin- AAA22974
2917	KVESTKYVSEQTSQS	filamentous hemagglutinin- AAA22974
2918	NLTGATIADLSGKGN	filamentous hemagglutinin- AAA22974
2919	KVDGAVNAQNLKDYR	filamentous hemagglutinin- AAA22974
2920	KDGGSGGLNVGISST	filamentous hemagglutinin- AAA22974
2921	LAPTVGVAFGRVAGE	filamentous hemagglutinin- AAA22974
2922	YQAEQRATIDVGQTK	filamentous hemagglutinin- AAA22974
2923	PARLQVGGGVKGTLN	filamentous hemagglutinin- AAA22974
2924	TVKLTDENGKPQTYT	filamentous hemagglutinin- AAA22974
2925	NRREDLMKLNGKVLS	filamentous hemagglutinin- AAA22974
2926	KTTLGLEQTFRLRSR	filamentous hemagglutinin- AAA22974
2927	SAARTTGSSMKPTNR	filamentous hemagglutinin- AAA22974
2928	LRAALAAIASAAHAD	Fim2 pilic subunit- CAD12823.1 Q8VVA0
2929	GTIVITGTITDTTCV	Fim2 pilic subunit- CAD12823.1 Q8VVA0
2930	FRLANLNGQHIRMGT	Serotype 3 fimbrial subunit-CAA35920 P17835
2931	KTTQAAQTFTGKVTN	Serotype 3 fimbrial subunit-CAA35920 P17835
2932	SKSYTLRYLASYVKK	Serotype 3 fimbrial subunit-CAA35920 P17835
2933	KEDVDAAQITSYVGF	Serotype 3 fimbrial subunit-CAA35920 P17835
2934	GNMNRATPCRGAVRA	tracheal colon factor- CAA08832. O86135
2935	ALALLGAGMWTLSPP	tracheal colon factor- CAA08832. O86135
2936	PPVAPAPPAGNGVYD	tracheal colon factor- CAA08832. O86135
2937	GTHTLTTPASAAVSL	tracheal colon factor- CAA08832. O86135
2938	SSSHGVWQAEMNALS	tracheal colon factor- CAA08832. O86135
2939	RMGELRLTPVAGGVW	tracheal colon factor- CAA08832. O86135
2940	RAFGRRQDVDNRVSR	tracheal colon factor- CAA08832. O86135
2941	FRQTISGFELGADTA	tracheal colon factor- CAA08832. O86135
2942	AGYIPVNPNLSKLFG	Bif. hemolysin-adenylate cyclaseprec- P15318
2943	APEVIARADNDVNSS	Bif. hemolysin-adenylate cyclaseprec- P15318
2944	AHGHTAVDLTLSKER	Bif. hemolysin-adenylate cyclaseprec- P15318
2945	DYLRQAGLVTGMADG	Bif. hemolysin-adenylate cyclaseprec- P15318
2946	VASNHAGYEQFEFRV	Bif. hemolysin-adenylate cyclaseprec- P15318
2947	ETSDGRYAVQYRRKG	Bif. hemolysin-adenylate cyclaseprec- P15318
2948	HWGQRALQGAQAVAA	Bif. hemolysin-adenylate cyclaseprec- P15318
2949	QRLVHAIALMTQFGR	Bif. hemolysin-adenylate cyclaseprec- P15318
2950	GSTNTPQEAASLSAA	Bif. hemolysin-adenylate cyclaseprec- P15318
2951	FGLGEASSAVAETVS	Bif. hemolysin-adenylate cyclaseprec- P15318
2952	FFRGSSRWAGGFGVA	Bif. hemolysin-adenylate cyclaseprec- P15318
2953	GAMALGGGIAAAVGA	Bif. hemolysin-adenylate cyclaseprec- P15318
2954	FITPLAAPGEEQRRR	Bif. hemolysin-adenylate cyclaseprec- P15318
2955	KTGKSEFTTFVEIVG	Bif. hemolysin-adenylate cyclaseprec- P15318
2956	QDRWRIRDGAADTTI	Bif. hemolysin-adenylate cyclaseprec- P15318
2957	LAKVVSQLVDANGVL	Bif. hemolysin-adenylate cyclaseprec- P15318

2958	HSIKLDVIGGDGDDV	Bif. hemolysin-adenylate cyclaseprec- P15318
2959	LANASRIHYDGGAGT	Bif. hemolysin-adenylate cyclaseprec- P15318
2960	HEYGFGIEADLSREW	Bif. hemolysin-adenylate cyclaseprec- P15318
2961	RKASALGVDYYDNVR	Bif. hemolysin-adenylate cyclaseprec- P15318
2962	VENVIGTSMKDVLIG	Bif. hemolysin-adenylate cyclaseprec- P15318
2963	AQANTLMGQGGDDTV	Bif. hemolysin-adenylate cyclaseprec- P15318
2964	GGDGDDLLFGGDGND	Bif. hemolysin-adenylate cyclaseprec- P15318
2965	LYGDAGNDTLYGGLG	Bif. hemolysin-adenylate cyclaseprec- P15318
2966	DTIRINAGADQLWFA	Bif. hemolysin-adenylate cyclaseprec- P15318
2967	QGNDLEIRILGTDDA	Bif. hemolysin-adenylate cyclaseprec- P15318
2968	TVHDWYRDADHRVEI	Bif. hemolysin-adenylate cyclaseprec- P15318
2969	HAANQAVDQAGIEKL	Bif. hemolysin-adenylate cyclaseprec- P15318
2970	EAMAQYPDPGAAAAA	Bif. hemolysin-adenylate cyclaseprec- P15318
2971	PAARVPDTLMQSLAV	Bif. hemolysin-adenylate cyclaseprec- P15318
2972	TFADGFKANSYMVGL	Out. M.porin protein prec-CAA41398.1 Q04064
2973	APIGGASNVFGSWQM	Out. M.porin protein prec-CAA41398.1 Q04064
2974	DPKLTGGDEKMNVFS	Out. M.porin protein prec-CAA41398.1 Q04064
2975	GYTYDLSKRTNLYAY	Out. M.porin protein prec-CAA41398.1 Q04064
2976	SYAKNFAFLEDAKST	Out. M.porin protein prec-CAA41398.1 Q04064
2977	MRRLLVVAAMAAGSS	Out. M.porin protein OmpQ- CAD12825. Q8VV98
2978	ANGQQASKAYVVTLG	Out. M.porin protein OmpQ- CAD12825. Q8VV98
2979	RQDLSARTSLYAYGG	Out. M.porin protein OmpQ- CAD12825. Q8VV98
2980	MKGYDPEDPFASDVG	Out. M.porin protein OmpQ- CAD12825. Q8VV98
2981	VGRATRFGVGMTQRF	Out. M.porin protein OmpQ- CAD12825. Q8VV98
2982	HVDHGKTTLVDQLLR	GTP-binding elongation factor- Q7VYR0
2983	SGTFRENQSVAERVM	GTP-binding elongation factor- Q7VYR0
2984	RDRLERELKSNVALR	GTP-binding elongation factor- Q7VYR0
2985	RDTGDDTVFEVSGRG	GTP-binding elongation factor- Q7VYR0
2986	LHLTILLENMRREGY	GTP-binding elongation factor- Q7VYR0
2987	LAVSRPRVVFKEIDG	GTP-binding elongation factor- Q7VYR0
2988	KCEPFEALTVDVEDA	GTP-binding elongation factor- Q7VYR0
2989	QGGVMEELGRRKGDL	GTP-binding elongation factor- Q7VYR0
2990	SGAGRALTLAGSTID	Bordetella resistance to killing- AAA51646
2991	TEGGIPAVVRRGGTL	Bordetella resistance to killing- AAA51646
2992	LDGVTVAGGEGMEPM	Bordetella resistance to killing- AAA51646
2993	VSDAGSRLSVRGGVL	Bordetella resistance to killing- AAA51646
2994	GEAPGVGLVRAAQGG	Bordetella resistance to killing- AAA51646
2995	ASIIDATLQSILGPA	Bordetella resistance to killing- AAA51646
2996	AQGGNGVFLQQHSTI	Bordetella resistance to killing- AAA51646
2997	VAVALESGALARGDI	Bordetella resistance to killing- AAA51646
2998	ADGNKPLDAGISLSV	Bordetella resistance to killing- AAA51646
2999	SGAAWHGATQVLQSA	Bordetella resistance to killing- AAA51646
3000	LGKGGTWVVNADSRV	Bordetella resistance to killing- AAA51646
3001	DMSMRGGRVEFQAPA	Bordetella resistance to killing- AAA51646
3002	MLWRTSGKRYRASNG	Bordetella resistance to killing- AAA51646
3003	RVKVDANTATLGRLG	Bordetella resistance to killing- AAA51646
3004	RFGRRIALAGGNIVQ	Bordetella resistance to killing- AAA51646

3005	YARLGWTQEFKSTGD	Bordetella resistance to killing- AAA51646
3006	RTNGIGHAGAGRHGR	Bordetella resistance to killing- AAA51646
3007	ELGAGVDAALGKGHN	Bordetella resistance to killing- AAA51646
3008	ALSTSGANSPAAWLL	Vag8 protein (Autotr.) CAD12828 Q8VV95
3009	GGSAQFRDTVLRTVG	Vag8 protein (Autotr.) CAD12828 Q8VV95
3010	ASHGVDVAAHSEVEL	Vag8 protein (Autotr.) CAD12828 Q8VV95
3011	HAQVRADGQGAHGLV	Vag8 protein (Autotr.) CAD12828 Q8VV95
3012	TRSSAMVRAGSLVES	Vag8 protein (Autotr.) CAD12828 Q8VV95
3013	GDGAAALLESGHLTV	Vag8 protein (Autotr.) CAD12828 Q8VV95
3014	GLSQRQRLDTGYGPW	Vag8 protein (Autotr.) CAD12828 Q8VV95
3015	KQTVSGIELGLDRRV	Vag8 protein (Autotr.) CAD12828 Q8VV95
3016	GGATTAWSVGMLAGY	Vag8 protein (Autotr.) CAD12828 Q8VV95
3017	ETRRDGGAYRAGHVH	Vag8 protein (Autotr.) CAD12828 Q8VV95
3018	AHVGAYVSYLNDSGS	Vag8 protein (Autotr.) CAD12828 Q8VV95
3019	VDGVVKYNRFRHGFD	Vag8 protein (Autotr.) CAD12828 Q8VV95
3020	EVAAIALGFNGEANI	putative autotransporter- AAC31207
3021	GGSLSVEDGAVLTTL	putative autotransporter- AAC31207
3022	PDAVEYYYDYALSME	putative autotransporter- AAC31207
3023	LPADAPLTPVRVTLS	putative autotransporter- AAC31207
3024	GARASGETLIAHGGL	putative autotransporter- AAC31207
3025	PMTLRLSSGVDARGD	putative autotransporter- AAC31207
3026	VYGFELGADHAIAGQ	putative autotransporter- AAC31207
3027	GRWHVGGLLGYTRAR	putative autotransporter- AAC31207
3028	SFIDDGAGHTDSAHI	putative autotransporter- AAC31207
3029	AYAAYVADNGFYFDS	putative autotransporter- AAC31207
3030	LRASRFENDFTVTAT	putative autotransporter- AAC31207
3031	AVSVRGKYRANGVGA	putative autotransporter- AAC31207

Table A1b. List of the unique mutant variant peptides printed on the microarray

1	SNARYVSQQTRANPNPYTSR	74	DGWYGVDVSGSSVELAQSIV
2	GTLVRMAPVVGACMARQAES	75	PAGGAVPGGFGPGGFGPGGF
3	VTRVYHNGITGETTTTEYPN	76	DIKLKECPQELGALKLYFEP
4	TTTTEYPNARYVSQQTRANP	77	YKQTYNASGNGNLSTVSSAA
5	SVASIVGTLVRMAPVMGACM	78	LSTVSSAAKAKGVEFRLANL
6	APVMGACMARQAESSEAMAA	79	AGATPFDIKLKECPQELGAL
7	GAVPGGAVPGGAVPGGAVPG	80	CPQELGALKLYFEPGITTNY
8	VTRVYHNGITGETTTTEYSN	81	ASGNGNLSTVSSATKAKGVE
9	TTTTEYSNARYVSQQTRANP	82	SVASIVGTLVRMAPVVGACM
10	SVASIVGTLVRMAPVIGACM	83	APVVGACMARQAESSEAMAA
11	APVIGACMARQAESSEAMAA	84	NGITGETTTTEYPNARYVSQ
12	NGITGETTTTEYSNARYVSQ	85	PNARYVSQQTRANPNPYTSR
13	GSTSPGASGAMPPSEGERPD	86	GTLVRMAPVMGACMARQAES
14	EATQQAAGFDPEVQTGGTSR	87	AVPGGAVPGGAVPGGFGPGG
15	VQTGGTSRTVTMRYLASYVK	88	GTLVRMAPVIGACMARQAES

16	QQAAGFDPEVQTGGTSKTVT	89	ASGAMPPSEGERPDSGMSDS
17	GGTSKTVTMRYLASYVKKNG	90	AGFDPEVQTGGTSRTVTMRY
18	FGPGGFGPGGFGPVLDGWYG	91	SRTVTMRYLASYVKKNGDVE
19	GDAPAGGAVPGGAVPGGFGP	92	DPEVQTGGTSKTVTMRYLAS
20	AVPGGFGPGGFGPGGFGPGG	93	RATIRRGDAPAGGAVPGGAV
21	PGGFGPGGFGPGGFGPVLDG	94	GAVPGGAVPGGFGPGGFGPG
22	GAVPGGAVPGGFGPVLDGWY	95	GPGGFGPGGFGPGGFGPGGF
23	GPVLDGWYGVDVSGSSVELA	96	AVPGGFGPVLDGWYGVDVSG
24	VSSATKAKGVEFRLANLNGQ	97	GDAPAGGAVPGGAVPGGAVP
25	SGAGSTSPGASGGAGKDAMP	98	PDAGMASGAGSTSPGASGGA
26	PDAGMASGAGSTSPGASDGA	99	SPGASGGAGKDAMPPSEGER
27	SPGASDGAGKDAMPPSEGER	100	SGAGSTSPGASDGAGKDAMP
28	GMASGAGSTSPGVSGGAGKD	101	GSGPDAGMASGAGSTSPGVS
29	VSGGAGKDAMPPSEGERPDS	102	GSTSPGVSGGAGKDAMPPSE
30	GPGGFGPGGFGPGGFGPVLD	103	GMASGAGSTSPGASGAMPPS
31	GGFGPVLDGWYGVDVSGSSV	104	PGGFGPGGFGPVLDGWYGVD
32	PGGFGPVLDGWYGVDVSGSS	105	GPGGFGPGGFGPVLDGWYGV
33	AVPGGFGPGGFGPVLDGWYG	106	VLDGWYGVDVSGSSVELAQS
34	PVLDGWYGVDVSGSSVELAQ	107	GPGGFGPVLDGWYGVDVSGS
35	GDAPAGGAVPGGFGPGGFGP	108	RATIRRGDAPAGGAVPGGFG
36	TPFDIKLKECPQELGALKLY	109	GAVPGGFGPGGFGPGGFGPV
37	ELGALKLYFEPGITTNYDTG	110	TYNASGNGNLSTVSSAAKAK
38	NGNLSTVSSAAKAKGVEFRL	111	VSSAAKAKGVEFRLANLNGQ
39	GDTAGATPFDIKLKECPQEL	112	NGNLSTVSSATKAKGVEFRL
40	LKECPQELGALKLYFEPGIT	113	SIVGTLVRMAPVVGACMARQ
41	TYNASGNGNLSTVSSATKAK	114	VGACMARQAESSEAMAAWSE
42	SRRSVASIVGTLVRMAPVVG	115	TGETTTTEYPNARYVSQQTR
43	VRMAPVVGACMARQAESSEA	116	SRRSVASIVGTLVRMAPVMG
44	VYHNGITGETTTTEYPNARY	117	VRMAPVMGACMARQAESSEA
45	TEYPNARYVSQQTRANPNPY	118	GGAVPGGAVPGGFGPGGFGP
46	SIVGTLVRMAPVMGACMARQ	119	SRRSVASIVGTLVRMAPVIG
47	MGACMARQAESSEAMAAWSE	120	VRMAPVIGACMARQAESSEA
48	PGGAVPGGAVPGGAVPGGFG	121	AMPPSEGERPDSGMSDSGRG
49	VYHNGITGETTTTEYSNARY	122	DPEVQTGGTSRTVTMRYLAS
50	TEYSNARYVSQQTRANPNPY	123	EATQQAAGFDPEVQTGGTSK
51	SIVGTLVRMAPVIGACMARQ	124	VQTGGTSKTVTMRYLASYVK
52	IGACMARQAESSEAMAAWSE	125	IRRGDAPAGGAVPGGAVPGG
53	TGETTTTEYSNARYVSQQTR	126	PGGAVPGGFGPGGFGPGGFG
54	SPGASGAMPPSEGERPDSGM	127	GFGPGGFGPGGFGPGGFGPV
55	QQAAGFDPEVQTGGTSRTVT	128	PAGGAVPGGAVPGGFGPVLD
56	GGTSRTVTMRYLASYVKKNG	129	PAGGAVPGGAVPGGAVPGGA
57	AGFDPEVQTGGTSKTVTMRY	130	GMASGAGSTSPGASGGAGKD
58	SKTVTMRYLASYVKKNGDVE	131	ASGGAGKDAMPPSEGERPDS
59	HLQRATIRRGDAPAGGAVPG	132	GSTSPGASDGAGKDAMPPSE
60	PAGGAVPGGAVPGGFGPGGF	133	PDAGMASGAGSTSPGVSGGA

61	GGFGPGGFGPGGFGPGGFGP	134	SPGVSGGAGKDAMPPSEGER
62	PGGAVPGGFGPVLDGWYGVD	135	SGAGSTSPGASGAMPPSEGE
63	LDGWYGVDVSGSSVELAQSI	136	FGPGGFGPVLDGWYGVDVSG
64	ATKAKGVEFRLANLNGQHIR	137	GFGPGGFGPVLDGWYGVDVS
65	GSTSPGASGGAGKDAMPPSE	138	PGGAVPGGFGPGGFGPVLDG
66	GMASGAGSTSPGASDGAGKD	139	GFGPVLDGWYGVDVSGSSVE
67	ASDGAGKDAMPPSEGERPDS	140	IRRGDAPAGGAVPGGFGPGG
68	SGAGSTSPGVSGGAGKDAMP	141	ASGNGNLSTVSSAAKAKGVE
69	PDAGMASGAGSTSPGASGAM	142	AAKAKGVEFRLANLNGQHIR
70	GFGPGGFGPGGFGPVLDGWY	143	YKQTYNASGNGNLSTVSSAT
71	GGFGPGGFGPGGFGPVLDGW	144	LSTVSSATKAKGVEFRLANL
72	FGPVLDGWYGVDVSGSSVEL		
73	GGFGPGGFGPVLDGWYGVDV		

Table S2. Pertussis Differential recognition analysis.

1) Vs. Placebo - SAM, PAM, Cluster on PAM
2) Vs. Infected - SAM, PAM, Cluster on PAM

1) Each group compared to PLACEBO

SAM Analysis
Red: strong IgG response in 10/10 serum samples
Green: weak IgG response in 10/10 serum samples

2c vs Placebo

84 overresponding - 25 underresponding

top 20

	Gene.ID	Score.d.	FC	q.value...	Protein
1	GVGYGHDTIYESGGG	8.25	2.08	0	Bif. hemolysin-adenylate cyclaseprec- P15318_01593
2	AKVEVVPRPKVETAQ	7.99	2.03	0	filamentous hemagglutinin- AAA22974_03353
3	LEKRLDIDDALAAVL	6.01	1.74	0	filamentous hemagglutinin- AAA22974_02217
4	ARASTPGIVIPPQEQ	5.76	2.58	0	Pertussis toxin subunit 2 prec- P04978_00025
5	VSQLVDANGVLKHSI	5.1	1.3	0	Bif. hemolysin-adenylate cyclaseprec- P15318_00893
6	LGDVAIATTKASEHG	5.04	1.3	0	Vag8 protein (Autotr.) CAD12828 Q8VV95_00001
7	LAQGDIIMDAKGGTL	4.86	1.36	0	filamentous hemagglutinin- AAA22974_01105
8	MSLTDDAPAGQKAAA	4.65	1.46	0	Bif. hemolysin-adenylate cyclaseprec- P15318_00553
9	TELAVGVASQLGKHG	4.38	1.61	0	tracheal colon factor- CAA08832. O86135_00001
10	DDFEAVKVIGNAAGI	4.37	1.35	0	Bif. hemolysin-adenylate cyclaseprec- P15318_00169
11	LVAKLMDSAGTVGKA	4.36	1.71	0	filamentous hemagglutinin- AAA22974_02429
12	PGSLIAEVQENIDNK	4.29	1.37	0	filamentous hemagglutinin- AAA22974_01705
13	KVRLDGGRTELAVGV	4.26	1.36	0	tracheal colon factor- CAA08832. O86135_00001
14	ALTVAELRGNAELQT	4.22	1.3	0	Pertussis toxin subunit 3 prec- P04979_00057
15	SASRVAISAHGALDV	4.18	1.21	0	filamentous hemagglutinin- AAA22974_00777
16	ARRSRVRALAWLLAS	4.18	1.26	0	Pertussis toxin subunit 4 prec- P0A3R5_00017
17	SIALALAAARGVTSG	4.14	1.25	0	Bif. hemolysin-adenylate cyclaseprec- P15318_00585
18	LRAALAAIASAAHAD	4.14	1.41	0	Fim2 pilic subunit- CAD12823.1 Q8VVA0_00013
19	PLGSLFAILSSTTET	4.12	1.26	0	filamentous hemagglutinin- AAA22974_02657
20	LTVNTLAGSGLFRMN	4.08	1.24	0	P.69A protein (pertactin)- CAA09473_00473

top 20 Under

	Gene.ID	Score.d.	FC	q.value...	Protein
1	IDVFIDSGAQWRGMT	-5.43	0.53	0	putative autotransporter- AAC31207_00297
2	DRLYGEAGDDWFFQD	-5.27	0.49	0	Bif. hemolysin-adenylate cyclaseprec- P15318_01453
3	LSWLQEFKGVTTVRT	-4.95	0.66	0	putative autotransporter- AAC31207_00689

4	LSVKDEGGTSAVLRL	-4.94	0.71	0	putative autotransporter- AAC31207_00653
5	ASNGLRVRADGAHSW	-4.89	0.67	0	Vag8 protein (Autotr.) CAD12828 Q8VV95_00001
6	DFTVTATDAVSVRGK	-4.82	0.7	0	putative autotransporter- AAC31207_00593
7	AVPGGFGPGGFGPVLDGWYG	-4.81	0.59	0	A13:9
8	DNGDAVAYALWKLQD	-4.63	0.6	0	GTP-binding elongation factor- Q7VYR0_00493
9	LISQDNGDAVAYALW	-4.5	0.65	0	GTP-binding elongation factor- Q7VYR0_00489
10	LEIRILGTDDALTVH	-4.3	0.67	0	Bif. hemolysin-adenylate cyclaseprec- P15318_01629
11	DGGWRASFGLESGFD	-4.24	0.64	0	Out. M.porin protein OmpQ- CAD12825. Q8VV98_00001
12	RVVQPYMKLSWVQEF	-4.22	0.59	0	tracheal colon factor- CAA08832. O86135_00001
13	RFENDFTVTATDAVS	-4.22	0.75	0	putative autotransporter- AAC31207_00589
14	SADLSDGDLLVVSDE	-4.17	0.76	0	putative autotransporter- AAC31207_00373
15	VVTSLLTGALNGILR	-4.01	0.56	0	Bif. hemolysin-adenylate cyclaseprec- P15318_00697
16	EYAAGDRINIPWSFH	-3.82	0.64	0	Bordetella resistance to killing- AAA51646_00989
17	FAEPQAEVMLWRTSG	-3.76	0.65	0	Bordetella resistance to killing- AAA51646_00881
18	TPTNELSTTATAAVN	-3.73	0.72	0	Vag8 protein (Autotr.) CAD12828 Q8VV95_00001
19	WFARQGNDLEIRILG	-3.69	0.72	0	Bif. hemolysin-adenylate cyclaseprec- P15318_01621
20	RASFGLESGFDAANG	-3.62	0.67	0	Out. M.porin protein OmpQ- CAD12825. Q8VV98_00001

PAM

45 predictive peptides

Top20:

	id	1-score	2-score	Protein
1	GVGYGHDTIYESGGG	0.641	-0.641	Bif. hemolysin-adenylate cyclaseprec- P15318_01593
2	AKVEVVPRPKVETAQ	0.6017	-0.6017	filamentous hemagglutinin- AAA22974_03353
3	ARASTPGIVIPPQEQ	0.4354	-0.4354	Pertussis toxin subunit 2 prec- P04978_00025
4	LEKRLDIDDALAAVL	0.3253	-0.3253	filamentous hemagglutinin- AAA22974_02217
5	DRLYGEAGDDWFFQD	-0.3093	0.3093	Bif. hemolysin-adenylate cyclaseprec- P15318_01453

6	IDVFIDSGAQWRGMT	-0.2993	0.2993	putative autotransporter- AAC31207_00297
7	AVPGGFGPGGFGPVLDGWYG	-0.1877	0.1877	A13:9
8	DNGDAVAYALWKLQD	-0.1571	0.1571	GTP-binding elongation factor- Q7VYR0_00493
9	LSWLQEFKGVTTVRT	-0.1449	0.1449	putative autotransporter- AAC31207_00689
10	LVAKLMDSAGTVGKA	0.138	-0.138	filamentous hemagglutinin- AAA22974_02429
11	ASNGLRVRADGAHSW	-0.1294	0.1294	Vag8 protein (Autotr.) CAD12828 Q8VV95_00001
12	GVESLTLVELPEGSQ	0.1285	-0.1285	putative autotransporter- AAC31207_00405
13	RVVQPYMKLSWVQEF	-0.1215	0.1215	tracheal colon factor- CAA08832. O86135_00001
14	TELAVGVASQLGKHG	0.1171	-0.1171	tracheal colon factor- CAA08832. O86135_00001
15	LISQDNGDAVAYALW	-0.1116	0.1116	GTP-binding elongation factor- Q7VYR0_00489
16	VVTSLLTGALNGILR	-0.1094	0.1094	Bif. hemolysin-adenylate cyclaseprec- P15318_00697
17	LSVKDEGGTSAVLRL	-0.0956	0.0956	putative autotransporter- AAC31207_00653
18	MSLTDDAPAGQKAAA	0.0918	-0.0918	Bif. hemolysin-adenylate cyclaseprec- P15318_00553
19	DGGWRASFGLESGFD	-0.0896	0.0896	Out. M.porin protein OmpQ- CAD12825. Q8VV98_00001
20	DFTVTATDAVSVRGK	-0.0884	0.0884	putative autotransporter- AAC31207_00593

PAM cluster on 45 peptides

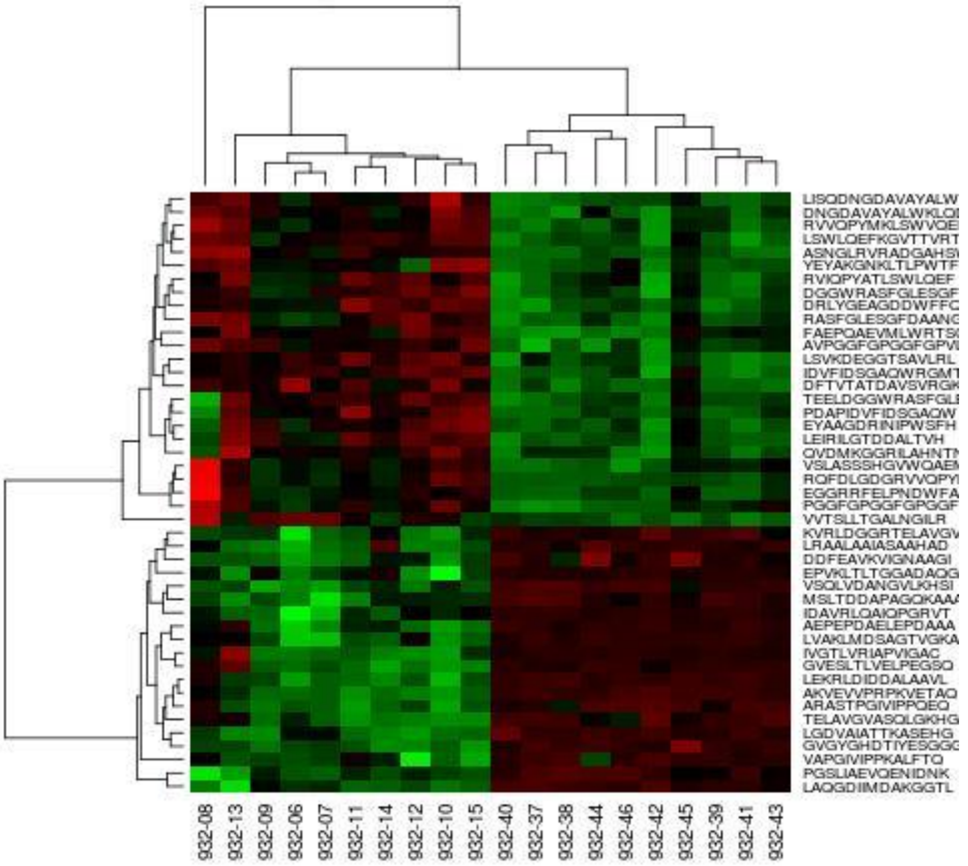

SAM

5c vs Placebo

64 overresponding - 63 underresponding

top 20 Over

	Gene.ID	Score.d.	Fold.Change	q.value...	Protein
1	GVESLTLVELPEGSQ	11.85	2.21	0	putative autotransporter- AAC31207_00405
2	HFIGYIYEVRADNNF	5.07	1.23	0	Pertussis toxin subunit 1 prec- O69258_00117
3	ARASTPGIVIPPQEQ	5.03	1.93	0	Pertussis toxin subunit 2 prec- P04978_00025
4	QGEYEKAKMAVQAVE	4.98	1.18	0	filamentous hemagglutinin- AAA22974_02021
5	LEKRLDIDDALAAVL	4.96	1.26	0	filamentous hemagglutinin- AAA22974_02217

6	VLAAGAGLTLSNGAI	4.87	1.23	0	filamentous hemagglutinin- AAA22974_02165
7	GWSVEVASTASARSS	4.81	1.18	0	filamentous hemagglutinin- AAA22974_02857
8	AKVEVVPRPKVETAQ	4.7	1.46	0	filamentous hemagglutinin- AAA22974_03353
9	GMTVVAAGALAARNL	4.69	1.1	0	filamentous hemagglutinin- AAA22974_00533
10	ALTVAELRGNAELQT	4.63	1.16	0	Pertussis toxin subunit 3 prec- P04979_00057
11	KEQTVLAAGAGLTLS	4.56	1.26	0	filamentous hemagglutinin- AAA22974_02161
12	PGSLIAEVQENIDNK	4.53	1.2	0	filamentous hemagglutinin- AAA22974_01705
13	LGAAIRVGRGARVTV	4.48	1.1	0	P.69A protein (pertactin)- CAA09473_00317
14	GLSDKLVVMQDASGQ	4.37	1.12	0	P.69A protein (pertactin)- CAA09473_00493
15	GRYAEHGEVSIQGDY	4.33	1.15	0	filamentous hemagglutinin- AAA22974_01393
16	QSIVEAPELGAAIRV	4.28	1.22	0	P.69A protein (pertactin)- CAA09473_00309
17	GMAESSGGHNPGVGG	4.19	1.13	0	tracheal colon factor- CAA08832. O86135_00001
18	IGVQGGEAVSVANAN	4.1	1.26	0	filamentous hemagglutinin- AAA22974_00553
19	TGMSLEDFKRSLQES	4.07	1.21	0	tracheal colon factor- CAA08832. O86135_00001
20	GGNARLTAAVALLNK	3.99	1.16	0	filamentous hemagglutinin- AAA22974_01769

top 20 Under

	Gene.ID	Score.d.	FC	q.value...	Protein
1	ISVLGFEPQSGSGPA	-7.28	0.45	0	Bordetella resistance to killing- AAA51646_00397
2	SPDAHVPFCFGKDLK	-6.15	0.57	0	Pertussis toxin subunit 4 prec- P0A3R5_00085
3	ALMLACTGLPLVTHA	-5.38	0.63	0	filamentous hemagglutinin- AAA22974_00057
4	FLSLGKGFASLMDEP	-5.17	0.52	0	Bif. hemolysin-adenylate cyclaseprec- P15318_01501
5	GPGGFGPGGFGPGGFGPVLD	-4.86	0.61	0	A1:9
6	DRLYGEAGDDWFFQD	-4.75	0.55	0	Bif. hemolysin-adenylate cyclaseprec- P15318_01453
7	IDGGWYVEPQASVAW	-4.62	0.57	0	Vag8 protein (Autotr.) CAD12828 Q8VV95_00001
8	GAVPGGAVPGGFGPVLDGWY	-4.38	0.5	0	B17:9
9	ANAGNLLDGGDGRDT	-4.34	0.6	0	Bif. hemolysin-adenylate cyclaseprec- P15318_01469
10	SMEHLPADAPLTPVR	-4.29	0.63	0	putative autotransporter- AAC31207_00181
11	AVPGGFGPGGFGPVLDGWYG	-4.26	0.56	0	A13:9

12	YGGYMKGYDPEDPFA	-4.24	0.46	0	Out. M.porin protein OmpQ- CAD12825. Q8VV98_00001
13	PEPFAHGGAINAWLL	-4.1	0.7	0	Out. M.porin protein OmpQ- CAD12825. Q8VV98_00001
14	WLLGLEVPVHGNGAW	-4.08	0.62	0	Out. M.porin protein OmpQ- CAD12825. Q8VV98_00001
15	PGGFGPVLDGWYGVDVSGSS	-4.06	0.75	0	A9:9
16	EGGRRFELPNDWFAE	-4.02	0.65	0	Bordetella resistance to killing- AAA51646_00869
17	EYYYDYALSMEHLPA	-3.96	0.65	0	putative autotransporter- AAC31207_00173
18	PQALQAGFTYDFEAL	-3.94	0.69	0	Out. M.porin protein OmpQ- CAD12825. Q8VV98_00001
19	NIVEPYARLGWAQEL	-3.93	0.6	0	Vag8 protein (Autotr.) CAD12828 Q8VV95_00001
20	DVFLQDLGVWSNQLD	-3.92	0.67	0	Bif. hemolysin-adenylate cyclaseprec- P15318_01065

PAM
100 predictive peptides.

Top 20:

	id	X1.score	X2.score	Protein
1	GVESLTLVELPEGSQ	1.5	-1.5	putative autotransporter- AAC31207_00405
2	ISVLGFEPQSGSGPA	-0.84	0.84	Bordetella resistance to killing- AAA51646_00397
3	SPDAHVPFCFGKDLK	-0.59	0.59	Pertussis toxin subunit 4 prec- P0A3R5_00085
4	FLSLGKGFASLMDEP	-0.46	0.46	Bif. hemolysin-adenylate cyclaseprec- P15318_01501
5	ARASTPGIVIPPQEQ	0.43	-0.43	Pertussis toxin subunit 2 prec- P04978_00025
6	ALMLACTGLPLVTHA	-0.43	0.43	filamentous hemagglutinin- AAA22974_00057
7	DRLYGEAGDDWFFQD	-0.38	0.38	Bif. hemolysin-adenylate cyclaseprec- P15318_01453
8	GPGGFGPGGFGPGGFGPVLD	-0.37	0.37	A1:9
9	IDGGWYVEPQASVAW	-0.35	0.35	Vag8 protein (Autotr.) CAD12828 Q8VV95_00001
10	GAVPGGAVPGGFGPVLDGWY	-0.33	0.33	B17:9
11	YGGYMKGYDPEDPFA	-0.31	0.31	Out. M.porin protein OmpQ- CAD12825. Q8VV98_00001
12	AKVEVVPRPKVETAQ	0.3	-0.3	filamentous hemagglutinin- AAA22974_03353
13	ANAGNLLDGGDGRDT	-0.29	0.29	Bif. hemolysin-adenylate cyclaseprec- P15318_01469
14	AVPGGFGPGGFGPVLDGWYG	-0.29	0.29	A13:9

15	SMEHLPADAPLTPVR	-0.27	0.27	putative autotransporter- AAC31207_00181
16	WLLGLEVPVHGNGAW	-0.24	0.24	Out. M.porin protein OmpQ- CAD12825. Q8VV98_00001
17	LEKRLDIDDALAAVL	0.24	-0.24	filamentous hemagglutinin- AAA22974_02217
18	FGPGGFGPGGFGPVLDGWYG	-0.22	0.22	B1:9
19	HFIGYIYEVRADNNF	0.22	-0.22	Pertussis toxin subunit 1 prec- O69258_00117
20	NIVEPYARLGWAQEL	-0.22	0.22	Vag8 protein (Autotr.) CAD12828 Q8VV95_00001

PAM cluster - 100 peptides

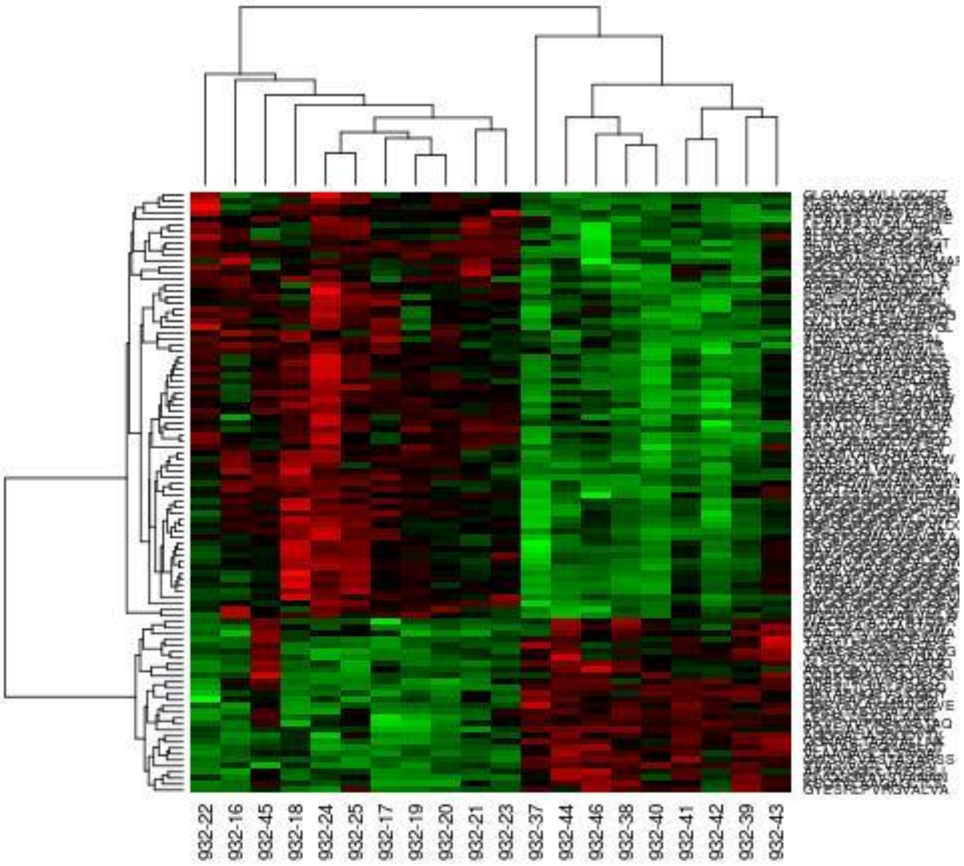

SAM

WH vs Placebo

73 overresponding - 19 underresponding

top 20 Over

	Gene.ID	Score.d.	FC	q.value	Protein
1	ADSTLQGAGGVQIER	8.12	1.77	0	P.69A protein (pertactin)- CAA09473_00153
2	GSAATFTLANKDGKV	5.49	1.47	0	P.69A protein (pertactin)- CAA09473_00533
3	HFIGYIYEVRADNNF	4.97	1.27	0	Pertussis toxin subunit 1 prec- O69258_00117
4	YSRLRKMLYLIYVAG	4.92	1.47	0	Pertussis toxin subunit 2 prec- P04978_00173
5	SVRVHVSKEEQYYDY	4.63	1.45	0	Pertussis toxin subunit 2 prec- P04978_00189
6	GRCPNGTRALTVAEL	4.63	1.29	0	Pertussis toxin subunit 3 prec- P04979_00049
7	MRCTRAIRQTARTGW	4.51	1.35	0	Pertussis toxin subunit 1 prec- O69258_00001
8	LYVAGEQAQASIADS	4.47	1.23	0	P.69A protein (pertactin)- CAA09473_00141
9	PGSLIAEVQENIDNK	4.36	1.23	0	filamentous hemagglutinin- AAA22974_01705
10	AEAGRFKVLTVNTLA	4.29	1.32	0	P.69A protein (pertactin)- CAA09473_00465
11	VLAAGAGLTLSNGAI	4.26	1.22	0	filamentous hemagglutinin- AAA22974_02165
12	GGVGLASTLWYAESN	4.11	1.39	0	P.69A protein (pertactin)- CAA09473_00617
13	AGGGALIASGGDVTV	3.99	1.2	0	filamentous hemagglutinin- AAA22974_02497
14	TGWLTWLAILAVTAP	3.95	1.22	0	Pertussis toxin subunit 1 prec- O69258_00013
15	VFVRSGQPVIGACTS	3.9	1.25	0	Pertussis toxin subunit 2 prec- P04978_00149
16	ARASTPGIVIPPQEQ	3.88	1.48	0	Pertussis toxin subunit 2 prec- P04978_00025
17	GGMDAETKEVDGIIQ	3.86	1.38	0	filamentous hemagglutinin- AAA22974_02057
18	AGNVLDIGGADINRN	3.84	1.22	0	filamentous hemagglutinin- AAA22974_02805
19	LSIDNATWVMTDNSN	3.84	1.33	0	P.69A protein (pertactin)- CAA09473_00433
20	ALTVAELRGNAELQT	3.84	1.16	0	Pertussis toxin subunit 3 prec- P04979_00057

Top 20 Under

Gene.ID	Score.d.	FC	q.value...	Prot

1	IDVFIDSGAQWRGMT	-5.85	0.5	0	putative autotransporter- AAC31207_00297
2	FLSLGKGFASLMDEP	-5.4	0.55	0	Bif. hemolysin-adenylate cyclaseprec- P15318_01501
3	GLGILADLGAGRVDK	-3.98	0.77	0	Bif. hemolysin-adenylate cyclaseprec- P15318_01369
4	IHISVEHKNPMSKPA	-3.63	0.74	0	Bordetella resistance to killing- AAA51646_00101
5	WLLGLEVPVHGNGAW	-3.57	0.66	0	Out. M.porin protein OmpQ- CAD12825. Q8VV98_00001
6	GLGAAGLWLLGDKDT	-3.55	0.71	0	putative autotransporter- AAC31207_00109
7	GASNVFGSWQMVDPK	-3.54	0.59	0	Out. M.porin protein prec-CAA41398.1 Q04064_00001
8	LHDGWFVEPQSEVSL	-3.52	0.69	0	putative autotransporter- AAC31207_00625
9	WPGAWYVEPQLEVAA	-3.42	0.71	0	tracheal colon factor- CAA08832. O86135_00001
10	PDAPIDVFIDSGAQW	-3.42	0.74	0	putative autotransporter- AAC31207_00293
11	GPGGFGPGGFGPVLDGWYGV	-3.26	0.7	0	A7:9
12	LLTDDELKLVLPTGM	-3.26	0.78	0	tracheal colon factor- CAA08832. O86135_00001
13	MSGLHSIGSPGGFWA	-3.25	0.6	0	Vag8 protein (Autotr.) CAD12828 Q8VV95_00001
14	IDGGWYVEPQASVAW	-3.23	0.66	0	Vag8 protein (Autotr.) CAD12828 Q8VV95_00001
15	FRLANVGKAVDLGTW	-3.19	0.87	0	Bordetella resistance to killing- AAA51646_00673
16	PNDWFAEPQAEVMLW	-3.12	0.69	0	Bordetella resistance to killing- AAA51646_00877
17	VAIENAELLGASGMY	-3.09	0.67	0	putative autotransporter- AAC31207_00041
18	GGATTAWSVGMLAGY	-3.08	0.67	0	Vag8 protein (Autotr.) CAD12828 Q8VV95_00001
19	SMEHLPADAPLTPVR	-3.04	0.69	0	putative autotransporter- AAC31207_00181

PAM

42 predictive peptides

Top 20

	id	X1.score	X2.score	Protein
1	ADSTLQGAGGVQIER	0.86	-0.86	P.69A protein (pertactin)- CAA09473_00153
2	IDVFIDSGAQWRGMT	-0.54	0.54	putative autotransporter- AAC31207_00297
3	FLSLGKGFASLMDEP	-0.44	0.44	Bif. hemolysin-adenylate cyclaseprec- P15318_01501

4	GSAATFTLANKDGKV	0.39	-0.39	P.69A protein (pertactin)- CAA09473_00533
5	YSRLRKMLYLIYVAG	0.3	-0.3	Pertussis toxin subunit 2 prec- P04978_00173
6	SVRVHVSKEEQYYDY	0.25	-0.25	Pertussis toxin subunit 2 prec- P04978_00189
7	HFIGYIYEVRADNNF	0.22	-0.22	Pertussis toxin subunit 1 prec- O69258_00117
8	MRCTRAIRQTARTGW	0.2	-0.2	Pertussis toxin subunit 1 prec- O69258_00001
9	GRCPNGTRALTVAEL	0.19	-0.19	Pertussis toxin subunit 3 prec- P04979_00049
10	AEAGRFKVLTVNTLA	0.16	-0.16	P.69A protein (pertactin)- CAA09473_00465
11	GGVGLASTLWYAESN	0.16	-0.16	P.69A protein (pertactin)- CAA09473_00617
12	ARASTPGIVIPPQEQ	0.14	-0.14	Pertussis toxin subunit 2 prec- P04978_00025
13	LYVAGEQAQASIADS	0.13	-0.13	P.69A protein (pertactin)- CAA09473_00141
14	GVESLTLVELPEGSQ	0.12	-0.12	putative autotransporter- AAC31207_00405
15	GGMDAETKEVDGIIQ	0.12	-0.12	filamentous hemagglutinin- AAA22974_02057
16	PGSLIAEVQENIDNK	0.11	-0.11	filamentous hemagglutinin- AAA22974_01705
17	GASNVFGSWQMVDPK	-0.11	0.11	Out. M.porin protein prec-CAA41398.1 Q04064_00001
18	GLGILADLGAGRVDK	-0.11	0.11	Bif. hemolysin-adenylate cyclaseprec- P15318_01369
19	VLAAGAGLTLSNGAI	0.1	-0.1	filamentous hemagglutinin- AAA22974_02165
20	LSIDNATWVMTDNSN	0.09	-0.09	P.69A protein (pertactin)- CAA09473_00433

PAM cluster – 42 peptides

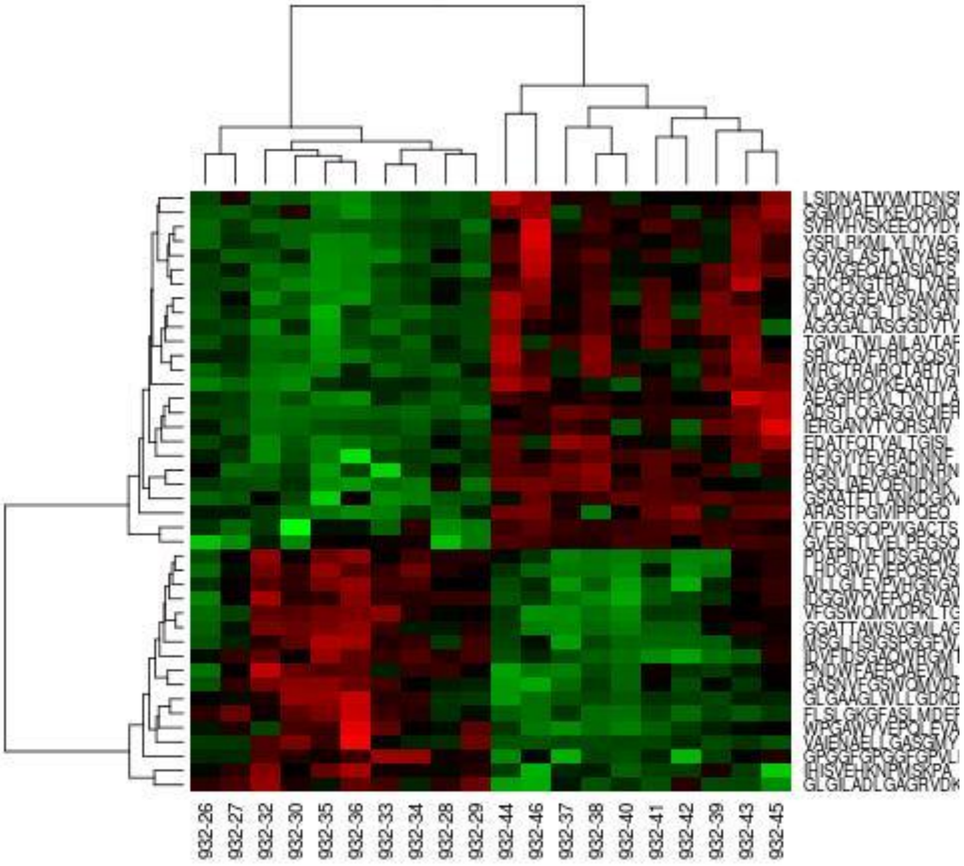

SAM

Infected vs Placebo

94 overresponding - 135 underresponding

top 20 Over

	Gene.ID	Score.d.	FC	q.value...	Protein
1	PGSLIAEVQENIDNK	7.91	1.39	0	filamentous hemagglutinin- AAA22974_01705
2	QSIVEAPELGAAIRV	6.27	1.34	0	P.69A protein (pertactin)- CAA09473_00309
3	LWAAGELTVKAQNIT	5.87	1.27	0	filamentous hemagglutinin- AAA22974_01745
4	PLRRTTLAMALGALG	5.82	1.31	0	P.69A protein (pertactin)- CAA09473_00013
5	TWFDTMLGFAISAYA	5.81	1.21	0	Pertussis toxin subunit 5 prec- P04981_00085

6	RVRALAWLLASGAMT	5.4	1.2	0	Pertussis toxin subunit 4 prec- P0A3R5_00021
7	GGNARLTAAVALLNK	5.35	1.23	0	filamentous hemagglutinin- AAA22974_01769
8	EAPELGAAIRVGRGA	5.27	1.33	0	P.69A protein (pertactin)- CAA09473_00313
9	HFIGYIYEVRADNNF	5.26	1.24	0	Pertussis toxin subunit 1 prec- O69258_00117
10	LGAVQARGALALDGG	5.19	1.24	0	filamentous hemagglutinin- AAA22974_00905
11	GGRAAGVAAMQGAVV	5.18	1.26	0	P.69A protein (pertactin)- CAA09473_00237
12	VLAAGAGLTLSNGAI	5.17	1.29	0	filamentous hemagglutinin- AAA22974_02165
13	APVAVVTSLLTGALN	5.14	1.52	0	Bif. hemolysin-adenylate cyclaseprec- P15318_00693
14	WSERAGEAMVLVYYE	5.09	1.25	0	Pertussis toxin subunit 1 prec- O69258_00249
15	ALTVAELRGNAELQT	5.04	1.21	0	Pertussis toxin subunit 3 prec- P04979_00057
16	ALSIDSMTALGAIGV	5	1.16	0	filamentous hemagglutinin- AAA22974_00617
17	ALGAIGVQAGGSVSA	4.97	1.18	0	filamentous hemagglutinin- AAA22974_00625
18	QTARTGWLTWLAILA	4.93	1.24	0	Pertussis toxin subunit 1 prec- O69258_00009
19	AGNVLDIGGADINRN	4.86	1.25	0	filamentous hemagglutinin- AAA22974_02805
20	LKANPMHTIASILLS	4.81	1.23	0	Pertussis toxin subunit 5 prec- P04981_00009

Top 20 Under

	Gene.ID	Score.d.	FC	q.value...	Protein
1	FLSLGKGFASLMDEP	-9.23	0.46	0	Bif. hemolysin-adenylate cyclaseprec- P15318_01501
2	GRRFDQKVAGFELGA	-8.86	0.59	0	P.69A protein (pertactin)- CAA09473_00665
3	FAADLRTVYAKQADQ	-8.46	0.64	0	filamentous hemagglutinin- AAA22974_02073
4	FKVAGSDGYAVKGKY	-8.39	0.55	0	P.69A protein (pertactin)- CAA09473_00745
5	SEHCTVGNTFCGRTR	-7.6	0.62	0	filamentous hemagglutinin- AAA22974_00021
6	SIWYAEGNALSKRLG	-7.58	0.7	0	putative autotransporter- AAC31207_00473
7	SPDAHVPFCFGKDLK	-7.52	0.47	0	Pertussis toxin subunit 4 prec- P0A3R5_00085
8	AGKRGAEIAFYPKEQ	-7.24	0.6	0	filamentous hemagglutinin- AAA22974_02149
9	VYGFELGADHAIAGQ	-7.24	0.58	0	putative autotransporter- AAC31207_00521
10	TENVQYRHVELARVG	-7.17	0.57	0	Bif. hemolysin-adenylate cyclaseprec- P15318_00985

11	TYTINRREDLMKLNG	-7.13	0.56	0	filamentous hemagglutinin- AAA22974_03541
12	VRGMLVPVSEHCTVG	-7.1	0.7	0	filamentous hemagglutinin- AAA22974_00013
13	PGVSGASAHWGQRAL	-6.9	0.52	0	Bif. hemolysin-adenylate cyclaseprec- P15318_00449
14	DAAQATVVQRNKHWA	-6.83	0.62	0	filamentous hemagglutinin- AAA22974_03241
15	SMEHLPADAPLTPVR	-6.69	0.51	0	putative autotransporter- AAC31207_00181
16	GVDARGDIVTLPPSA	-6.44	0.49	0	putative autotransporter- AAC31207_00225
17	TFCGRTRGQARSGAR	-6.38	0.58	0	filamentous hemagglutinin- AAA22974_00029
18	YASYEYAAGDRINIP	-6.29	0.68	0	Bordetella resistance to killing- AAA51646_00985
19	HSIKLDVIGGDGDDV	-6.25	0.72	0	Bif. hemolysin-adenylate cyclaseprec- P15318_00905
20	CFGKDLKRPGSSPME	-6.21	0.53	0	Pertussis toxin subunit 4 prec- P0A3R5_00093

PAM

32 predictive peptides

Top 20

	id	X1.score	X2.score	Protein
1	FLSLGKGFASLMDEP	-0.64	0.64	Bif. hemolysin-adenylate cyclaseprec- P15318_01501
2	GRRFDQKVAGFELGA	-0.46	0.46	P.69A protein (pertactin)- CAA09473_00665
3	FKVAGSDGYAVKGKY	-0.43	0.43	P.69A protein (pertactin)- CAA09473_00745
4	SPDAHVPFCFGKDLK	-0.37	0.37	Pertussis toxin subunit 4 prec- P0A3R5_00085
5	FAADLRTVYAKQADQ	-0.35	0.35	filamentous hemagglutinin- AAA22974_02073
6	SEHCTVGNTFCGRTR	-0.26	0.26	filamentous hemagglutinin- AAA22974_00021
7	TENVQYRHVELARVG	-0.25	0.25	Bif. hemolysin-adenylate cyclaseprec- P15318_00985
8	TYTINRREDLMKLNG	-0.25	0.25	filamentous hemagglutinin- AAA22974_03541
9	PGVSGASAHWGQRAL	-0.25	0.25	Bif. hemolysin-adenylate cyclaseprec- P15318_00449
10	VYGFELGADHAIAGQ	-0.25	0.25	putative autotransporter- AAC31207_00521
11	AGKRGAEIAFYPKEQ	-0.23	0.23	filamentous hemagglutinin- AAA22974_02149
12	SMEHLPADAPLTPVR	-0.22	0.22	putative autotransporter- AAC31207_00181

13	GVDARGDIVTLPPSA	-0.19	0.19	putative autotransporter- AAC31207_00225
14	SIWYAEGNALSKRLG	-0.17	0.17	putative autotransporter- AAC31207_00473
15	PGSLIAEVQENIDNK	0.17	-0.17	filamentous hemagglutinin- AAA22974_01705
16	AVVHLQRATIRRGDA	-0.17	0.17	P.69A protein (pertactin)- CAA09473_00249
17	DAAQATVVQRNKHWA	-0.16	0.16	filamentous hemagglutinin- AAA22974_03241
18	GGATTAWSVGMLAGY	-0.13	0.13	Vag8 protein (Autotr.) CAD12828 Q8VV95_00001
19	CFGKDLKRPGSSPME	-0.13	0.13	Pertussis toxin subunit 4 prec- P0A3R5_00093
20	TFCGRTRGQARSGAR	-0.13	0.13	filamentous hemagglutinin- AAA22974_00029

PAM cluster – 32 peptides

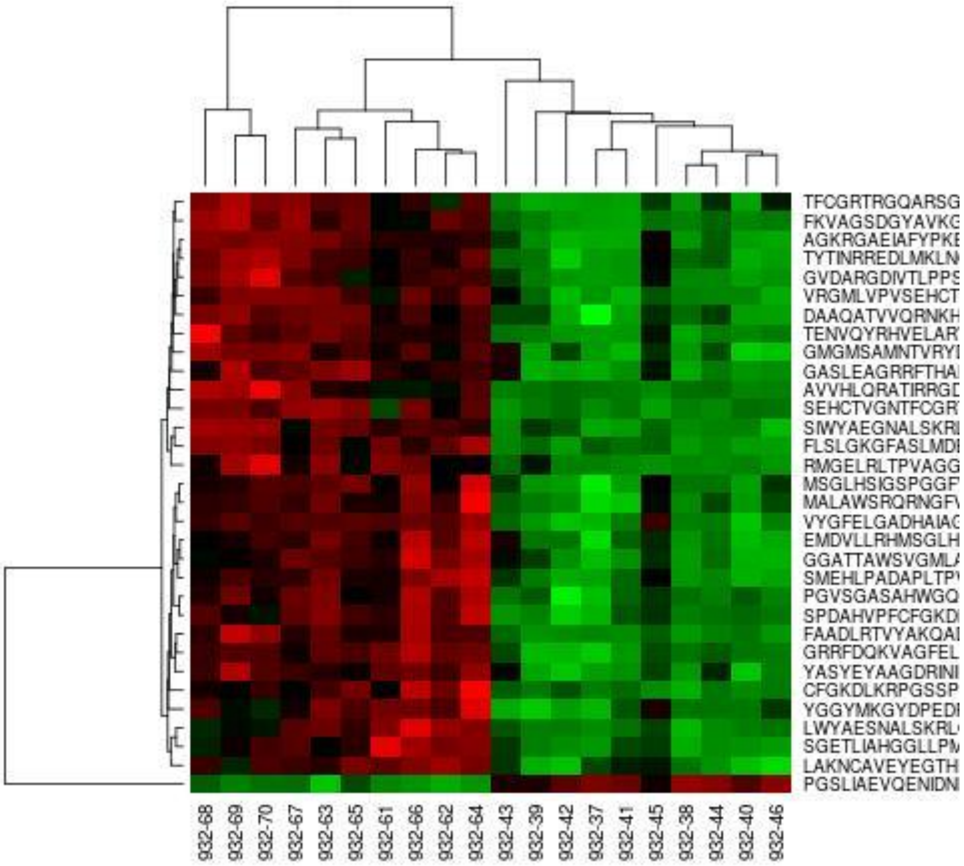

2) Each group compared to INFECTED individuals

SAM

2c vs Infected

80 overresponding - 13 underresponding

top 20 Over

	Gene.ID	Score.d.	Fold. Change	q.value.	Protein
1	GVGYGHDTIYESGGG	7.69	2.02	0	Bif. hemolysin-adenylate cyclaseprec- P15318_01593
2	TFCGRTRGQARSGAR	6.76	1.91	0	filamentous hemagglutinin- AAA22974_00029
3	AVVHLQRATIRRGDA	6.43	2.74	0	P.69A protein (pertactin)- CAA09473_00249
4	ARASTPGIVIPPQEQ	6.3	2.54	0	Pertussis toxin subunit 2 prec- P04978_00025
5	AKVEVVPRPKVETAQ	6.23	1.81	0	filamentous hemagglutinin- AAA22974_03353
6	GASLEAGRRFTHADG	6.02	1.68	0	P.69A protein (pertactin)- CAA09473_00765
7	FAADLRTVYAKQADQ	5.75	1.61	0	filamentous hemagglutinin- AAA22974_02073
8	TELAVGVASQLGKHG	5.41	1.74	0	tracheal colon factor- CAA08832. O86135_00001
9	YFFEQIGYKPDRAAR	5.31	1.53	0	filamentous hemagglutinin- AAA22974_02381
10	DGTAAGAMYGKHITL	5.13	1.6	0	filamentous hemagglutinin- AAA22974_00289
11	MQRQAGLPLKANPMH	4.95	2.18	0	Pertussis toxin subunit 5 prec- P04981_00001
12	LEKRLDIDDALAAVL	4.85	1.59	0	filamentous hemagglutinin- AAA22974_02217
13	CFGKDLKRPGSSPME	4.79	1.88	0	Pertussis toxin subunit 4 prec- P0A3R5_00093
14	FKVAGSDGYAVKGKY	4.36	1.76	0	P.69A protein (pertactin)- CAA09473_00745
15	GVDARGDIVTLPPSA	4.35	1.97	0	putative autotransporter- AAC31207_00225
16	GRRFDQKVAGFELGA	4.33	1.53	0	P.69A protein (pertactin)- CAA09473_00665

17	RLNPDAGGAWGRGF A	4.17	1.51	0	P.69A protein (pertactin)- CAA09473_00641
18	KSYRNANLNFGGGSV	4	1.41	0	filamentous hemagglutinin- AAA22974_02789
19	HSIKLDVIGGDGDDV	3.98	1.48	0	Bif. hemolysin-adenylate cyclaseprec- P15318_00905
20	GVESLTLVELPEGSQ	3.79	1.84	0	putative autotransporter- AAC31207_00405

top 13 Under

	Gene.ID	Score.d.	Fold.Change	q.value...	Protein
1	SADLSDGDLLVVSDE	-5.42	0.69	0	putative autotransporter- AAC31207_00373
2	TTIDLAKVVSQLVDA	-4.88	0.64	0	Bif. hemolysin-adenylate cyclaseprec- P15318_00885
3	QKQQLDNKAGRRFDQ	-4.21	0.76	0	putative autotransporter- AAC31207_00505
4	TPTNELSTTATAAVN	-4.09	0.66	0	Vag8 protein (Autotr.) CAD12828 Q8VV95_00001
5	DYYDNVRNVENVIGT	-3.95	0.72	0	Bif. hemolysin-adenylate cyclaseprec- P15318_01249
6	ASNGLRVRADGAHSW	-3.76	0.74	0	Vag8 protein (Autotr.) CAD12828 Q8VV95_00001
7	AANNLSVKDEGGTSA	-3.69	0.67	0	putative autotransporter- AAC31207_00649
8	ETRRDGGAYRAGHVH	-3.67	0.62	0	Vag8 protein (Autotr.) CAD12828 Q8VV95_00001
9	DNGDAVAYALWKLQD	-3.6	0.68	0	GTP-binding elongation factor- Q7VYR0_00493
10	TAQPLPPRPVAAQVV	-3.34	0.67	0	filamentous hemagglutinin- AAA22974_03329
11	NQAQGLRVGTENAPD	-3.27	0.79	0	Vag8 protein (Autotr.) CAD12828 Q8VV95_00001
12	WSERAGEAMVLVYYE	-3.14	0.68	0	Pertussis toxin subunit 1 prec- O69258_00249
13	VVTSLLTGALNGILR	-3.12	0.61	0	Bif. hemolysin-adenylate cyclaseprec- P15318_00697

PAM

29 predictive peptides

Top 20

	id	X1.score	X2.score	Protein
1	GVGYGHDTIYESGGG	0.54	-0.54	Bif. hemolysin-adenylate cyclaseprec- P15318_01593

2	AVVHLQRATIRRGDA	0.51	-0.51	P.69A protein (pertactin)- CAA09473_00249
3	ARASTPGIVIPPQEQ	0.47	-0.47	Pertussis toxin subunit 2 prec- P04978_00025
4	TFCGRTRGQARSGAR	0.41	-0.41	filamentous hemagglutinin- AAA22974_00029
5	AKVEVVPRPKVETAQ	0.33	-0.33	filamentous hemagglutinin- AAA22974_03353
6	GASLEAGRRFTHADG	0.27	-0.27	P.69A protein (pertactin)- CAA09473_00765
7	MQRQAGLPLKANPMH	0.25	-0.25	Pertussis toxin subunit 5 prec- P04981_00001
8	TELAVGVASQLGKHG	0.23	-0.23	tracheal colon factor- CAA08832. O86135_00001
9	FAADLRTVYAKQADQ	0.22	-0.22	filamentous hemagglutinin- AAA22974_02073
10	CFGKDLKRPGSSPME	0.19	-0.19	Pertussis toxin subunit 4 prec- P0A3R5_00093
11	DGTAAGAMYGKHITL	0.16	-0.16	filamentous hemagglutinin- AAA22974_00289
12	YFFEQIGYKPDRAAR	0.15	-0.15	filamentous hemagglutinin- AAA22974_02381
13	GVDARGDIVTLPPSA	0.15	-0.15	putative autotransporter- AAC31207_00225
14	LEKRLDIDDALAAVL	0.13	-0.13	filamentous hemagglutinin- AAA22974_02217
15	TTIDLAKVVSQLVDA	-0.12	0.12	Bif. hemolysin-adenylate cyclaseprec- P15318_00885
16	SADLSDGDLLVVSDE	-0.12	0.12	putative autotransporter- AAC31207_00373
17	FKVAGSDGYAVKGKY	0.11	-0.11	P.69A protein (pertactin)- CAA09473_00745
18	WQMVDPKLTGGDEKM	0.06	-0.06	Out. M.porin protein prec-CAA41398.1 Q04064_00001
19	GVESLTLVELPEGSQ	0.05	-0.05	putative autotransporter- AAC31207_00405
20	GRRFDQKVAGFELGA	0.05	-0.05	P.69A protein (pertactin)- CAA09473_00665

PAM cluster – 29 peptides

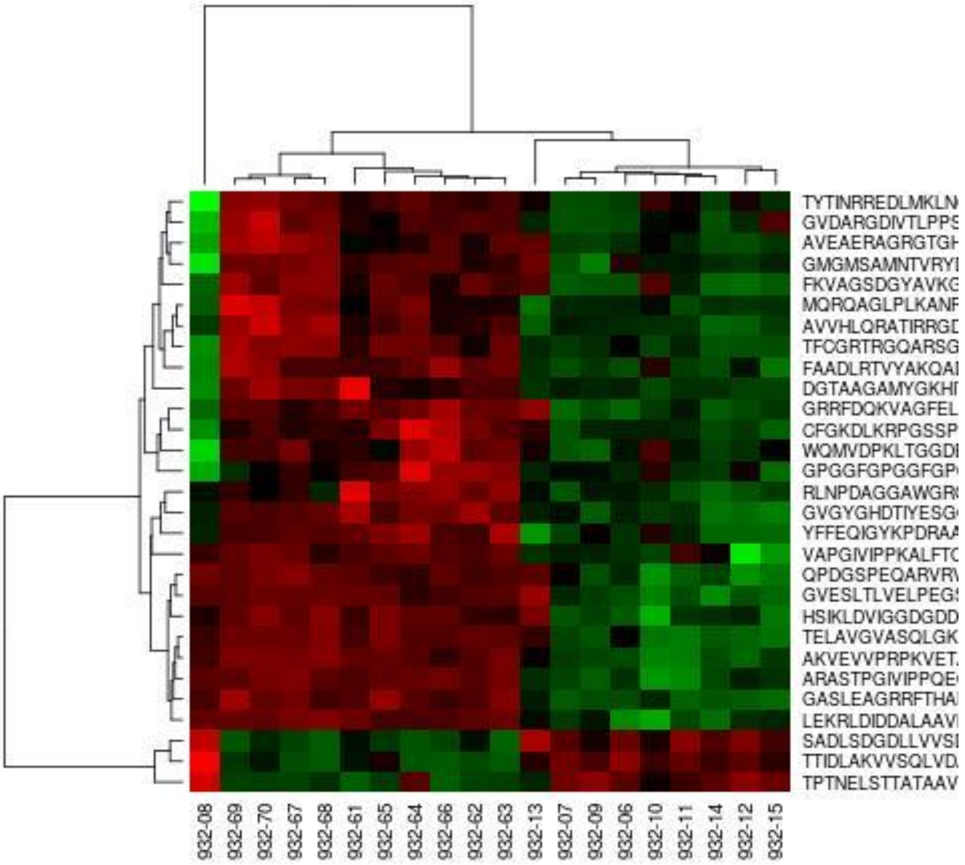

SAM

5c vs Infected

161 overresponding - 23 underresponding

top 20 Over

	Gene.ID	Score	Fold.Change	q.value	Protein
1	GASLEAGRRFTHADG	11.19	1.74	0	P.69A protein (pertactin)- CAA09473_00765
2	DAAQATVVQRNKHWA	10.9	2.13	0	filamentous hemagglutinin- AAA22974_03241
3	GVESLTLVELPEGSQ	10.61	2.16	0	putative autotransporter- AAC31207_00405
4	TFCGRTRGQARSGAR	10.12	2.03	0	filamentous hemagglutinin- AAA22974_00029
5	FKVAGSDGYAVKGKY	9.75	1.99	0	P.69A protein (pertactin)- CAA09473_00745

6	TYTINRREDLMKLNG	8.45	1.81	0	filamentous hemagglutinin- AAA22974_03541
7	FAADLRTVYAKQADQ	8.45	1.59	0	filamentous hemagglutinin- AAA22974_02073
8	TENVQYRHVELARVG	8.14	1.85	0	Bif. hemolysin-adenylate cyclaseprec- P15318_00985
9	SEHCTVGNTFCGRTR	8.01	1.71	0	filamentous hemagglutinin- AAA22974_00021
10	AESTFESYSESHNFH	7.93	1.73	0	filamentous hemagglutinin- AAA22974_02981
11	GRRFDQKVAGFELGA	7.44	1.53	0	P.69A protein (pertactin)- CAA09473_00665
12	AGKRGAEIAFYPKEQ	7.26	1.75	0	filamentous hemagglutinin- AAA22974_02149
13	GVDARGDIVTLPPSA	7.19	2.11	0	putative autotransporter- AAC31207_00225
14	AIIAHVDHGKTTLVD	7.1	1.39	0	GTP-binding elongation factor- Q7VYR0_00009
15	YFFEQIGYKPDRAAR	6.65	1.54	0	filamentous hemagglutinin- AAA22974_02381
16	AVVHLQRATIRRGDA	6.42	2.37	0	P.69A protein (pertactin)- CAA09473_00249
17	AVEAERAGRGTGHFI	6.27	1.85	0	Pertussis toxin subunit 1 prec- O69258_00105
18	LWYAESNALSKRLGE	6.22	1.65	0	P.69A protein (pertactin)- CAA09473_00625
19	SYAKNFAFLEDAKST	6.08	1.54	0	Out. M.porin protein prec-CAA41398.1 Q04064_00001
20	RARRSFIDDGAGHTD	6	1.35	0	putative autotransporter- AAC31207_00549

top 20 Under

	Gene.ID	Score.d.	Fold.Change	q.value...	Protein
1	TTIDLAKVVSQLVDA	-5.77	0.7	0	Bif. hemolysin-adenylate cyclaseprec- P15318_00885
2	ADGNKPLDAGISLSV	-5.38	0.76	0	Bordetella resistance to killing- AAA51646_00537
3	SADLSDGDLLVVSDE	-5.04	0.78	0	putative autotransporter- AAC31207_00373
4	YIKASVLQEFDGAGT	-4.41	0.82	0	P.69A protein (pertactin)- CAA09473_00837
5	ISVLGFEPQSGSGPA	-4.29	0.64	0	Bordetella resistance to killing- AAA51646_00397
6	NLLDGGDGRDTVDFS	-4.29	0.82	0	Bif. hemolysin-adenylate cyclaseprec- P15318_01473
7	VNGIEDLHIGSTITD	-4.04	0.73	0	GTP-binding elongation factor- Q7VYR0_00277
8	GRIRAGEDMHLDAPR	-3.97	0.92	0	filamentous hemagglutinin- AAA22974_01785
9	AIALAAQVTQRGGAA	-3.96	0.89	0	filamentous hemagglutinin- AAA22974_01413

10	ELSTTATAAVNAMAI	-3.95	0.83	0	Vag8 protein (Autotr.) CAD12828 Q8VV95_00001
11	ALGAIGVQAGGSVSA	-3.94	0.87	0	filamentous hemagglutinin- AAA22974_00625
12	VTLGSVASDGALSVS	-3.94	0.89	0	filamentous hemagglutinin- AAA22974_00485
13	RPLFETRIKFIDQSK	-3.76	0.82	0	filamentous hemagglutinin- AAA22974_02361
14	ADGWFLEPQAELAVF	-3.76	0.88	0	P.69A protein (pertactin)- CAA09473_00777
15	NGGNGNAQLPERGDD	-3.75	0.86	0	tracheal colon factor- CAA08832. O86135_00001
16	APQAAPLSITLQAGA	-3.75	0.75	0	P.69A protein (pertactin)- CAA09473_00353
17	GTHTLTTPASAAVSL	-3.72	0.92	0	tracheal colon factor- CAA08832. O86135_00001
18	PGSLIAEVQENIDNK	-3.71	0.87	0	filamentous hemagglutinin- AAA22974_01705
19	IFLQDDETVSDDIDG	-3.7	0.89	0	Bif. hemolysin-adenylate cyclaseprec- P15318_01189
20	KISQSVLAAKGDKGK	-3.7	0.9	0	filamentous hemagglutinin- AAA22974_01149

PAM

43 predictive peptides

Top 20

	id	X1.score	X2.score	Protein
1	DAAQATVVQRNKHWA	0.99	-0.99	filamentous hemagglutinin- AAA22974_03241
2	GVESLTLVELPEGSQ	0.96	-0.96	putative autotransporter- AAC31207_00405
3	GASLEAGRRFTHADG	0.88	-0.88	P.69A protein (pertactin)- CAA09473_00765
4	TFCGRTRGQARSGAR	0.86	-0.86	filamentous hemagglutinin- AAA22974_00029
5	FKVAGSDGYAVKGKY	0.8	-0.8	P.69A protein (pertactin)- CAA09473_00745
6	TYTINRREDLMKLNG	0.58	-0.58	filamentous hemagglutinin- AAA22974_03541
7	TENVQYRHVELARVG	0.55	-0.55	Bif. hemolysin-adenylate cyclaseprec- P15318_00985
8	FAADLRTVYAKQADQ	0.49	-0.49	filamentous hemagglutinin- AAA22974_02073
9	SEHCTVGNTFCGRTR	0.49	-0.49	filamentous hemagglutinin- AAA22974_00021

10	AESTFESYSESHNFH	0.49	-0.49	filamentous hemagglutinin- AAA22974_02981
11	GVDARGDIVTLPPSA	0.47	-0.47	putative autotransporter- AAC31207_00225
12	AGKRGAEIAFYPKEQ	0.4	-0.4	filamentous hemagglutinin- AAA22974_02149
13	AVVHLQRATIRRGDA	0.37	-0.37	P.69A protein (pertactin)- CAA09473_00249
14	GRRFDQKVAGFELGA	0.35	-0.35	P.69A protein (pertactin)- CAA09473_00665
15	AVEAERAGRGTGHFI	0.29	-0.29	Pertussis toxin subunit 1 prec- O69258_00105
16	YFFEQIGYKPDRAAR	0.25	-0.25	filamentous hemagglutinin- AAA22974_02381
17	LWYAESNALSKRLGE	0.23	-0.23	P.69A protein (pertactin)- CAA09473_00625
18	ARASTPGIVIPPQEQ	0.23	-0.23	Pertussis toxin subunit 2 prec- P04978_00025
19	AIIAHVDHGKTTLVD	0.22	-0.22	GTP-binding elongation factor- Q7VYR0_00009
20	PGVSGASAHWGQRAL	0.2	-0.2	Bif. hemolysin-adenylate cyclaseprec- P15318_00449

PAM cluster – 43 peptides

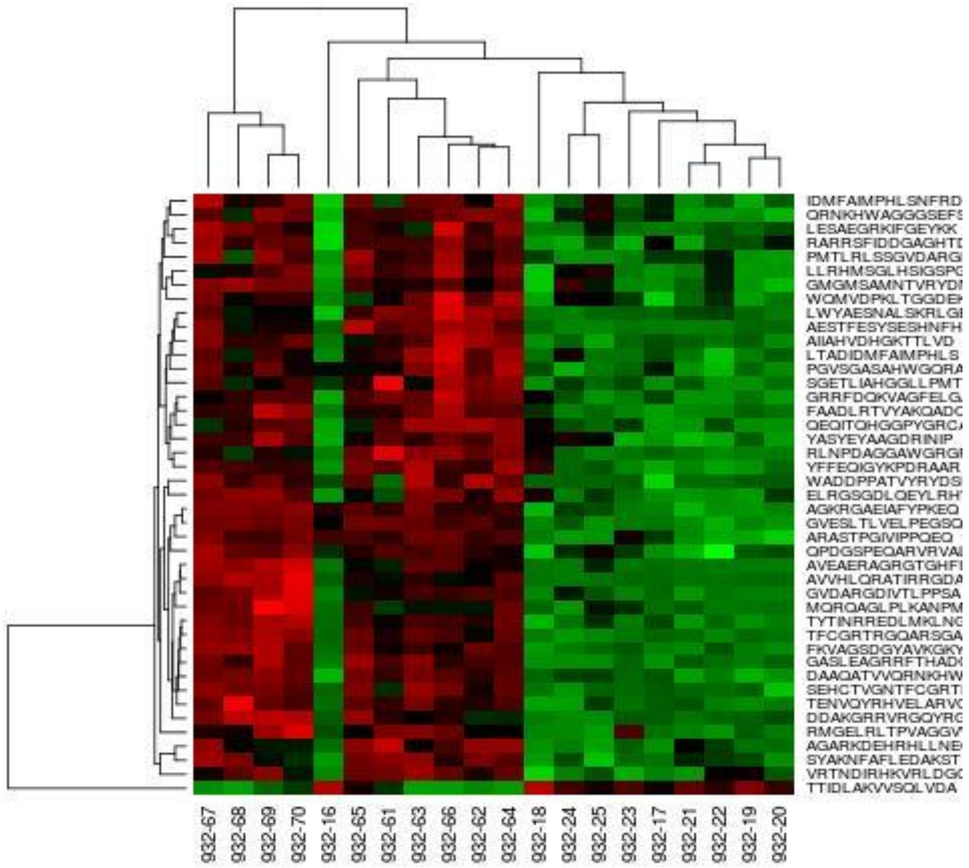

SAM

WH vs Infected

163 overresponding - 24 underresponding

top 20 Over

	Gene.ID	Score.d.	Fold.Change	q.value...	Protein
1	GASLEAGRRFTHADG	11.19	1.74	0	P.69A protein (pertactin)- CAA09473_00765
2	DAAQATVVQRNKHWA	10.9	2.13	0	filamentous hemagglutinin- AAA22974_03241
3	GVESLTLVELPEGSQ	10.61	2.16	0	putative autotransporter- AAC31207_00405
4	TFCGRTRGQARSGAR	10.12	2.03	0	filamentous hemagglutinin- AAA22974_00029
5	FKVAGSDGYAVKGKY	9.75	1.99	0	P.69A protein (pertactin)- CAA09473_00745
6	TYTINRREDLMKLNG	8.45	1.81	0	filamentous hemagglutinin- AAA22974_03541
7	FAADLRTVYAKQADQ	8.45	1.59	0	filamentous hemagglutinin- AAA22974_02073
8	TENVQYRHVELARVG	8.14	1.85	0	Bif. hemolysin-adenylate cyclaseprec- P15318_00985
9	SEHCTVGNTFCGRTR	8.01	1.71	0	filamentous hemagglutinin- AAA22974_00021
10	AESTFESYSESHNFH	7.93	1.73	0	filamentous hemagglutinin- AAA22974_02981
11	GRRFDQKVAGFELGA	7.44	1.53	0	P.69A protein (pertactin)- CAA09473_00665
12	AGKRGAEIAFYPKEQ	7.26	1.75	0	filamentous hemagglutinin- AAA22974_02149
13	GVDARGDIVTLPPSA	7.19	2.11	0	putative autotransporter- AAC31207_00225
14	AIIAHVDHGKTTLVD	7.1	1.39	0	GTP-binding elongation factor- Q7VYR0_00009
15	YFFEQIGYKPDRAAR	6.65	1.54	0	filamentous hemagglutinin- AAA22974_02381
16	AVVHLQRATIRRGDA	6.42	2.37	0	P.69A protein (pertactin)- CAA09473_00249
17	AVEAERAGRGTGHFI	6.27	1.85	0	Pertussis toxin subunit 1 prec- O69258_00105
18	LWYAESNALSKRLGE	6.22	1.65	0	P.69A protein (pertactin)- CAA09473_00625
19	SYAKNFAFLEDAKST	6.08	1.54	0	Out. M.porin protein prec-CAA41398.1 Q04064_00001

20	RARRSFIDDGAGHTD	6	1.35	0	putative autotransporter- AAC31207_00549

top 20 Under

	Gene.ID	Score.d.	Fold.Change	q.value...	Protein
1	TTIDLAKVVSQLVDA	-5.77	0.7	0	Bif. hemolysin-adenylate cyclaseprec- P15318_00885
2	ADGNKPLDAGISLSV	-5.38	0.76	0	Bordetella resistance to killing- AAA51646_00537
3	SADLSDGDLLVVSDE	-5.04	0.78	0	putative autotransporter- AAC31207_00373
4	YIKASVLQEFDGAGT	-4.41	0.82	0	P.69A protein (pertactin)- CAA09473_00837
5	ISVLGFEPQSGSGPA	-4.29	0.64	0	Bordetella resistance to killing- AAA51646_00397
6	NLLDGGDGRDTVDFS	-4.29	0.82	0	Bif. hemolysin-adenylate cyclaseprec- P15318_01473
7	VNGIEDLHIGSTITD	-4.04	0.73	0	GTP-binding elongation factor- Q7VYR0_00277
8	GRIRAGEDMHLDAPR	-3.97	0.92	0	filamentous hemagglutinin- AAA22974_01785
9	AIALAAQVTQRGGAA	-3.96	0.89	0	filamentous hemagglutinin- AAA22974_01413
10	ELSTTATAAVNAMAI	-3.95	0.83	0	Vag8 protein (Autotr.) CAD12828 Q8VV95_00001
11	ALGAIGVQAGGSVSA	-3.94	0.87	0	filamentous hemagglutinin- AAA22974_00625
12	VTLGSVASDGALSVS	-3.94	0.89	0	filamentous hemagglutinin- AAA22974_00485
13	RPLFETRIKFIDQSK	-3.76	0.82	0	filamentous hemagglutinin- AAA22974_02361
14	ADGWFLEPQAELAVF	-3.76	0.88	0	P.69A protein (pertactin)- CAA09473_00777
15	NGGNGNAQLPERGDD	-3.75	0.86	0	tracheal colon factor- CAA08832. O86135_00001
16	APQAAPLSITLQAGA	-3.75	0.75	0	P.69A protein (pertactin)- CAA09473_00353
17	GTHTLTTPASAAVSL	-3.72	0.92	0	tracheal colon factor- CAA08832. O86135_00001
18	PGSLIAEVQENIDNK	-3.71	0.87	0	filamentous hemagglutinin- AAA22974_01705
19	IFLQDDETVSDDIDG	-3.7	0.89	0	Bif. hemolysin-adenylate cyclaseprec- P15318_01189
20	KISQSVLAAKGDKGK	-3.7	0.9	0	filamentous hemagglutinin- AAA22974_01149

PAM

42 predictive peptides

Top 20

	id	X1.score	X2.score	Protein
1	TFCGRTRGQARSGAR	0.82	-0.82	filamentous hemagglutinin- AAA22974_00029
2	YFFEQIGYKPDRAAR	0.78	-0.78	filamentous hemagglutinin- AAA22974_02381
3	GASLEAGRRFTHADG	0.76	-0.76	P.69A protein (pertactin)- CAA09473_00765
4	GGMDAETKEVDGIIQ	0.67	-0.67	filamentous hemagglutinin- AAA22974_02057
5	FAADLRTVYAKQADQ	0.53	-0.53	filamentous hemagglutinin- AAA22974_02073
6	ELRGSGDLQEYLRHV	0.53	-0.53	Pertussis toxin subunit 2 prec- P04978_00061
7	SEHCTVGNTFCGRTR	0.49	-0.49	filamentous hemagglutinin- AAA22974_00021
8	RARRSFIDDGAGHTD	0.42	-0.42	putative autotransporter- AAC31207_00549
9	AVVHLQRATIRRGDA	0.39	-0.39	P.69A protein (pertactin)- CAA09473_00249
10	SVRVHVSKEEQYYDY	0.35	-0.35	Pertussis toxin subunit 2 prec- P04978_00189
11	AESTFESYSESHNFH	0.35	-0.35	filamentous hemagglutinin- AAA22974_02981
12	GRRFDQKVAGFELGA	0.33	-0.33	P.69A protein (pertactin)- CAA09473_00665
13	LWYAESNALSKRLGE	0.31	-0.31	P.69A protein (pertactin)- CAA09473_00625
14	VEIIHAANQAVDQAG	0.31	-0.31	Bif. hemolysin-adenylate cyclaseprec- P15318_01653
15	GASAHWGQRALQGAQ	0.3	-0.3	Bif. hemolysin-adenylate cyclaseprec- P15318_00453
16	RLNPDAGGAWGRGFA	0.29	-0.29	P.69A protein (pertactin)- CAA09473_00641
17	MRAKKVESTKYVSEQ	0.27	-0.27	filamentous hemagglutinin- AAA22974_02837
18	PGVSGASAHWGQRAL	0.26	-0.26	Bif. hemolysin-adenylate cyclaseprec- P15318_00449
19	YSRLRKMLYLIYVAG	0.26	-0.26	Pertussis toxin subunit 2 prec- P04978_00173
20	TYTINRREDLMKLNG	0.25	-0.25	filamentous hemagglutinin- AAA22974_03541

PAM cluster – 42 peptides

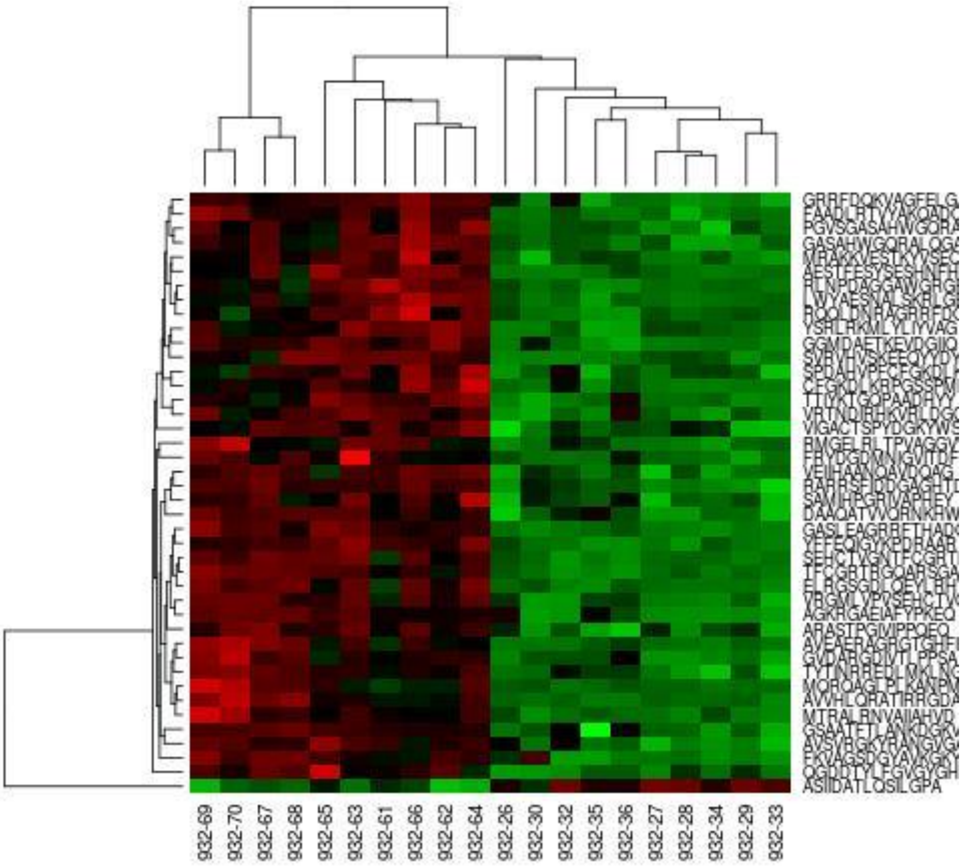

SAM

placebo vs Infected

129 overresponding - 78 underresponding

top 20 Over

	Gene.ID	Score.d.	Fold.Change	q.value...	Protein
1	FLSLGKGFASLMDEP	9.23	2.19	0	Bif. hemolysin-adenylate cyclaseprec- P15318_01501
2	GRRFDQKVAGFELGA	8.86	1.69	0	P.69A protein (pertactin)- CAA09473_00665
3	FAADLRTVYAKQADQ	8.46	1.56	0	filamentous hemagglutinin- AAA22974_02073
4	FKVAGSDGYAVKGKY	8.39	1.81	0	P.69A protein (pertactin)- CAA09473_00745

5	SEHCTVGNTFCGRTR	7.6	1.61	0	filamentous hemagglutinin- AAA22974_00021
6	SIWYAEGNALSKRLG	7.58	1.44	0	putative autotransporter- AAC31207_00473
7	SPDAHVPFCFGKDLK	7.52	2.11	0	Pertussis toxin subunit 4 prec- P0A3R5_00085
8	AGKRGAEIAFYPKEQ	7.24	1.67	0	filamentous hemagglutinin- AAA22974_02149
9	VYGFELGADHAIAGQ	7.24	1.71	0	putative autotransporter- AAC31207_00521
10	TENVQYRHVELARVG	7.17	1.76	0	Bif. hemolysin-adenylate cyclaseprec- P15318_00985
11	TYTINRREDLMKLNG	7.13	1.79	0	filamentous hemagglutinin- AAA22974_03541
12	VRGMLVPVSEHCTVG	7.1	1.42	0	filamentous hemagglutinin- AAA22974_00013
13	PGVSGASAHWGQRAL	6.9	1.94	0	Bif. hemolysin-adenylate cyclaseprec- P15318_00449
14	DAAQATVVQRNKHWA	6.83	1.61	0	filamentous hemagglutinin- AAA22974_03241
15	SMEHLPADAPLTPVR	6.69	1.97	0	putative autotransporter- AAC31207_00181
16	GVDARGDIVTLPPSA	6.44	2.06	0	putative autotransporter- AAC31207_00225
17	TFCGRTRGQARSGAR	6.38	1.73	0	filamentous hemagglutinin- AAA22974_00029
18	YASYEYAAGDRINIP	6.29	1.46	0	Bordetella resistance to killing- AAA51646_00985
19	HSIKLDVIGGDGDDV	6.25	1.38	0	Bif. hemolysin-adenylate cyclaseprec- P15318_00905
20	CFGKDLKRPGSSPME	6.21	1.9	0	Pertussis toxin subunit 4 prec- P0A3R5_00093

top 20 Under

	Gene.ID	Score.d.	Fold.Change	q.value...	Protein
1	PGSLIAEVQENIDNK	-7.91	0.72	0	filamentous hemagglutinin- AAA22974_01705
2	QSIVEAPELGAAIRV	-6.27	0.74	0	P.69A protein (pertactin)- CAA09473_00309
3	LWAAGELTVKAQNIT	-5.87	0.79	0	filamentous hemagglutinin- AAA22974_01745
4	PLRRTTLAMALGALG	-5.82	0.76	0	P.69A protein (pertactin)- CAA09473_00013
5	TWFDTMLGFAISAYA	-5.81	0.83	0	Pertussis toxin subunit 5 prec- P04981_00085
6	RVRALAWLLASGAMT	-5.4	0.83	0	Pertussis toxin subunit 4 prec- P0A3R5_00021
7	GGNARLTAAVALLNK	-5.35	0.81	0	filamentous hemagglutinin- AAA22974_01769
8	EAPELGAAIRVGRGA	-5.27	0.75	0	P.69A protein (pertactin)- CAA09473_00313
9	HFIGYIYEVRADNNF	-5.26	0.81	0	Pertussis toxin subunit 1 prec- O69258_00117

10	LGAVQARGALALDGG	-5.19	0.81	0	filamentous hemagglutinin- AAA22974_00905
11	GGRAAGVAAMQGAVV	-5.18	0.79	0	P.69A protein (pertactin)- CAA09473_00237
12	VLAAGAGLTLSNGAI	-5.17	0.78	0	filamentous hemagglutinin- AAA22974_02165
13	APVAVVTSLLTGALN	-5.14	0.66	0	Bif. hemolysin-adenylate cyclaseprec- P15318_00693
14	WSERAGEAMVLVYYE	-5.09	0.8	0	Pertussis toxin subunit 1 prec- O69258_00249
15	ALTVAELRGNAELQT	-5.04	0.83	0	Pertussis toxin subunit 3 prec- P04979_00057
16	ALSIDSMTALGAIGV	-5	0.86	0	filamentous hemagglutinin- AAA22974_00617
17	ALGAIGVQAGGSVSA	-4.97	0.85	0	filamentous hemagglutinin- AAA22974_00625
18	QTARTGWLTWLAILA	-4.93	0.81	0	Pertussis toxin subunit 1 prec- O69258_00009
19	AGNVLDIGGADINRN	-4.86	0.8	0	filamentous hemagglutinin- AAA22974_02805
20	LKANPMHTIASILLS	-4.81	0.81	0	Pertussis toxin subunit 5 prec- P04981_00009

PAM

32 predictive peptides

Top 20

	id	X1.score	X2.score	Protein
1	FLSLGKGFASLMDEP	0.64	-0.64	Bif. hemolysin-adenylate cyclaseprec- P15318_01501
2	GRRFDQKVAGFELGA	0.46	-0.46	P.69A protein (pertactin)- CAA09473_00665
3	FKVAGSDGYAVKGKY	0.43	-0.43	P.69A protein (pertactin)- CAA09473_00745
4	SPDAHVPFCFGKDLK	0.37	-0.37	Pertussis toxin subunit 4 prec- P0A3R5_00085
5	FAADLRTVYAKQADQ	0.35	-0.35	filamentous hemagglutinin- AAA22974_02073
6	SEHCTVGNTFCGRTR	0.26	-0.26	filamentous hemagglutinin- AAA22974_00021
7	TENVQYRHVELARVG	0.25	-0.25	Bif. hemolysin-adenylate cyclaseprec- P15318_00985
8	TYTINRREDLMKLNG	0.25	-0.25	filamentous hemagglutinin- AAA22974_03541
9	PGVSGASAHWGQRAL	0.25	-0.25	Bif. hemolysin-adenylate cyclaseprec- P15318_00449
10	VYGFELGADHAIAGQ	0.25	-0.25	putative autotransporter- AAC31207_00521
11	AGKRGAEIAFYPKEQ	0.23	-0.23	filamentous hemagglutinin- AAA22974_02149

12	SMEHLPADAPLTPVR	0.22	-0.22	putative autotransporter- AAC31207_00181
13	GVDARGDIVTLPPSA	0.19	-0.19	putative autotransporter- AAC31207_00225
14	SIWYAEGNALSKRLG	0.17	-0.17	putative autotransporter- AAC31207_00473
15	PGSLIAEVQENIDNK	-0.17	0.17	filamentous hemagglutinin- AAA22974_01705
16	AVVHLQRATIRRGDA	0.17	-0.17	P.69A protein (pertactin)- CAA09473_00249
17	DAAQATVVQRNKHWA	0.16	-0.16	filamentous hemagglutinin- AAA22974_03241
18	GGATTAWSVGMLAGY	0.13	-0.13	Vag8 protein (Autotr.) CAD12828 Q8VV95_00001
19	CFGKDLKRPGSSPME	0.13	-0.13	Pertussis toxin subunit 4 prec- P0A3R5_00093
20	TFCGRTRGQARSGAR	0.13	-0.13	filamentous hemagglutinin- AAA22974_00029

PAM cluster – 32 peptides

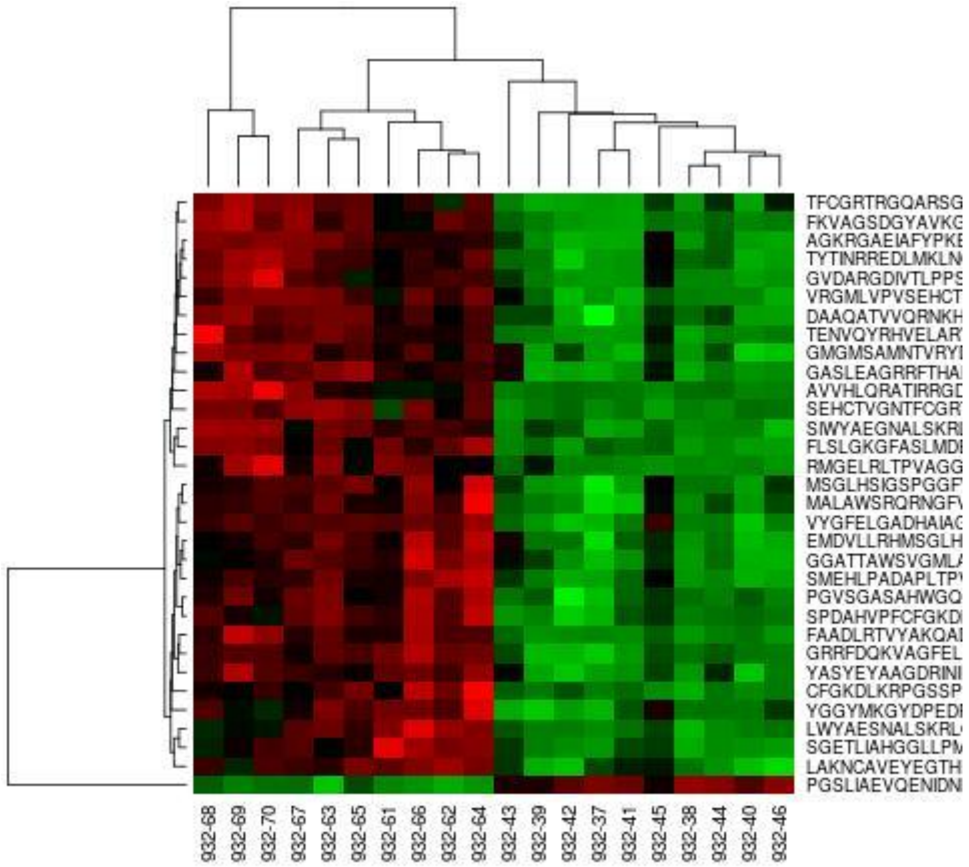

Table S3. B-cell epitopes from B. pertussis proteins retrieved from the B-cell database in Immune Epitope Database (http://www.immuneepitope.org/).

**Peptide**	**Source protein**	**Ab source**
VQVRI	Fim2	human sera
RISNLND	Fim2	human sera
VQTGGTSRTVTMRYLAS	Fim2	human sera
SYVK	Fim2	human sera
ITTYV	Fim2	human sera
GPNHTKV	Fim2	human sera
KVVQLPKISKNALKANG	Fim2	human sera
GRTPFII	Fim2	human sera
IKLKDCP	Fim2	human sera
VITGTI	Fim2	human sera
SSLGNGV	Fim2	human sera
YFEPGPT	Fim2	human sera
TGDLRAY	Fim2	human sera
KMVYATN	Fim2	human sera
QLSNIT	Fim2	human sera
SSATK	Fim3	human sera
AKGVEFR	Fim3	human sera
RLANLNG	Fim3	human sera
IRMGTDK	Fim3	human sera
GKVTNGS	Fim3	human sera
LRYLA	Fim3	human sera
ASYVKKPKEDVD	Fim3	human sera
TSYVG	Fim3	human sera
KVVQLPKISKNALRNDG	Fim3	human sera
KLKECPQ	Fim3	human sera
QALGALK	Fim3	human sera
LKLYFEP	Fim3	human sera
DLIAYKQ	Fim3	human sera
VITGSI	Fim3	human sera
GIRRFL	pertactin	MAb
TWDDD	pertactin	MAb
SLQPED	pertactin	MAb
GASEL	pertactin	MAb
GGAVP	pertactin	MAb
SITLQAGAH	pertactin	MAb
KALLYR	pertactin	MAb
TELPSIPG	pertactin	MAb
LAGSGL	pertactin	MAb

TFTLANK	pertactin	MAb
PAPQPP	pertactin	MAb
SNALSKRL	pertactin	MAb
DNRAG	pertactin	MAb
HLGGLAGY	pertactin	MAb
GILLEN	pertactin	MAb
YSKVTATBLLASTNSRLCAVFVRDG	Pertussis toxin subunit 1	human sera
LEHRMQEAVEAERAGRGTGHFI	Ptx S1	human sera
DDPPATVYRYDSRPPED	Ptx S1	human sera
RIPPENIRRVT	Ptx S1	human sera
SEYLAHRRIPPENIRRVTRV	Ptx S1	human sera
GITGETTTTEYSNARYV	Ptx S1	human sera
EAVEAERAGRGTG	Ptx S1	human sera
AFVSTSSSRRYTEVY	Ptx S1	human sera
LEHRMQEAVEAERAGRGTGHFI	Ptx S1	human sera
GAASSYFEYVDTYG	Ptx S1	human sera
QQTRANPNPYTSRRSVAS	Ptx S1	human sera
GTLVRMAPVIG	Ptx S1	human sera
AMAAWSERAGEA	Ptx S1	human sera
PPATVYRYDSRPPE	Ptx S1	human sera
DVFQNGFTAWGNND	Ptx S1	human sera
GAFDLKTTFCIMTTRNTGQPA	Ptx S2	human sera
AGFIYRETFCITTIYKTGQPAADHYYSKVTA	Ptx S3 pre	human sera
YYDYEDATFQTYALTGISLCNPAASIC	Ptx S3 pre	human sera
SGTIK	bapC	human sera

Table S4: Peptides recognized in all serum samples from infants in a group (10/10) containing B-cell epitopes retrieved from the B-cell database in Immune Epitope Database (http://www.immuneepitope.org/). * mutant peptides recognized among these proteins; interestingly the canonical sequence was not recognized. DT: Diphtheria Tetanus vaccine; DPTwc: Diphtheria Tetanus Pertussis whole cell vaccine; DTPa2: Diphtheria Tetanus Pertussis 2 components vaccine.

Groups	Peptide sequence	Source protein	Canonical peptide (not recognized)
DT	PGGAVPGGFGPVLDGWYGVD	prn13*	PGGAVPGGFGPGGFGPVLDGWYGVD
Whooping cough	PFIIKLKDCPSSLGN	Fim2	
	DAAQITSYVGFSVVY	Fim3	
	AAQITSYVGFSVVYP	Fim3	
	GAVPGGAVPGGFGPVLDGWY	prn13*	GAVPGGAVPGGFGP GGFGPVLDGWY
	PGGAVPGGFGPVLDGWYGVD	prn13*	PGGAVPGGFGPGGFGPVLDGWYGVD
	LWYAESNALSKRLGE	prn	
	TTIYKTGQPAADHYY	Ptx3	
	AVEAERAGRGTGHFI	Ptx1	
DTPa2	PAGGAVPGGFGPGGFGPGGF	Prn5*	PAGGAVPGGAVPGGFGPGGFGPGGFGPGGF
	GAVPGGAVPGGFGPVLDGWY	Prn13*	GAVPGGAVPGGFGP GGFGPVLDGWY
	PGGAVPGGFGPVLDGWYGVD	Prn13*	PGGAVPGGFGPGGFGPVLDGWYGVD
	PGGAVPGGFGPGGFGPGGFG	prn9*	GGGVPGGAVPGGFGPGGFG
DTPwc	PFIIKLKDCPSSLGN	Fim2*	
	GAVPGGAVPGGFGPVLDGWY	Prn13*	GAVPGGAVPGGFGP GGFGPVLDGWY
	PGGAVPGGFGPVLDGWYGVD	Prn13*	PGGAVPGGFGPGGFGPVLDGWYGVD
